# Structure and Function of the α‐Hydroxylation Bimodule of the Mupirocin Polyketide Synthase

**DOI:** 10.1002/ange.202312514

**Published:** 2023-10-16

**Authors:** Ashley J. Winter, R. Nisha Khanizeman, Abigail M. C. Barker‐Mountford, Andrew J. Devine, Luoyi Wang, Zhongshu Song, Jonathan A. Davies, Paul R. Race, Christopher Williams, Thomas J. Simpson, Christine L. Willis, Matthew P. Crump

**Affiliations:** ^1^ School of Chemistry University of Bristol Bristol BS8 1TS UK; ^2^ School of Biochemistry University of Bristol Bristol BS8 1TD UK; ^3^ current address School of Natural and Environmental Sciences Newcastle University Newcastle upon Tyne NE1 7RU UK; ^4^ Institute of Microbiology Chinese Academy of Sciences Beijing 100101 China

**Keywords:** Mupirocin, Natural Products, Polyketides, Pseudomonic Acid, Flavin Monooxygenases

## Abstract

Mupirocin is a clinically important antibiotic produced by a *trans*‐AT Type I polyketide synthase (PKS) in *Pseudomonas fluorescens*. The major bioactive metabolite, pseudomonic acid A (PA−A), is assembled on a tetrasubstituted tetrahydropyran (THP) core incorporating a 6‐hydroxy group proposed to be introduced by α‐hydroxylation of the thioester of the acyl carrier protein (ACP) bound polyketide chain. Herein, we describe an in vitro approach combining purified enzyme components, chemical synthesis, isotopic labelling, mass spectrometry and NMR in conjunction with in vivo studies leading to the first characterisation of the α‐hydroxylation bimodule of the mupirocin biosynthetic pathway. These studies reveal the precise timing of hydroxylation by MupA, substrate specificity and the ACP dependency of the enzyme components that comprise this α‐hydroxylation bimodule. Furthermore, using purified enzyme, it is shown that the MmpA KS^0^ shows relaxed substrate specificity, suggesting precise spatiotemporal control of *in trans* MupA recruitment in the context of the PKS. Finally, the detection of multiple intermodular MupA/ACP interactions suggests these bimodules may integrate MupA into their assembly.

## Graphical abstract

Mupirocin is a clinically important polyketide antibiotic bearing a 6‐hydroxy group that is essential for biological activity. NMR spectroscopy, authentic synthetic substrates and mass spectrometry were used to establish the mechanistic steps governing incorporation of this key 6‐hydroxy group by an α‐hydroxylation bimodule that recruits a *trans* acting monooxygenase, MupA.

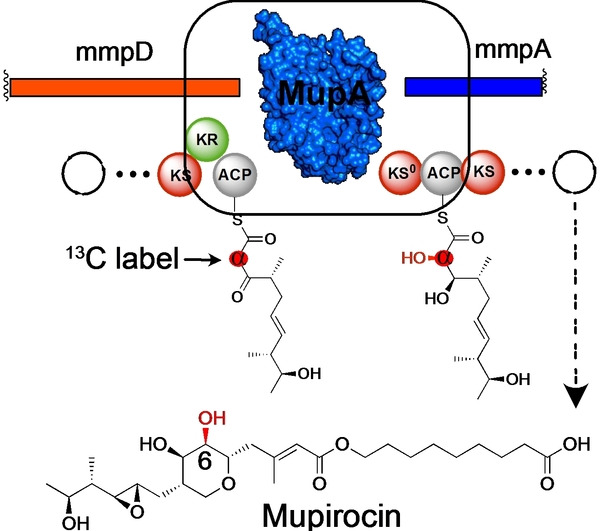

## Introduction

Polyketides are a structurally diverse class of natural products that have been exploited in the production of numerous high‐value compounds including antibiotic, antifungal and anticancer therapeutics. Their basic assembly on large, multifunctional and multimodular polyketide synthases (PKSs) stems from a controlled series of Claisen condensations of malonyl‐CoA that recursively extend the acyl carrier protein (ACP) bound polyketide thioester by two carbon atoms. Structural diversification, essential for bioactivity, is introduced by reduction and dehydration during assembly as well as tailoring steps that can include cyclisation, methylation, epoxidation and hydroxylation. The incorporation of a hydroxy group at the α carbon of the thioester, known as α‐hydroxylation, is observed in many biologically active polyketide natural products. There have, however, only been a limited number of studies on the timing and mechanism of this modification in the context of a PKS and these include the zwittermicin A and oocydin biosynthetic pathways.^[1]^ Herein we focus on the mupirocin biosynthetic pathway to gain further insights into the timing and mechanism of α‐hydroxylation in polyketide biosynthesis.

**Figure 1:**
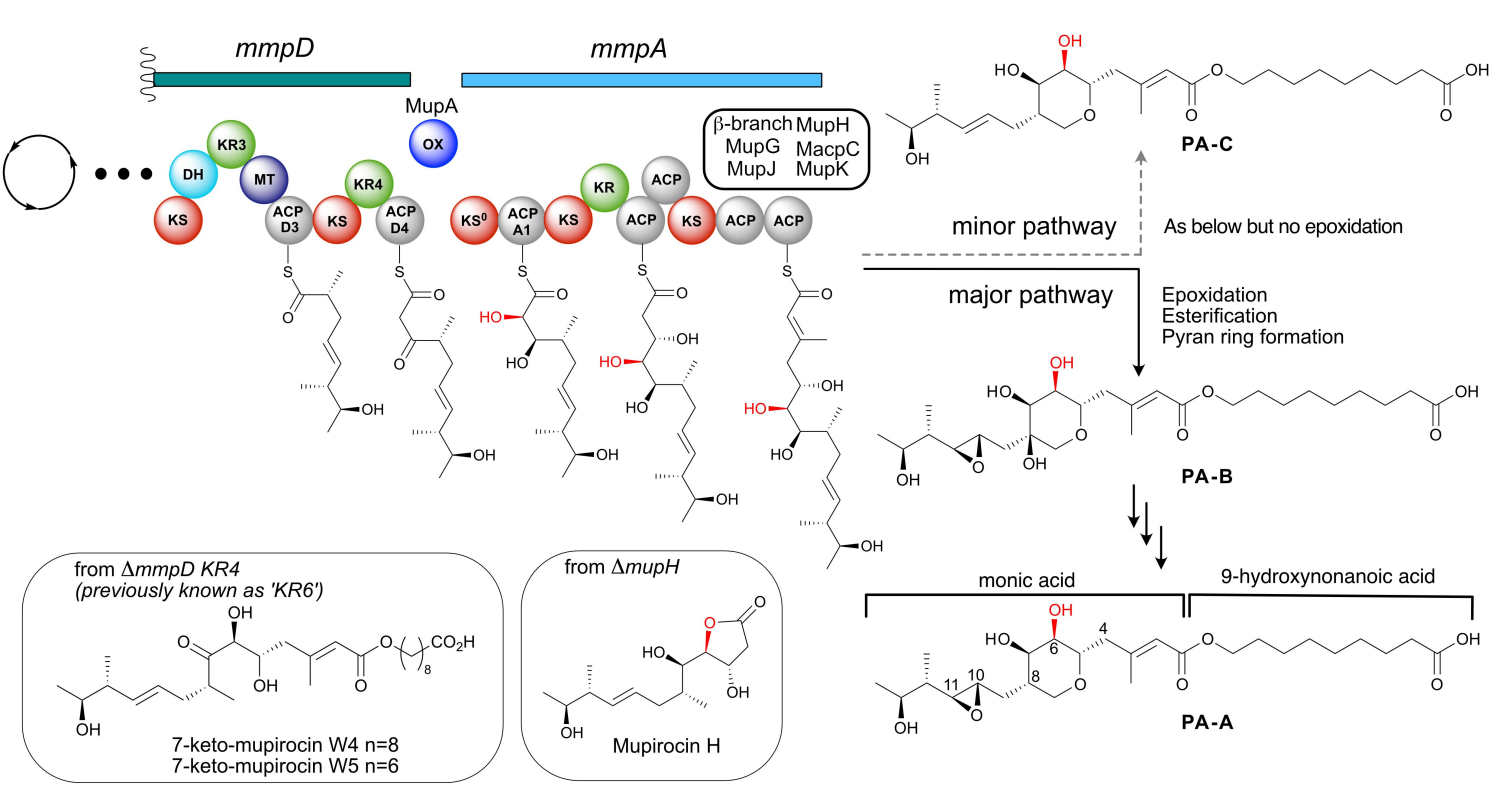
The α‐hydroxylation bimodule of the mupirocin biosynthetic pathway. α‐Hydroxylation at position C6 occurs via a putative α‐hydroxylation module involving an oxygenase at the juncture between MmpD and MmpA. Following polyketide assembly, esterification of the C_17_ monic acid backbone with the fatty acid chain and tetrahydropyran ring formation yields PA−B. PA−B is subsequently converted into PA−A. PA−C is produced in a parallel pathway that is identical other than lacking the epoxidation of 10,11‐alkene.

The antibiotic mupirocin, isolated from *Pseudomonas fluorescens* NCIMB 10585, is among the most effective topical skin treatments for Gram‐positive bacteria including methicillin‐resistant *Staphylococcus aureus* (MRSA).^[2]^ Mupirocin exists as a mixture of pseudomonic acids (PAs) A−C, and the major component is PA−A (Figure 1).^[3]^ The PA structure consists of a C_17_ monic acid polyketide containing a tetrahydropyran (THP) core esterified with a 9‐hydroxynonanoic acid (9‐HN). This compound is biosynthesised by a hybrid *trans*‐acyltransferase (*trans*‐AT) polyketide synthase (PKS) and elucidation of the biosynthetic pathway has proven challenging. The biosynthetic gene cluster encodes six large open reading frames (ORFs), termed the mupirocin multifunctional proteins (*mmpA* to *mmpF*) and 29 ORFs [*mupA*‐*mupZ* and five *trans*‐acting acyl carrier proteins (ACPs), *macpA‐macpE*]. Using a combination of in vitro and in vivo approaches, many of the complex, nonlinear processing steps within the biosynthetic pathway have been resolved. These include introduction of the 15‐methyl group through a β‐branching mechanism,^[4]^ THP formation,^[5a]^ epoxidation of the 10,11‐alkene,^[5b]^ generation of the 3‐hydroxypropionyl fatty acid starter unit,^[6]^ 9‐HN biosynthesis,^[7]^ as well as the multi‐step conversion of PA−B to PA−A involving removal of the 8‐hydroxy group.^[8]^

A key outstanding question in mupirocin biosynthesis concerns the mechanism of 6‐hydroxylation and its precise timing during PA biosynthesis. Isotopic labelling studies have revealed that all the oxygen atoms of PA−A are derived from acetate, except the 10,11‐epoxide and 6‐hydroxy group.^[9]^ Of the three oxygen inserting enzymes (MupA, MupW and MmpE_OR) encoded within the pathway only MupA lacks a proven function.[^5^, ^10^] MupA is predicted to be a flavin‐dependent monooxygenase (FMO).^[10a]^ This group of enzymes are responsible for reactions that incorporate oxygen including halogenation, hydroxylation, epoxidation, Baeyer–Villiger (BV) oxidation and sulfoxidation of organic substrates.^[11]^ They utilise a reduced flavin cofactor (FADH_2_ or FMNH_2_) to generate peroxyflavin or hydroperoxyflavin which subsequently oxygenates a substrate (Scheme S1).^[12]^

Interestingly, knock‐out of the *mmpD*_*KR4* (previously known as KR6) gave 7‐keto‐mupirocins W4 and W5^[5b]^ whilst disruption of any of the genes from the “HCS cassette” responsible for introduction to the β‐methyl branch all led to accumulation of mupirocin H (Figure 1).^[4]^ In addition, all characterised metabolites possessing a THP ring also contain either a 6‐hydroxy or 6‐carbonyl group, indicating that incorporation of the alcohol occurs during the initial stages of polyketide assembly.^[5b]^ Taking these results together led to the proposal that MupA is responsible for 6‐hydroxylation, acting at a point prior to, or just after, the transition of the polyketide chain between the PKS modules MmpD and MmpA. This intermodular juncture and the associated timing of hydroxylation has been detected in a number of biosynthetic pathways and is proposed to comprise an ‘α‐hydroxylation’ bimodule, often housing an associated non‐elongating KS^0^.^[11b]^ Interestingly, a related oxygenase, OocM, from the oocydin polyketide biosynthetic pathway has been shown to α‐hydroxylate a simplified polyketide mimic but required the KS^0^ for activity.^[1b]^ Several key questions therefore remain around the precise enzymatic composition, mechanism and substrate specificity of these oxygenases and the overall architecture of these α‐hydroxylation bimodules remains relatively unexplored.

Herein, we report an interdisciplinary approach which gives refined insights into the mechanism and substrate specificity of MupA and the α‐hydroxylation bimodule of the mupirocin biosynthetic pathway. Our findings have wider relevance in establishing the molecular basis of α‐hydroxylation in other polyketide natural products.

## Results and Discussion

MupA is predicted to be a FMNH_2_‐dependent oxygenase and member of the FMO family. FMOs are grouped into eight subclasses (A−H) based on amino acid sequence, structural fold and activity.[^12b^, ^12d^, ^13^] Sequence homology and ab initio three‐dimensional structural homology modelling of MupA revealed high similarity to the (α/β) eight‐stranded barrel (TIM barrel) of the archetypal subclass C *Vibrio harveyi* luciferase α‐subunit (LuxA; PDB: 1LUC; Figure S1).^[10a][11a]^ This class of monooxygenases are two‐component systems that require an external reductase and work together in conjunction with an NAD(P)H and FMN/FAD redox pair. MupA has high sequence and predicted structural identity with the *trans*‐acting monooxygenases CalD and OocM from the calyculin A^[14]^ and oocydin A^[15]^ PKS pathways, which are themselves proposed to form part of α‐hydroxylation bimodules (Figure S2 and S3).^[1b]^

**Figure 2:**
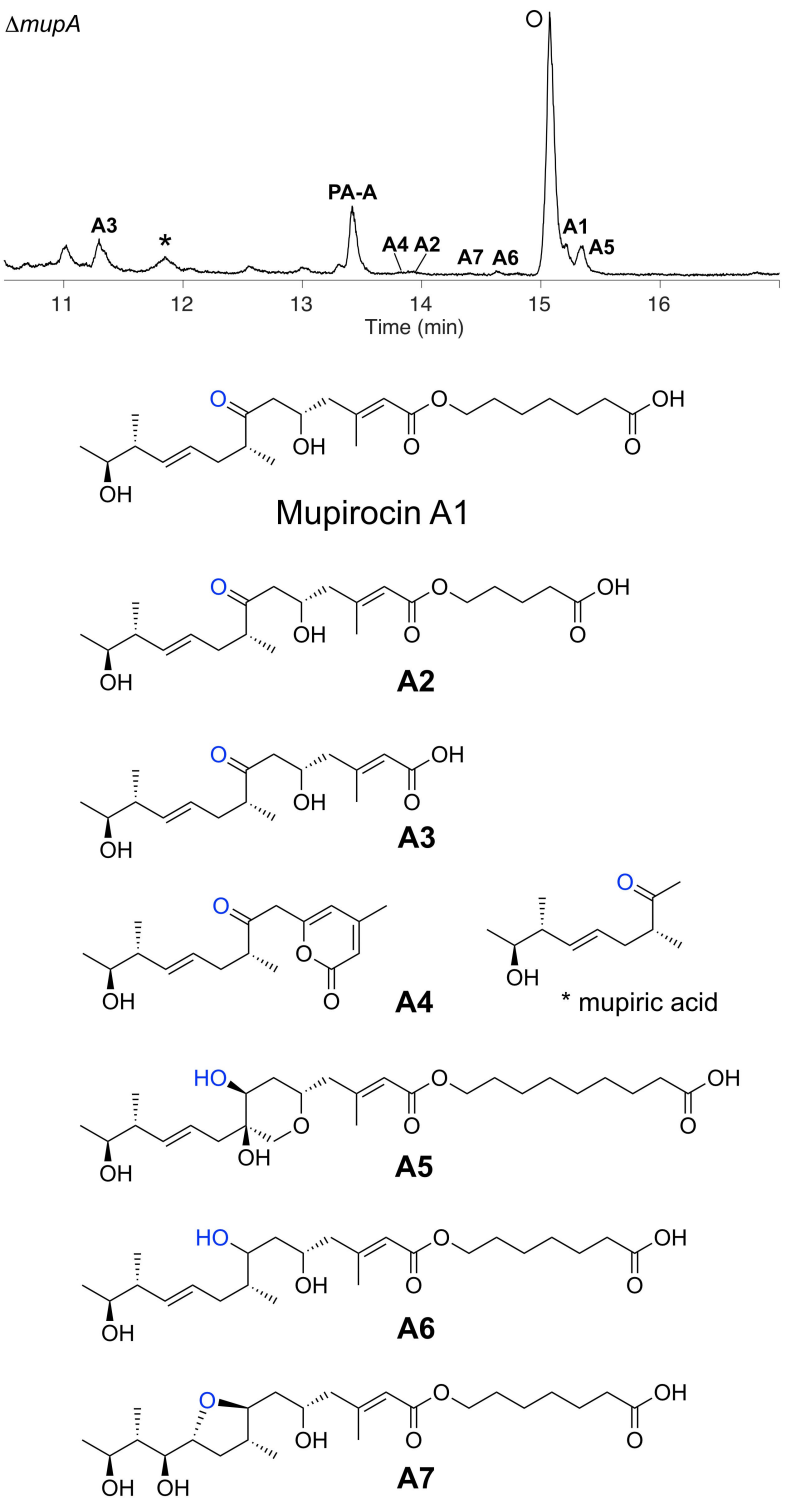
Novel metabolites isolated from extracts of the Δ*mupA* mutant of *Pseudomonas fluorescens* NCIMB 10585. Species corresponding to novel isolated metabolites are labelled A1‐A7. * indicates mupiric acid; O indicates the quorum sensing compound, *N*‐acylhomoserine lactone.^[10a]^

Examination of the Δ*mupA* knockout strain of *P. fluorescens* NCIMB 10586 and analysis of the purified extracts identified seven novel minor mupirocin metabolites, mupirocins A1‐A7 (0.13–2.04 mg L^−1^), as well as the curtailed C_8_ intermediate mupiric acid. The structures of the new metabolites were elucidated using both high‐resolution NMR and electrospray mass spectrometry (ESMS) analysis (Figure 2; See Supporting Information for details). Each of the metabolites lacked the 6‐hydroxy group and contained either a 7‐hydroxy or carbonyl group and varying degrees of polyketide processing, including THP formation, epoxidation (leading to A7 following attack on the 10, 11‐epoxide by the 7‐hydroxy group) and different fatty acid chain lengths. None of the metabolites A1−A7 were detected in extracts from WT *P. fluorescens* NCIMB 10586.

The presence of a 7‐ketone suggested these metabolites escaped reduction by MmpD KR4 (previously known as KR6), the final modular KR of MmpD, and leaves the precise timing of α‐hydroxylation ambiguous. Isolation of PA−A, albeit in very low titres (cf <1 mg L^−1^ vs 50 mg L^−1^ in WT) was persistent in replicates but potential contamination was rigorously excluded. This observation suggested the presence of a homologous enzyme encoded within *P. fluorescens* that could weakly compensate for MupA. BLAST searches indicated several candidate luciferase‐like monooxygenase proteins homologous to MupA that could act as surrogates (Figure S4). Mupirocin H, which is abundant in knock‐out experiments of the “HCS cassette” genes, was not detected, consistent with the requirement for the 6‐hydroxy group in the formation of the γ‐lactone.

### Synthesis and Biotransformation Studies

**Scheme 1:**
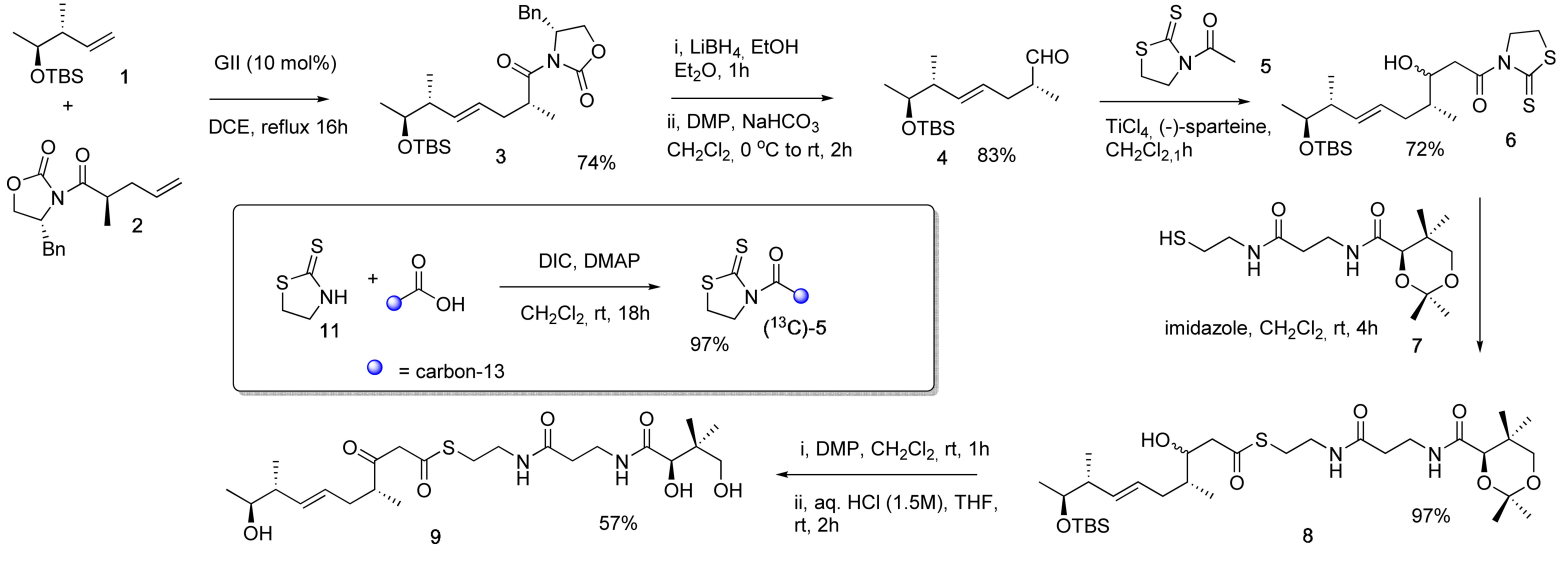
Synthesis of β‐ketothioester **9** and inset, modification to incorporate a specific ^13^C‐label. DMP=Dess–Martin periodinane, GII=Grubbs 2^nd^ generation catalyst, TBS=*tert*‐butyldimethylsilyl, Bn=benzyl, DIC=diisopropylcarbodiimide.

In the following discussion, the β‐keto group corresponds to the C7 position in PA−A and the α carbon relates to C6. Installation of the hydroxy group at the α carbon must occur on precursors bearing either a β‐ketone or hydroxy group, with the oxidation state of the β carbon exclusively mediated by MmpD KR4.^[5b]^ Hydroxylation in FMOs has been proposed to proceed through attack of the substrate on the electrophilic OH donated by hydroperoxyflavin (Scheme S1).^[12d]^ We hypothesised that the presence of the 7‐ketone could enhance the nucleophilicity of the C6 position and this would be the most likely substrate. In this scenario, MmpD KR4 would only reduce the β‐ketone once α‐hydroxylation had taken place. Therefore, β‐keto thioester **9** was synthesised for feeding studies utilising in vivo whole‐cell biotransformations. The synthetic route to **9** was designed to enable selective incorporation of carbon‐13 α to the thioester as shown in Scheme 1. To begin, cross metathesis of alkenes **1** and **2** using Grubbs second generation catalyst gave *E*‐alkene **3** in 74 % yield. Reductive cleavage of the chiral auxiliary followed by oxidation of the resultant primary alcohol using Dess‐Martin periodinane (DMP) provided aldehyde **4**.

Acetyl thiazolidinethione **5** was selected for the aldol‐type reaction with **4** as it is a nonvolatile oil which may be readily prepared by coupling thiazolinethione **11** with (2‐^13^C)‐acetic acid. The Lewis acid‐mediated coupling of acetyl thiazolidinethione **5** with aldehyde **4** gave alcohol **6** as a mixture of diastereomers which were not separated, but used in the reaction with protected pantetheine **7** giving **8** in 70 % yield over the 2 steps. Oxidation of **8** with DMP followed by global deprotection under acidic conditions gave the target β‐hydroxy thioester **9** as a mixture of keto‐enol tautomers.

Thioester **9** was incubated with whole live *Escherichia coli* cells of a strain that overexpressed MupA, supplemented with glucose as previously described.^[5a]^ Extraction and analysis by liquid chromatography‐mass spectrometry (LC–MS) gave no detectable product and traces of **9** and the hydrolysed thioester were recovered (Figure S5).

With potential issues of substrate and product stability, we turned instead to in vitro assays that would offer a greater degree of sensitivity and control. The gene encoding MupA was therefore cloned, over‐expressed and purified to homogeneity (Figure S6). MupA was dimeric by size exclusion chromatography (SEC), and a dimer of dimers at higher concentrations (>2 mg/ml), akin to other FMOs.[^11a^, ^16^] Class C FMOs such as MupA require a reductive partner but analysis of the mupirocin biosynthetic gene cluster failed to identify an unannotated NAD(P)H:flavin oxidoreductase, which is presumably recruited from elsewhere (Figure S4). To circumvent this, we chose the oxidoreductase Fre (GenBank:M61182) as it possesses a useful cofactor promiscuity that removes the uncertainty of selecting specific reductive assay components. Fre is able to utilise FAD or FMN coupled to NADH or NADPH^[17]^ and has been successfully applied as a surrogate oxidoreductase in other subclass C monooxygenase systems.^[18]^ Overexpression of recombinant Fre in *E. coli* yielded protein that purified as the expected dimer (Figure S6) with activity confirmed as previously described (data not shown).^[19]^

### MupA MS and NMR α‐Hydroxylation Assays

Initially the free pantetheine substrate **9** was incubated with MupA, Fre, FMN and NADH and the reaction monitored by LCMS (Figure S7). A single peak was observed that eluted with an identical retention time to **9** (10.1 min) and was confirmed by mass (m/z 511.4 [M+Na]^+^) to be starting material. No product species was identified.

**Figure 3:**
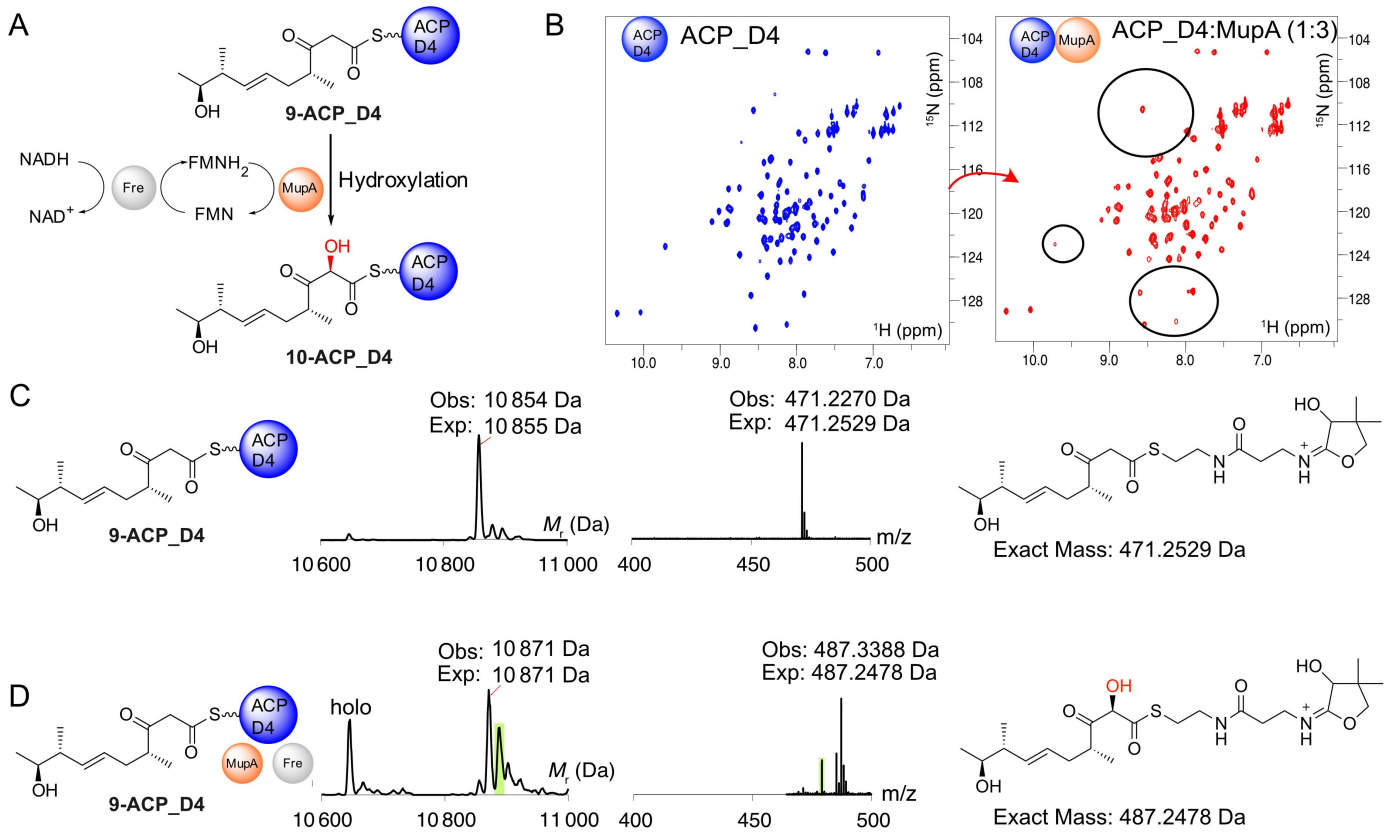
Hydroxylation of 9‐bound ACP_D4. A) Proposed reaction Scheme for the hydroxylation of **9**‐ACP_D4. B) ^1^H−^15^N HSQC spectra of *apo*‐ACP_D4 (blue) and in the presence of 3‐fold excess of MupA (red). C) Deconvoluted ESMS spectra and corresponding Ppant ejection for **9**‐ACP_D4. D) ESMS spectrum for reaction of **9**‐ACP_D4+MupA+Fre+FMN+NADH and the corresponding Ppant ejection from **10**. Additional by‐products corresponding to substrate/product hydrolysis (*holo*‐ACP_D4 10,646 Da) and an unidentified product corresponding to modification to the body of ACP_D4 but not the pantetheine sidearm or substrate (green, Ppant ejection 479.38) were observed.

Next, we considered the requirement for the substrate to be tethered to an ACP (Figure 3A). In the mupirocin biosynthetic pathway hydroxylation may occur around the point that the polyketide chain is transferred from MmpD to MmpA. In this sequence, the final ACP (ACP_D4) of the first module MmpD, transfers the polyketide intermediate to the first KS (KS1) of the second module, MmpA, followed by transfer to the first ACP (ACP_A1). KS1 is predicted to be a non‐elongating KS^0^ and is essential for PA production.^[10a]^

α‐Hydroxylation could conceivably occur on either ACP, depending on any specific gatekeeping role of the KS^0^ or whether the β‐keto, or alternatively a β‐hydroxy substrate, is required by MupA.^[12c]^ We therefore cloned, expressed and purified both ACPs in the *apo* form for in vitro assays (Figure S6). Each excised ACP displayed a well‐dispersed ^1^H NMR spectrum indicative of correct folding.

First, to determine if there was an interaction between MupA and ACP_D4, nonlabelled MupA was added to a solution of ^15^N‐*apo*‐ACP_D4 and monitored by ^1^H−^15^N HSQC NMR spectroscopy. The ^1^H−^15^N HSQC of ^15^N‐*apo*‐ACP_D4 alone is well resolved and titration with MupA revealed line broadening of several peaks, confirming an interaction, albeit weak, with MupA (Figure 3B). Pantetheine derivative **9** was therefore chemoenzymatically upgraded and loaded onto *apo*‐ACP_D4 to yield **9**‐ACP_D4 (observed 10 854 Da; expected: 10 855 Da). The reaction was also monitored using ESMS phosphopantetheine (Ppant) ejection assays that fragment the Ppant arm from the ACP whilst leaving the attached substrate intact, thus permitting accurate detection of low molecular weight intermediates.^[20]^ Ppant ejection yielded the expected ion (observed 471.23 Da; expected 471.25 Da, Figure 3C). The derivatised ACP was then incubated with MupA, Fre and excess FMN and NADH. After 10 minutes, ESMS analysis showed depletion of **9**‐ACP_D4 and the appearance of a new ACP_D4 species (Figure 3D). This species showed the expected addition of 16 Da consistent with hydroxylated ACP‐conjugate **10** (observed 10 871 Da, expected: 10 871 Da). Ppant ejection assay yielded a fragment at 487.34 Da, corresponding to an ejected species fragment with the addition of 16.11 Da (*cf* 471.23 Da). Crucially, this confirmed hydroxylation must have occurred on the conjugated substrate and not elsewhere on the ACP and likewise incubation of *holo‐*ACP_D4 showed no turnover (Figure S8). Substitution of authentic substrate with acetoacetyl‐ACP_D4 failed to give observable turnover (data not shown) and control experiments lacking key assay components all failed to give hydroxylated product (Figure S8). Extended ESMS assay monitoring with active MupA led to a steady loss of product signal suggesting it was unstable in accord with previous synthetic studies of shortened α‐hydroxylated β‐keto thioester substrate mimics.[^1b^, ^21^]

We tested ACP specificity and investigated if ACP_A1 was also a substrate for MupA (Figure S9A). Purified uniformly ^15^N‐labelled *apo*‐ACP_A1 was titrated to a 3‐fold excess of MupA and the mixture monitored by ^1^H−^15^N HSQC spectra (Figure S9B). Surprisingly the ^1^H−^15^N HSQC showed significant broadening of peaks indicative of an interaction between these two proteins. Purified ACP_A1 was therefore loaded with **9** to yield **9**‐ACP_A1 (observed mass: 10 114 Da, expected mass: 10 114 Da) and confirmed by Ppant ejection assay (471.29 Da; Figure S9C). Incubation of **9**‐ACP_A1 with MupA, Fre, FMN and NADH was monitored by ESMS (Figure S9D). Hydroxylation was not, however, observed. Therefore, despite an observable interaction with MupA, this derivatised ACP did not appear to be a substrate for hydroxylation.

**Figure 4:**
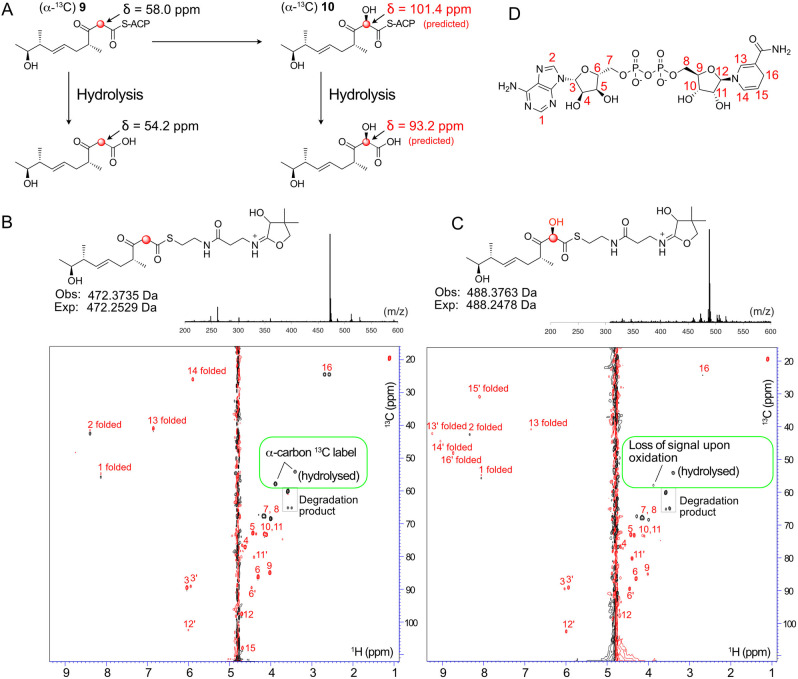
NMR assay to follow α‐hydroxylation. A) Structures of ACP bound intermediates. ^13^C chemical shifts for the hydroxylated species are predicted. B) ^1^H−^13^C HSQC spectrum of control hydroxylation assay mixture (with NADH (5 mM), FMN (1 mM), Fre (20 μM)) but lacking MupA. The resonances from the ^13^C label of (α‐^13^C) **9**‐ACP_D4 and the hydrolysed substrate are boxed in green. Signals from NADH (5 mM) are labelled in red. NAD^+^ signals are labelled in red and with a prime. ‘Folded’ indicates ^13^C shifts outside the upper bound of the ^13^C spectral width that are aliased back into the spectrum at lower ^13^C ppm values. C) As B) but with MupA (20 μM). B & C Top: Parallel ESMS Ppant ejection assays confirmed functional MupA. D) NADH and carbon numbering used.

Product instability suggested that further delineation of the site of hydroxylation would be extremely challenging. To circumvent this problem we opted to incorporate a single ^13^C label into the (^13^C)‐β‐keto thioester with the aim of combining the high proton sensitivity of a micro‐cryoprobe equipped 700 MHz NMR spectrometer and rapid ^1^H−^13^C SO‐FAST HSQC acquisition methods to observe specific shift of the ^13^C labelled C6 signal upon α‐hydroxylation (Figure 4A). The same synthetic approach (Scheme 1) was used for the synthesis of (α‐^13^C) **9** and analysis of the ^13^C NMR spectrum in CDCl_3_ clearly showed enriched singlets at δ55.9 ppm and δ98.3 ppm assigned to the keto and enol forms respectively. When CD_3_OD was used as the solvent the acidic protons α to the ketone and thioester readily exchanged and the ^13^C NMR spectrum displayed enriched multiplets at δ55.9 ppm and δ98.3 ppm due to coupling with deuterium (see Supporting Information).

(α‐^13^C) **9** was used to upgrade ACP_D4 to give (α‐^13^C) **9**‐ACP_D4 which was analysed by ^1^H−^13^C SO‐FAST HSQC spectroscopy in 90 % H_2_O/10 % D_2_O. In the absence of MupA, the ^13^C labelled enriched α‐methylene group yielded a very strong signal at δ ^13^C of 54.8 ppm (Figure 4B). This was observable well above the background from the highest concentration cofactor, NADH, that was present in a 5 mM excess (FMN at 1 mM was barely observable; Figure S10).

The spectrum also revealed the presence of a second resonance at δ ^13^C 50.7 ppm, which was assigned to the ^13^C labelled substrate following hydrolysis from the Ppant arm, which had also been detected in control experiments (Figure S10). These signals (δ ^13^C 54.8 and 50.7 ppm) were present in a ratio of≈2.4 : 1 after 10 minutes. The addition of MupA and rapid reacquisition of spectra for the same total time of 10 minutes led to the immediate depletion of the ^13^C α‐carbon signal at 54.8 ppm whereas the signal intensity of the hydrolysed product remained unchanged (Figure 4C, D). Further, the addition of MupA triggered conversion of NADH to NAD^+^ which could also be followed during the NMR experiment. The turnover was confirmed with parallel ESMS assays that again, demonstrated successful hydroxylation of **9**‐ACP_D4 (Figure 4B, C). The transitory hydroxylated ^13^C signal corresponding to (α‐^13^C) **10**‐ACP_D4 (expected at δ ^13^C≈101 ppm) or hydrolysed product could not be detected as this was very likely masked under the water peak (^1^H chemical shift predicted δ ^1^H≈4.8 ppm) and no alternate signals were detected that could account for the signal loss. Interestingly the NMR experiment confirmed a requirement for an ACP for substrate turnover as only the ACP bound substrate showed depletion of the ^13^C methylene signal, whereas the hydrolysed moiety was unaffected. Therefore, although substrate complexity and product instability made monitoring this reaction challenging, parallel NMR and ESMS assays allowed a sophisticated monitoring of the fate of the ^13^C labelled β‐keto substrate. Together they successfully confirmed the selective turnover of the **9**‐ACP_D4 and rapid loss of the α‐^13^C signal, providing compelling evidence for hydroxylation at this position. Further, the resolution afforded by NMR spectroscopy enabled the precise detection and quantification of hydrolysed substrate, confirming that this did not appear to turnover as suggested by ESMS. This study again demonstrated the power of a combination of NMR and ESMS ejection assays to precisely monitor complex enzymatic transformations using authentic substrates.

**Figure 5:**
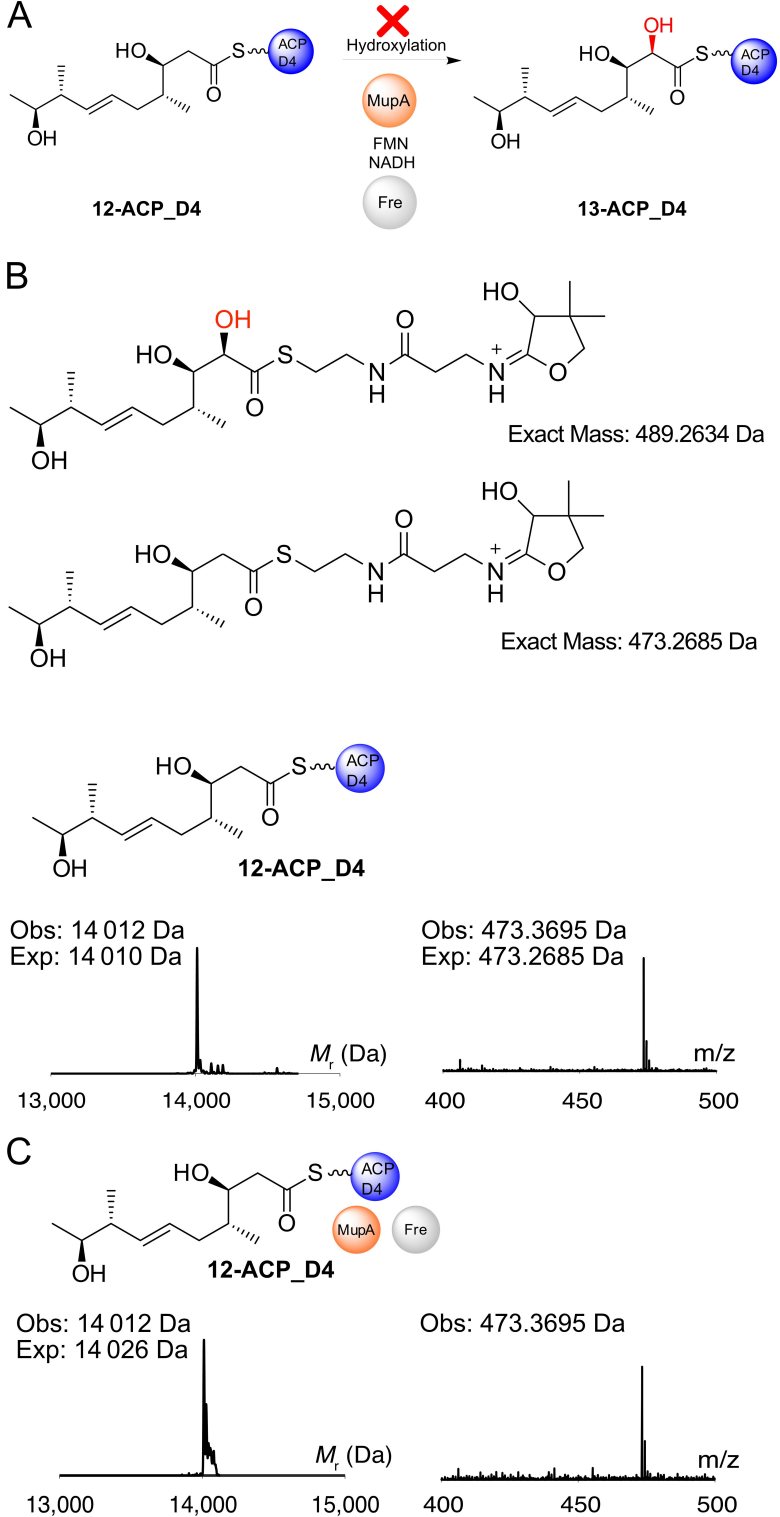
12‐ACP_D4 is not hydroxylated by MupA. A) Possible reaction scheme and Ppant ejection ions of **12**‐ACP_D4 and putative hydroxylated species **13**. ESMS deconvoluted spectra and corresponding Ppant ejection for B) **12**‐ACP_D4 and C) **12**‐ACP_D4+MupA+Fre+FMN+NADH.

The conversion of the β‐keto to a β‐hydroxy group should render the substrate inactive if the β‐keto group is required to increase the nucleophilicity of the α‐carbon (Figure 5A, Scheme S1). Hence the β‐hydroxy substrate **12** was synthesised (Scheme S2) and successfully upgraded onto *apo‐*ACP_D4 as confirmed by ESMS (Figure 5B). Incubation of **12**‐ACP_D4 with MupA, Fre, FMN and NADH was monitored by ESMS and no conversion to the hydroxylated product **13** or consumption of starting material was observed (Figure 5C). Equivalent biotransformation experiments using **12** (data not shown) as well as in vitro ESMS assays with the **12**‐ACP_A1 species (data not shown) both failed to show any conversion.

**Figure 6:**
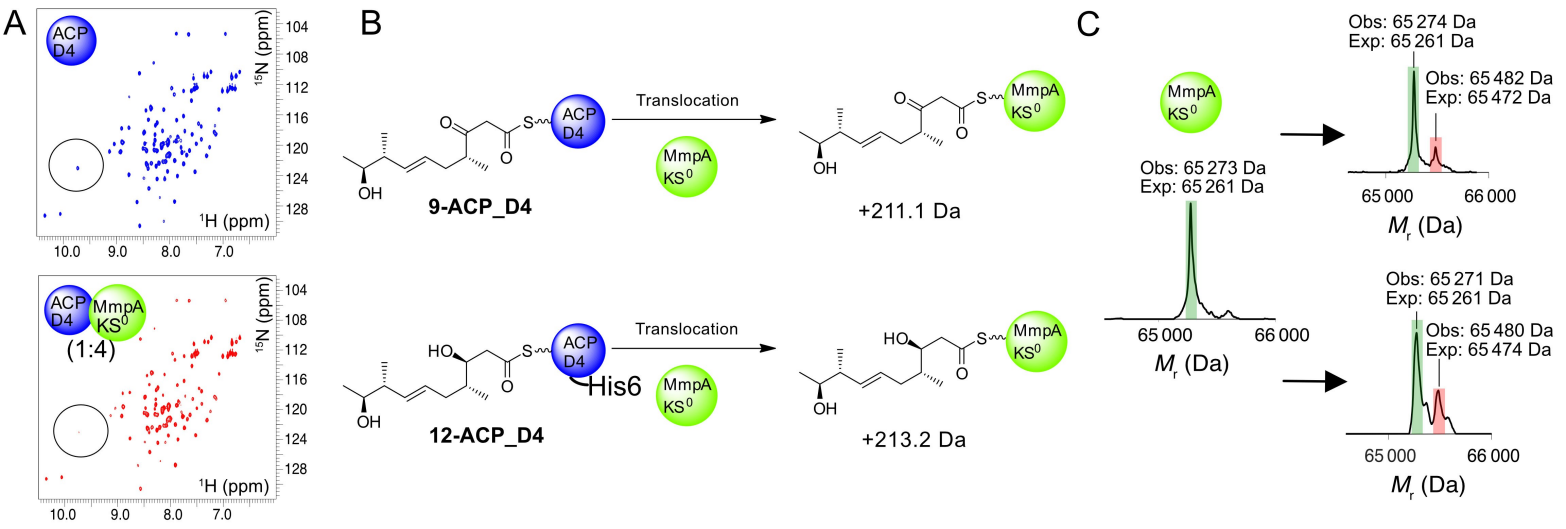
Demonstration of the substrate promiscuity of MmpA_KS^0^. A) ^1^H−^15^N HSQC spectra of *apo*‐ACP_D4 (blue) and in the presence of 4‐fold excess of MmpA_KS0 (red). B) The reaction scheme for the translocation of substrates **9**‐ and **12**‐ACP_D4 onto MmpA_KS^0^ with the expected mass increase indicated. C) Deconvoluted ESMS spectrum for derivatised MmpA_KS^0^ (green) and substrates (red) **9** (top) and **12** (bottom).

The bimodule KS^0^ is essential for PA production^[10a]^ but any role in substrate and ACP selectivity has not been demonstrated. To further explore the role of the MmpA_KS^0^ in vitro, residues 28–570 corresponding to the complete KS^0^ and flanking docking domain were cloned, expressed in recombinant form and purified to homogeneity (Figure S11). Initial application of the ^1^H−^15^N HSQC interaction screen using ^15^N‐labelled ACP_D4 confirmed a weak interaction with the KS^0^ (Figure 6A). Next, we tested whether the KS^0^ would exhibit any gate‐keeping activity as our in vivo results suggested non‐hydroxylated products can be processed, albeit in low yield. Incubation of KS^0^ with **9**‐ACP_D4 and analysis by ESMS revealed the emergence of a second species corresponding to transfer of the β‐keto polyketide (+209 Da versus expected 211.1 Da; Figure 6B). The corresponding assay with **12**‐ACP_D4 also revealed transfer of the β‐hydroxylated intermediate (+209 Da versus expected 213.2 Da) and surprisingly with a modest increase in efficiency. Substrate instability prevented us observing transfer of the α‐hydroxylated substrate when this was tested with ACP_D4 and the KS^0^ which we attributed to the lengthy timescales (up to 60 min) required to observe transfer in vitro. Nonetheless, both assays demonstrated that in isolation, the KS^0^ was not sensitive to the oxidation level of the β‐carbon position.

### Structure and Function of an α‐Hydroxylation Bimodule

**Figure 7:**
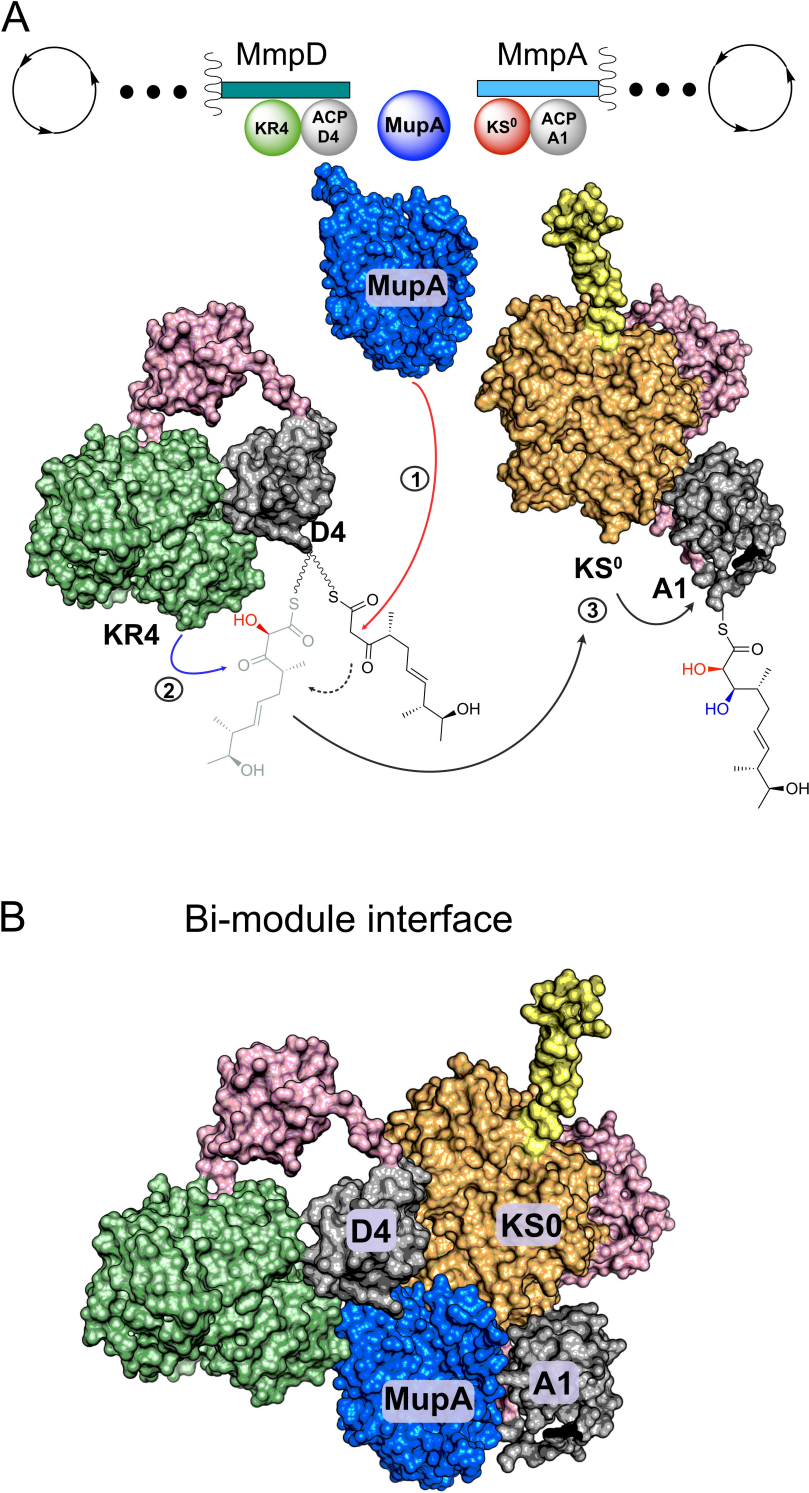
Schematic of the MmpD/MmpA bimodule junction. A) polyketide processing steps. Linker regions are shown in pink and the helical N‐terminal domain of MmpA is shown in yellow. The biosynthetic steps are indicated as 1) α‐hydroxylation 2) reduction of the β‐keto to a β‐hydroxy group 3) transfer to MmpA KS^0^ and then to ACP_A1. B) Depiction of the α‐hydroxylation bimodule interface incorporating inter‐modular contacts that may be formed by MupA.

In this study, we have shown that MupA functions as an α‐hydroxylase, responsible for 6‐hydroxylation in the biosynthesis of the polyketide mupirocin. This hydroxy group is critical for the efficient and downstream processing of biosynthetic intermediates. Incorporation of both hydroxy moieties at C6 and C7 appear to influence recognition and processing of intermediates by MmpE_OR and MupW/T, consistent with previous studies of Δ*mmpD*_*KR4* knockouts.^[5b]^ Hydroxylation of substrates by MupA is, however, strongly influenced by the oxidation state of the β‐carbon position, which in turn is controlled by MmpD KR4. Only ACP_D4 loaded with the β‐keto thioester substrate **9** was successfully hydroxylated by MupA, indicating that the timing of ketoreduction is tightly controlled and occurs strictly post 6‐hydroxylation (Figure 7, Figure S12). Although the precise mechanism for class C FMOs is debated, the requirement for the β‐keto group is in line with a nucleophilic attack on the hydroperoxyflavin. (Scheme S1).^[12c]^

In vitro assays demonstrate that MupA exerts both ACP and β‐ketoacyl substrate specificity. No activity was observed using substrates attached to ACP_A1 implying that hydroxylation occurs before substrate transfer to the first KS of MmpA and subsequent transfer to ACP_A1. There is a strict requirement for the presence of an ACP and hydroxylation of the authentic free β‐ketoacyl pantetheine substrate in solution by MupA was not observed. Other *trans*‐acting type 1 PKS BVMOs have been reported to accept SNAC substrates but are not encoded within modules with an ‘α‐hydroxylation’ bimodule‐like architecture, e.g. OocK and LmbC_OX.^[22]^

A related study by Piel and co‐workers recently reported the α‐hydroxylation activity of OocM, a *trans*‐encoded monooxygenase with homology to MupA from the oocydin A pathway from *Serratia plymuthica* 4Rx13.^[1b]^ These in vitro assays which used short acyl substrate mimics attached to the equivalent of ACP_D4 (OocL_ACP4_) and chemically derivatised products, confirmed α‐hydroxylation and substrate specificity in the context of these simplified precursors. No additional FMN or FMN reductase was required although these may have been present when cell lysate from a *ΔoocM* mutant was added which improved turnover. Interestingly turnover also required the presence of the purified homologous KS^0^ (OocN_KS0_) at the N‐terminal of the next module. The requirement of OocN_KS0_ for OocM activity suggests it may assist in stabilising OocM or stabilise the hydroxylated product prior to trapping by chemical derivatisation in the assays employed. Nonetheless our observations suggest that this may not be a universal requirement and offers the intriguing possibility that α‐hydroxylation modules may offer several different modes of action.

In vitro, the KS^0^ was able to recognise ACP_D4 and accept non‐α‐hydroxylated substrates from ACP_D4, with variable oxidation of the β‐carbon, albeit at a low level. This suggested that in the less stringent context of excised modular domains and in vitro assays, MmpA_KS^0^ did not perform a strict gate‐keeping role. However, the complete absence of metabolites A1‐A7 in extracts from *P. fluorescens* WT suggests that under normal conditions, the correct processing of α‐hydroxylated substrates is tightly controlled. How this is achieved remains unclear as α‐hydroxylated substrates could not be studied directly, but a question that naturally arises is how are all these components coordinated at the interface of these bimodules?

Interestingly, NMR titration experiments revealed a strong interaction of MupA with ACP_A1 downstream of MmpA_KS^0^ and although **9** was not hydroxylated by MupA on this ACP, it was rapidly hydrolysed. Presumably any potential structural interaction with ACP_A1 must be directed in a way as to not hydrolyse the α‐hydroxylated product. One possibility is that the MupA/ACP_A1 interaction may play a role in docking MmpD and MmpA. Known docking motifs for *trans*‐AT modules include the 4‐helix bundle that is frequently observed at intermodular ACP and KS interfaces and the dehydratase docking domains (DHD) that occur as a series of protein‐protein interactions between an unstructured region at the C‐terminal of the KS and the downstream DH of the next module.^[23]^ Amino acid sequence analysis and secondary structure prediction of the ACP C‐ and KS^0^ N‐termini of the mupirocin, calyculin A and oocydin A α‐hydroxylation bimodules fail to reveal the presence of either of these docking motifs (Figures S13 and S14).^[24]^ Structural predictions revealed that the MmpA KS^0^ possesses an N‐terminal helix analogous to those found in the N‐terminal KSs of *cis*‐AT PKS modules and which have been shown to form a homodimeric coiled‐coil with a partner KS.^[25]^ In these *cis*‐AT PKSs this motif forms a 4‐helix bundle with two homodimeric α‐helices at the C‐terminal of the dimerised upstream module and which are distinct to the α‐helices of the C‐terminal ACP. However, the equivalent MmpD C‐terminal lacks any homologous helical motif, instead ending abruptly after ACP_D4. This suggests an alternative set of inter‐modular protein‐protein interactions may engage the N‐terminal KS^0^ helix or this is an evolutionary artefact. Alternatively, a more complex array of interactions involving MupA, ACP_D4, ACP_A1 as well as KR4 and KS^0^ may be involved (eg Figure 7B).^[26]^ The requirement for the presence of the KS^0^ in the oocydin assays may also point to a key role of the KS^0^ in other systems. For example, the patellazole biosynthetic pathway positions the putative α‐hydroxylation domain *in cis* to the KS^0^ which may form important protein‐protein interactions.^[27]^ More generally the bimodule assembly may help stabilise the labile C6 hydroxylated substrate prior to further downstream chain elongation and tailoring.

## Conclusion

In summary, this study precisely defines that the minimum requirement for α‐hydroxylation is MupA, an FMN reductase, a complementary ACP and the β‐keto substrate. We successfully used *E. coli* Fre as a surrogate FMN reductase however in vivo this activity may be supplied by one of three alternate homologs that we identified in the *P. fluorescens* genome. A combination of MS and NMR spectroscopy confirmed ACP_D4 to be essential for substrate recognition and non‐ACP bound substrates were not α‐hydroxylated. Transfer of an α‐hydroxylated species to a purified sample of the downstream MmpA KS^0^ could not be tested in vitro due to substrate instability, however this KS^0^ showed relaxed specificity for substrates with variable oxidation of the β‐carbon. Neither the downstream ACP_A1 or MmpA_KS^0^ were required for functional α‐hydroxylation assays but NMR spectroscopy revealed interactions with both of these components. In light of there being no recognizable docking interface between the two PKS modules, MmpD and MmpA and the requirement of KS^0^ being present in previous functional assays, we suggest an interface between these modules may assemble through interactions involving MupA, ACP_D4, ACP_A1 as well as KR4 and KS^0^. Understanding the precise interplay of *in cis* and *in trans* components, the structure of these assemblies and their influence on substrate channelling and kinetics in *trans*‐AT assemblies is currently the subject of structural investigation and in vitro NMR assay development.

## Conflict of interest

The authors declare no conflict of interest.

1

## Supporting information

As a service to our authors and readers, this journal provides supporting information supplied by the authors. Such materials are peer reviewed and may be re‐organized for online delivery, but are not copy‐edited or typeset. Technical support issues arising from supporting information (other than missing files) should be addressed to the authors.

Supporting Information

## Data Availability

The data that support the findings of this study are available from the corresponding author upon reasonable request.
