# Supplementary material for: Structure and Function of the α‐Hydroxylation Bimodule of the Mupirocin Polyketide Synthase
Source: Angew Chem Weinheim Bergstr Ger. 2023 Oct 16;135(47):e202312514. doi: 10.1002/ange.202312514 (PMC10952193; doi:10.1002/ange.202312514)
Supplement: Supplementary file 1 — Supporting Information [file ANGE-135-0-s001.pdf]

Angewandte Chemie
Eine Zeitschrift der Gesellschaft Deutscher Chemiker

Supporting Information

Structure and Function of the α-Hydroxylation Bimodule of the Mupirocin Polyketide Synthase

A. J. Winter, R. N. Khanizeman, A. M. C. Barker-Mountford, A. J. Devine, L. Wang, Z. Song, J. A. Davies, P. R. Race, C. Williams, T. J. Simpson, C. L. Willis, M. P. Crump**

Table of Contents

1. General experimental procedures 3

2. Fermentation procedure for the Δ*mupA* strain of *Pseudomonas fluorescens* NCIMB 10586 3

3. Characterisation of Mupirocin A1 and Mupirocin A5 4

4. Characterisation of Mupirocin A2 and Mupirocin A4 5

5. Characterisation of Mupirocin A3 5

6. Characterisation of Mupirocin A6 6

7. Characterisation of Mupirocin A7 7

8. Synthesis of 7-keto pantetheine (9) and 7-hydroxyl pantetheine substrates (12) and Scheme S2 8 -

9. Materials and methods for molecular biology techniques 27

10. Plasmid generation, protein expression and purification 27

11 Whole cell biotransformation with MupA 28

12 *In-vitro* LCMS assays 29

13 ESMS 29

14 MupA ESMS assay 29

15 NMR parameters 29

Scheme S1 31

Figures S1-S14 32

General Synthesis NMR spectra 46

Protein Sequences 99

References 101

1. General experimental procedures for chemical synthesis

Commercially available compounds were used without further purification. All air and moisture sensitive reactions were carried out in flame-dried glassware under an N_2 atmosphere using standard Schlenk syringe-septa techniques. Anhydrous solvents: dichloromethane, diethyl ether, tetrahydrofuran, and toluene were obtained by passing through a modified Grubbs system of alumina columns, manufactured by Anhydrous Engineering. Anhydrous DMF and acetone were obtained from commercial suppliers and used without further drying. DIPA and NEt_3 were distilled over CaH_2 prior to use. Petroleum ether is of the 40-60 °C boiling point range. DIPA Analytical thin layer chromatography (TLC) was performed on Merck, aluminium backed 60 F254 silica plates. TLC plates were visualised by UV fluorescence (UV254 lamp) or basic $KMnO_4$ solution. Flash column chromatography was performed with silica gel (technical grade 40-63, Sigma Aldrich) and eluting with the stated solvent system.

Infrared (IR) spectra were recorded on a Perkin Elmer Spectrum One Fourier Transform Infrared Spectrometer (FT-IR). Optical rotations were recorded on a Bellingham and Stanley ADP220 polarimeter with [α] values are quoted as in units 10^{-1} deg cm^2 g^{-1}. Small molecule high resolution mass spectrometry (HRMS) was performed on a Bruker microTOF spectrometer using electrospray ionisation (ESI). ^1H and ^{13}C NMR spectra were recorded using Jeol ECZ 400, Jeol ECS 400, Bruker Nano 400 and Bruker Avance III HD 500 Cryo spectrometers at ambient temperature. Chemical shifts (δ) are quoted in parts per million (ppm) and coupling constants (J) are in Hertz (Hz) rounded to 0.5 Hz intervals. Residual solvent peaks were used as the internal reference for proton and carbon chemical shifts. Two-dimensional NMR techniques (HSQC, COSY, HMBC) were used routinely for structural assignment.

LC-MS data were obtained on a Waters LCMS system comprising Waters 2767 autosampler, Waters 515 HPLC pump, Waters 2998 Diode Array detector, Waters 2424 ELS detector and Waters Quatro Micro mass spectrometer. HPLC grade H_2O and MeCN were added with 0.05% formic acid as solvent system. Analytical LC-MS data were obtained using a Phenomenex Kinetex column (C_{18}, 250 x 4.60 mm, 5 μM) at a flow rate of 1 ml/min, with a gradient of 5-95% MeCN in 20 mins. Preparative HPLC purification were carried out using a Phenomenex Kinetex column (C_{18}, 250 x 21.20 mm, 5 μM) at a flow rate of 16 mL/min. HRESIMS data were obtained on a Bruker Daltonic micrOTOF II instrument. NMR data on isolated compounds from fermentation experiments were collected on Bruker 500 MHz spectrometer equipped with a 5mm ^{13}C optimised cryogenic probe, a Varian DDR 600 MHz spectrometer equipped with a 6.5 mm triple resonance cryogenic probe or Bruker *AVANCE*^{HD} 700 MHz spectrometer equipped with a 1.7 mm triple resonance micro-cryocoil probe.

2. Fermentation procedure for the Δ*mupA* strain of *Pseudomonas fluorescens* NCIMB 10586

The Δ*mupA-pJH2* mutant strain was incubated on LB agar plates (1% Bacto tryptone, 0.5% yeast extract, 0.5% sodium chloride, 2% agar) containing tetracycline (30 μg/ml) and incubated at 30 °C for 2 days. The seed medium was prepared in 100 ml of LB media (1% Bacto tryptone, 0.5% yeast extract, 0.5% sodium chloride, 1% glucose) in a 500 ml flask. The seed medium containing 30 μg/ml tetracycline was inoculated with a single colony from a freshly incubated LB agar plate and incubated at 200 rpm, 25 °C for 20 h.

Fermentation was inoculated with 1% seed culture in modified LB medium (1% Bacto tryptone, 0.5% yeast extract, 0.5%, sodium chloride) supplemented with 4% w/v glucose (24 x 500 ml baffled flasks). The culture was incubated at 200 rpm at 22 °C for 50 h, then centrifuged at 8000 rpm for 15 mins.

The supernatant was extracted with EtOAc three times and the combined extracts were evaporated *in vacuo* to give a crude extract, which was resuspended in MeOH for LC-MS analysis or further purification. After initial purification via preparative LC-MS, samples were further purified by prep-TLC in $CHCl_3$, MeOH, H_2O (80/18/2).

3. Characterisation of Mupirocin A1 and Mupirocin A5

A 4.4 L scale fermentation of Δ*mupA P. fluorescens* NCIMB 10586 was carried out as per general procedure before the crude extract was subjected to LCMS analysis. The isolated extract was subjected to further purification to yield Mupirocin A1 (, 2.04 mgL^{-1}) and Mupirocin A5, 0.22 mgL^{-1}).

Mupirocin A1

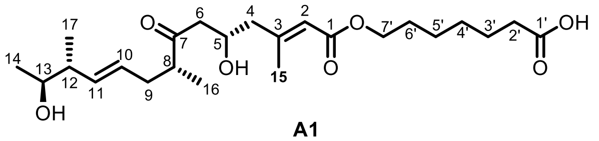

A1

δ_H (500 MHz, CD_3OD) 5.73 (1H, s, 2-H), 5.41-5.39 (2H, m, 11-H and 10-H), 4.30 (1H, tt, *J* 8.0, 6.5, 5-H), 4.08 (2H, t, *J* 6.5, 7′-H_2), 3.59 (1H, m, 13-H), 2.68 (1H, m, 6-H*H*), 2.65 (1H, m, 8-H), 2.60 (1H, m, 6-*H*H), 2.35 (1H, m, 9-H*H*), 2.30 (2H, m, 4- H_2), 2.25 (2H, t, *J* 7.5, 2′- H_2), 2.19 (3H, d, *J* 1.5, 15-H_3), 2.12 (1H, m, 12-H), 2.09 (1H, m, 9-*H*H), 1.66 (2H, m, 6′-H_2), 1.62 (2H, m, 3′-H_2), 1.40 (2H, m, 5′-H_2), 1.39 (2H, m, 4′-H_2), 1.08 (3H, *J* 7.0, 14-H_3), 1.05 (3H, d, *J* 7.0, 16-H_3), 0.97 (3H, d, *J* 7.0, 17-H_3). δ_C (126 MHz, CD_3OD) 214.7 (C-7), 179.0 (C-1′), 168.0 (C-1), 157.9 (C-3), 135.9 (C-11), 128.6 (C-10), 118.8 (C-2), 71.9 (C-13), 66.5 (C-5), 64.7 (C-7′), 49.3 (C-6), 49.3 (C-4), 47.7 (C-8), 45.1 (C-12), 36.8 (C-9), 36.0 (C-2′), 29.9 (C-4′), 29.6 (C-6′), 26.7 (C-5′), 26.4 (C-3′), 20.1 (C-14), 19.0 (C-15), 16.4 (C-17), 15.8 (C-16). HRMS calc. for $[C_{24}H_{40}O_7+Na]^+$ 463.2672, found 463.2622.

Mupirocin A5

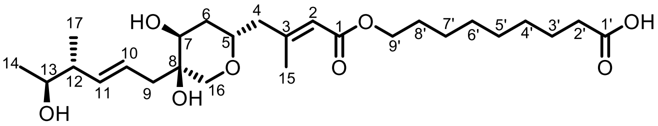

A5

δ_H (500 MHz, CD_3OD) 5.72 (1H, s, 2-H), 5.56 (1H, m, 10-H), 5.48 (1H, m, 11-H), 4.08 (2H, t, *J* 6.5, 9′-H_2), 3.94 (1H, s, 5-H), 3.74 (1H, t, *J* 3.5, 7-H)), 3.62 (1H, m, 13-H), 3.52 (1H, d, *J* 11.0, 16-H*H*), 3.41 (1H, d, *J* 11.0, 16-*H*H), 2.36 (2H, m, 9-H_2), 2.35 (1H, m, 4-H*H*), 2.26 (1H, m, 4-*H*H), 2.23 (2H, t, *J* 7.5, 2′- H_2), 2.17 (4H, m, 12-H, 15-H_3), 1.69 (2H, m, 6-H_2), 1.64 (2H, m, 3′-H_2), 1.64 (2H, m, 8′-H_2), 1.35 (2H, m, 4′-H_2), 1.34 (2H, m, 5′-H_2), 1.29 (2H, m, 6′-H_2), 1.12 (3H, d, *J* 6.5, 14-H_3), 1.01 (3H, d, *J* 7.0, 17-H_3). δ_C (126 MHz, CD_3OD) 179.8 (C-1′), 168.3 (C-1), 158.1 (C-3), 137.3 (C-11), 126.1 (C10), 118.5 (C-2), 72.1 (C-13), 71.2 (C-8), 71.0 (C-5), 70.4 (C-7), 70.0 (C-16), 64.9 (C-9′), 47.2 (C-4), 45.5 (C-12), 39.8 (C-9), 37.0 (C-6), 36.6 (C-2′), 30.5 (C-6′), 30.4 (C-5′), 30.3 (C-4′), 29.8

(C-8′), 27.1 (C-7′), 26.7 (C-3′), 20.2 (C-14), 19.1 (C-15), 16.5 (C-17). HRMS calc. for $[C_{24}H_{44}O_8+Na]^+$ 507.2934, found 507.2920.

4. Characterisation of Mupirocin A2 and Mupirocin A4

A 4.4 L scale fermentation was performed as previously described, before the crude extract was subject to LC-MS analysis. The isolated extract was subjected to further purification to yield Mupirocin A2 (0.13 mgL^{-1}) in a mixture with Mupirocin A4 (0.13 mg/ml).

Mupirocin A2

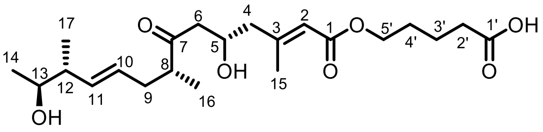

A2

δ_H (500 MHz, CD_3OD) 5.74 (1H, s, 2-H), 5.45 – 5.34 (2H, m, 11-H and 10-H)), 4.29 (1H, d, *J* 7.5, 5-H), 4.09 (2H, t, *J* 6.0, 5′-H), 3.61 (1H, m, 3-H), 2.68 (1H, dd, *J* 17.0, 8.0, 6-H*H*), 2.65 (1H, m, 8-H), 2.58 (1H, dd, *J* 17.0, 5.0, 6-*H*H), 2.35 (1H, m, 9-H*H*), 2.30 (2H, m, $4H_2$), 2.25 (2H, m, 2′-H), 2.19 (3H, s, 15-H), 2.09 (1H, m, 9-*H*H), 1.67 (4H, m, 3′-H, 4′-H), 1.08 (3H, d, *J* 6.5, 14-H_3), 1.05 (3H, d, *J* 7.0, 16-H_3), 0.97 (3H, d, *J* 7.0, 17-H_3).

Mupirocin A4

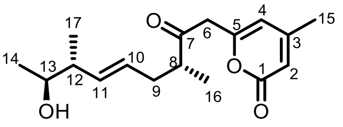

A4

δ_H (500 MHz, CD_3OD) 6.21 (1H, d, *J* 1.5, 4-H), 6.05 (1H, q, *J* 1.5, 2-H), 5.42 (2H, m, 11-H and 10-H), 3.75 (6-*H*D, very weak), 3.60 (1H, dd, *J* 6.5, 5.0, 13-H), 2.78 (1H, p, *J* 7.0, 8-H), 2.38 (1H, dt, *J* 13.5, 6.5, 9-H*H*), 2.19 (3H, s, 15-H), 2.15 (1H, m, 9-*H*H), 2.13 (1H, ddd, *J* 13.0, 9.5, 6.5, 12-H), 1.12 (3H, d, *J* 1.0, 16-H_3), 1.08 (3H, dd, *J* 6.5, 1.5, 14-H_3), 0.98 (3H, d, *J* 7.0, 17-H_3). δ_C (126 MHz, CD_3OD) 211.1 (C-7), 165.7 (C-1), 160.7 (C-3), 159.0 (C-5), 136.5 (C-11), 128.3 (C-10), 111.8 (C-2), 110.4 (C-4), 72.0 (C-13), 47.6 (C-8), 46.5 (C-6), 45.3 (C-12), 36.9 (C-9), 21.4 (C-15), 20.3 (C-14), 16.6 (C-17), 16.0 (C-16). HRMS calc. For $[C_{17}H_{24}O_4+Na]^+$ 315.1572, found 315.1577.

5. Characterisation of Mupirocin A3

A 4.4 L scale fermentation was performed as previously described, before the crude extract was subject to LC-MS analysis. The isolated extract was subjected to further purification to yield Mupirocin A3 (0.15 mgL^{-1}) as a mixture of two isomers of A and B (3:2).

Mupirocin A3 (A)

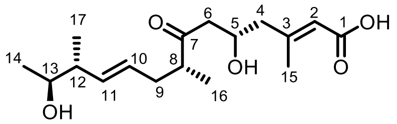

A3(A)

δ_H (500 MHz, CD_3OD) 5.74 (1H, s, 2-H), 5.42-5.39 (2H, m, 11-H and 10-H), 4.29 (1H, m, 5-H), 3.58 (1H, m, 13-H), 2.67 (1H, m, 6-H*H*), 2.62 (1H, dd, *J* 4.5, 2.0, 6-*H*H), 2.57 (1H, m, 8-H), 2.35 (1H, m, 9-H*H*), 2.26 (2H, m, 4H_2), 2.22 (1H, m, 9-*H*H), 2.12 (3H, d, *J* 1.5, 15-H_3), 2.12 (1H, m, 12-H), 1.20 (3H, d, *J* 7.0, 16-H_3), 1.05 (3H, d, *J* 6.5, 14-H_3), 0.93 (3H, d, *J* 7.0, 17-H_3). δ_C (126 MHz, CD_3OD) 215.3 (C-7), 169.1 (C-1), 153.9 (C-3), 136.2 (C-11), 128.7 (C-10), 105.6 (C-2), 72.1 (C-13), 66.7 (C-5), 49.6 (C-6), 49.4 (C-4), 45.2 (C-12), 39.7 (C-8), 38.9 (C-9), 20.0 (C-14), 18.9 (C-15), 18.3 (C-16), 16.3 (C-17).

Mupirocin A3 (B)

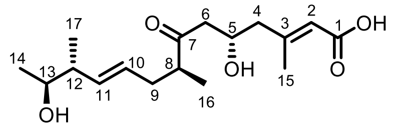

A3(B)

δ_H (500 MHz, CD_3OD) 5.74 (1H, s, 2-H), 5.42-5.39 (2H, m, 11-H and 10-H), 4.29 (1H, m, 5-H), 3.58 (1H, m, 13-H), 2.66 (1H, dd, *J* 7.5, 5.5, 8-H), 2.34 (1H, m, 9-H*H*), 2.26 (2H, m, 4H_2), 2.12 (3H, d, *J* 1.5, 15-H_3), 2.12 (1H, m, 12-H), 2.08 (1H, m, 9-*H*H), 1.08 (3H, d, *J* 6.5, 14-H_3), 1.05 (3H, d, *J* 6.5, 16-H_3), 0.97 (3H, d, *J* 7.0, 17-H_3). δ_C (126 MHz, CD_3OD) 215.3 (C-7), 169.1 (C-1), 153.9 (C-3), 136.0 (C-11), 128.8 (C-10), 105.8 (C-2), 72.1 (C-13), 66.7 (C-5), 49.6 (C-6), 49.4 (C-4), 48.0 (C-8), 45.3 (C-12), 37.0 (C-9), 20.3 (C-14), 18.9 (C-15), 16.6 (C-17), 15.9 (C-16).

6. Characterisation of Mupirocin A6

A 4.4 L scale fermentation was performed as previously described, before the crude extract was subject to LC-MS analysis. The isolated extract was subjected to further purification to yield Mupirocin A6 (0.57 mgL^{-1}).

Mupirocin A6

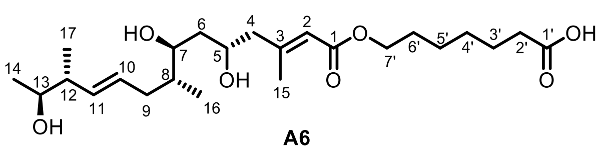

A6

δ_H (500 MHz, CD_3OD) 5.73 (1H, s, 2-H), 5.41 (2H, m, 11-H and 10-H), 4.07 (2H, t, *J* 6.5 Hz, 7′-H_2), 4.02 (1H, m, 5-H), 3.62 (1H, m, 7-H), 3.61 (1h, m, 13-H), 2.38 (1H, m, 4-H*H*), 2.23 (1H, m, 4-*H*H), 2.19 (3H, d, *J* 1.5, 3H, 15-H_3), 2.17 (1H, m, 9-H*H*), 2.15 (4H, m, 2′-H_2, 12-H), 1.89 (1H, m, 9-*H*H), 1.65 (2H, m, 6′-H_2), 1.62 (1H, m, 6-H*H*), 1.60 (2H, m, 3′-H_2), 1.59 (1H, m, 8-H), 1.54 (1H, m, 6-*H*H),

1.39 (2H, m, 5′-H_2), 1.33 (2H, m, 4′-H_2), 1.09 (3H, d, *J* 7.0, 14-H_3), 0.99 (3H, d, *J* 7.0, 17-H_3), 0.89 (3H, d, *J* 7.0, 16-H_3). δ_C (126 MHz, CD_3OD) 182.8 (C-1′), 168.2 (C-1), 158.4 (C-3), 134.8 (C-11), 130.2 (C-10), 118.5 (C-2), 74.8 (C-7), 72.1 (C-13), 69.8 (C-5), 64.9 (C-7′), 49.6 (C-4), 45.3 (C-12), 40.7 (C-6), 40.5 (C8), 39.2 (C-2′), 36.5 (C-9), 30.4 (C4′), 29.8 (C-6′), 27.6 (C-3′), 26.9 (C-5′), 20.2 (C-14), 19.2 (C-15), 16.6 (C-17), 15.4 (C-16). HRMS calc. For $[C_{24}H_{42}O_7+Na]^+$ 465.2828, found 465.2817.

7. Characterisation of Mupirocin A7

A 4.4 L scale fermentation was performed as previously described, before the crude extract was subject to LC-MS analysis. The isolated extract was subjected to further purification to yield Mupirocin A7 (0.38 mgL^{-1}).

Mupirocin A7

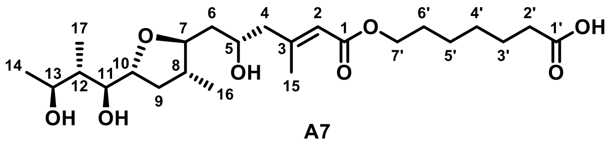

A7

δ_H (500 MHz, CD_3OD) 5.75 (1H, s, 2-H), 4.10 (1H, m, 10-H), 4.07 (2H, t, *J* 6.5, 7′-H_2), 4.02 (1H, m, 5-H), 4.00 (1H, m, 13-H), 3.65 (2H, dd, *J* 7.0, 3.0, 11-H), 3.58 (1H, m, 7-H), 2.40 (1H, m, 4-H*H*), 2.34 (1H, dt, *J* 13.0, 7.5, 9-H*H*), 2.27 (1H, dd, *J* 14.0, 8.5, 4-*H*H), 2.19 (3H, d, *J* 1.5, 15-H_3), 2.15 (2H, ddd, *J* 10.0, 6.5, 4.5, 2′-H), 2.01 (1H, m, 9-*H*H), 1.90 (1H, m, 8-H), 1.75 (1H, ddd, *J* 14.0, 5.0, 3.0, 6-H*H*), 1.60 (1H, m, 6-*H*H), 1.58 (1H, m, 12-H), 1.64 (2H, m, 6′-H_2), 1.61 (2H, m, 5′-H_2), 1.38 (2H, m, 3′H_2), 1.29 (2H, m, 4′H_2), 1.12 (3H, d, *J* 6.5, 14-H_3), 1.05 (3H, d, *J* 6.5, 16-H_3), 0.84 (3H, d, *J* 7.0, 17-H_3). δ_C (126 MHz, CD_3OD) 182.3 (C-1′), 167.8 (C-1), 158.0 (C-3), 118.2 (C-2), 84.8 (C-7), 80.5 (C-10), 75.9 (C11), 69.6 (C-13), 68.8 (C-5), 64.6 (C-7′), 49.3 (C-4), 43.7 (C-12), 42.4 (C-6), 41.6 (C-8), 38.9 (C-2′), 35.0 (C-9), 30.4 (C-4′), 29.6 (C-6′), 27.6 (C-5′), 26.7 (C-3′), 18.8 (C-15), 18.8 (C14), 10.9 (C-17). HRMS calc. For $[C_{24}H_{41}O_7+Na]^+$ 481.2777, found 481.2788.

8. Synthesis of 7-keto pantetheine (9) and 7-hydroxyl pantetheine substrates (12)

Scheme S2. General synthetic scheme of 7-keto pantetheine (9) and 7-hydroxyl pantetheine (12) substrates.

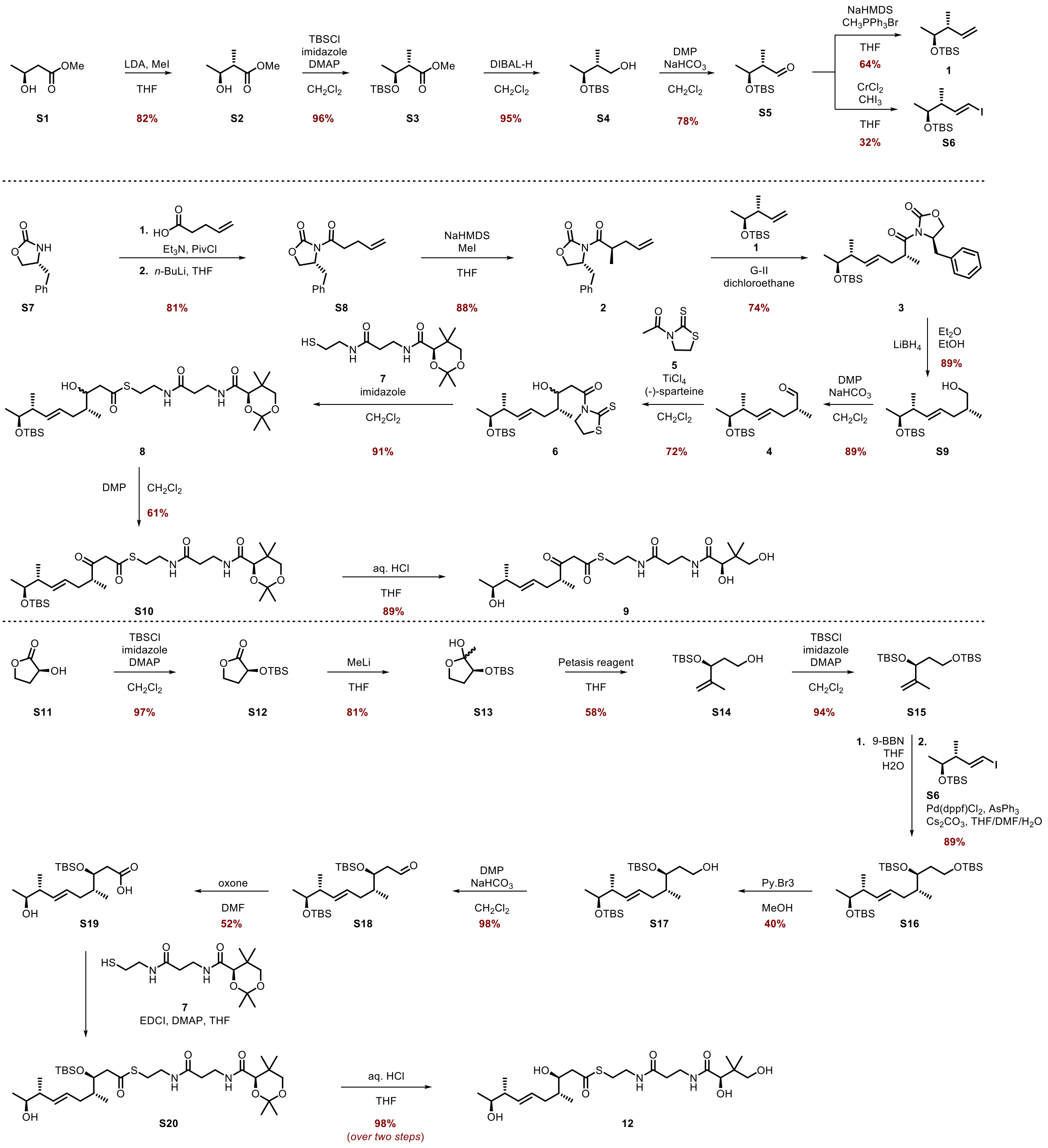

Methyl (2*S*,3*S*)-3-hydroxy-2-methylbutanoate S2

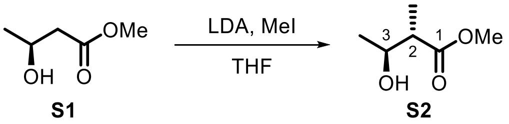

DIPA (3.77 ml, 26.7 mmol) was dissolved in anhydrous THF (20 mL) and the solution cooled to –78 °C. *n*-BuLi (16.7 mL, 26.7 mmol, 1.61 M in hexane) was added dropwise and the reaction mixture stirred at –78 °C for 1 h. A solution of ester **S1** (1.40 mL, 12.8 mmol) in anhydrous THF (10 mL) was then added dropwise at –78 °C before the cooling bath was removed and the reaction mixture was stirred at room temperature for 20 mins. The reaction mixture and was then re-cooled to –78 °C. MeI (0.96 mL, 15.4 mmol) was added dropwise and the reaction mixture was allowed to warm to 0 °C and stirred for 3 h. The reaction was quenched by the addition of HCl (6 M, 10 mL) and the aqueous layer was extracted with Et_2O (3 × 30 mL). The combined organic extracts were dried over $MgSO_4$, filtered and concentrated *in vacuo*. The crude residue was purified by column chromatography (5% EtOAc in petroleum ether) to give ester **S2** as a colourless oil (1.20 g, 82%). δ_H (400 MHz, $CDCl_3$) 3.87 (1H, p, *J* 7.0, 3-H), 3.69 (3H, s, OCH_3), 2.44 (1H, p, *J* 7.0, 2-H), 1.20 (3H, d, *J* 7.0, 4-H_3), 1.15 (3H, d, *J* 7.0, 2-CH_3). δ_C (101 MHz, $CDCl_3$) 176.3 (C-1), 63.4 (C-3), 51.7 (OCH_3), 46.9 (C-2), 20.7 (2-CH_3), 14.0 (C-4). Data in accordance with the literature [1].

Methyl (2*S*,3*S*)-3-(*tert*-butyldimethylsilyloxy)-2-methylbutanoate S3

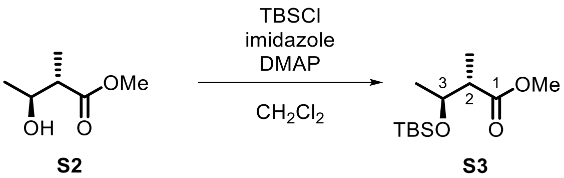

Alcohol **S2** (600 mg, 4.54 mmol), imidazole (463 mg, 6.91 mmol) and DMAP (55 mg, 0.45 mmol) were dissolved in CH_2Cl_2 (15 mL). TBSCl (1.23 g, 8.17 mmol) was added and the reaction stirred for 16 h. The reaction was quenched with H_2O (*ca.* 10 mL) and the aqueous layer extracted with CH_2Cl_2 (3 × 15 mL). The combined organic extracts were dried over $MgSO_4$, filtered and concentrated *in vacuo*. The crude residue was purified by column chromatography (2% EtOAc in petroleum ether) to give ester **S3** as a colourless oil (1.08 g, 96%). $[\alpha]_D^{24}$ +35.4 (*c* 1.3, $CHCl_3$), $[\alpha]_D^{24}$ +42.0 (c 1.25, $CHCl_3$) [2]. δ_H (400 MHz, $CDCl_3$) 3.98 (1H, dq, *J* 7.0, 6.0, 3-H), 3.63 (3H, s, OMe), 2.47 (1H, pent, *J* 7.0, 2-H), 1.10 (3H, d, *J* 6.0, 4-H_3), 1.06 (3H, d, *J* 7.0, 2-CH_3), 0.89 (9H, s, $C(CH_3)_3$), 0.03 (3H, s, $Si(CH_3)_2$), 0.00 (3H, s, $Si(CH_3)_2$). δ_C (101 MHz, $CDCl_3$) 175.7 (C-1), 70.2 (C-3), 51.4 (OCH_3), 48.1 (C-2), 25.7 ($SiC(\underline{C}H_3)_3$),

20.6 (C-4), 17.9 (SiC(CH3)3), 12.7 (2-CH3), –4.3 (Si(CH3)2), –5.1 (Si(CH3)2). Data in accordance with the literature [3].

(2*R*,3*S*)-3-(*tert*-Butyldimethylsilyloxy)-2-methylbutan-1-ol S4

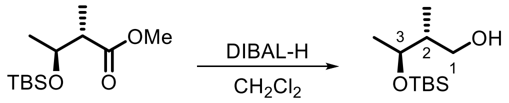

Ester **S3** (500 mg, 2.15 mmol) was dissolved in CH_2Cl_2 (6 mL) and the solution cooled to –78 °C. DIBAL-H (1 M in hexane, 4.52 mL, 4.52 mmol) was added dropwise and the reaction mixture was allowed to warm to room temperature and stirred for 2 h. Sat. aq. potassium sodium tartrate (10 mL) was added and the reaction mixture was stirred vigorously overnight. The layers were separated and the aqueous layer was extracted with CH_2Cl_2 (3 × 10 mL). The combined organic extracts were dried over $MgSO_4$, filtered, and concentrated *in vacuo* to give **S4** (447 mg, 95%) as a colourless oil. $[\alpha]_D^{24}$ = +12.0 (*c* 1.0, $CHCl_3$), $[\alpha]_D^{24}$ = +23.0 (*c* 1.0, $CHCl_3$) [4]. δ_H (400 MHz, $CDCl_3$) 3.72 (1H, m, 1-*H*H), 3.66 (1H, m, 3-H), 2.73 (1H, m, 1-H*H*), 1.52 (1H, m, OH), 1.12 (3H, d, *J* 7.0, 4-H_3), 0.87 (3H, d, *J* 7.0, 2-CH_3), 0.80 (9H, s, SiC(CH_3)$_3$), 0.00 (3H, s, SiCH_3), 0.00 (3H, s, SiCH_3). δ_C (101 MHz, $CDCl_3$) 74.1 (C-3), 65.9 (C-1), 41.7 (C-2), 25.8 (SiC(CH3)3), 22.2 (C-4), 17.9 (SiC(CH3)3), 14.7 (2-CH3), –4.2 (Si(CH3)2), –5.0 (Si(CH3)2). Data in accordance with the literature [5].

(2*S*,3*S*)-3-(*tert*-Butyldimethylsilyloxy)-2-methylbutanal S5

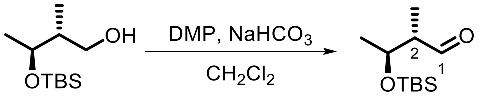

Alcohol **S4** was dissolved in CH_2Cl_2 (32 mL) and the solution cooled to 0 ˚C. $NaHCO_3$ (2.03 g, 24.2 mmol) and DMP (4.79 g, 11.28 mmol) were added and the reaction mixture was allowed to warm to room temperature and stirred for 2 h. The reaction was quenched by the addition of 10 % aq. $Na_2S_2O_3$ (30 mL) and sat. aq. $NaHCO_3$ (30 mL). The aqueous layer was extracted with CH_2Cl_2 (3 × 15 mL) and the combined organic extracts were dried over Na_2SO_4, filtered, and concentrated *in vacuo*. The crude residue was purified by column chromatography (0 – 15% Et_2O in pentane) to give **S5** (1.37 g, 78%) as a colourless oil. $[\alpha]_D^{24}$ = +43.0 (*c* 1.0, $CHCl_3$), $[\alpha]_D^{24}$ = +47.0 (*c* 1.0, $CHCl_3$) [2]. δ_H (400 MHz, $CDCl_3$) 9.69 (1H, d, *J* 2.5, 1-H), 3.96 (1H, m, 3-H), 2.30 (1H, app. pd, *J* 6.0, 2.5, 2-H), 1.15 (3H, d, *J* 6.0, 4-H_3), 1.00 (3H, d, *J* 6.0, 2-CH_3), 0.80 (9H, s, SiC(CH_3)$_3$), 0.00 (3H, s, SiCH_3), –0.01 (3H, s, SiCH_3). δ_C (101 MHz, $CDCl_3$) 205.2 (C-1), 69.9 (C-3), 53.7 (C-2), 25.7 (SiC(CH3)3), 21.8 (C-4), 18.0 (SiC(CH3)3), 10.6 (2-CH3), –4.2 (SiCH3), –5.0 (SiCH3). Data in accordance with the literature [2].

(3*R*,4*S*)-4-(*tert*-Butyldimethylsilyloxy)-3-methylpent-1-ene 1

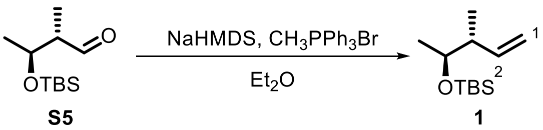

CH_3PPh_3Br (1.16 g, 3.25 mmol) was suspended anhydrous Et_2O (10 mL) and the suspension cooled to –78 °C. NaHMDS (1 M in Et_2O, 3.33 ml, 3.33 mmol) was added and the reaction mixture was stirred for 1 h at –78 °C. Aldehyde **S5** (600 mg, 2.78 mmol) was added dropwise and the reaction mixture was allowed to warm to room temperature and stirred for 16 h. The reaction was quenched by the addition of sat. aq. NH_4Cl (10 mL) and the aqueous layer was extracted with Et_2O (3 × 10 mL). The combined organic extracts were dried over $MgSO_4$, filtered, and concentrated *in vacuo*. The crude residue was purified by column chromatography (1% EtOAc in petroleum ether) to give **1** as a colourless oil (446 mg, 64%). $[\alpha]_D^{23.6}$ = +7.5 (*c* 2.0, $CHCl_3$), $[\alpha]_D^{26}$ = +8.2 (*c* 2.3, $CHCl_3$)[6]. δ_H (400 MHz, $CDCl_3$) 5.75 (1H, ddd, *J* 17.0, 10.5, 7.5, 2-H), 4.96 (1H, m, 1-*H*H), 4.92 (1H, m, 1-*H*H), 3.67 (1H, qd, *J* 6.0, 4.5, 4-H), 2.13 (1H, m, 3-H), 1.01 (3H, d, *J* 6.5, 5-H_3), 0.95 (3H, d, *J* 6.5, 3-CH_3), 0.85 (9H, s, $SiC(CH_3)_3$), 0.00 (3H, s, $SiCH_3$), 0.00 (3H, s, $SiCH_3$). δ_C (101 MHz, $CDCl_3$) 141.2 (C-2), 114.2 (C-1), 71.7 (C-4), 45.4 (C-3), 25.9 ($SiC(\underline{C}H_3)_3$), 20.6 (C-5), 18.1 ($Si\underline{C}(CH_3)_3$), 15.5 (3-CH_3), –4.3 ($SiCH_3$), –4.8 ($SiCH_3$). Data in accordance with the literature [6].

***N*-Acetylthiazolidine-2-thione 5**

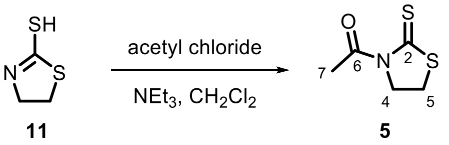

4,5-dihydrothiazole-2-thiol **11** (2.00 g, 16.8 mmol) was dissolved in CH_2Cl_2 (27 mL) and the solution cooled to 0 ˚C. Acetyl chloride (1.19 mL, 16.8 mmol) and Et_3N (2.34 mL, 16.8 mmol) were added and the reaction mixture was then allowed to warm to room temperature and stirred for 3 h. The reaction was quenched with sat. aq. NH_4Cl (27 mL). The layers were separated and the aqueous layer was extracted with CH_2Cl_2 (3 × 15 mL). The combined organic extracts were dried over Na_2SO_4, filtered and concentrated *in vacuo*. The crude residue was purified by column chromatography (0 – 30% EtOAc in petroleum ether) to give **5** (2.70 g, quant.) as a bright yellow oil. δ_H (500 MHz, $CDCl_3$) 4.56 (2H, t, *J* 7.5, 4-H_2), 3.28 (2H, t, *J* 7.5, 5-H_2), 2.76 (1H, s, 7-H_3). δ_C (126 MHz, $CDCl_3$) 202.1 (C-2), 171.5 (C-6), 55.7 (C-4), 28.3 (C-5), 27.1 (C-7). Data in accordance with the literature [7].

(7-^{13}C)-*N*-Acetylthiazolidine-2-thione 5

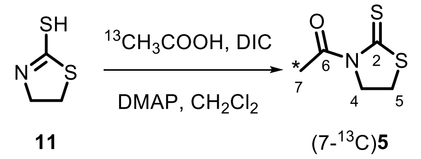

4,5-dihydrothiazole-2-thiol **11** (1.50 g, 12.60 mmol), DMAP (0.257 g, 2.10 mmol) and DIC (2.44 mL, 15.75 mmol) were dissolved in CH_2Cl_2 (90 mL). (2-^{13}C)acetic acid (0.60 mL, 10.50 mmol) was added and the reaction mixture was stirred at room temperature for 18 h. The volatiles were removed *in vacuo* and the crude residue was purified by column chromatography (20 % EtOAc in petroleum ether) to give (7-^{13}C)**5** (1.64 g, 97%) as a bright yellow oil. δ_H (500 MHz, $CDCl_3$): 4.58 (2H, t, *J* 7.5, 4-H_2), 3.29 (2H, t, *J* 7.5, 5-H_2),2.78 (3H, d, *J* 131.0, 7-H_3). δ_C (126 MHz, $CDCl_3$): 202.1 (C-2), 171.6 (d, *J* 52.5, C-6), 55.7 (C-4), 28.3 (C-5), 27.1 (C-7 (enhanced)).

(*R*)-4-Benzyl-3-(pent-4′-enoyl)oxazolidin-2-one S8

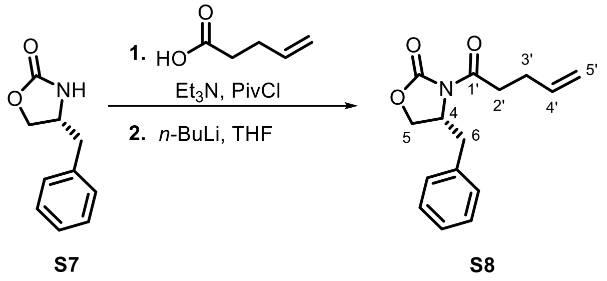

4-Pentenoic acid (1.00 mL, 10.2 mmol) was dissolved in anhydrous THF (5 mL) and the solution cooled to –78 °C. Triethylamine (1.81 mL, 13.2 mmol) was added and then pivaloyl chloride (1.25 mL, 10.2 mmol) was added dropwise. The reaction mixture was stirred at –78 °C for 30 mins, then warmed to room temperature for 2 h before being re-cooled to –78 °C. In a separate flask, Evans' auxiliary **S7** (1.64 g, 10.2 mmol) was dissolved in anhydrous THF (10 mL) and the solution cooled to –78 °C. *n*-BuLi (1.6 M in hexanes, 6.45 mL, 10.2 mmol) was added slowly to the solution of **S7** at and the mixture was then allowed to warm to room temperature and stirred for 30 mins. The anion was slowly transferred to the mixed anhydride solution at –78 °C and the reaction mixture was then allowed to warm to room temperature and stirred for 16 h. The reaction was quenched by the addition of sat. aq. $NaHCO_3$ (20 mL) and the layers were separated. The aqueous layer was extracted with EtOAc (3 × 20 mL) and the combined organic extracts dried over $MgSO_4$, filtered and concentrated *in vacuo*. The crude residue was purified by column chromatography (10-20% EtOAc in petroleum ether) to give **S8** as a colourless oil (2.10 g, 81%). δ_H (400 MHz, $CDCl_3$) 7.25 (3H, m, Ar-H), 7.15 (2H, m, Ar-H), 5.81 (1H, m, 4′-H), 5.00 (2H, m, 5′-H_2), 4.62 (1H, m, 5-H*H*), 4.12 (1H, m, 5-*H*H), 4.05 (1H, m, 4-H), 3.25 (1H, dd, *J* 13.0, 3.0, 3′-*H*H), 2.97 (2H, m, 6-H_2), 2.71 (1H, m, 3′-H*H*), 2.40 (2H, m, 2′-H_2). δ_C (101 MHz, $CDCl_3$) 172.5 (C-2), 153.4 (C-1′), 136.7 (C-4′), 135.2 (Ar-C), 129.4 (2 × Ar-C), 129.0 (2 × Ar-C), 127.3 (Ar-C), 115.7 (C-5′), 66.2 (C-5), 55.2 (C-4), 38.1 (C-3′), 37.9 (C-6), 28.2 (C-2′). Data in accordance with the literature [8].

(*R*)-4-Benzyl-3-((*R*)-2′-methylpent-4′-enoyl)oxazolidin-2-one 2

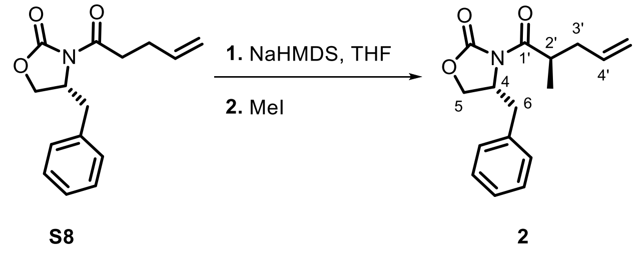

Alkene **S8** (0.20 g, 1.18 mmol) was dissolved in anhydrous THF (4 mL) and cooled to –78 °C. NaHMDS (1 M in hexanes, 1.77 mL, 1.77 mmol) was added dropwise and the reaction mixture was

stirred for 1 h at –78 °C. MeI (0.21 mL, 4.72 mmol) was then added dropwise at –78 °C. After stirring for a further 1 h at –78 °C, the reaction was quenched by the addition of sat. aq. NH_4Cl (10 mL). The aqueous layer was extracted with EtOAc (3 × 15 mL) and the combined organic extracts were dried over $MgSO_4$, filtered and concentrated *in vacuo*. The crude residue was purified by column chromatography (20% EtOAc in petroleum ether) to give **2** as a colourless oil (0.23 g, 88%). $[\alpha]_D^{24}$ –2.0 (*c* 1.0, $CHCl_3$). δ_H (400 MHz, $CDCl_3$) 7.22 (3H, m, Ar-H), 7.11 (2H, m, Ar-H), 5.66 (1H, m, 4′-H), 4.93 (2H, m, 5′-H_2), 4.53 (1H, m, 5-*H*H), 4.08 (1H, m, 5-H*H*), 4.06 (1H, m, 4-H), 3.71 (1H, m, 2′-H), 3.15 (1H, dd, *J* 13.0, 3.0, 3′-*H*H), 2.66 (1H, m, 3′-H*H*), 2.37 (1H, m, 6-H*H*), 2.08 (1H, m, 6-*H*H), 1.13 (3H, d, *J* 7.0, 2′-CH_3). δ_C (101 MHz, $CDCl_3$) 176.6 (C-2), 153.1 (C-1′) 135.7 (C-4′), 135.4 (Ar-C), 129.6 (2 × Ar-C), 129.1 (2 × Ar-C), 127.5 (Ar-C) 117.2 (C-5′), 66.2 (C-4), 55.5 (C-5), 38.1 (C-2′), 37.7 (C-3′), 37.6 (C-6), 17.2 (2′-CH_3). Data in accordance with the literature [8] (No optical rotation recorded in literature).

(*R*)-4-Benzyl-3-((2*R*,6*R*,7*S*,*E*)-7′-((*tert*-butyldimethylsilyl)oxy)-2′,6′-dimethyloct-4′-enoyl)oxazolidin-2-one 3

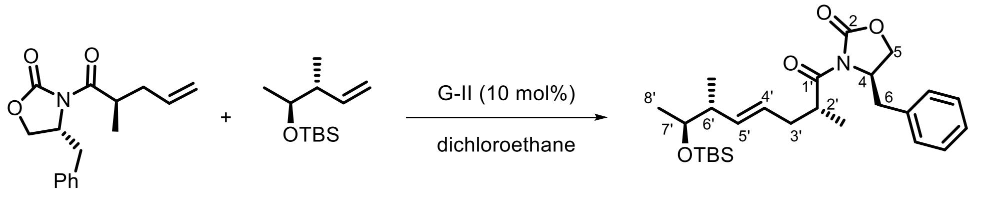

2 **1** **3**

Alkene **2** (0.823 g, 3.01 mmol) and alkene **1** (1.77 g, 8.27 mmol) were dissolved DCE (7 mL) and a solution of Grubbs-II (0.251 g, 0.295 mmol) in DCE (15 mL) was added. The reaction was stirred at reflux for 16 h. The reaction mixture was cooled to room temperature and concentrated *in vacuo*. The crude residue was purified by column chromatography (0-5% EtOAc in petroleum ether) to give **3** (1.00 g, 74%) as a colourless oil. δ_H (500 MHz, $CDCl_3$): 7.35 – 7.32 (2H, m, Ar-H), 7.29 – 7.24 (1H, m, Ar-H), 7.21 (2H, d, *J* 7.5, Ar-H), 5.50 – 5.43 (1H, dd, *J* 15.5, 7.5, 5′-H), 5.43 – 5.31 (1H, m, 4′-H), 4.70 – 4.62 (1H, m, 4-H), 4.19 (2H, d, *J* 5.0, 5-H_2), 3.80 (1H, m, 2′-H), 3.74 – 3.64 (1H, m, 7′-H), 3.29 (1H, dd, *J* 13.5, 3.5, 6-*H*H), 2.80 (1H, dd, *J* 13.5, 9.5, 6-H*H*), 2.42 (1H, m, 3′-*H*H), 2.23 – 2.07 (2H, m, 3′-H*H* and 6′-H), 1.24 (3H, d, *J* 7.0, 2′-CH_3), 1.04 (3H, d, *J* 6.0, 8′-H_3), 0.96 (1H, d, *J* 7.0, 6′-CH_3), 0.90 (9H, s, $SiC(CH_3)_3$), 0.05 (6H, s, $SiCH_3$). δ_C (126 MHz, $CDCl_3$): 176.8 (C-1′), 153.2 (C-2), 135.7 (C-5′), 135.5 (Ar-C), 129.6 (2 × Ar-C), 129.06 1 (2 × Ar-C), 127.5 (Ar-C), 126.7 (C-4′), 71.9 (C-7′), 66.1 (C-5), 55.5 (C-4), 44.3 (C-6′), 38.0 (C-6), 37.96 (C-2′), 36.85 (C-3′), 26.0 ($SiC(\underline{C}H_3)_3$), 20.7 (C-8′), 18.2 ($Si\underline{C}(CH_3)$)), 16.9 (2′-CH_3), 16.2 (6′-CH_3), –4.2 ($SiCH_3$), –4.7 ($SiCH_3$). HRMS calc. for $[C_{26}H_{41}NO_4Si+Na]^+$ 482.2697, found 482.2694. ν_{max} (neat)/cm^{-1} 2957, 2927, 2855, 1779 , 1698, 1455, 1381, 1249.

(2*R*,6*R*,7*S*,*E*)-7-((*tert*-Butyldimethylsilyl)oxy)-2,6-dimethyloct-4-en-1-ol S9

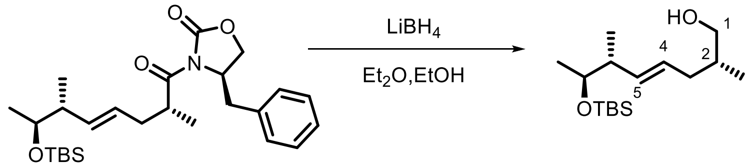

3 **S9**

Oxazolidinone **3** (0.80 g, 1.74 mmol) was dissolved in Et_2O (10 mL) and the solution was cooled to 0 °C. EtOH (0.7 mL, 12.0 mmol) was added, followed by the dropwise addition of $LiBH_4$ (1.60 mL, 3.20 mmol, 2.0 M in THF). The reaction mixture was stirred at 0 °C for 1 h, then at room temperature for 0.5 h. The reaction was quenched by the addition of aq. NaOH (4 mL, 1 M). The layers were separated, and the aqueous layer was extracted with Et_2O (3 × 5 mL). The combined organic extracts were dried over Na_2SO_4, filtered and concentrated *in vacuo*. The crude residue was purified by column chromatography (40% EtOAc in petroleum ether) to give **S9** (0.446 g, 89%) as a colourless oil. $[\alpha]_D^{25}$= +80.0 (*c* 1.0, $CHCl_3$). δ_H (400 MHz, $CDCl_3$) 5.36 (2H, m, 4-H and 5-H), 3.65 (1H, m, 7-H), 3.47 (1H, m, 1-*H*H), 3.42 (1H, m, 1-H*H*), 2.08 (1H, m, 6-H), 2.04 (1H, m, 3-*H*H), 1.89 (1H, m, 3-*H*H), 1.66 (1H, m, 2-H), 1.00 (3H, d, *J* 6.5, 8-H_3), 0.93 (3H, d, *J* 6.5, 6-H_3), 0.88 (3H, d, *J* 6.5, 2-CH_3), 0.85 (9H, s, $SiC(CH_3)_3$), 0.03 (3H, s, $SiCH_3$), 0.03 (3H, s, $SiCH_3$). δ_C (101MHz, $CDCl_3$) 134.6 (C-5), 128.2 (C-4), 72.1 (C-7), 68.2 (C-1), 44.4 (C-6), 36.8 (C-3), 36.1 (C-2), 26.1 ($Si\underline{C}(CH_3)_3$), 26.0 ($SiC(\underline{C}H_3)_3$), 20.8 (C-8), 16.5 (2-CH_3), 16.2 (6-CH_3), –4.2 ($SiCH_3$), –4.7 ($SiCH_3$). HRMS calc. for $[C_{16}H_{34}O_2Si+Na]^+$ 309.2220, found 309.2221. ν_{max} (neat)/cm^{-1} 3339, 2957, 2928, 2857, 1462, 1374, 1252.

(2*R*,6*R*,7*S*,*E*)-7-((*tert*-Butyldimethylsilyl)oxy)-2,6-dimethyloct-4-enal 4

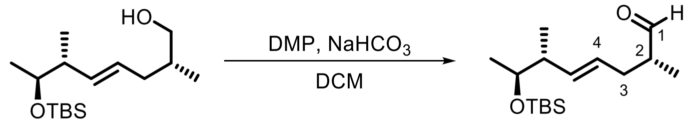

S9 **4**

Alcohol **S9** (140 mg, 0.52 mmol) was dissolved in CH_2Cl_2 (15 mL), $NaHCO_3$ was added and the reaction mixture cooled to 0 °C. A solution of DMP (0.3 M in CH_2Cl_2, 1.8 mL, 0.68 mmol) was added dropwise and the reaction mixture was stirred at room temperature for 1.5 h. The reaction was quenched by addition of sat. aq. $Na_2S_2O_3$ (10 mL), the layers were separated, and the aqueous layer was extracted with CH_2Cl_2 (3 × 10 mL). The combined organic extracts were dried over $MgSO_4$, filtered and concentrated *in vacuo*. The crude residue was purified by column chromatography (5% EtOAc in petroleum ether) to give aldehyde **4** as a colourless oil (125 mg, 89%). $[\alpha]_D^{25}$= +120 (*c* 1.0, $CHCl_3$). δ_H (400 MHz, $CDCl_3$) 9.65 (1H, d, *J* 1.5, 1-H), 5.46 (1H, ddt, *J* 15.5, 8.0, 1.0, 4-H), 5.33 (1H, m, 5-H),

3.67 (1H, qd, J 6.0, 4.0, 7-H), 2.40 (2H, m, 2-H and 3*H*H), 1.95 (2H, m, 6-H, 3*H*H), 1.09 (3H, d, J 7.0, 2-CH_3), 1.02 (3H, d, J 7.0, 8-H_3), 0.95 (3H, d, J 7.0, 6-H_3), 0.88 (9H, s, $C(CH_3)_3$), 0.03 (3H, s, $SiCH_3$), 0.03 (3H, s, $SiCH_3$). δ_C (101 MHz, $CDCl_3$) 205.0 (C-1), 135.7 (C-4), 126.0 (C-5), 71.8 (C-7), 46.3 (C-2), 44.3 (C-6), 33.9 (C-3), 25.9 ($SiC(\underline{C}H_3)_3$), 20.8 (C-8), 18.1 ($Si\underline{C}(CH_3)_3$), 16.2 (6-CH_3), 13.1 (2-CH_3), –4.3 ($SiCH_3$), –4.8 ($SiCH_3$). HRMS calc. for $[C_{16}H_{32}O_2Si+Na]^+$ 307.2064, found 307.2065. ν_{max} (neat)/cm^{-1} 2957, 2928, 2956, 1727, 1461, 1374, 1252.

(4*R*,8*R*,9*S*,*E*)-9-((tert-Butyldimethylsilyl)oxy)-3-hydroxy-4,8-dimethyl-1-(2-thioxothiazolidin-3-yl)dec-6-en-1-one 6

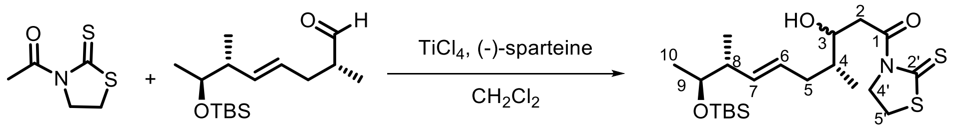

5 4 6

N-acetylthiazolidine-2-thione **5** (0.221 g, 1.37 mmol) was dissolved in CH_2Cl_2 (3 mL) and the solution cooled to 0 °C. A solution of $TiCl_4$ (1 M in CH_2Cl_2, 2.74 mL, 2.74 mmol) was added and a colour change from yellow to bright orange was observed. The reaction mixture was stirred for 10 mins and then cooled to –40 °C. (–)-Sparteine (0.315 mL, 0.457 mmol) was added and a colour change from orange to dark purple was observed. The reaction mixture was stirred at –40 °C for 1 h and then cooled to –78 °C. Aldehyde **4** (0.130 g, 0.457 mmol) in CH_2Cl_2 (2 mL) was then added and the reaction mixture was stirred at –78 °C a further 1 h. The reaction was quenched by dropwise addition of sat. aq. NH_4Cl (8 mL), the layers were separated, and the aqueous layer was extracted with CH_2Cl_2 (3 × 10 mL). The combined organic extracts were dried over Na_2SO_4, filtered and concentrated *in vacuo*. The crude residue was purified by column chromatography (10 – 50% Et_2O in pentane) to give **6** (0.147 g, 72%) as a colourless oil. δ_H (400 MHz, $CDCl_3$): 5.52 – 5.30 (2H, m, 6-H and 7-H), 4.66 – 4.53 (2H, m, 4′-H_2), 4.07 (1H, ddd, J 9.5, 4.5, 3.0, 3-H), 3.97 (1H_{dias}, ddd, J 10.0, 6.0, 2.0, 3-H), 3.68 (1H, qd, J 6.0, 4.0, 9-H), 3.56 – 3.37 (1H, m, 2-*H*H), 3.35 – 3.25 (2H, m, 5′-H_2), 2.59 – 2.40 (1H, br s, OH), 2.33 – 2.17 (1H, m, 5-*H*H), 2.16 – 2.07 (1H, m, 8-H), 2.00 – 1.81 (1H, m, 2-H*H*), 1.76 – 1.52 (1H, m, 4-H), 1.03 (3H, d, J 6.0, 10-H_3), 0.96 (3H, d, J 7.0, 8-CH_3), 0.95 (3H, d, J 6.5, 8_{dias}-CH_3), 0.94 (3H, d, J 7.0, 4-CH_3), 0.91 (3H, d, J 7.0, 4_{dias}-CH_3), 0.88 (9H, s, $C(CH_3)_3$), 0.88 (9H, s, $_{dias}C(CH_3)_3$), 0.03 (6H, s, $SiCH_3$). δ_C (126 MHz, $CDCl_3$): 202.0 (C-2′), 202.0 (C-2′), 174.8 (C-1), 174.7 (C-1_{dias}), 134.8 (C-7), 134.8 (C-7_{dias}), 128.2 (C-6), 128.1 (C-6_{dias}), 72.1 (C-9), 71.8 (C-3), 70.9 (C-3_{dias}), 55.9 (C-4′), 44.4 (C-8), 44.4 (C-8_{dias}), 43.6 (C-2), 42.8 (C-2_{dias}), 38.7(C-4), 38.6 (C-4_{dias}), 36.6 (C-5), 36.0 (C-5_{dias}), 28.5 (C-5′), 26.0 ($SiC(\underline{C}H_3)_3$), 20.7 (C-10), 18.3 ($Si\underline{C}(CH_3)_3$), 16.2 (8-CH_3), 16.1 (8-CH_{3dias}), 14.4 (4-CH_3), –4.2 ($SiCH_3$), –4.7 ($SiCH_3$). HRMS calc. for $[C_{21}H_{39}NO_3S_2Si+Na]^+$ 438.2033, found 468.2042. ν_{max} (neat)/cm^{-1} 3472, 2957, 2928, 1693, 1462, 1369, 1280.

(*R*)-6-(2,2,9,9-Tetramethyl-1,3-dioxane-3-carboxamido)propionic acid S22

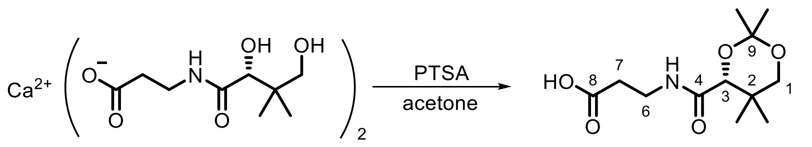

Anhydrous acetone (50 mL) was stirred with 4 Å molecular sieves for 20 mins before calcium D-pantothenate **S21** (1.0 g, 2.09 mmol) and PTSA (0.96 g, 5.56 mmol) were added. The resulting suspension was stirred at room temperature for 16 h and then filtered through a pad of celite which was washed with acetone (30 mL). The solvent was removed *in vacuo* to give a white residue which was dissolved in EtOAc (20 mL) and washed with brine (20 mL). The organic extract was dried over $MgSO_4$, filtered and concentrated *in vacuo*. The crude residue was triturated with hexanes (*ca.* 5 mL) to give **S22** as a white solid (444 mg, 82%). $[\alpha]_D^{25}$= +62.0 (*c* 1.0, $CHCl_3$), $[\alpha]_D^{25}$= +62.0 (*c* 1.0, $CHCl_3$). δ_H (400 MHz, $CDCl_3$) 7.01 (1H, m, NH), 4.10 (1H, s, 3-H), 3.70 (1H, d, *J* 12.0, 1-*H*H), 3.58 (1H, m, 6-*H*H), 3.49 (1H, m, 6-H*H*), 3.30 (1H, d, *J* 12.0, 1-*H*H), 2.69 (2H, t, *J* 6.0, 7-H_2), 1.46 (3H, s, 9-CH_3), 1.43 (3H, s, 9-CH_3), 1.04 (3H, s, 2-CH_3), 0.98 (3H, s, 2-CH_3). δ_C (101 MHz, $CDCl_3$) 176.5 (C-8), 170.3 (C-4), 99.2 (C-9), 77.1 (C-3), 71.5 (C-1), 34.2 (C-6), 33.9 (C-7), 33.0 (C-2), 29.5 (9-CH_3), 22.1 (2-CH_3), 18.9 (2-CH_3), 18.8 (9-CH_3). Data in accordance with the literature.[9]

Pantetheine 1,3-dimethyl ketal 7

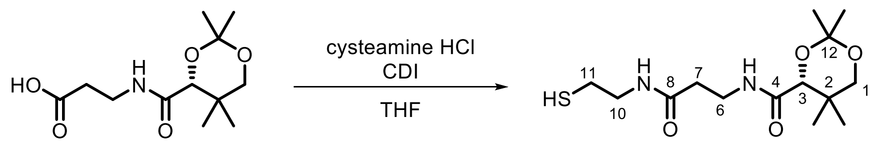

Acid **S22** (410 mg, 1.58 mmol) was dissolved in anhydrous THF (8 mL), CDI (374 mg, 2.30 mmol) was added and the reaction mixture was stirred at room temperature for 30 mins. Cysteamine HCl (262 mg, 3.40 mmol) was then added and the reaction mixture stirred for 16 h. The reaction was quenched by addition of sat. aq. NH_4Cl (10 mL) and extracted with CH_2Cl_2 (3 × 10 mL). The combined organic extracts were washed with brine (20 mL), dried over $MgSO_4$, filtered and concentrated *in vacuo.* The crude residue was purified by column chromatography (EtOAc) to give **7** as a white solid (377 mg, 75%). m.p 99-101 °C. $[\alpha]_D^{25}$= +33.4 (*c* 1.0, MeOH), $[\alpha]_D^{25}$= + 48.0 (*c* 1.0, $CHCl_3$) [10]. δ_H (400 MHz, $CDCl_3$) 6.90 (1H, br s, NH), 6.21 (1H, br s, NH), 4.08 (1H, s, 3-H), 3.70 (1H, d, *J* 12.0, 1-*H*H), 3.57 (2H, m, 6-H_2), 3.47 (2H, m, 10-H_2), 3.27 (1H, d, *J* 12.0, 1-*H*H), 2.66 (2H, q, *J* 7.0, 11-H_2), 2.47 (2H, t, *J* 6.0, 7-H_2), 1.46 (3H, s, 12-CH_3), 1.42 (3H, s, 12-CH_3), 1.36 (1H, t, *J* 8.5, SH), 1.09 (3H, s, 2-CH_3), 0.97 (3H, s, 2-CH_3). δ_C (101 MHz, $CDCl_3$) 171.1 (C-8), 170.2 (C-4),

99.1 (C-12), 77.1 (C-3), 71.4 (C-1), 42.4 (C-10), 36.1 (C-7), 34.8 (C-6), 32.9 (C-2), 29.5 (12-CH_3), 22.1 (2-CH_3), 18.9 (2-CH_3), 18.7 (12-CH_3). Data in accordance with the literature.[11]

***S*-((4*R*,8*R*,9*S*,*E*)-9-((*tert*-Butyldimethylsilyl)oxy)-3-hydroxy-1-oxo-4,8-dimethyldec-6-ene-1-yl)-pantetheine-1′,3′-dimethyl ketal 8**

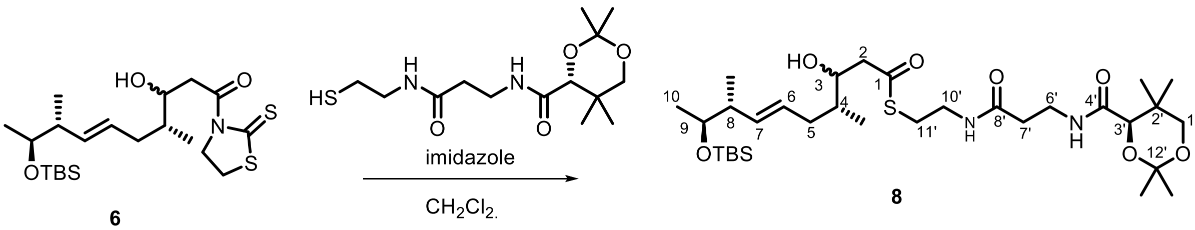

6

8

Thiazolidinethione **6** (37.0 mg, 0.0830 mmol) was dissolved in CH_2Cl_2 (1.0 mL). Imidazole (17.0 mg, 0.249 mmol) and pantetheine **7** (30.4 mg, 0.0955 mmol) were added and the yellow reaction mixture was stirred for 4 h. The solvent was removed *in vacuo* and the crude residue was purified by column chromatography (50 – 80% EtOAc in petroleum ether) to give **8** (48.9 mg, 91%) as a colourless oil. δ_H (400 MHz, $CDCl_3$): 6.99 (1H, br s, NH), 6.43 (1H, br s, NH), 5.55 – 5.21 (2H, m, 6-H and 7-H), 4.06 (1H, s, 3′-H), 4.04 – 3.85 (1H, m, 3-H), 3.66 (2H, m, 1′-*H*H and 9-H), 3.58 – 3.39 (4H, m, 6′-H_2 and 10′-H_2), 3.34 (2H, m, 10′$_{dias}$-H_2), 3.26 (1 H, d, *J* 11.5, 1′-H*H*), 3.16 – 3.03 (2H, m, 11′-*H*H), 3.03 – 2.91 (2H, m, 11′-H*H*), 2.78 – 2.59 (2H, m, 2-H_2), 2.40 (2H, t, *J* 6.5, 7′-H_2), 2.23 – 2.14 (1H, m, 5-*H*H), 2.12 – 2.05 (1H, m, 8-H), 1.86 (1H, m, 5-H*H*), 1.65 – 1.52 (1H, m, 4-H), 1.44 (3H, s, 12′-CH_3), 1.40 (3H, s, 12′-CH_3), 1.01 (3H, s, 2′-CH_3), 1.00 (3H, d, *J* 6.0, 10-H_3), 0.95 (3H, s, 2′-CH3), 0.94 (3H, d, *J* 5.5, 8-CH_3), 0.92 – 0.88 (3H, m, 4-CH_3), 0.86 (9H, s, $C(CH_3)_3$), 0.01 (6H, s, $SiCH_3$). δ_C (101 MHz, $CDCl_3$): 199.5 (C-1), 171.3 (C-8′), 170.6 (C-4′), 134.8 (C-7), 128.0 (C-6), 99.2 (C-12′), 77.2 (C-3′), 72.5 (C-3$_{dias}$), 72.0 (C-3), 71.9 (C-9), 71.5 (C-1′), 48.9 (C-2), 48.1 (C-2$_{dias}$), 44.4 (C-8), 39.3 (C-10′), 39.2 (C-10$_{dias}$), 39.0 (C-4), 38.9 (C-4$_{dias}$), 36.4 (C-5), 36.3 (C-7′), 35.3 (C-6′), 33.1 (C-2′), 29.6 (12′-CH_3), 28.8 (C-11′), 26.0 ($C(\underline{C}H_3)_3$), 22.3 (2′-CH_3), 20.8 (C-10), 19.0 (2′-CH_3), 18.8 (12′-CH_3), 18.2 ($\underline{C}(CH_3)_3$), 16.3 (8-CH_3), 15.2 (C-4$_{dias}$), 14.2 (C-4), –4.2 ($SiCH_3$), –4.7 ($SiCH_3$). HRMS calc. for $[C_{32}H_{60}N_2O_7SSi+H]^+$ 645.3963, found 645.3968. ν_{max} (neat)/cm^{-1} 3321, 2958, 2929, 1658, 1529, 1462, 1376, 1252.

***S*-((4*R*,8*R*,9*S*,*E*)-9-((*tert*-Butyldimethylsilyl)oxy)-1,3-dioxo-4,8-dimethyldec-6-ene-1-yl)-pantetheine-1′,3′-dimethyl ketal S10**

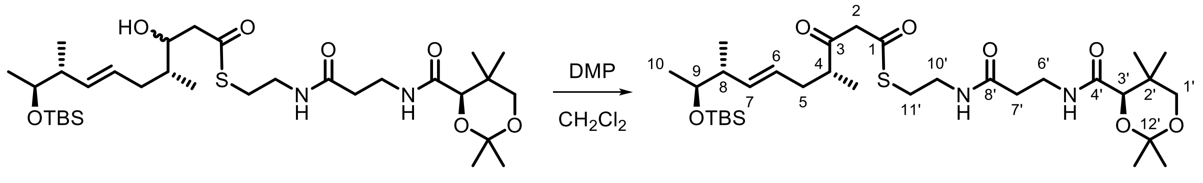

8

S10

Protected pantetheine **8** (0.22 g, 0.3411 mmol) was dissolved in CH_2Cl_2 (7 mL) and the solution cooled to 0 ˚C. DMP (0.159 g, 0.375 mmol) was added and the reaction mixture was stirred at 0 ˚C for 1 h. The reaction mixture was quenched by the addition of 10 % aq. $Na_2S_2O_3$ (3.5 mL) and sat. aq. $NaHCO_3$ (3.5 mL). The layers were separated and the aqueous layer was extracted with CH_2Cl_2 (3 × 10 mL). The combined organic extracts were washed with brine (3 x 10 mL) and sat. aq. $NaHCO_3$ (3 × 10 mL), dried over Na_2SO_4, filtered, and concentrated *in vacuo*. The crude residue was purified by column chromatography (10 – 40% EtOAc in petroleum ether) to give **S10** (0.134 g, 61%) as a colourless oil. $[\alpha]_D^{24}$ = + 5.0 (*c* 0.8, $CHCl_3$). δ_H (500 MHz, $CDCl_3$) 12.60 (1H, s, 2_{enol}-H), 7.04 (1H, br s, NH), 6.24 (1H, br s, NH), 5.54 – 5.37 (1H, m, 7-H), 5.35 – 5.20 (1H, m, 6-H), 4.07 (1H, s, 3′-H), 3.72 (2H, d, *J* 3.0, 2_{keto}-H_2), 3.68 (1H, d, *J* 12.0, 1′-*H*H), 3.68 – 3.62 (1H, m, 9-H), 3.63 – 3.52 (2H, m, 6′-H_2), 3.55 – 3.37 (2H, m, 10′-H_2), 3.27 (1H, d, *J* 11.5, 1′-H*H*), 3.10 – 3.02 (2H, m, 11′-H_2), 2.66 (1H, tq, *J* 10.0, 7.0 4_{keto}-H), 2.43 (2H, td, *J* 6.0, 3.0, 7′-H_2), 2.39 – 2.26 (1H, m, 5-*H*H), 2.22 (1H, m, 4_{enol}-H), 2.15 – 2.05 (3H, m, 5-H*H* and 8-H), 1.46 (3H, s, 12′-CH_3), 1.41 (3H, s, 12′-CH_3), 1.12 (3H, d, *J* 7.0, 4_{enol}-CH_3), 1.10 (3H, d, *J* 7.0, 4_{keto}-CH_3), 1.03 (3H, s, 2′-CH_3), 1.01 (3H, d, *J* 6.0, 10-H_3), 1.00 (3H, d, *J* 6.0, 10_{enol}-H_3), 0.97 (3H, s, 2′-CH_3), 0.94 (3H, d, *J* 7.0, 8-CH_3), 0.93 (3H, d, *J* 7.0, 8_{enol}-CH_3), 0.87 (9H, s, $C(CH_3)_3$), 0.02 (3H, s, $SiCH_3$), 0.02 (3H, s, $SiCH_3$). δ_C (126 MHz, $CDCl_3$) 205.7 (C-3), 194.3 (C-1_{enol}), 192.4 (C-1_{keto}), 180.9 (C-3_{enol}), 171.5 (C-8′), 171.3 (C-$8'_{enol}$), 170.3 (C-$4'_{enol}$), 170.2 (C-4′), 136.2 (C-7), 135.7 (C-7_{enol}), 126.7 (C-6_{enol}), 126.1 (C-6), 99.2 (C-$12'_{enol}$), 99.2 (C-12′), 98.3 (C-2_{enol}), 77.3 (C-3′), 72.0 (C-9_{enol}), 71.9 (C-9_{keto}), 71.6 (C-1′), 55.9 (C-2), 47.2 (C-4), 44.4 (C-8), 44.4 (C-8_{enol}), 39.9 (C-4_{enol}), 39.8 (C-$10'_{enol}$), 39.3 (C-10′), 37.4 (C-5), 36.8 (C-5_{enol}), 36.4 (C-7′), 34.9 (C-$6'_{enol}$), 34.8 (C-6′), 33.1 (C-2′), 29.6 (12′-CH_3), 29.3 (C-11′), 27.9 (C-$11'_{enol}$), 26.0 ($C(\underline{C}H_3)_3$), 22.3 (2′-CH_3), 21.0 (C-10_{keto}), 20.9 (C-10_{enol}), 19.0 (2′-CH_3), 18.8 (12′-CH_3), 18.2 ($\underline{C}(CH_3)_3$), 17.4 (4_{enol}-CH_3), 16.5 (8_{keto}-CH_3), 16.3 (8_{enol}-CH_3), 15.7 (4_{keto}-CH_3), –4.2 ($SiCH_3$), –4.7 ($SiCH_3$). HRMS calc. for $[C_{32}H_{58}N_2O_7SSi+Na]^+$ 665.3626, found 665.3624. ν_{max} (neat)/cm^{-1} 3313, 2957, 2930, 2858, 1722, 1659, 1613, 1525, 1461, 1375, 1252.

***S*-((4*R*,8*R*,9*S*,*E*)-9-((*tert*-Butyldimethylsilyl)oxy)-1,3-dioxo-4,8-dimethyldec-6-ene-1-yl)-pantetheine 9**

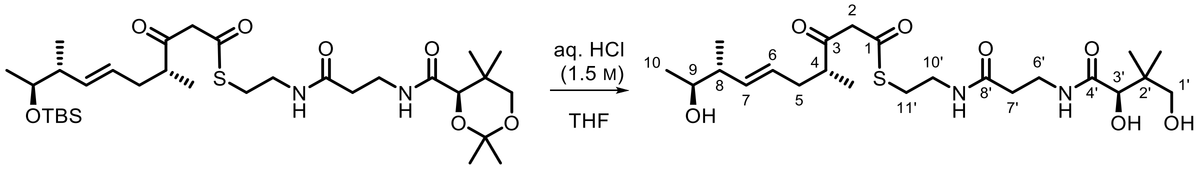

S10 **9**

S10 (0.1113 g, 0.1731 mmol) was dissolved in THF (3.0 mL) and aq. HCl (1.5 M, 0.26 mL, 0.389 mmol) was added. The reaction mixture was stirred at room temperature for 2.3 h. Saturated aq. $NaHCO_3$ (1.5 mL) was added and the layers were separated. The aqueous layer was extracted with EtOAc (4 × 5 mL). The combined organic extracts were dried over Na_2SO_4, filtered and concentrated *in vacuo*. The crude residue was purified by column chromatography (0 – 10% MeOH in CH_2Cl_2) to give **9** (0.0754 g, 89%) as a colourless oil. $[\alpha]_D^{25}$ = + 20.0 (*c* 0.1, $CHCl_3$). δ_H (500 MHz, $CDCl_3$): 5.50 (1H, s, 2_{enol}-H), 5.48 – 5.28 (2H, m, 6-H and 7-H), 3.90 (1H, s, 3′-H), 3.89 – 3.81 (2H, m, 2_{keto}-H_2), 3.63 – 3.57 (1H, m, 9-H), 3.55 – 3.41 (3H, m, 6′-H_2 and 1′-*H*H), 3.41 – 3.33 (3H, m, 10′-H_2 and 1′-H*H*), 3.06 (2H, td, *J* 7.0, 2.5, 11′-H_2), 2.75 (1H, tq, *J* 10.5, 7.0, 4_{keto}-H), 2.42 (2H, t, *J* 6.5, 7′-H_2), 2.40 – 2.31 (1H, m, 5-*H*H), 2.36 – 2.24 (1H, m, 4_{enol}-H), 2.20 – 2.07 (2H, m, 5-H*H* and 8-H), 1.13 (3H, d, *J* 6.5, 4_{enol}-CH_3), 1.09 (3H, d, *J* 6.5, 4_{keto}-CH_3), 1.08 (3H, d, *J* 6.5, 10_{keto}-H_3), 1.07 (3H, d, *J* 6.5, 10_{enol}-H_3), 0.98 (3H, d, *J* 7.0, 8_{keto}-CH_3), 0.96 (3H, d, *J* 7.0, 8_{enol}-CH_3), 0.92 (6H, s, 2′-CH_3). δ_C (126 MHz, $CDCl_3$) 207.7 (C-3), 195.4 (C-1_{enol}), 193.7 (C-1), 180.9 (C-3_{enol}), 176.0 (C-$4'_{enol}$), 176.0 (C-4′), 173.9 (C-8′), 173.9 (C-$8'_{enol}$), 136.4 (C-7), 136.1 (C-7_{enol}), 128.7 (C-6_{enol}), 128.3 (C-6), 99.3 (C-2_{enol}), 77.3 (C-3′), 72.0 (C-9), 70.3 (C-1′), 56.6 (C-2), 48.0 (C-4), 45.2 (C-8), 45.2 (C-8_{enol}), 40.8 (C-4_{enol}), 40.5 (C-10′), 39.8 (C-$10'_{enol}$), 38.4 (4_{enol}-CH_3), 36.8 (C-5), 36.4 (C-7′), 36.3 (C-$6'_{enol}$), 36.3 (C-6′), 29.7 (C-11), 28.4 (C-11_{enol}), 21.3 (C-2′), 20.9 (2′-CH_3 × 2), 20.3 (C-10), 20.1 (C-10_{enol}), 17.7 (4-CH_3), 16.5 (8-CH_3), 16.4 (8_{enol}-CH_3), 15.9 (4_{enol}-CH_3). HRMS calc. for $[C_{23}H_{40}N_2O_7S+Na]^+$ 511.2448, found 511.2458. ν_{max} (neat)/cm^{-1} 3301, 2966, 2928, 2875, 1719, 1614, 1645, 1531, 1453, 1405, 1375, 1291.

(2-^{13}C)-*S*-((4*R*,8*R*,9*S*,*E*)-9-((*tert*-Butyldimethylsilyl)oxy)-1,3-dioxo-4,8-dimethyldec-6-ene-1-yl)-pantetheine 9

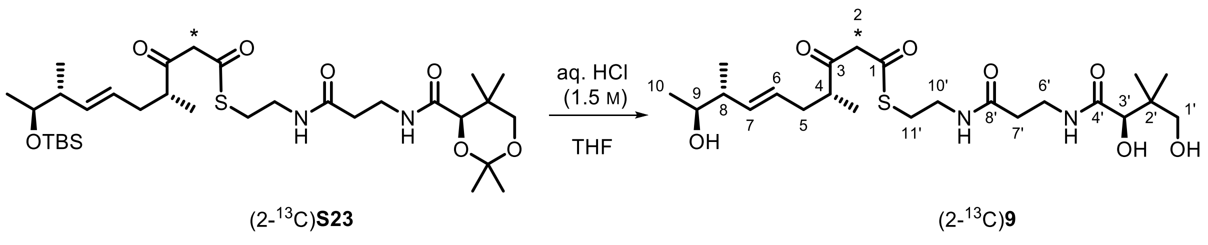

(2-^{13}C)**9** was prepared in accordance with procedures of compounds **S7** to **9**. $[\alpha]_D^{25}$ = +12.0 (*c* 0.5, $CHCl_3$). δ_H (500 MHz, $CDCl_3$) 5.48 – 5.32 (2H, m, 6-H and 7-H), 3.89 (1H, s, 3′-H), 3.66 – 3.55 (1H, m, 9-H), 3.55 – 3.42 (3H, m, 6′-H_2 and 1′-*H*H), 3.41 – 3.33 (3H, m, 10′-H_2 and 1′-H*H*), 3.06 (2H, t, *J* 7.0, 11′-H_2), 2.75 (1H, tq, *J* 10.5, 7.0, 4_{keto}-H), 2.42 (2H, t, *J* 6.5, 7′-H_2), 2.39 – 2.32 (1H, m, 5-*H*H), 2.31 – 2.23 (1H, m, 4_{enol}-CH_3), 2.20 – 2.03 (2H, m, 5-H*H* and 8-H), 1.13 (3H, d, *J* 6.0, 4_{enol}-CH_3), 1.08 (3H, d, *J* 6.0, 4_{keto}-CH_3), 1.08 (3H, d, *J* 6.5, 10_{keto}-H_3), 1.06 (3H, d, *J* 6.5, 10_{enol}-H_3), 0.98 (3H, d, *J* 7.0, 8_{keto}-CH_3), 0.96 (3H, d, *J* 7.0, 8_{enol}-CH_3), 0.92 (6H, s, 2′-CH_3). δ_C (126 MHz, $CDCl_3$) 207.8 (d, *J* 36.0, C-3), 193.7 (d, *J* 46.3, C-1_{enol}), 192.3 (C-1), 180.9 (d, *J* 64.0 C-3_{enol}), 176.0 (C-4′), 174.0 (C-8′), 173.9 (C-8′$_{enol}$), 136.4 (C-7), 136.1 (C-7_{enol}), 128.7 (C-6_{enol}), 128.3 (C-6), 100.22 – 98.06 (m, C-2_{enol}), 77.3 (C-3′), 72.0 (C-9), 70.3 (C-1′), 59.9 – 54.4 (m, C-2), 50.1 (C-4_{keto}), 45.3 (C-8), 45.2 (C-8_{enol}), 40.4 (C-10′$_{keto}$), 39.9 (C-10′$_{enol}$), 36.8 (C-5), 36.4 (C-7′), 36.3 (C-6′$_{enol}$), 36.3 (C-6′), 29.7 (C-11′), 28.4 (C-11′$_{enol}$), 21.3 (C-2′), 20.9 (2′-CH_3 × 2), 20.3 (C-10), 20.1 (C-10_{enol}), 17.7 (4_{enol}-CH_3), 16.5 (8-CH_3), 16.4 (8_{enol}-CH_3), 15.8 (4-CH_3). HRMS calc. for $[^{13}CC_{22}H_{40}N_2O_7S+H]^+$ 490.2663, found 490.2665. ν_{max} (neat)/cm^{-1} 3302, 2967, 2930, 2875, 1718, 1646, 1598, 1530, 1452, 1374, 1267.

(*S*)-3-(*tert*-Butyldimethylsilyloxy)dihydrofuran-2(3*H*)-one S12

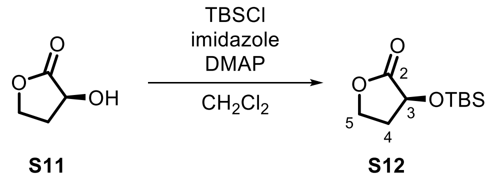

Lactone **S11** (200 mg, 1.96 mmol) was dissolved in CH_2Cl_2 (10 mL). Imidazole (200 mg, 2.94 mmol), TBSCl (532 mg, 3.53 mmol) and DMAP (24.0 mg, 0.20 mmol) were added and the reaction was stirred for 16 h at room temperature. The reaction was quenched by addition of H_2O (10 mL), the layers were separated and the aqueous layer was extracted with CH_2Cl_2 (3 × 10 mL). The combined organic extracts were dried over $MgSO_4$, filtered and concentrated *in vacuo* to give **S12** as a colourless oil (413 mg, 97%). $[\alpha]_D^{25}$= –10.0 (*c* 1.0, $CHCl_3$). δ_H (400 MHz, $CDCl_3$) 4.38 (2H, m, 5-*H*H and 3-H), 4.18 (1H, tdd, *J* 9.0, 6.5, 0.5, 5-*H*H), 2.45 (1H, dddd, *J* 12.5, 7.5, 6.5, 3.5, 4-*H*H), 2.21 (1H, dddd, *J* 12.5, 9.0, 8.5, 8.0, 4-*H*H), 0.90 (9H, s, $SiC(CH_3)_3$), 0.16 (3H, s, $SiCH_3$), 0.14 (3H, s, $SiCH_3$). δ_C (101

MHz, $CDCl_3$) 176.0 (C-2), 68.4 (C-3), 64.9 (C-5), 32.5 (C-4), 25.8 (SiC(<u>C</u>H_3)$_3$), 18.4 (Si<u>C</u>(CH_3)$_3$), –4.6 ($SiCH_3$), –5.1 ($SiCH_3$). HRMS calc. for $[C_{10}H_{20}O_3Si+Na]^+$ 239.1074, found 239.1077. ν_{max} (neat)/cm^{-1} 2935, 2864, 1784, 1151.

(3*S*)-3-(*tert*-Butyldimethylsilyloxy)-2-methyltetrahydrofuran-2-ol S13

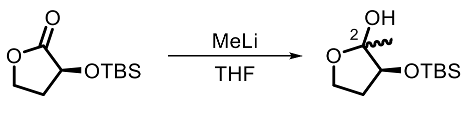

S12 **S13**

Lactone **S12** (2.40 g, 11.2 mmol) was dissolved in anhydrous THF (25 mL) and the solution cooled to –78 °C. MeLi (1.6 M in Et_2O, 7.69 mL, 12.3 mmol) was added and the reaction mixture was stirred for 3 h at –78 °C. The reaction was quenched by the addition of sat. aqueous NH_4Cl (15 mL), the layers were separated and the aqueous layer was extracted with EtOAc (3 × 20 mL). The combined organic extracts were dried over $MgSO_4$, filtered and concentrated *in vacuo*. The crude residue was purified by column chromatography (20% EtOAc in petroleum ether) to give lactol **S13** as a colourless oil (2.1 g, 81%). ^{1}H NMR showed a *dr* of 3:1, spectral data reported for the major product. δ_H (400 MHz, $CDCl_3$) 4.03 (1H, m, 3-H), 4.00 (1H, m, 5-*H*H), 3.80 (1H, m, 5-*H*H), 2.14 (1H, m, 4-*H*H), 1.81 (1H, m, 4-*H*H), 1.39 (3H, s, CH_3), 0.91 (9H, s, SiC(CH_3)$_3$), 0.12 (6H, s, $SiCH_3$ × 2). δ_C (101 MHz, $CDCl_3$) 102.6 (C-2), 76.1 (C-3), 64.7 (C-5), 33.5 (C-4), 25.7 (SiC(<u>C</u>H_3)$_3$), 25.1 (CH_3), 18.0 (Si<u>C</u>(CH_3)$_3$), –4.7 ($SiCH_3$), –5.1 ($SiCH_3$). HRMS calc. for $[C_{11}H_{23}O_2Si+H–H_2O]^+$ 215.1462, found 215.1469. ν_{max} (neat)/cm^{-1} 3434, 2961, 2929, 2864, 1252, 1103.

(*S*)-3-(*tert*-Butyldimethylsilyloxy)-4-methylpent-4-en-1-ol S14

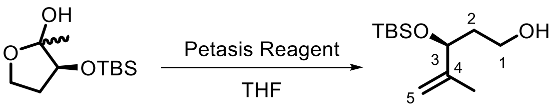

S13 **S14**

Lactol **S13** (112 mg, 0.50 mmol) was dissolved in anhydrous THF (2 mL) in a boiling tube. Petasis reagent (0.21 M, 7.14 mL, 1.5 mmol,) was added and the reaction was stirred at 75 °C for 16 h. The reaction mixture was cooled to room temperature, diluted with petroleum ether (10 mL) and filtered through celite. The filtrate was washed with 2 M HCl (10 mL) and brine (*ca.* 10 mL), dried over $MgSO_4$, filtered and concentrated *in vacuo*. The crude residue was purified by column chromatography (20% EtOAc in petroleum ether) to give alcohol **S14** as a pale orange oil (67 mg, 58%). $[\alpha]_D^{25} = -12$ (*c* 0.5, $CHCl_3$). δ_H (400 MHz, $CDCl_3$) 4.98 (1H, s, 5-*H*H), 4.84 (1H, s, 5-H*H*), 4.29 (1H, t, *J* 5.5, 3-H), 3.73 (2H, m, 1-H_2), 2.36 (1H, t, *J* 5.5, OH), 1.79 (2H, q, *J* 6.5, 5.5, 2-H_2), 1.69 (3H, s, 4-CH_3), 0.91 (9H, s, SiC(CH_3)$_3$), 0.08 (3H, s, $SiCH_3$), 0.03 (3 H, s, $SiCH_3$). δ_C (101 MHz, $CDCl_3$) 147.0 (C-4), 111.2 (C-5), 75.9 (C-3), 60.5 (C-1), 37.7 (C-2), 26.0 (SiC(<u>C</u>H_3)$_3$), 18.3 (Si<u>C</u>(CH_3)$_3$), 18.1

(4-CH_3), –4.6 ($SiCH_3$), –5.2 ($SiCH_3$). HRMS calc. for $[C_{12}H_{26}O_2Si+Na]^+$ 253.1594, found 253.1595. ν_{max} (neat)/cm^{-1} 3430, 2955, 2930, 2858, 2251, 1471, 1256.

(4*S*,3*R*,*E*)-4-(*tert*-Butyldimethylsilyloxy)-1-iodo-3-methylpent-1-ene S6

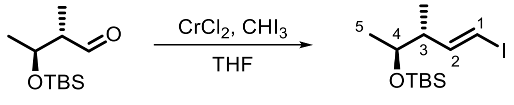

Anhydrous THF (10 mL) was stirred vigorously at 0 °C. $CrCl_2$ (3.58 g, 29.3 mmol) and CHI_3 (2.55 g, 6.48 mmol) were added to the stirred THF portionwise over 10 mins. Aldehyde **S5** (700 mg, 3.24 mmol) in anhydrous THF (16 mL) was then added dropwise. The resulting solution was stirred at 0 °C for 20 mins, followed by 2 h at room temperature. The reaction was quenched by addition of sat. aq. $Na_2S_2O_3$ (30 mL) and then stirred for a further 30 mins. The layers were separated and the aqueous layer extracted with Et_2O (3 × 25 mL), the combined organic extracts were dried over $MgSO_4$ and the solvent removed *in vacuo*. The crude residue was purified by column chromatography (100% petroleum ether) to give vinyl iodide **S6** as a pale yellow oil (350 mg, 32%). $[\alpha]_D^{25}$= +17.0 (*c* 1.0, $CHCl_3$). δ_H (400 MHz, $CDCl_3$) 6.46 (1H, dd, *J* 14.5, 8.5, 2-H), 5.97 (1H, dd, *J* 14.5, 1.0, 1-H), 3.64 (1H, qd, *J* 6.0, 5.0, 4-H), 2.17 (1H, m, 3-H), 1.07 (3H, d, *J* 6.0, 5-H_3), 0.98 (3H, d, *J* 7.0, 3-CH_3), 0.89 (9H, s, $Si(CH_3)_3$), –0.04 (6H, s, 2 x $SiCH_3$). δ_C (101 MHz, $CDCl_3$) 149.2 (C-2), 74.9 (C-1), 71.4 (C-4), 48.5 (C-3), 26.0 ($SiC(\underline{C}H_3)_3$), 21.4 (C-5), 18.2 ($Si\underline{C}(CH_3)_3$), 15.9 (3-CH_3), –4.2 ($SiCH_3$), –4.6 ($SiCH_3$). HRMS calc. for $[C_{12}H_{25}IOSi+Na]^+$ 363.0612, found 363.0608. ν_{max} (neat)/cm^{-1} 2955, 2928, 2856, 2462, 1372, 1250.

(*S*)-1,3-Bis(*tert*-butyldimethylsilyloxy)-4-methylpent-4-ene S15

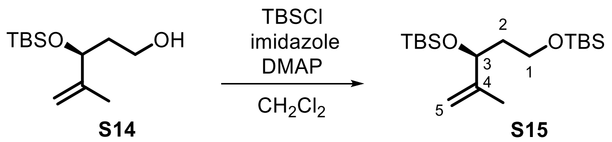

Alcohol **S14** (200 mg, 0.77 mmol) was dissolved in anhydrous CH_2Cl_2 (5 mL) and TBSCl (142 mg, 1.16 mmol), imidazole (206 mg, 1.37 mmol) and DMAP (5 mg, 0.08 mmol) were added. The reaction mixture was stirred at room temperature for 16 h before the addition of H_2O (5 mL). The aqueous layer was extracted with CH_2Cl_2 (3 × 10 mL), the combined organic extracts were dried over $MgSO_4$, and the solvent removed *in vacuo*. The crude residue was purified by filtration through a pad of silica which was washed with EtOAc (*ca.* 40 mL). The solvent was removed *in vacuo* to give silyl ether **S15** as a colourless oil (268 mg, 94%). $[\alpha]_D^{25}$= –14 (*c* 0.5, $CHCl_3$). δ_H (400 MHz, $CDCl_3$) 4.86 (1H, s, 5-

*H*H), 4.75 (1H, s, 5-*H*H), 4.21 (1H, dd, *J* 7.5, 5.0, 3-H), 3.63 (2H, m, 1-H_2), 1.73-1.64 (2H, m, 2-H_2), 1.68 (3H, s, 4-CH_3), 0.89 (9H, s, $SiC(CH_3)_3$), 0.89 (9H, s, $SiC(CH_3)_3$), 0.04 (3H, s, $SiCH_3$), 0.04 (6H, s, $SiCH_3$ × 2), 0.01 (3H, s, $SiCH_3$). δ_C (101 MHz, $CDCl_3$) 148.0 (C-4), 110.6 (C-5), 73.6 (C-3), 59.9 (C-1), 39.7 (C-2), 26.1 ($SiC(\underline{C}H_3)_3$), 26.0 ($SiC(\underline{C}H_3)_3$), 18.4 ($Si\underline{C}(CH_3)_3$), 18.4 ($Si\underline{C}(CH_3)_3$), –4.6 ($SiCH_3$), –5.0 ($SiCH_3$), –5.1 ($SiCH_3$), –5.1 ($SiCH_3$). HRMS calc. for $[C_{18}H_{40}O_2Si_2+Na]^+$ 366.2465, found 366.2456. ν_{max} (neat)/cm^{-1} 2954, 2929, 2857, 1471, 1361, 1253.

(3*S*,4*R*,8*R*,9*S*,*E*)-1,3,9-Tris(*tert*-butyldimethylsilyloxy)-4,8-dimethyldec-6-ene S16

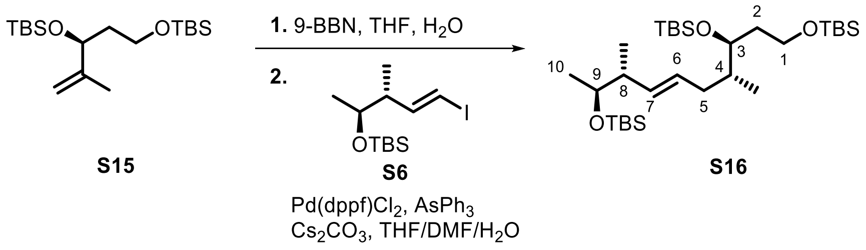

S15 **S6** **S16**

Alkene **S15** (150 mg, 0.40 mmol) was dissolved in degassed anhydrous THF (1.5 mL) and the solution cooled to –78 °C. 9-BBN in THF (0.5 M, 2.40 mL, 1.20 mmol) was added and the resulting solution was allowed to warm to room temperature and stirred for 14 h. The reaction was quenched by the addition of degassed H_2O (1.5 mL) and stirred for a further 1 h. In a separate flask, vinyl iodide **S6** (115 mg, 0.34 mmol) was dissolved in degassed DMF (9 mL). Cs_2CO_3 (397 mg, 1.22 mmol), Pd(dppf)Cl_2 (66 mg, 0.09 mmol) and $AsPh_3$ (26 mg, 0.09 mmol) were added and the mixture was stirred for 10 mins at room temperature. The previously prepared borane solution was then added dropwise to this mixture and the reaction stirred at room temperature for 8 h. The reaction was quenched by the addition of H_2O (10 mL) and the mixture was filtered through a pad of celite. The aqueous layer was extracted with Et_2O (3 × 30 mL) and the combined organic extracts were dried over $MgSO_4$ and the solvent removed *in vacuo*. The crude residue was purified by column chromatography (petroleum ether) to give silyl ether **S16** as a colourless oil (168 mg, 89%). $[\alpha]_D^{25}$= –2.0 (*c* 1.0, $CHCl_3$). δ_H (400 MHz, $CDCl_3$) 5.35 (2H, m, 6-H and 7-H), 3.73-3.68 (3H, m, 9-H and 1-H_2), 3.63 (1H, dt, *J* 10.0, 7.5, 3-H), 2.13 (1H, qd, *J* 6.5, 4.0, 8-H), 2.02 (1H, ddd, *J* 11.0, 9.0, 5.5, 5-*H*H), 1.79 (1H, m, 5-*H*H), 1.63 (1H, m, 4-H), 1.58 (2H, m, 2-H_2), 1.03 (3H, d, *J* 6.5, 10-H_3), 0.96 (3H, d, *J* 6.5, 8-CH_3), 0.90 (9H, s, $SiC(CH_3)_3$, 0.89 (18H, s, $SiC(CH_3)_3$ ×2), 0.86 (3H, d, *J* 6.5, 4-CH_3), 0.05 (6H, s, $SiCH_3$ ×2), 0.04 (12H, s, $SiCH_3$ ×4). δ_C (101 MHz, $CDCl_3$) 134.0 (C-7), 129.1 (C-6), 72.2 (C-3), 72.1 (C-9), 60.7 (C-1), 44.4 (C-8), 39.6 (C-4), 36.4 (C-5), 36.2 (C-2), 26.2 ($SiC(\underline{C}H_3)_3$), 26.1 ($SiC(\underline{C}H_3)_3$), 26.1 ($SiC(\underline{C}H_3)_3$), 20.6 (C-10), 18.5 ($Si\underline{C}(CH_3)_3$), 18.3 ($Si\underline{C}(CH_3)_3$), 18.3 ($Si\underline{C}(CH_3)_3$), 16.1 (8-CH_3), 14.4 (4-CH_3), –4.2 ($SiCH_3$), –4.2 ($SiCH_3$), –4.4 ($SiCH_3$), –4.6 ($SiCH_3$), –5.1 ($SiCH_3$), –5.1 ($SiCH_3$). HRMS calc. for $[C_{30}H_{66}O_3Si_3+Na]^+$ 580.4217, found 580.4211. ν_{max} (neat)/cm^{-1} 2955, 2928, 2856, 1471, 1462, 1252.

(3*S*,4*R*,8*R*,9*S*,*E*)-3,9-Bis(*tert*-butyldimethylsilyloxy)-4,8-dimethyldec-6-en-1-ol S17

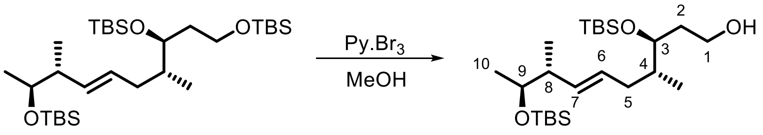

TBS ether **S16** (100 mg, 0.18 mmol) was dissolved in anhydrous MeOH (2 mL) and the solution cooled to 0 °C. Py.Br$_3$ (2.87 mg, 0.009 mmol) was added and the reaction mixture was stirred at 0 °C for 2.5 h. The reaction was quenched by the addition of sat. aq. NaHCO$_3$ (*ca.* 5 mL), the layers were separated and the aqueous layer extracted with EtOAc (3 × 10 mL). The combined organic extracts were dried over MgSO$_4$ and the solvent removed *in vacuo*. The crude residue was purified by column chromatography (5% EtOAc in petroleum ether) to give alcohol **S17** as a colourless oil (32 mg, 40%). $[\alpha]_D^{25}$= -7.0 (*c* 1.0, CHCl$_3$). δ_H (400 MHz, CDCl$_3$) 5.33 (2 H, m, 6-H and 7-H), 3.79 (1H, dt, *J* 8.5, 4.0, 3-H), 3.74 (2H, m, 1-H$_2$), 3.68 (1H, m, 9-H), 2.16 (1H, m, OH), 2.13 (1H, m, 8-H), 2.01 (1H, m, 5-*H*H), 1.79 (1H, ddd, *J* 13.5, 8.5, 5.5, 5-*H*H), 1.70 (1H, m, 4-H), 1.66 (H, m, 2-H$_2$), 1.03 (H, d, *J* 6.5, 10-H$_3$), 0.96 (3H, d, *J* 6.5, 8-CH$_3$), 0.90 (9H, s, SiC(CH$_3$), 0.88 (9H, s, SiC(CH$_3$), 0.86 (3H, d, *J* 6.5, 4-CH$_3$), 0.09 (3H, s, SiCH$_3$), 0.07 (3 H, s, SiCH$_3$), 0.03 (3H, s, SiCH$_3$), 0.03 (3H, s, SiCH$_3$). δ_C (101 MHz, CDCl$_3$) 133.1 (C-7), 128.5 (C-6), 74.7 (C-3), 72.1 (C-9), 61.1 (C-1), 44.4 (C-8), 39.1 (C-4), 36.9 (C-5), 33.2 (C-2), 26.0 (SiC($\underline{C}$H$_3$)$_3$ x 2), 20.7 (C-10), 18.3 (Si$\underline{C}$(CH$_3$)), 18.2 (Si$\underline{C}$(CH$_3$)), 16.2 (8-CH$_3$), 14.0 (4-CH$_3$), -4.2 (SiCH$_3$), –4.2 (SiCH$_3$), –4.4 (SiCH$_3$), –4.7 (SiCH$_3$). HRMS calc. for [C$_{24}$H$_{52}$O$_3$Si$_2$+Na]$^+$ 467.3358, found 467.3353. ν_{max} (neat)/cm^{-1} 3789, 2953, 2929, 2836, 1251.

(3*S*,4*R*,8*R*,9*S*,*E*)-3,9-Bis(*tert*-butyldimethylsilyloxy)-4,8-dimethyldec-6-enal S18

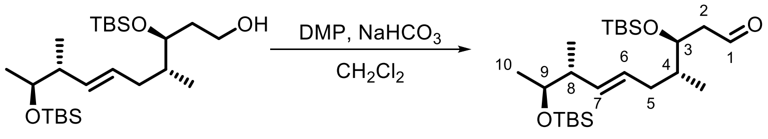

Alcohol **S17** (130 mg, 0.29 mmol) was dissolved in CH$_2$Cl$_2$ (10 mL), NaHCO$_3$ (121 mg, 1.45 mmol) was added and the reaction mixture was cooled to 0 °C. DMP (0.35 M solution in CH$_2$Cl$_2$, 1.08 mL, 0.38 mmol) was added dropwise and the reaction mixture stirred at room temperature for 1.5 h. The reaction was quenched by the addition of sat. aq. Na$_2$S$_2$O$_3$ (*ca.* 5 mL), the layers were separated and the aqueous layer extracted with CH$_2$Cl$_2$ (3 × 10 mL). The combined organic extracts were dried over MgSO$_4$ and the solvent removed *in vacuo*. The crude residue was triturated with Et$_2$O (*ca.* 2 mL), filtered and concentrated *in vacuo* to give aldehyde **S18** (125 mg, 98%) as a colourless oil which was used without further purification. $[\alpha]_D^{25}$= +2.0 (*c* 1.0, CHCl$_3$). δ_H (400 MHz, CDCl$_3$) 9.79 (1H, dd, *J* 3.0, 2.0, 1-H), 5.34 (2H, m, 6-H and 7-H), 4.15 (1H, dt, *J* 8.0, 4.0, 3-H), 3.68 (1H, qd, *J* 6.5, 4.0, 9-H),

2.50 (1 H, ddd, *J* 15.5, 8.0, 3.0, 2-*H*H), 2.38 (1H, ddd, *J* 15.5, 4.0, 2.0, 2-*H*H), 2.11 (1H, m, 8-H), 2.01 (1H, m, 5-*H*H), 1.82 (1H, m, 5-*H*H), 1.71 (1H, m, 4-H), 1.02 (3H, d, *J* 6.5, 10-H$_3$), 0.95 (3H, d, *J* 6.5, 8-CH$_3$), 0.87 (21H, m, 2 x Si(CH$_3$)$_3$ and 4-CH$_3$), 0.03 (12H, m, 4 × SiCH$_3$). δ_C (126 MHz, CDCl$_3$) 202.8 (C-1), 134.8 (C-7), 128.1 (C-6), 72.0 (C-9), 71.1 (C-3), 46.5 (C-2), 44.4 (C-8), 39.7 (C-4), 36.6 (C-5), 26.0 (C($\underline{C}$H$_3$)$_3$), 25.9 (C($\underline{C}$H$_3$)$_3$), 20.8 (C-10), 18.3 ($\underline{C}$(CH$_3$)$_3$), 18.2 ($\underline{C}$(CH$_3$)$_3$), 16.3 (8-CH$_3$), 14.2 (4-CH$_3$), –4.2 (SiCH$_3$), –4.3 (SiCH$_3$), –4.5 (SiCH$_3$), –4.7 (SiCH$_3$). HRMS calc. for [$C_{24}H_{50}O_3Si_2$+H]$^+$ 443.3371, found 443.3373. ν_{max} (neat)/cm^{-1} 2929, 2857, 1729, 1462, 1375, 1253.

(3*S*,4*R*,8*R*,9*S*,*E*)-3-(*tert*-Butyldimethylsilyloxy)-9-hydroxy-4,8-dimethyldec-6-enoic acid S19

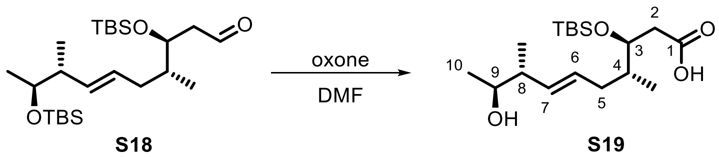

Aldehyde **S18** (30 mg, 0.07 mmol) was dissolved in anhydrous DMF (0.7 mL), oxone (20 mg, 0.07 mmol) was added and the reaction mixture was stirred at room temperature for 3 h. The reaction was quenched by the addition of HCl (1 M, *ca.* 1 mL), the layers were separated and the aqueous layer extracted with EtOAc (3 × 1 mL). The combined organic extracts were washed with water (3 × 5 mL), dried over MgSO$_4$ and concentrated *in vacuo*. The crude residue was purified by column chromatography (20-100% EtOAc in petroleum ether) to give acid **S19** (12.5 mg, 52%) as a colourless oil. $[\alpha]_D^{25}$= +4.0 (*c* 1.0, CHCl$_3$). δ_H (500 MHz, CDCl$_3$) 5.48 (1H, dt, *J* 14.5, 7.0, 6H), 5.38 (1H, ddt, *J* 15.5, 8.5, 1.5, 7-H), 4.09 (1H, m, 3-H), 3.53 (1H, p, *J* 6.0, 9-H), 2.43 (2H, d, *J* 6.0, 2-H$_2$), 2.08 (2H, m, 8-H and 5-*H*H), 1.88 (1H, m, 5-H*H*), 1.73 (1H, m, 4-H), 1.16 (3H, d, *J* 6.5, 10-H$_3$), 1.00 (3H, d, *J* 6.5, 8-CH$_3$), 0.88 (3H, d, *J* 6.5, 4-CH$_3$), 0.87 (9H, s, SiC(CH$_3$)$_3$, 0.07 (3H, s, SiCH$_3$), 0.06 (3H, s, SiCH$_3$). δ_C (126 MHz, CDCl$_3$) 176.4 (C-1), 133.9 (C-7), 130.4 (C-6), 72.2 (C-3), 71.2 (C-9), 44.9 (C-8), 39.0 (C-4), 37.6 (C-2), 36.2 (C-5), 25.8 (SiC($\underline{C}$H$_3$)$_3$), 20.2 (C-10), 18.0 (Si$\underline{C}$(CH$_3$)$_3$), 16.6 (8-CH$_3$), 14.3 (4-CH$_3$), –4.6 (SiCH$_3$), –4.8 (SiCH$_3$). HRMS calc. for [$C_{18}H_{35}O_4Si$-H]$^-$ 343.2305, found 343.2313. ν_{max} (neat)/cm^{-1} 3410, 2959, 2929, 2866, 1712, 1462, 1378, 1252, 1080.

***S*-((3*S*,4*R*,8*R*,9*S*,*E*)-3,9-dihydroxy-1-oxo-4,8-dimethyldec-6-ene-1-yl)-pantetheine 12**

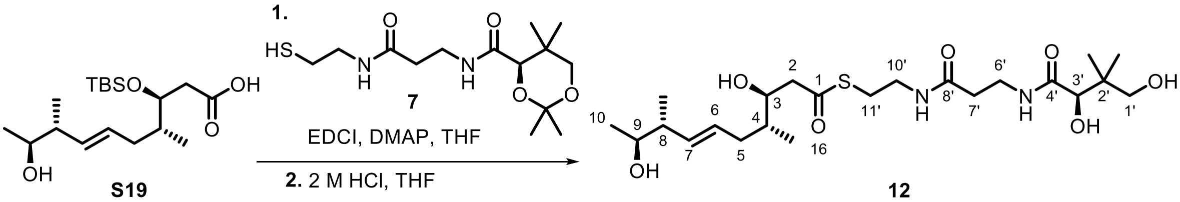

Acid **S19** (9.5 mg, 0.03 mmol) was dissolved in CH$_2$Cl$_2$ (0.2 mL). Protected pantetheine **7** (29 mg, 0.09 mmol) and DMAP (0.4 mg, 0.003 mmol) were added. The reaction mixture was cooled to 0 °C and EDCI (7.2 mg, 0.038 mmol) was added. The reaction was stirred at 0 °C for 5 mins and then

allowed to warm to room temperature and stirred for 16 h. The reaction was quenched by the addition of HCl (1 M, 1 mL), the layers were separated and the aqueousf layer was extracted with EtOAc (3 ×1 mL). The combined organic extracts dried over $MgSO_4$ and the solvent removed *in vacuo* to give a pale yellow oil (16.5 mg, 0.03 mmol) which was dissolved in THF (0.5 mL). HCl (2 M, 0.25 mL) was added and the reaction mixture was stirred at room temperature for 9 h. The reaction was quenched by the addition of sat. aq. $NaHCO_3$ (*ca*. 1 mL), the layers were separated and the aqueous layer extracted with EtOAc (3 × 5 mL). The combined organic layers were dried over $MgSO_4$ and concentrated *in vacuo*. The crude residue was purified by column chromatography (5-15% MeOH in CH_2Cl_2) to give **12** as a colourless oil (14.7 mg, 98% over two steps). $[\alpha]_D^{25}$= +4.0 (*c* 0.5, CD_3OD). δ_H (500 MHz, CD_3OD) 5.46 (2H, m, 6-H and 7-H), 3.97 (1H, ddd, *J* 9.0, 6.0, 3.0, 3-H), 3.92 (1 H, s, 3′-H), 3.63 (1H, qd, *J* 6.0, 5.0, 9-H), 3.47 (2 H, m, 6′-H_2), 3.47 (1 H, m, 1′-*H*H), 3.40 (1H, m, 9′-H*H*), 3.36 (2H, m, 10′-H_2), 3.03 (2H, t, *J* 6.5, 11′-H_2), 2.74 (1H, dd, *J* 15.0, 3.5, 2-*H*H), 2.69 (1H, m, 2-H*H*), 2.42 (2H, t, *J* 6.5, 7′-H_2), 2.21 (1 H, m, 5-*H*H), 2.16 (1H, m, 8-H), 1.91 (1H, m, 5-*H*H), 1.64 (1H, m, 4-H), 1.12 (3H, d, *J* 6.5,10-H_3), 1.02 (3H, d, *J* 6.5, 8-CH_3), 0.93 (6H, s, 2 × 2′-CH_3), 0.90 (3H, d, *J* 6.5, 4-CH_3). δ_C (126 MHz, CD_3OD) 199.4 (C-1), 176.1 (C-4′), 174.0 (C-8′), 135.3 (C-7), 130.1 (C-6), 77.3 (C-4′), 73.0 (C-3), 72.2 (C-9), 70.4 (C-1′), 49.1 (C-2), 45.3 (C-8), 40.4 (C-4), 40.0 (C-10′), 36.7 (C-5), 36.4 (C-7′), 36.3 (C-6′), 29.3 (C-11′), 21.3 (C-2′), 20.9 (2′-CH_3 × 2), 20.2 (C-10), 16.6 (8-CH_3), 15.6 (4-CH_3). HRMS calc. for $[C_{23}H_{42}N_2O_7S+Na]^+$ 513.2570, found 513.2572. ν_{max} (neat)/cm^{-1} 3676, 3344, 2972, 2920, 2904, 1708, 1655, 1578, 1451, 1404, 1252.

9. Materials and methods for molecular biology techniques.

Reagents were purchased from Sigma-Aldrich, Thermo Fisher or Merck Millipore. *E. coli* competent cells were purchased from New England Biolabs (T7 Express and 5-α) or Merck Millipore (Novagen BL21 (DE3)). All enzymes used were purchased from Thermo Fisher Scientific.

10. Plasmid generation, protein expression and purification

The gene encoding MupA was amplified from *P. fluorescens* NCIMB 10856 genomic DNA with MupA_FOR:

5′-AAGTTCTGTTTCAGGGCCCGATGTCAGTTGAACAATTACTCGGCCTGGGTGTG-3’

and MupA_REV:

5′- ATGGTCTAGAAAGCTTTACTGTGCTGACCGCTCCTGGAACGAG-3’

primers and subcloned into the pOPINF vector (pOPINF-MupA), bearing an N-terminal His_6 tag and a 3C protease cleavage site (LEVLFQGP). The nucleotide sequence for ACP_D4, ACP_A1 and Fre were synthesized and sub-cloned into a pET151-D/TOPO plasmid bearing an N-terminal His_6 tag and a tobacco etch virus (TEV) cleavage site (ENLYFQ) by Thermo Fisher. Both MupN and CoaA/CoaD/CoaE were expressed and purified as described previously [12].

Individual plasmids were transformed into *E. coli* T7 Express cells. Cultures were grown to OD_{600} = 0.7 in LB media (37 °C) supplemented with carbenicillin (100 μg/mL) and induced (0.5 mM isopropyl β-D-1-thiogalactopyranoside) at 16 °C (16 h) before cell pellets were harvested by centrifugation (6000 rpm, 10 mins) and resuspended in buffer A (50 mM Tris-HCl, 500 mM NaCl, 10% (v/v) glycerol, pH 8.0). Harvested cells were sonicated and the soluble fraction was purified by immobilized metal affinity chromatography (IMAC) via a HiTrap 5 ml HP Ni column (GE Healthcare). Protein was eluted using a linear gradient from 6-100% of Buffer B (50 mM Tris-HCl, 500 mM NaCl, 10% (v/v) glycerol, 800 mM imidazole, pH 8.0). Eluted protein was further purified by size exclusion chromatography (SEC) using either a HiPrep 26/60 Sephacryl S100 or S200 column (GE Healthcare) in Buffer C (25 mM Tris-HCl, 150 mM NaCl, pH 7.5, 1 mM DTT) before protein concentration. Proteins were either immediately used or stored at -20 °C. Both ACP_D4 and ACP_A1 were cleaved overnight using in house TEV protease prior to SEC. Purified protein (50 μM) was analysed by analytical size exclusion chromatography using either a Superdex 75 10/300 or Superdex 200 increase 10/300 GL column (GE Healthcare) calibrated with molecular weight standards (GE Healthcare) [13].

For NMR studies ^{15}N labelled protein was produced from cells grown to OD_{600} = 2.0 in LB media supplemented with carbenicillin (100 μg/ml) at 37°C. Cells were then pelleted by centrifugation (6000 rpm, 10 mins), washed twice with sterile M9 media and then exchanged into M9 minimal media at a 4:1 volumetric ratio. Cells were supplemented with 1 gL^{-1} $^{15}NH_4Cl$, 0.5% (v/v) glycerol and 0.05% (w/v) glucose and induced with 0.5 mM IPTG, then harvested after 16 hrs at 16 °C. Cells were resuspended into buffer A and purified as described above for the unlabelled protein.

11 Whole cell biotransformation with MupA

For *in vivo* biotransformations 50 ml of overnight culture of *E. coli* (T7 express) cells were harvested and resuspended in 2 ml 0.1 M K_2HPO4 pH 7.2 buffer supplemented with 20 mM glucose and 0.5 mg of 7-keto pantetheine **9** or 7-hydroxy pantetheine **12** dissolved in MeOH. Cells were incubated at 30 °C, 180 rpm for 6-16 hrs. Reactions were quenched by adding equal volume acetonitrile, then vortexed and centrifuged. The acetonitrile layer was injected for LC-MS analysis.

12 *In-vitro* LCMS assays

5 mM of 7-keto pantetheine substrate **(9)** dissolved in DMSO was incubated with 20 μM MupA, 20 μM Fre, 1 mM FMN and 10 mM NADH in Buffer C for 1 h. Proteins were then isolated by methanol chloroform precipitation and the resultant organic layer was injected for LC-MS analysis.

13 ESMS

Samples were desalted for ESMS analysis using a C_4 ZipTip (Merck) per the manufacturer's instructions. Denatured samples were analysed on a Synapt G2-Si (Waters) fitted with a TriVersa NanoMate (Advion) using the following parameters: sample cone, 10 V; capillary voltage 1.5 kV; trap collision energy, 10 V. The source as set to positive mode and spectra were acquired over 500-3000 m/z and analysed using MassLynx 4.1 software. For Ppant ejection assays, an appropriate charge state was isolated using the MSMS functionality. The transfer collision energy was increased until fragmentation was observed (typically 5 V to 20 V) and spectra were collected from 200-1000 m/z.

14 MupA ESMS assay

100 μM ACP_D4 and ACP_A1 were both upgraded with pantethine substrates: 7-keto pantetheine **9** or 7-hydroxyl-pantetheine **12** (100 μl reactions) as previously described [14] and desalted using a Zeba column (Thermo Fisher) equilibrated in buffer C. ^{13}C-7-keto pantetheine **9** was upgraded to ACP_D4 (100 μM) on a larger scale (1.5 ml) before desalting using a 5 ml HiTrap desalting column (GE Healthcare) and the upgraded ACP was further concentrated using VivaSpin concentrators (ThermoFisher).

Loaded ACP (100 μM), FMN (1 mM), Fre (20 μM) and NADH (5 mM) were mixed in Buffer C. Upon reduction of flavin, MupA was added (20 μM) and the reaction was incubated at room temperature in the absence of light for 0-1 h and analysed by MS (10 μl aliquot) at regular intervals. Ppant ejection was used to determine the outcome of the reaction.

15 NMR parameters

All protein NMR experiments were acquired on a Bruker *AVANCE*^{HD} 700 MHz spectrometer equipped with a 1.7 mm triple-resonance cryogenically cooled detection probe. For titrations of ACP_D4 and ACP_A1 with MupA or MmpA_KS^0, ^{1}H-^{15}N HSQC spectra were recorded at pH 8.0 and 298 K.

For ^{1}H-^{13}C SO-FAST HSQC assays, derivatised ACP samples (100-300 μM) were maintained at 288 K and experiments were carried out following the ESMS protocol. NMR data acquisition was completed

within 5-10 minutes after the addition of MupA. Higher (300 μM) derivatised ACP concentrations were found to offer no significant advantage in signal detection than using the lower concentration (100 μM). Both one-dimensional ^{1}H-NMR and ^{1}H-^{13}C SO-FAST HSQC spectra were acquired. Control spectra of 50 mM NADH, 50 mM FMN, 25 mM **9** and 10 mM ^{13}C **9** were also obtained. All spectra were acquired in 10% D_20 and were referenced using 4,4-dimethyl-4-silapentane-1-sulfonic acid (DSS).

Scheme S1. A) General mechanism for oxygen activation in flavin-dependent monooxygenases using flavin (R = FAD/FMN). The reaction of reduced flavin with oxygen generates the flavin C4a-(hydro) peroxide that reacts with electrophilic or nucleophilic substrates to form the flavin C4a-hydroxide. Release of water regenerates the oxidised FMN. B) Proposed mechanism for type I BVMO oxidations. The peroxyflavin attacks the substrate carbonyl via a nucleophilic mechanism. Within the active site, the arginine is responsible for activation of the substrate ketone, supported by the 2′OH from the ribose of the NADP$^+$. If this ketone is substituted for an alcohol, nucleophilic attack can no longer proceed. The proposed scheme is adapted from Scheme 3 of [15].

A

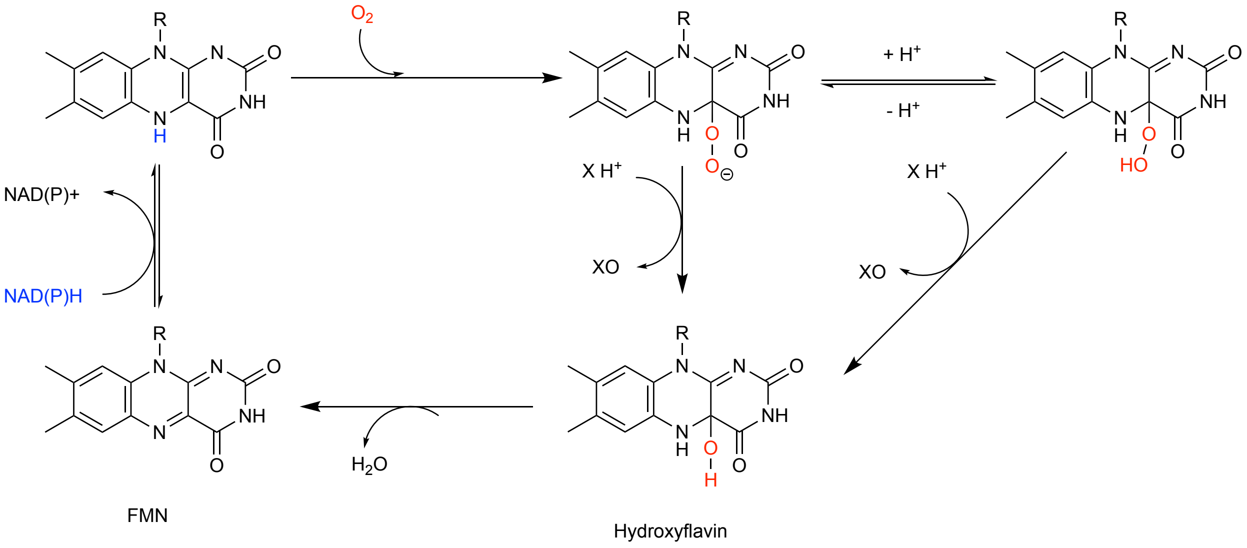

B

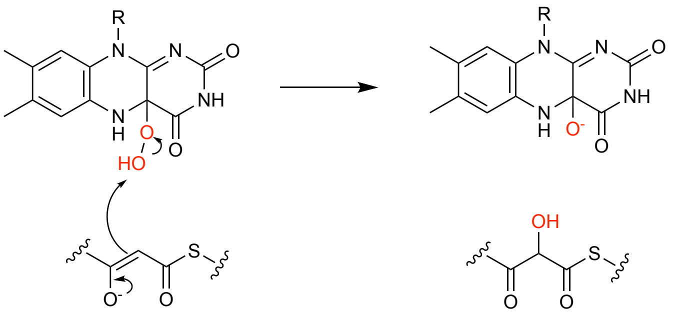

Figure S1. **Multiple sequence alignment of structurally characterised Class C monoxygenases**. A) *P. putida* CamE36 (uniprot: D7UER1), *V. harveryi* Luciferase LuxA subunit (uniprot: Q9S4D7) and *E. coli* SsuD (uniprot: P80645) with *P. fluorescens* MupA. The secondary structure elements of LuxA are included. (Inset) sequence identity between Class C monooxygenases and MupA (%). B) Three-dimensional X-ray crystallographic structures of several Class C monooxygenases, all of which display a TIM barrel fold (α/β)$_8$ with their PDB codes highlighted: CamE36 (light blue), SsuD (light green), Luciferase LuxA subunit (orange), LadA (light pink), MsuD (light yellow), the latter two are regarded as belonging to the SsuD sub-family of class C monooxygenases. An *ab initio* homology model of MupA (red) is included for structural comparison [16]. For clarity, only a single monomeric subunit is shown for each structure. Structures that have been co-crystallised with FMN are shown with sticks. Figure made using T-Coffee [17] and ESPRIPT [18].

A

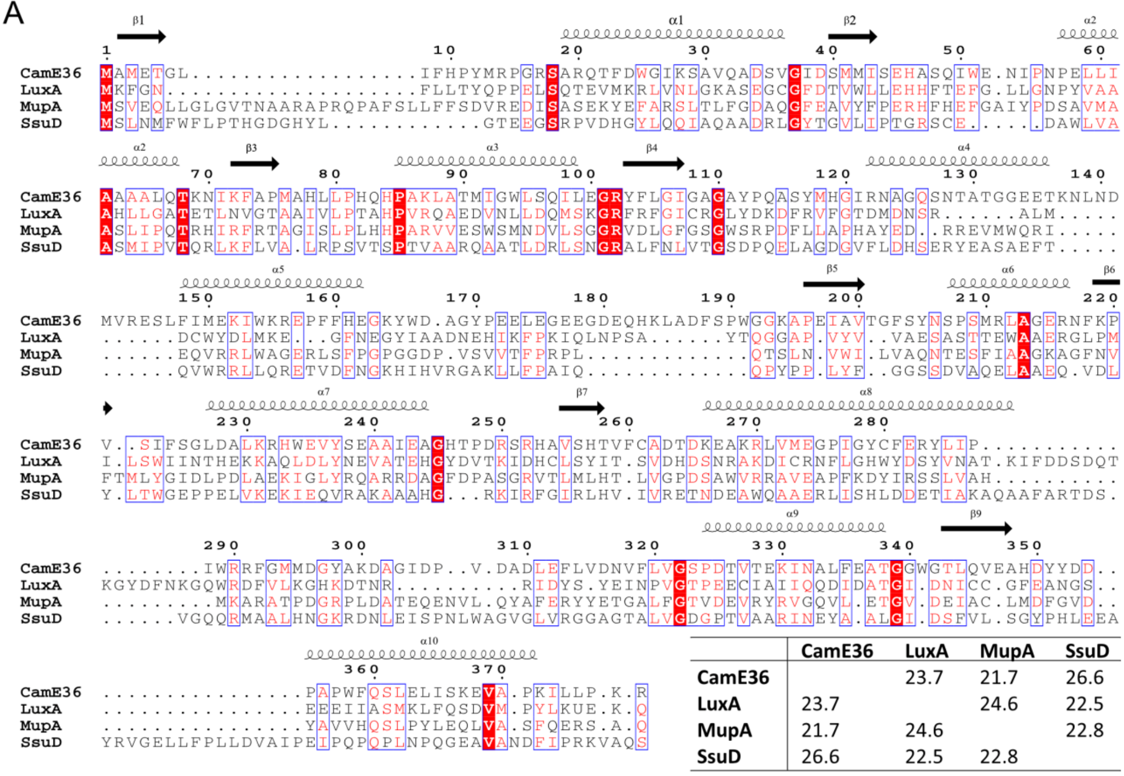

	CamE36	LuxA	MupA	SsuD
**CamE36**		23.7	21.7	26.6
**LuxA**	23.7		24.6	22.5
**MupA**	21.7	24.6		22.8
**SsuD**	26.6	22.5	22.8	

B

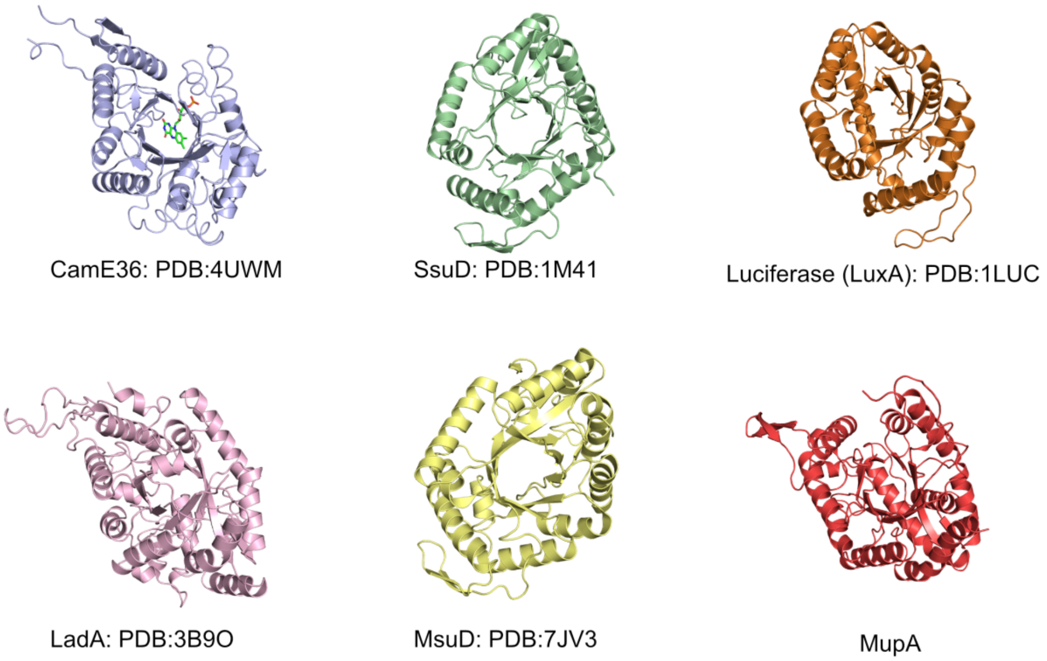

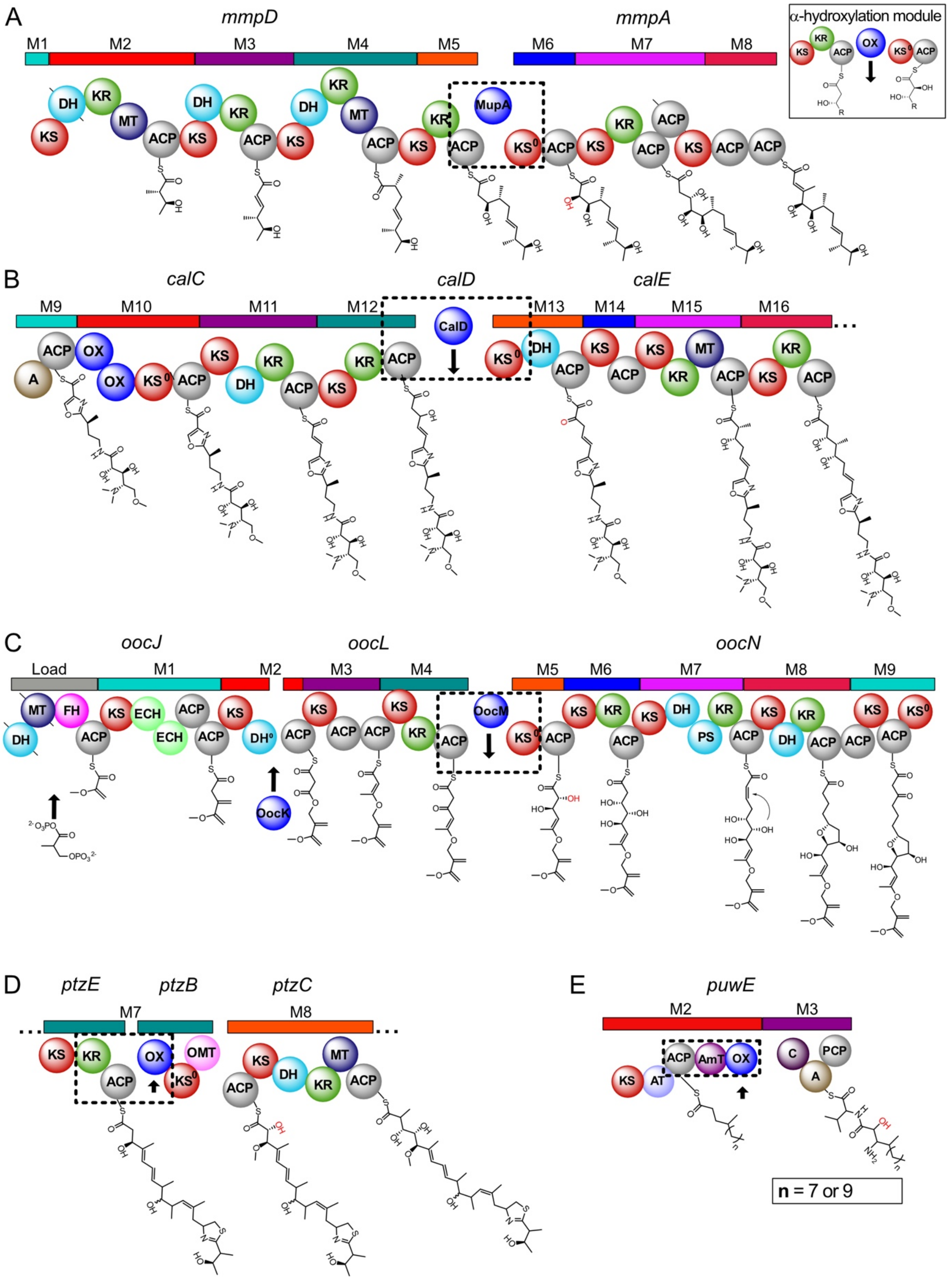

Figure S2. **Biosynthetic pathways that contain the monooxygenase domain and the proposed α-hydroxylation module architecture**. A) Proposed timing of action of the putative monooxygenase MupA within the mupirocin biosynthetic

pathway. MupA is proposed to act in between two type 1 modules (*mmpD* and *mmpA* prior to a KS^0 domain), on either a hydroxy or keto substrate. B) Proposed timing of action of the putative monooxygenase CalD within the calyculin A biosynthetic pathway. CalD is homologous to MupA and proposed to act between two type 1 modules (*calC* and *calE*) acting on a hydroxyl substrate. C) The timing of action of the monooxygenase OocM within the oocydin A biosynthetic pathway. OocM acts between two modules (OocL and OocN) to install an α-hydroxyl group at C14 on a β-keto substrate and constitutes a functioning α–hydroxylation module. D) The proposed timing of the *cis*-encoded monoxgenase PtzB_OX within the type 1 module *ptzB* within the Patellazole biosynthetic pathway. The monooxygenase (OX) acts in *cis* preceding a KS^0 domain rather than in *trans* as predicted for mupirocin and calyculin A and shown for the oocydin A biosynthetic pathways, reflecting an unusual α-hydroxylation module. E) Proposed timing of the cis-encoded monooxygenase (OX) encoded within the type 1 module *puwE* in the hybrid NRPS/PKS biosynthetic pathway of Puwainaphycin A. Here the PuwE_OX domain acts in *cis*, where it hydroxylates the growing acyl chain following the PKS module before the subsequent NRPS modules extend the chain to form the puwainphycins [19]. The fatty acid chain (n) is either a 7 or 9 carbon chain, with the R position referring to the Gln or Asn side chain. Inset: α-hydroxylation module whereby a *trans* acting monooxygenase acts inbetween two type I PKS modules. Within panels B, D and E only a snapshot of the type 1 PKS modules CalE, PtzE and PtzC are depicted. Domains with a strikethrough are inactive domains. A:Adenylation domain, ACP: Acyl carrier protein, AmT: Aminotransferase domain, C: Condensation domain, DH: Dehydratase domain, DH^0: DH-like domain, ECH: Enoyl CoA dehydratase, FH: FhkB like domain, KR: Ketoreductase, KS: Ketosynthase, KS^0: Ketosynthase domain incapable of chain extension, MT: Methyltransferase, OMT: O-methyltransferase domain, OX: monooxygenase domain, P: Peptide carrier protein, PS: Pyran synthase.

Figure S3. **Multiple sequence alignment of MupA (uniprot: Q9AH80) with Flavin dependent monooxygenases**. A) Sequences from *Candidatus Entotheonella* sp. (CalD, accession no. AB933566.1), *Serratia plymuthica* (OocM, AFX60335.1), *Theonella swinhoei* symbiont bacterium (OnnC, AY688304.2), *Candidatus Endolissoclinum faulkneri* strain L5 (PtzB_OX, K7ZDM8) and *Cylindrospermum alatosporum* CCALA 988 (PuwE_OX, A0A0A0WDC6). (Inset): MupA displays high sequence identity (%) to all these putative TIM $(\alpha/\beta)_8$ barrel containing monooxygenases. Lower sequence identity is observed with Flavin dependent monooxygenases from *Paederus fuscipes* symbiont bacterium (PedG: 17.7% identity, AAS547561.1) and *Serratia marcescens* strain MSMU97 (OocK: 19.0% identity, AFX60310.1) which clade together as FAD-containing monooxygenases.[20] B) Structural comparison of MupA with monoxygenase domains from related PKS systems. *Ab initio* homology models of MupA (red), CalD (blue), OocM (orange), OnnC (green), PtzB_OX (yellow), PedG (pink) and three-dimensional X-ray crystal structures of PuwE_OX (purple, PDB:6KET), and the type 1 BVMO Cyclohexanone monooxygenase (CHMO) (light green, PDB: 3UCL). Structural conservation between MupA and other monoxygenases is highlighted in parenthesis (RMSD). Both PedG and CHMO contain Rossman folds predicted to bind FAD and NAD(P)H.

A

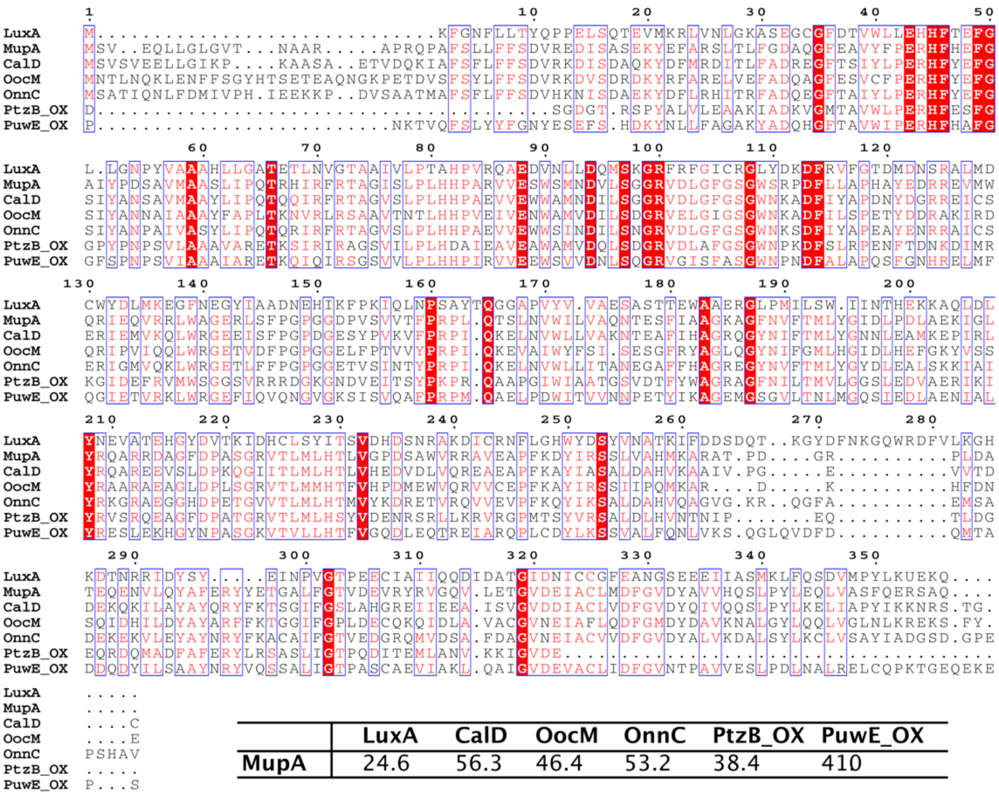

	LuxA	CalD	OocM	OnnC	PtzB_OX	PuwE_OX
**MupA**	24.6	56.3	46.4	53.2	38.4	410

B

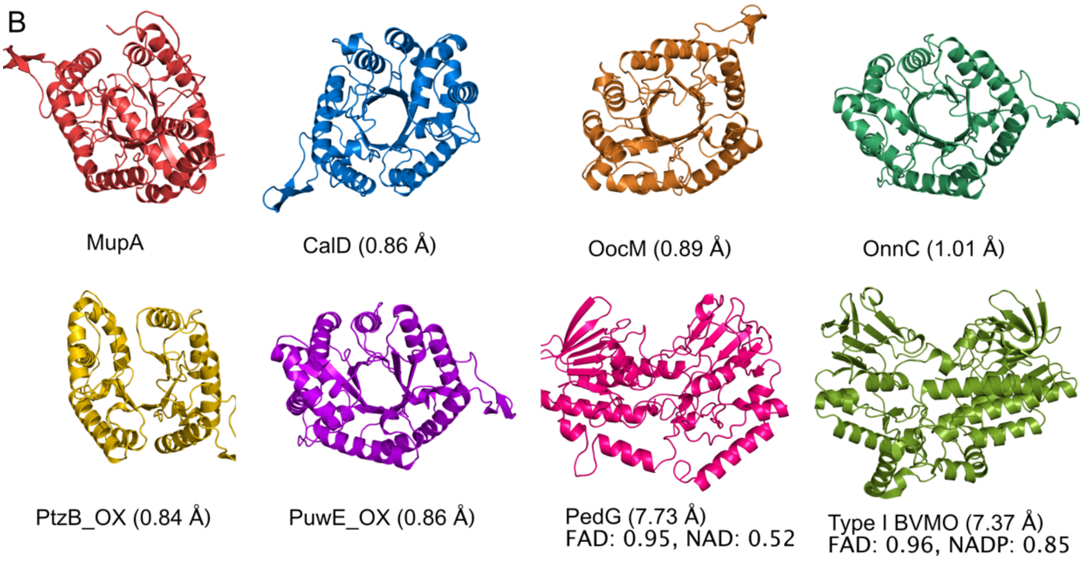

Figure S4. Sequence alignment of identified homologs of MupA and NAD(P)H: flavin dependent oxidoreductases encoded within *P. fluorescens* by protein-BLAST [21]. A) Multiple sequence alignment of MupA with LLM class flavin dependent oxidoreductases. The accession number of each homolog is used as their sequence name: WP_134924869 (91.60 %), WP_080889042 (35.00 %), WP_150675473 (27.59 %), WP_115488688 (24.83 %) and WP_224794946 (24.59 %) with the sequence identity to MupA in parenthesis. B) Multiple sequence alignments of candidate NAD(P)H: flavin dependent oxidoreductases with the model NAD(P)H: flavin dependent oxidoreductase *E. coli* Fre. The accession number of each homolog is used as their sequence name: WP_012535170 (40.62 %), OXS23022 (28.15 %) and MBM7764721 (27.64 %) with the sequence identity to Fre in parenthesis.

A

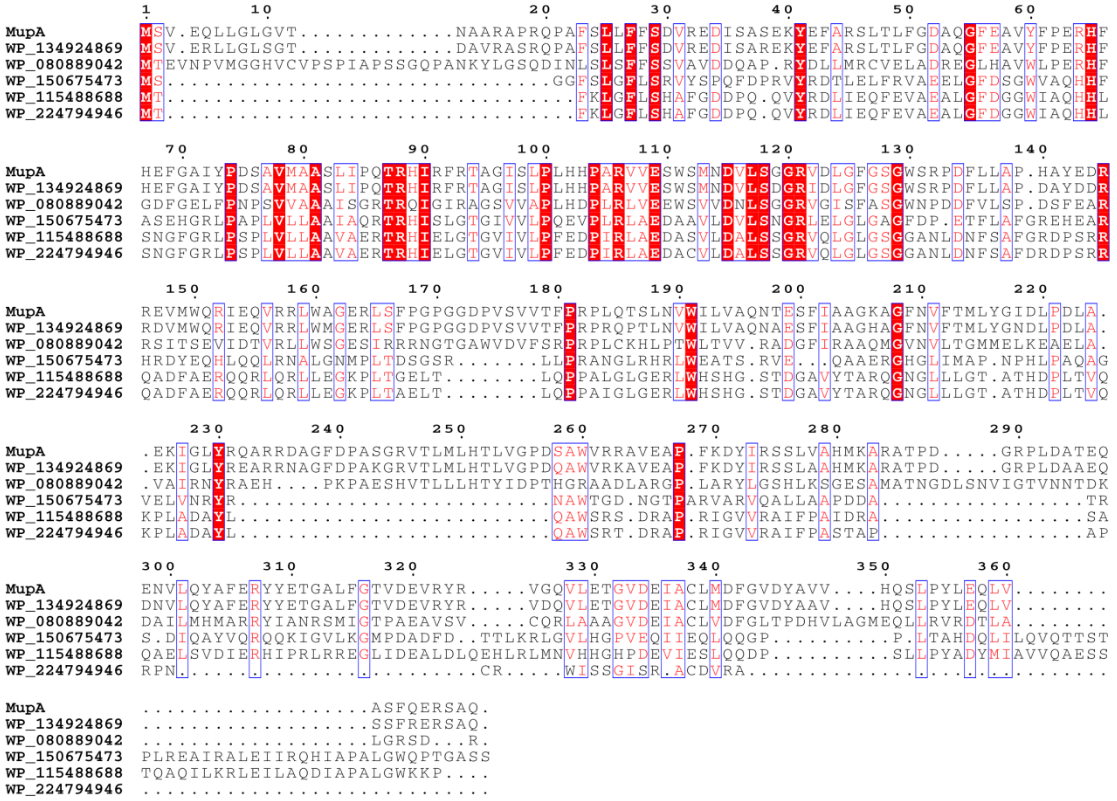

B

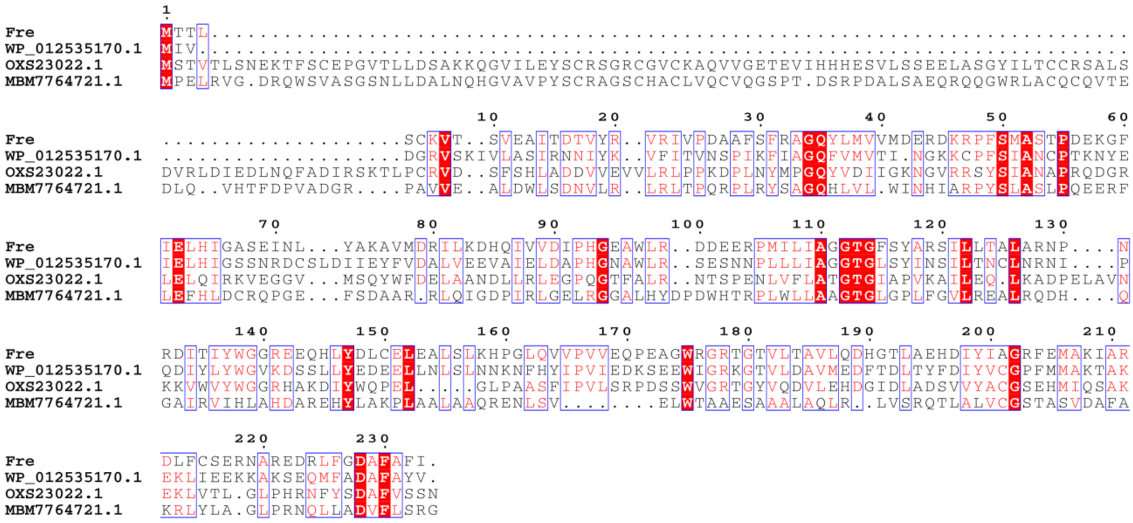

Figure S5*: In vivo* whole-cell biotransformations of 9 with MupA. LC-MS (total ion current; positive mode) traces of A) **9** (1 mg/ml), B) **9** (1 mg/ml) incubated with MupA- producing whole cells at 30 °C for 6 hr, C) **9** (1 mg/ml) incubated with MupA-producing whole cells at 30 °C for 16 hrs and D) MupA producing whole cells without **9** at 30 °C for 16 hrs. Mass highlighted at 10.1 min corresponds to **9**: Mass: 511.4 ($M+Na^+$). Hydrolysis corresponding to hydrolysis of the thioester (Mass: 229 Da) was observed at 9.02 min for B) and C).

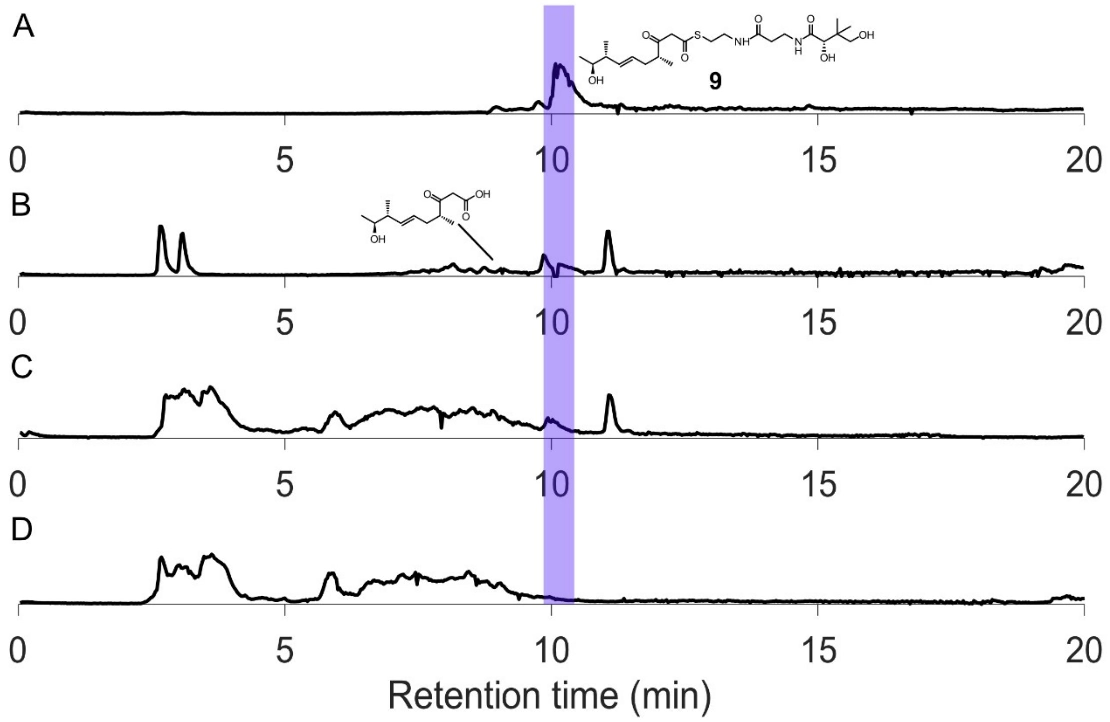

Figure S6. Protein purification and characterisation. A) **ACP_D4**. SDS-PAGE following the purification after sonication, IMAC before cleavage of the His_6 tag and SEC post cleavage. Analytical SEC showing ACP_D4.6His eluting as a monomer and non-native ESMS confirming the correct sequence of cleaved ACP_D4. B) **ACP_A1**. As A) with analytical SEC confirming a monomeric species and non-native ESMS confirming the correct sequence of cleaved ACP_A1. C) **MupA**. SDS-PAGE following the purification of MupA.6His after sonication to isolation via IMAC and SEC. Analytical SEC showing elution of MupA.6His as a dimer and non-native ESMS of denatured MupA.6His. D) **Fre**. SDS-PAGE following the purification of Fre.6His after sonication and isolation via IMAC and SEC. Analytical SEC showing elution of Fre.6His as a dimer and non-native ESMS of monomeric Fre.6His. E) ^{1}H NMR spectra of ACP_D4, ACP_A1, MupA and Fre. F) Calibration curve for the Analytical S75 and Analytical S200 column using the following calibrants: Aprotinin (AP), Ribonuclease A (RA), Carbonic Anhydrase (CA), Ovalbumin (OV), Conalbumin (CO), Cytochrome C (CC), Bovine Serum Albumin (BSA), Alcohol Dehydrogenase (ADH), β−Amylase (BA) and Thyroglobulin (TG).

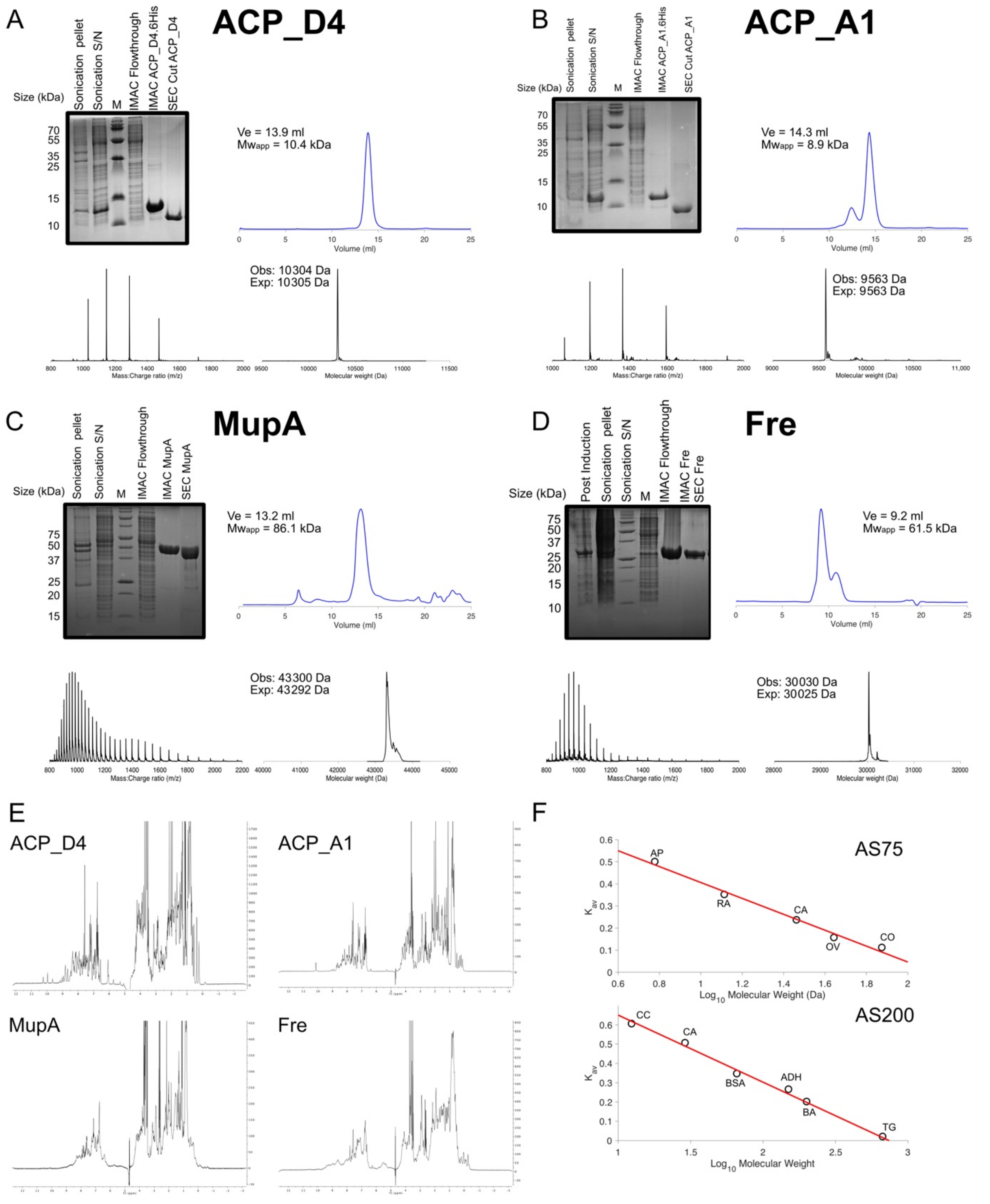

Figure S7. ***In vitro* bioassays with 9 (pantetheine substrate, no ACP).** LC-MS (total ion current; positive mode) traces of A) **9** (5 mM) and B) 5 mM **9** incubated with 20 µM MupA, 20 µM Fre, 1 mM FMN, 10 mM NADH incubated at 16 °C for 30 mins. The mass highlighted at 10 min corresponds to (**9**): Mass: 511.4 (M+Na$^+$).

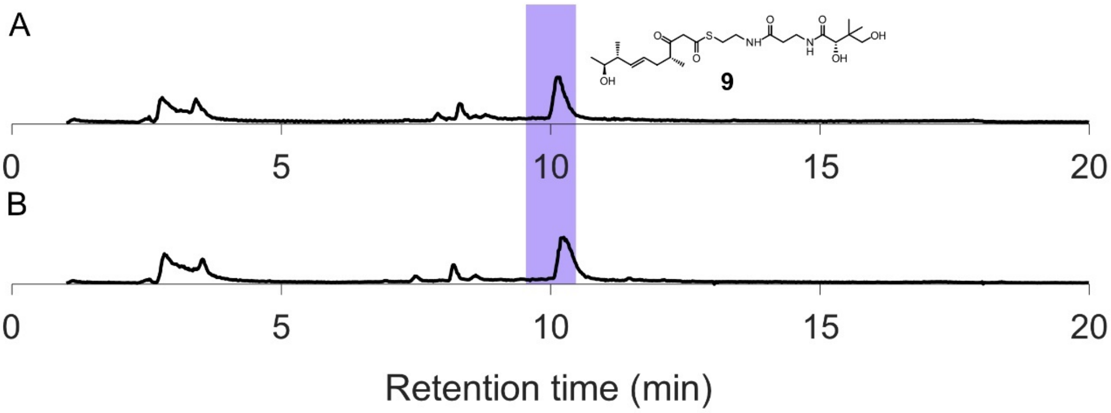

Figure S8. Control reactions of the hydroxylation reaction 9-ACP_D4 by MupA monitored by ESMS. A) Mass:Charge ratio (m/z) spectra of the hydroxylation reaction **9**-ACP_D4 with denatured MupA. (right): deconvoluted spectra of ACP_D4 after incubation with denatured MupA (*holo* ACP_D4: observed mass: 10,645 Da, expected mass: 10,646 Da, **9**-ACP_D4: observed mass:10,856 Da, expected mass: 10,855 Da). B) m/z spectra of the hydroxylation reaction of **9**-ACP_D4 when FMN, NADH and Fre were omitted. (right): deconvoluted spectra of ACP_D4 (**9**-ACP_D4: observed mass: 10,855 Da). C) m/z spectra of the hydroxylation reaction of **9**-ACP_D4 when NADH and Fre were omitted. (right): deconvoluted spectra of ACP_D4 (observed mass: 10,855 Da). D) m/z spectra of the hydroxylation reaction, where **9**-ACP_D4 was substituted for *holo* ACP_D4. (right): deconvoluted spectra of ACP_D4 (*holo* ACP_D4: observed mass: 10,645 Da). As with the native assay, a by-product at 10,732 Da was also observed corresponding to a modified *holo* ACP_D4 (+87 Da, 10,731 Da). Species corresponding to *holo*, **9**-ACP_D4 and a by-product are highlighted in pink, blue and grey respectively.

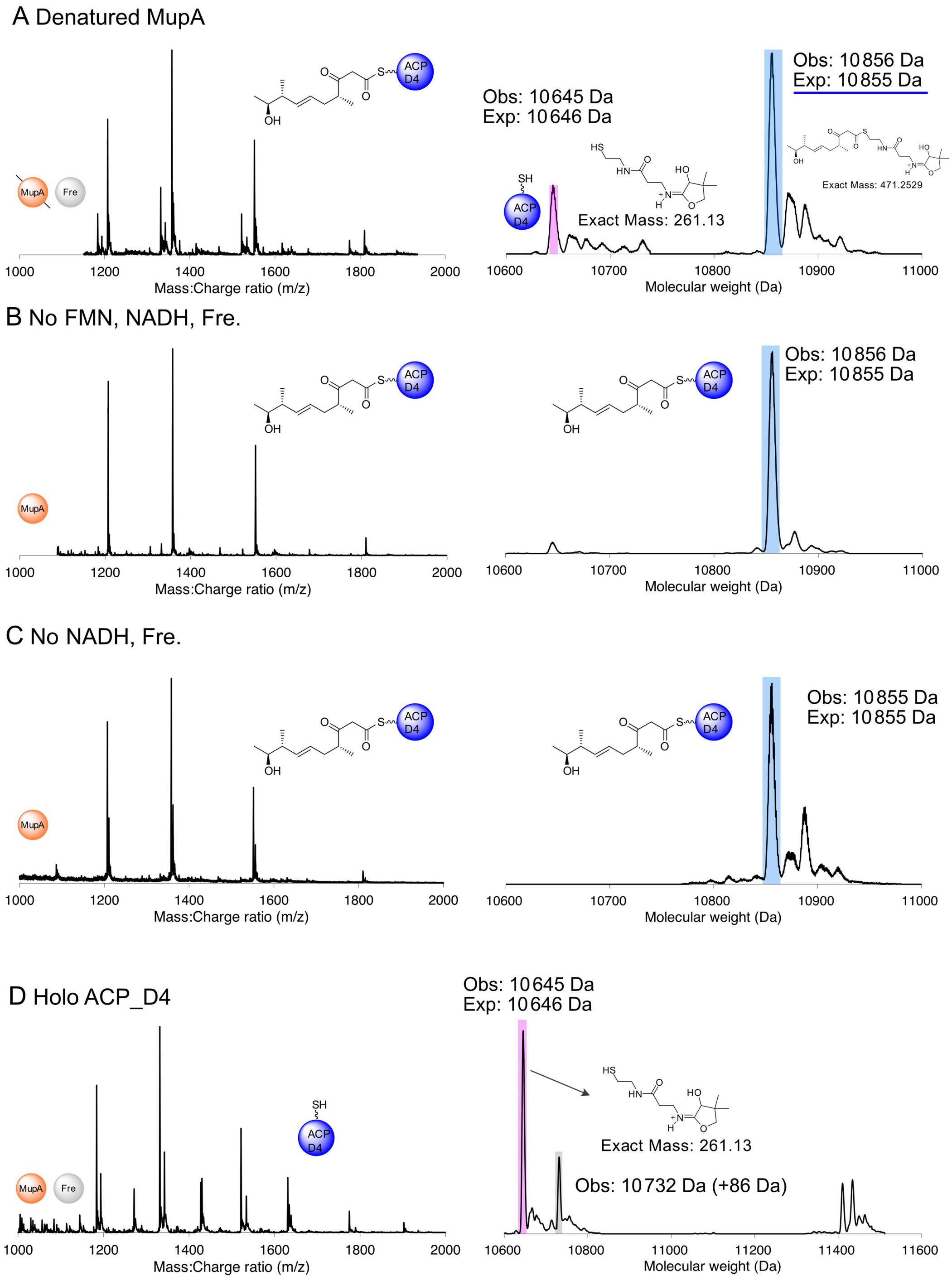

Figure S9. **Attempted hydroxylation of 9-ACP_A1**. A) Proposed reaction scheme for the hydroxylation of **9** attached to ACP_A1. B) ^{1}H-^{15}N HSQC spectra of apo ACP_A1 (blue) and in the presence of 3-fold excess of MupA (red). C) Deconvoluted ESMS spectrum for 9-ACP_A1(blue) with observed and expected mass indicated. (inset) Ppant ejection assay for this species. D) Deconvoluted ESMS spectrum for 9-ACP_A1 + MupA + Fre + FMN + NADH. *Holo* ACP_A1 (pink), **9**-ACP_A1 (blue) and a non-productive by-product (grey) are highlighted. The by-product appears to arise from modification of holo-ACP.

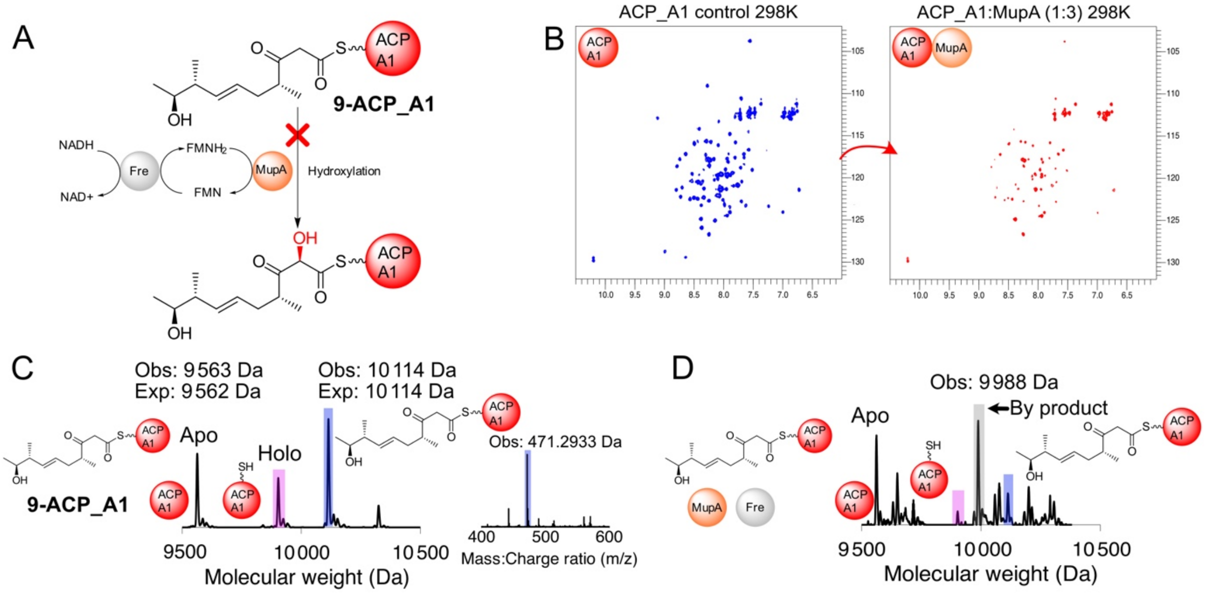

Figure S10. Reference ^{1}H-^{13}C spectra for cofactors and substrates. A) 50mM NAD(P)H with carbon atoms annotated. B) 50mM FMN C) 25mM **9** (no ^{13}C label) D) Mixture of NADH and ^{13}C labelled pantetheine derivative of the C-6 ^{13}C labelled β-keto substrate **9**. Hydrolysis product was visible after acquisition of this control spectrum. At 10mM, the minor enol species was also detectable at C-6 in D). This species was not visible at lower concentrations used in the NMR assays (Figure 4).

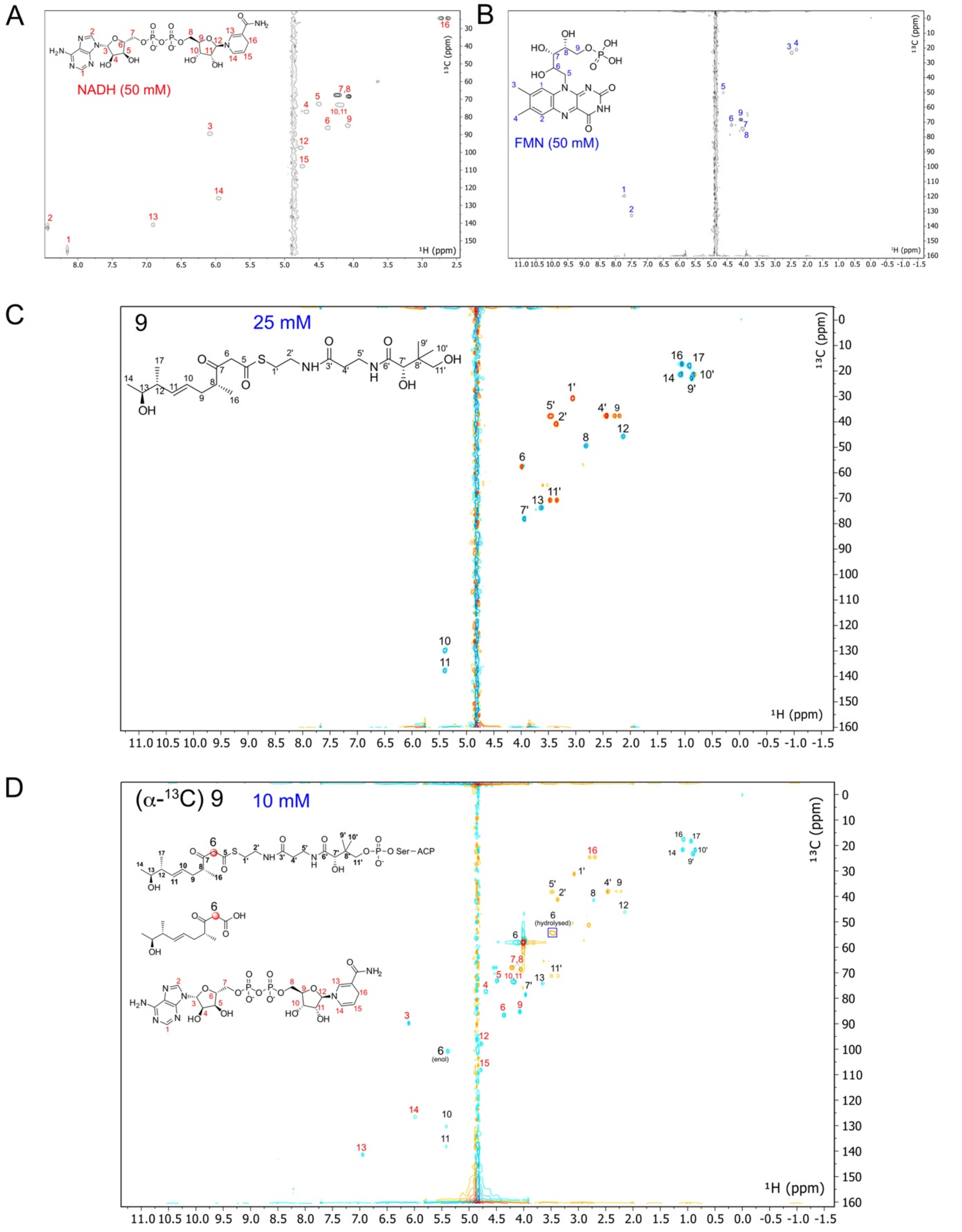

Figure S11. Purification and characterisation of MmpA_KS^0. A) SDS-PAGE following the purification of MmpA_KS^0.6His after sonication to isolation via IMAC and SEC. Analytical SEC showing elution of MmpA_KS^0.6His as a monomer and non-native ESMS of denatured MmpA_KS^0.6His.

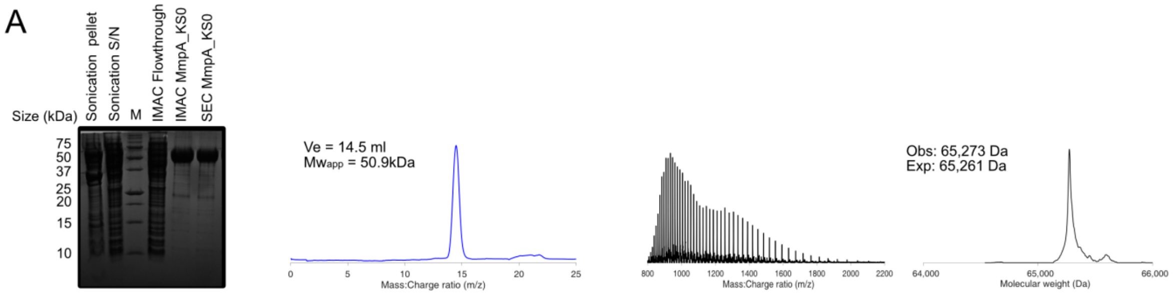

Figure S12: The oxidation state of C7 controls the timing of hydroxylation and ensures appropriate processing by downstream enzymes. A) Proposed mechanistic scheme for α-hydroxylation within the mupirocin biosynthetic pathway. Hydroxylation of a β-keto thioester attached to ACP_D4 occurs before ketoreduction by MmpD_KR6 enabling the correct downstream processing to produce PA-A. B) Ketoreduction of the β-keto thioester to a 7-OH group prevents hydroxylation by MupA resulting in metabolites lacking the 10,11-epoxide or THP ring and truncated fatty acid chains (mupirocin A5, A6 and A7, grey). C) If ketoreduction does not occur after hydroxylation of the β-keto thioester, no epoxidation or THP ring formation occurs with only 7-keto mupirocin W4 and 7-keto mupirocin W5 (grey) produced. D): The lack of hydroxylation and ketoreduction causes an accumulation of 7-keto analogues lacking the 10-11-epoxide, the THP ring and truncated fatty acid side-chains (mupirocin A1, A2, A3, grey). The oxidation state of C7 is highlighted in yellow and the α-hydroxyl moiety in red.

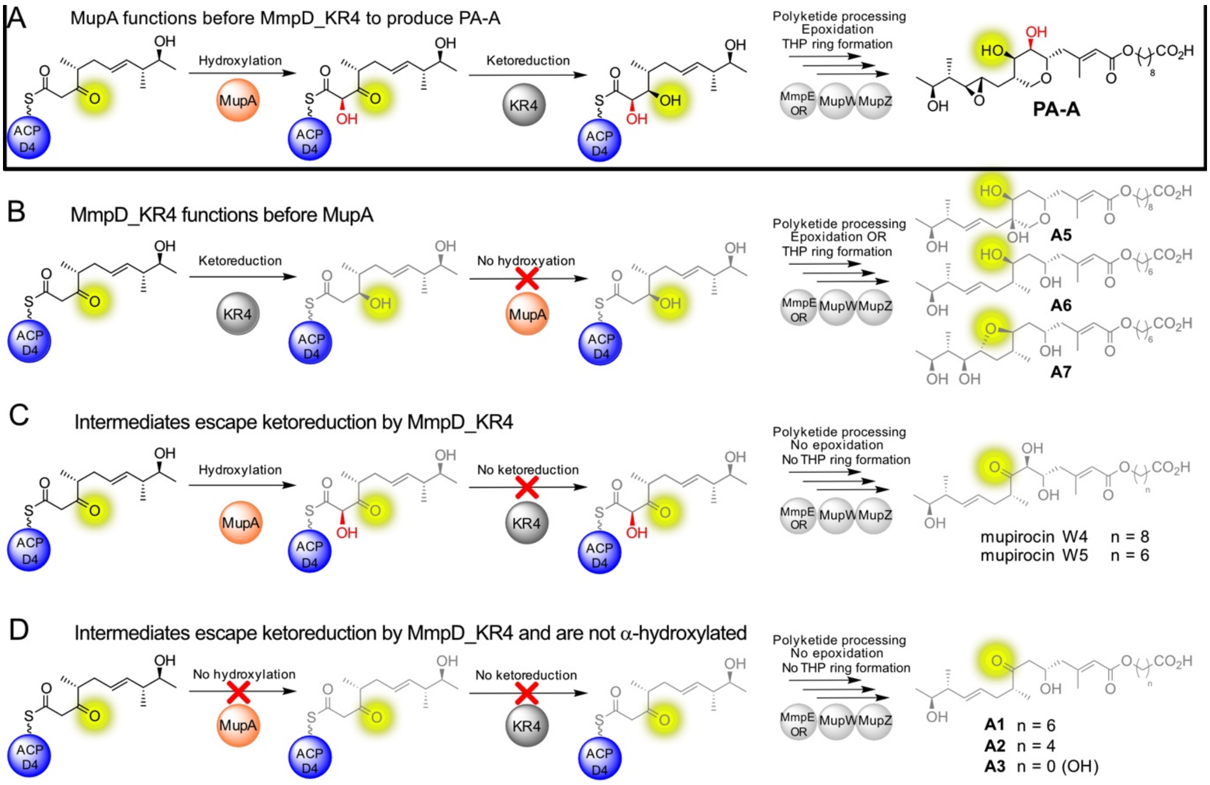

Figure S13. Sequence analysis of PKS termini within α-hydroxylation modules for putative docking domain regions. A) Analysis of the C-termini modules MmpD, CalC and OocL which contain an ACP domain, with comparison to the sequence of ACP Vir5b from the virginamycin *trans*-AT PKS which contains a C-terminal docking domain region (CDD) which is highlighted by a black bar. The stop site for each ACP is highlighted by a blue box, indicating the absence of an apparent CDD domain in MmpD, CalC and OocL. B) Analysis of the N-termini modules of MmpA, CalN and OocN which contain a KS domain, with comparison to the sequence of the VirFG KS domain which contains an N-terminal docking domain region (NDD), highlighted with a black bar. The conserved ketosynthase start site is highlighted with a black arrow.

A

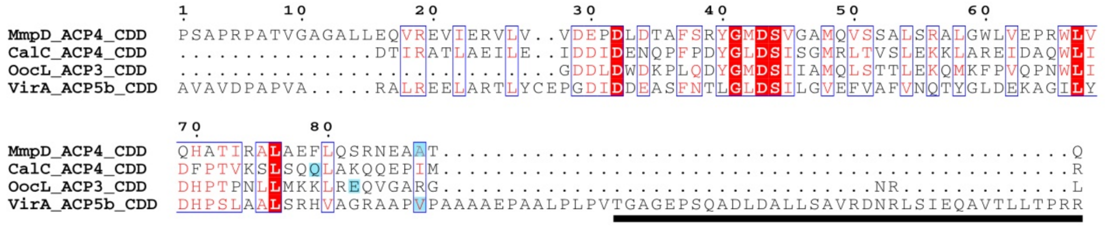

B

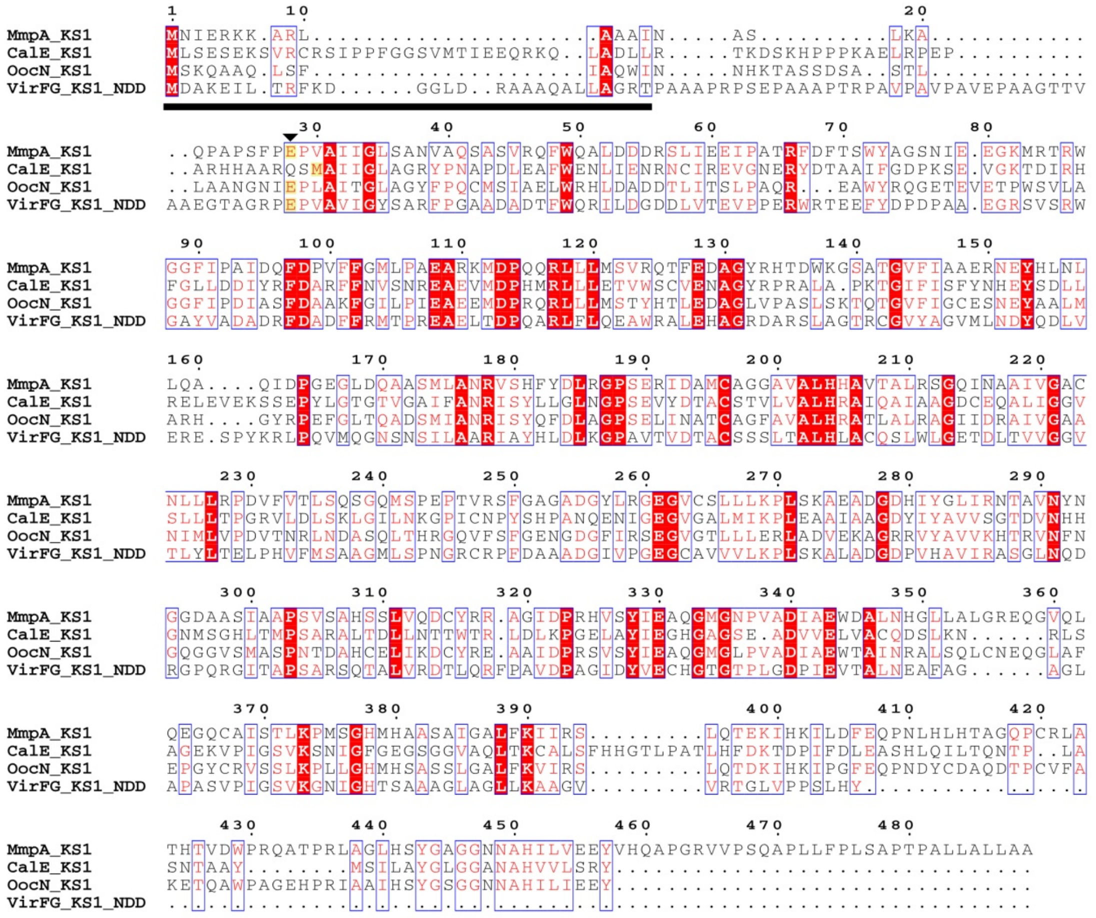

Figure S14. Bioinformatic analysis of the candidate docking domains within α-hydroxylation modules. A) MmpD/MmpA, B) CalC/CalE, C) OocL/OocN PKS interfaces and for comparison the characterised VirA/VirFG (D) sequences are included. Secondary structure prediction with PSIPRED [22] was carried out for each ACP_CDD sequence and the candidate NDD sequence identified from multiple sequence alignment. Intrinsic disorder/interaction propensity prediction using IUPred2A [23] was also carried out. For each prediction, the end site of the annotated ACP sequence is highlighted with a black arrow and candidate CDD sequences are highlighted in a black bar. The characterised CDD and NDD of VirA/VirFG is also highlighted with a black bar.

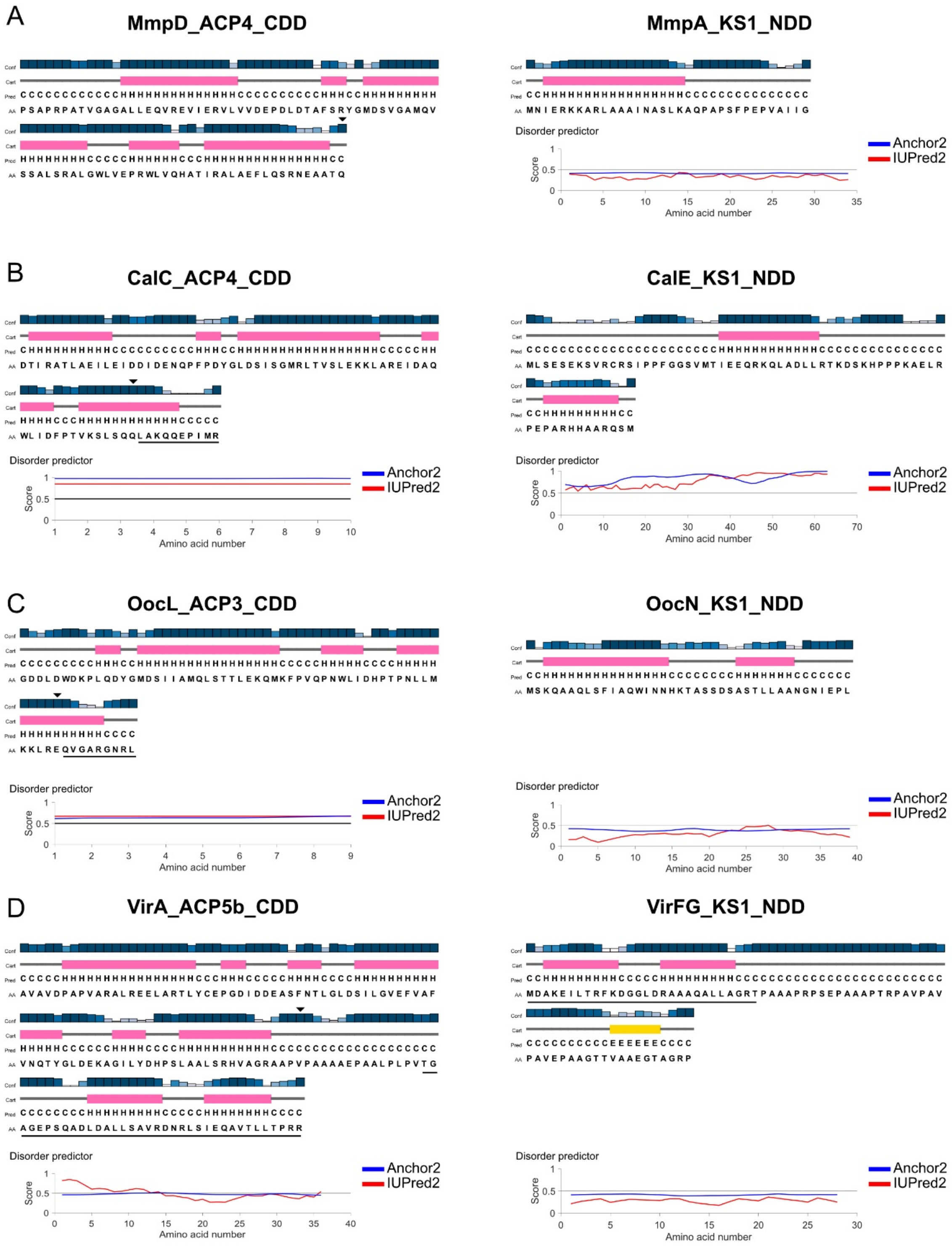

NMR data from general synthesis

Figure S15. ^{1}H (top) and ^{13}C (bottom) NMR spectra of **S2**

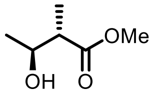

S2

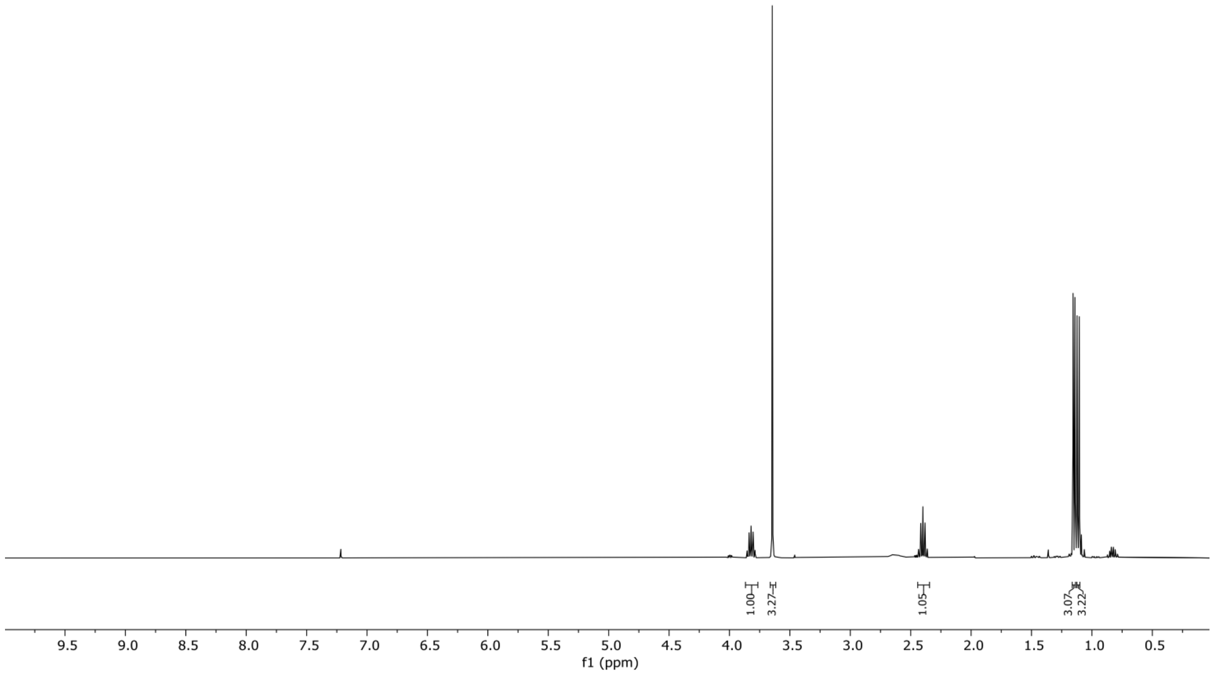

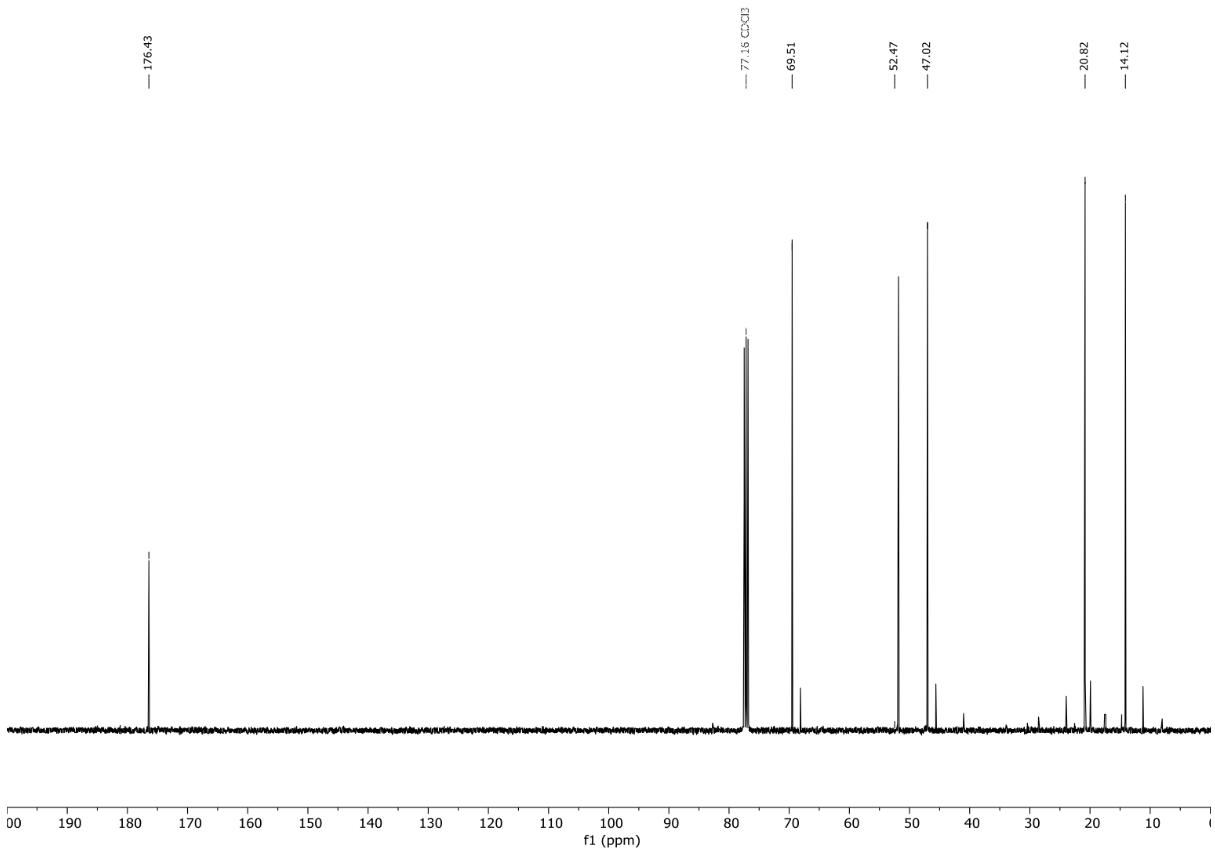
176.43
77.16 CDCl3
69.51
52.47
47.02
20.82
14.12
190
180
170
160
150
140
130
120
110
100
90
80
70
60
50
40
30
20
10
f1 (ppm)

Figure S16. ^{1}H (top) and ^{13}C (bottom) NMR spectra of **S3**

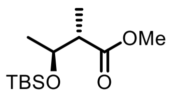

S3

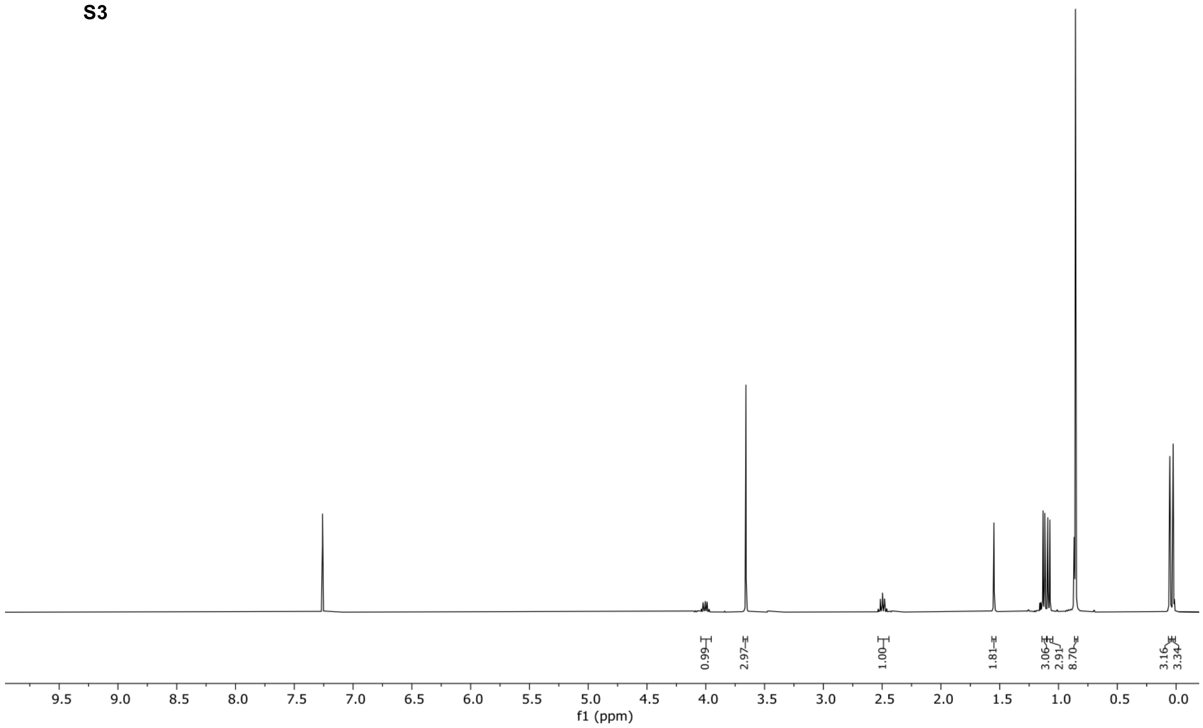

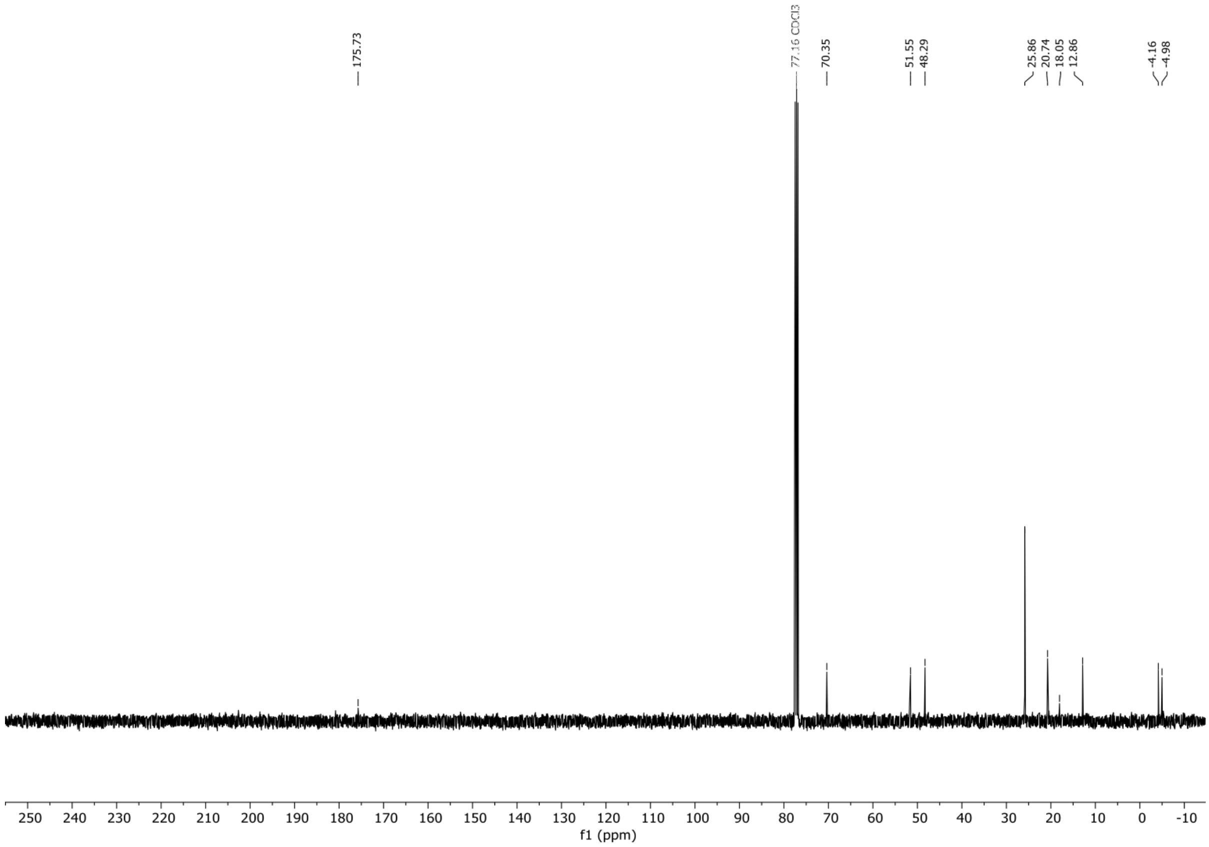
175.73
77.16 CDCl3
70.35
51.55
48.29
25.86
20.74
18.05
12.86
-4.16
-4.98
250 240 230 220 210 200 190 180 170 160 150 140 130 120 110 100 90 80 70 60 50 40 30 20 10 0 -10
f1 (ppm)

Figure S17. ^{1}H (top) and ^{13}C (bottom) NMR spectra of **S4**

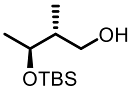

S4

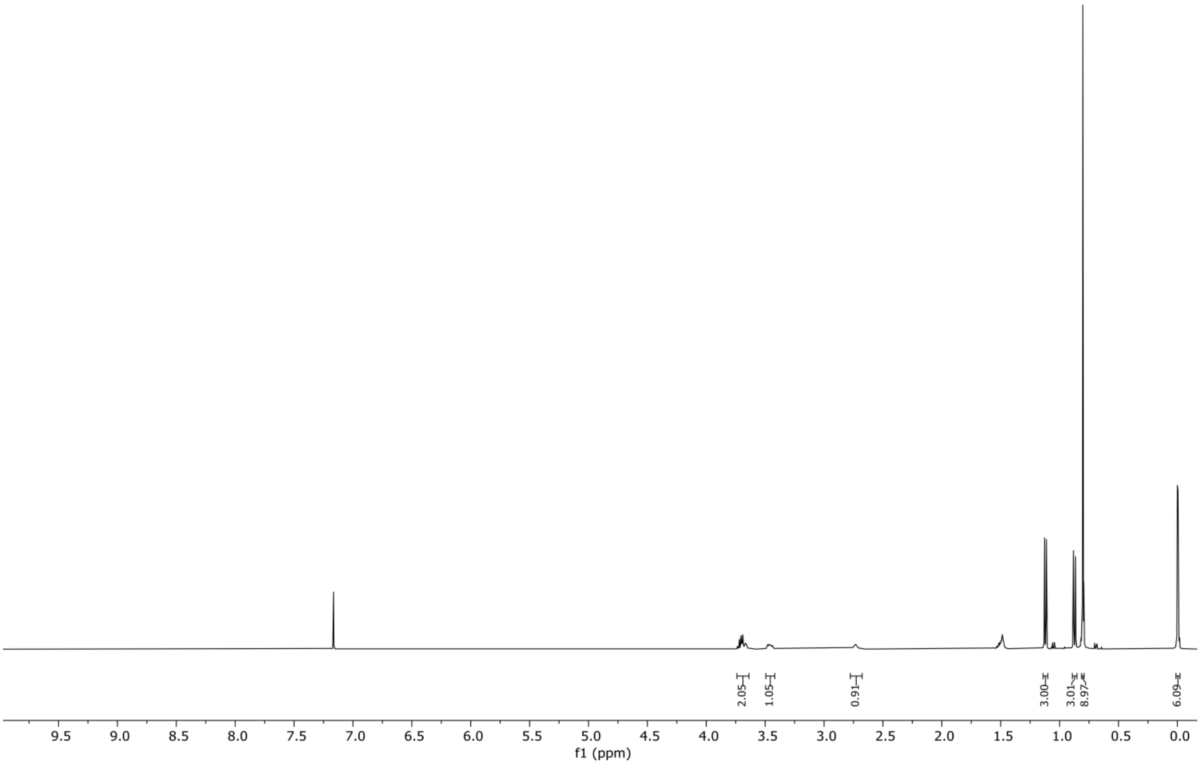

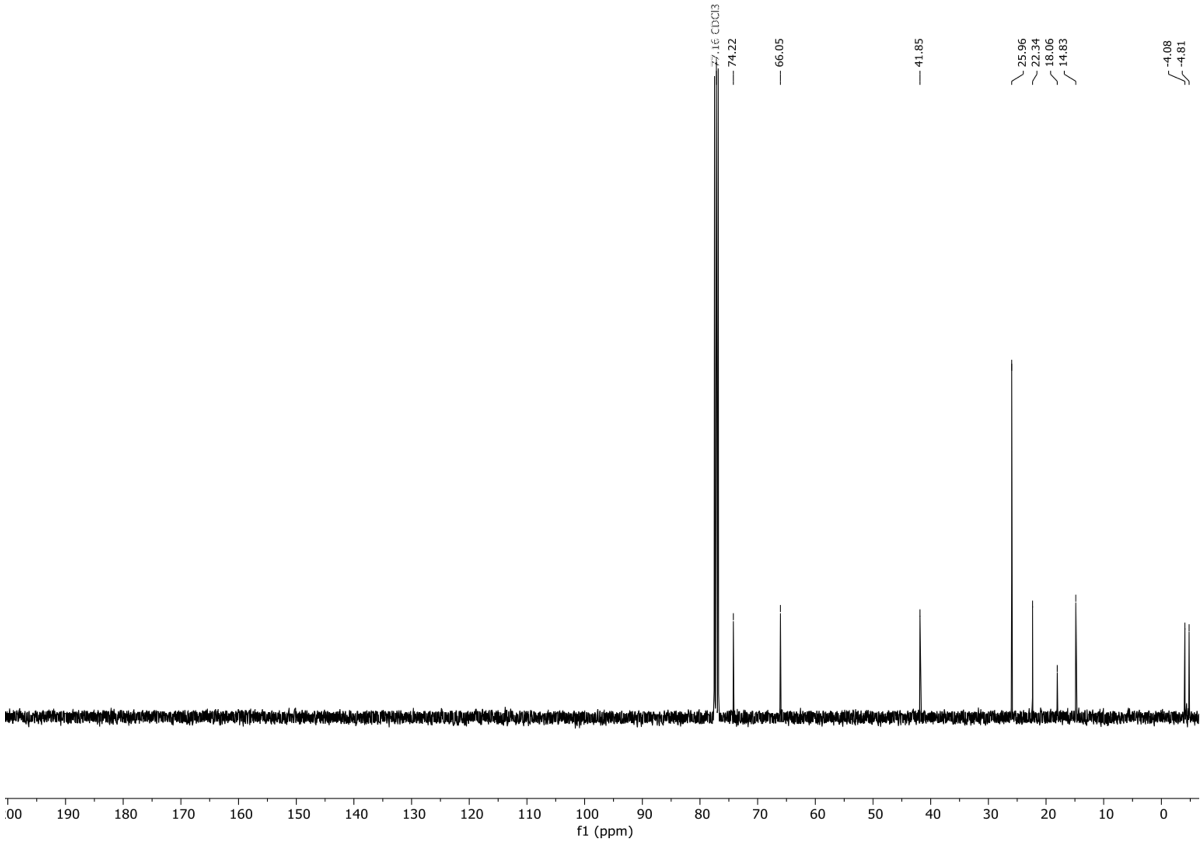

Figure S18. ^{1}H (top) and ^{13}C (bottom) NMR spectra of **S5**

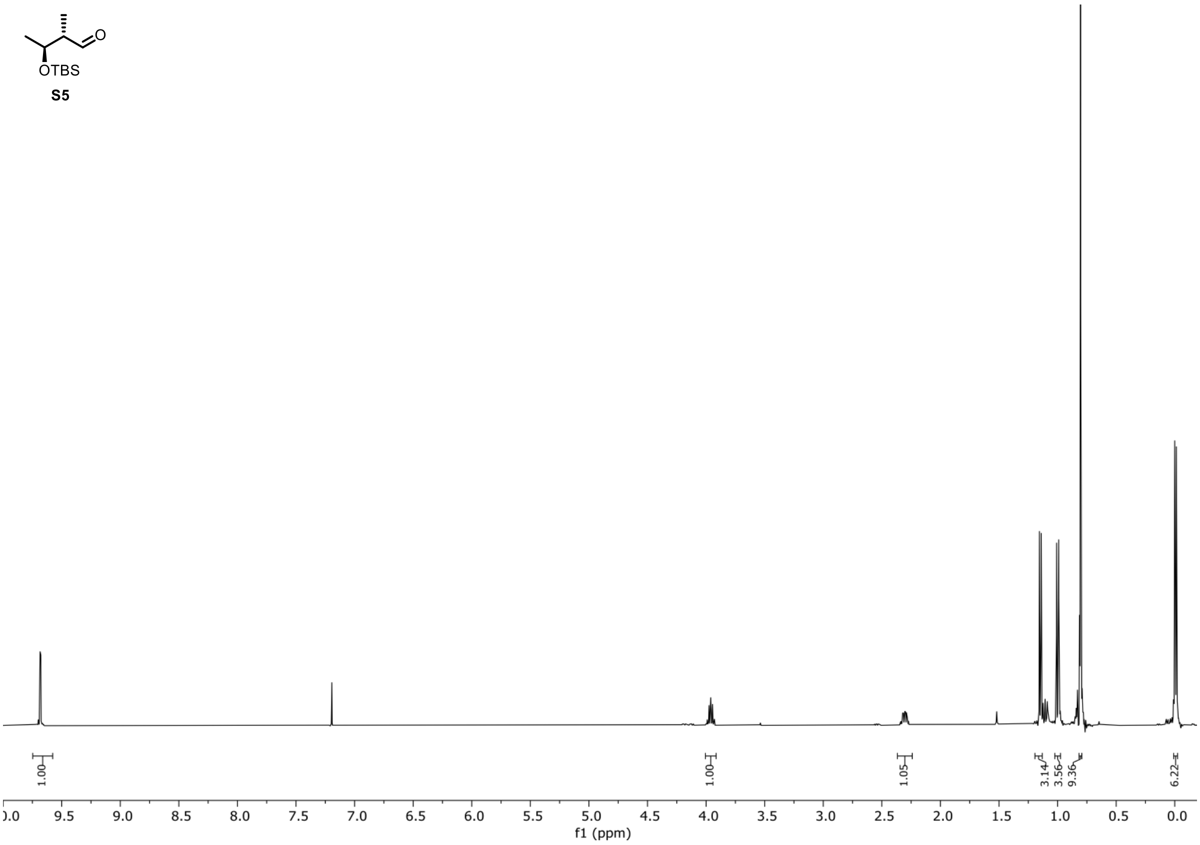

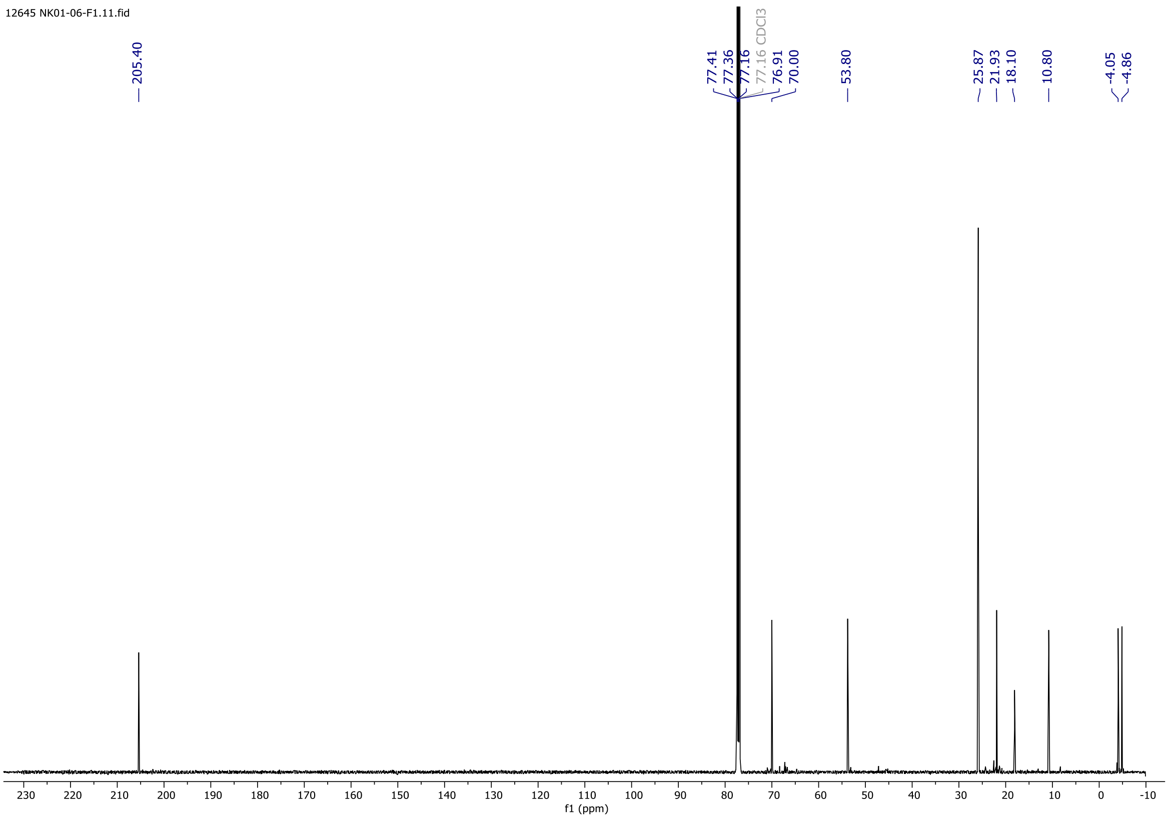

Figure S19. ^{1}H (top) and ^{13}C (bottom) NMR spectra of **1**

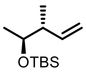

1

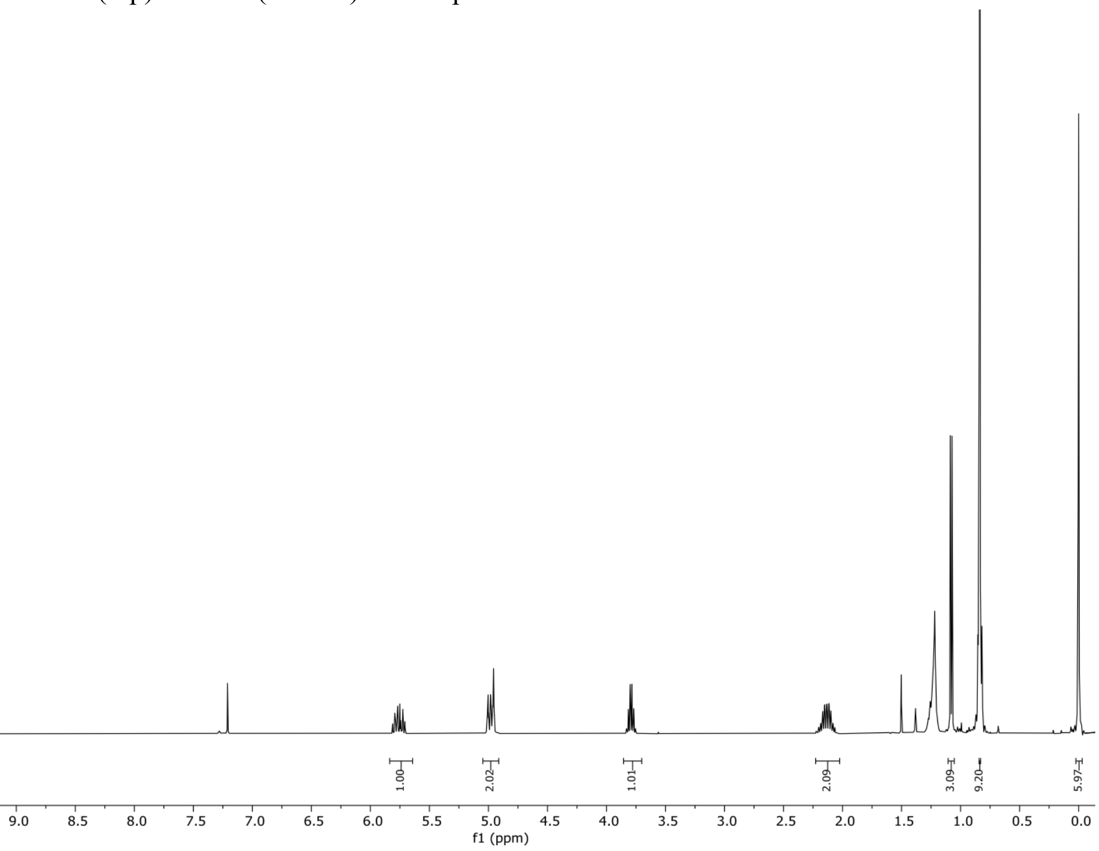

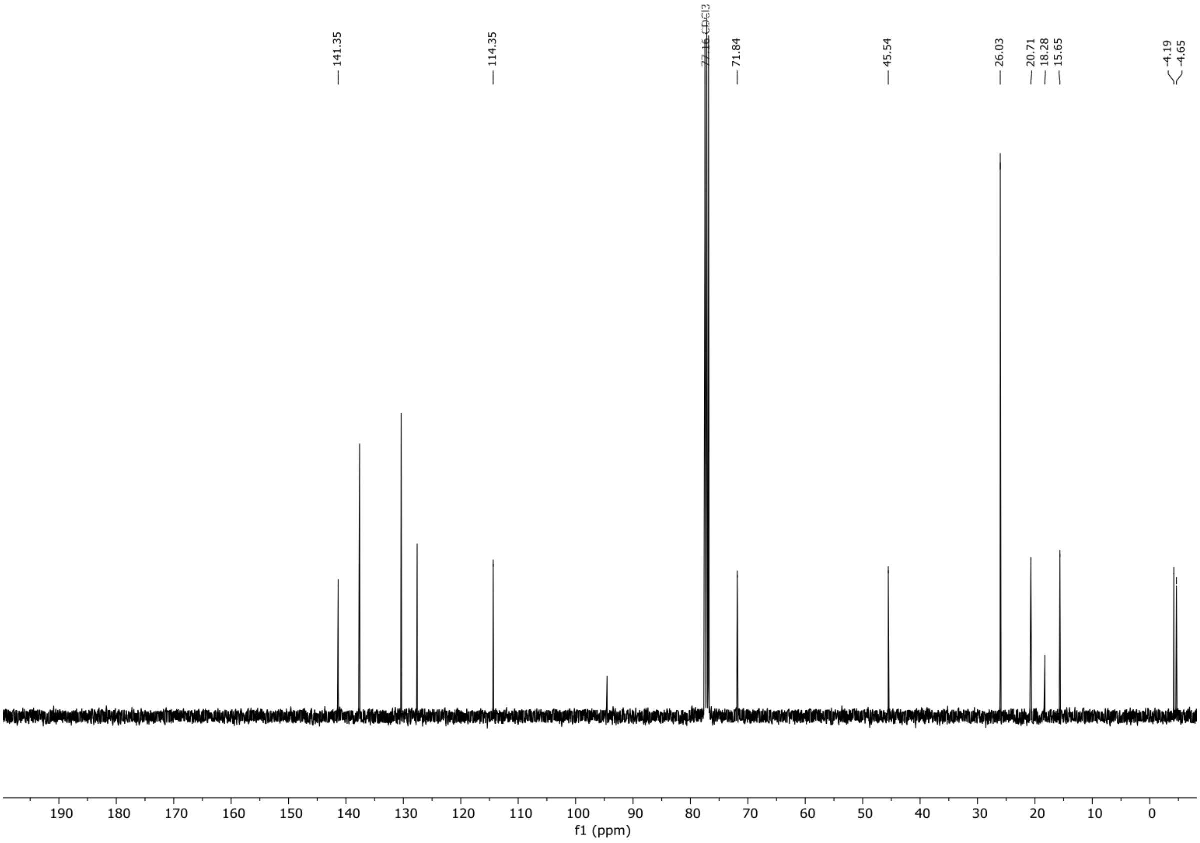

Figure S20. ^{1}H (top) and ^{13}C (bottom) NMR spectra of **5**

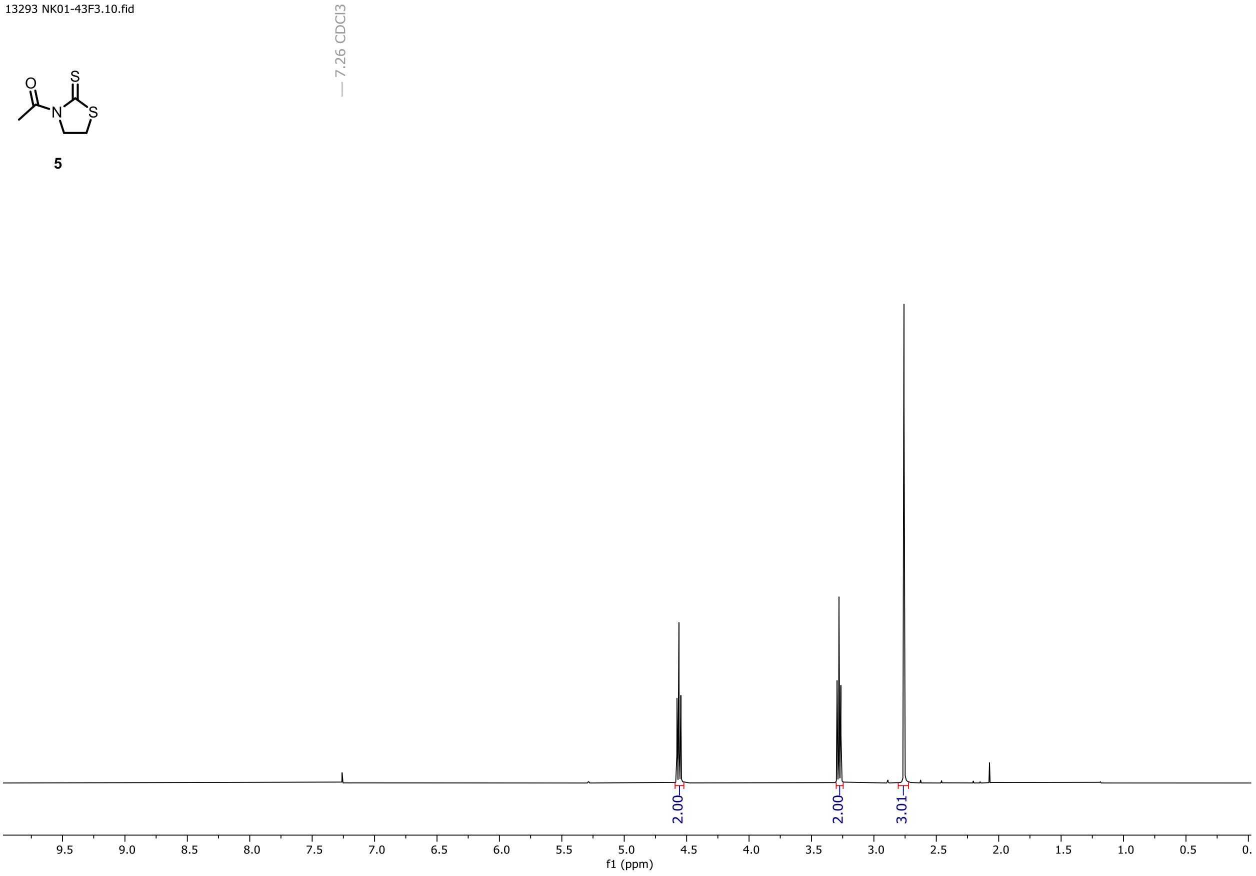

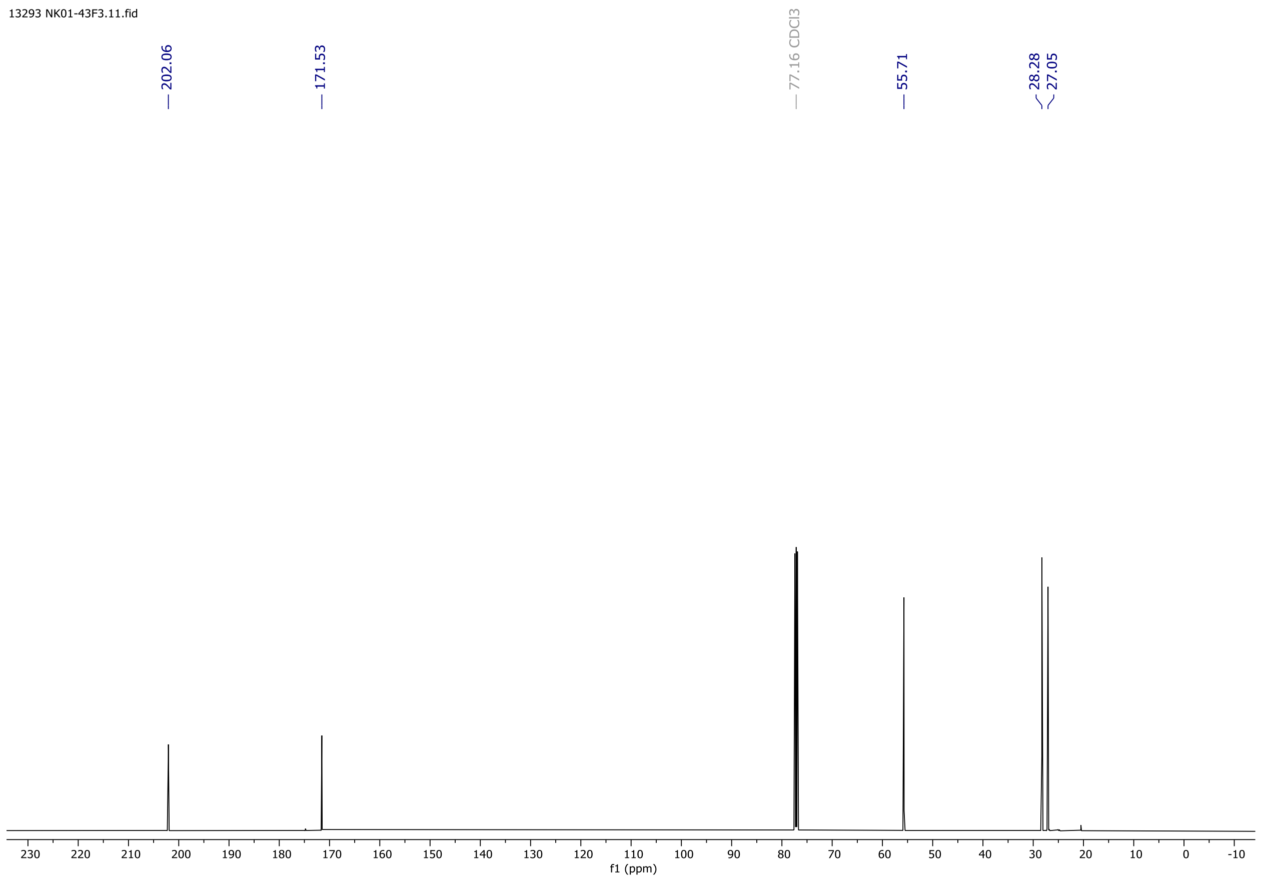
13293 NK01-43F3.11.fid
202.06
171.53
77.16 CDCl3
55.71
28.28
27.05
230 220 210 200 190 180 170 160 150 140 130 120 110 100 90 80 70 60 50 40 30 20 10 0 -10
f1 (ppm)

Figure S21. ^{1}H (top) and ^{13}C (bottom) NMR spectra of **(7-^{13}C) 5**

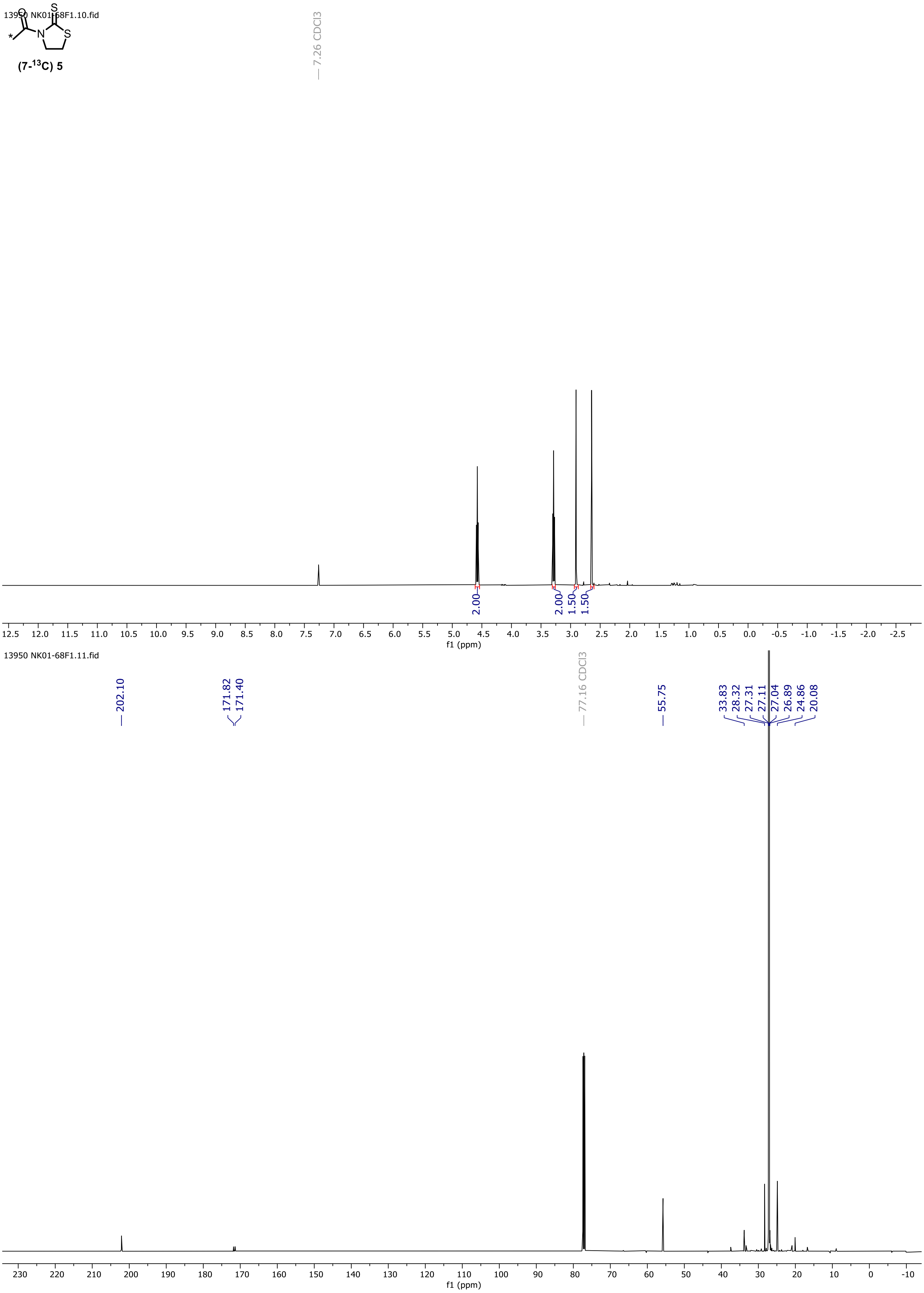

Figure S22. ^{1}H (top) and ^{13}C (bottom) NMR spectra of **S8**

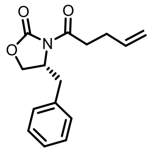

S8

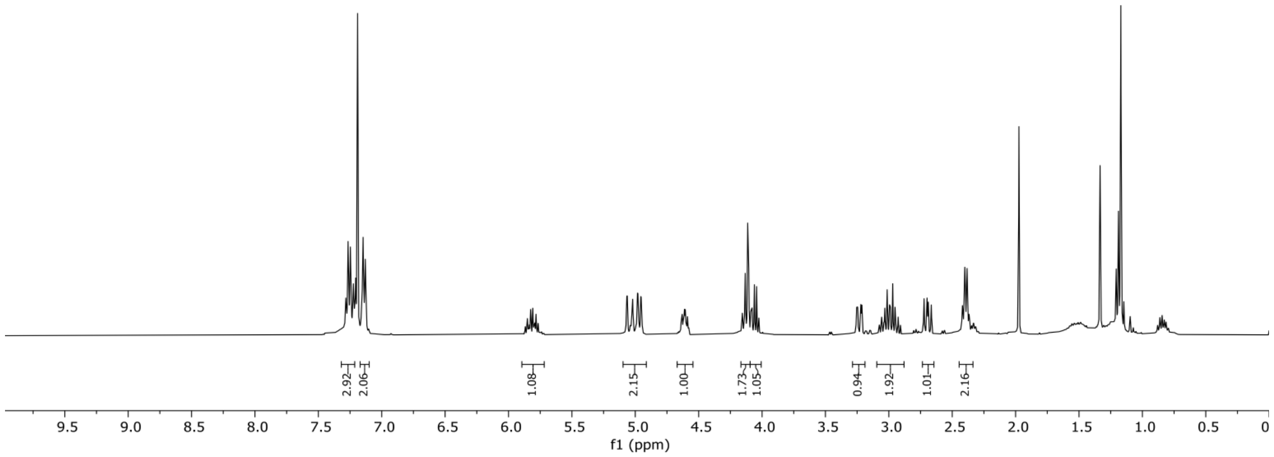

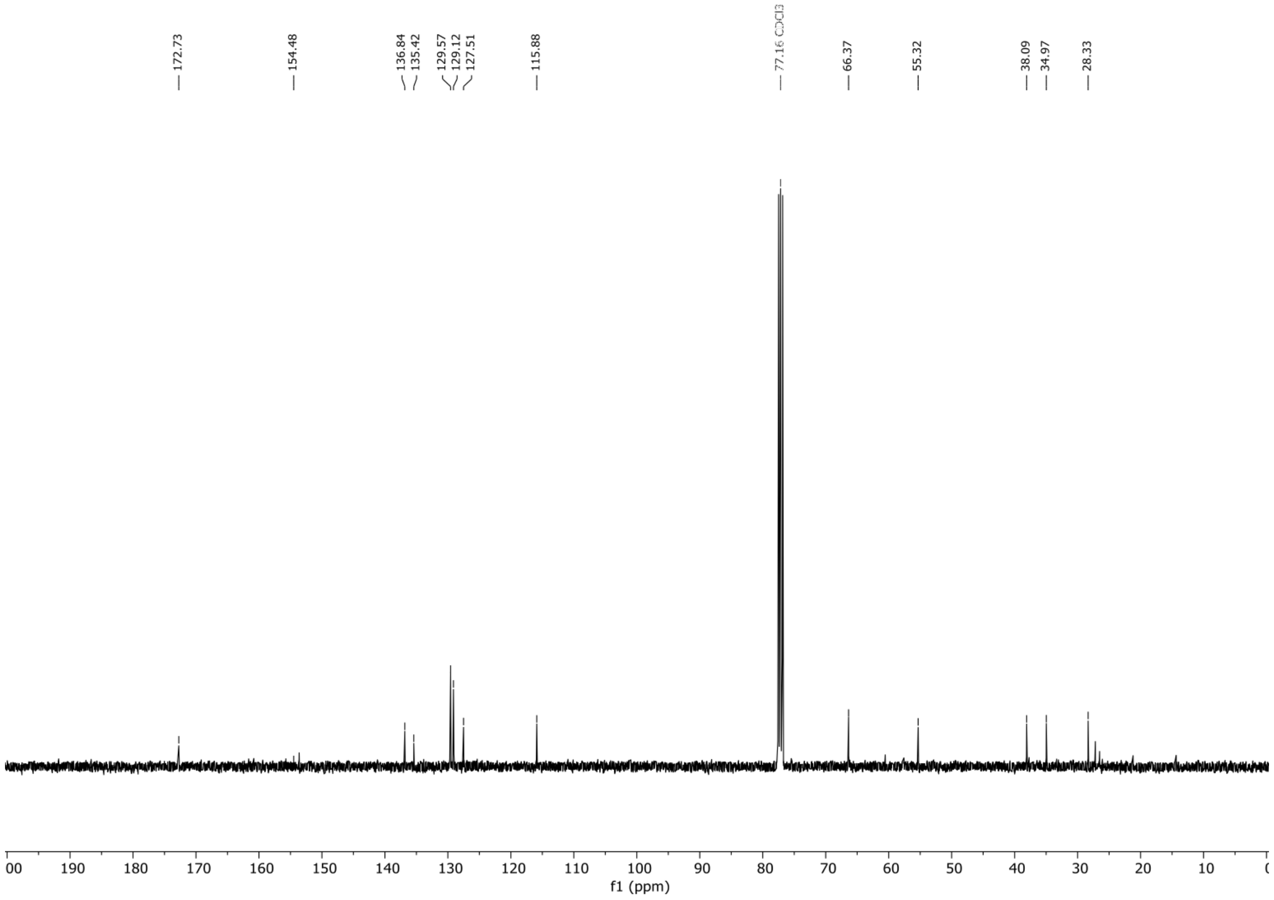

Figure S23. ^{1}H (top) and ^{13}C (bottom) NMR spectra of **2**

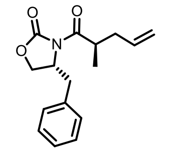

2

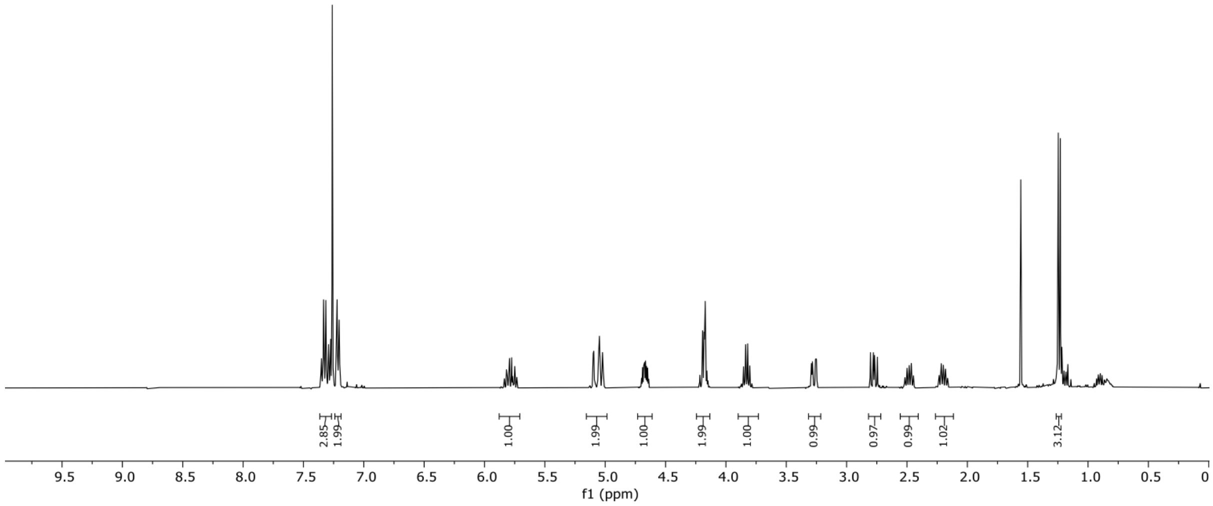

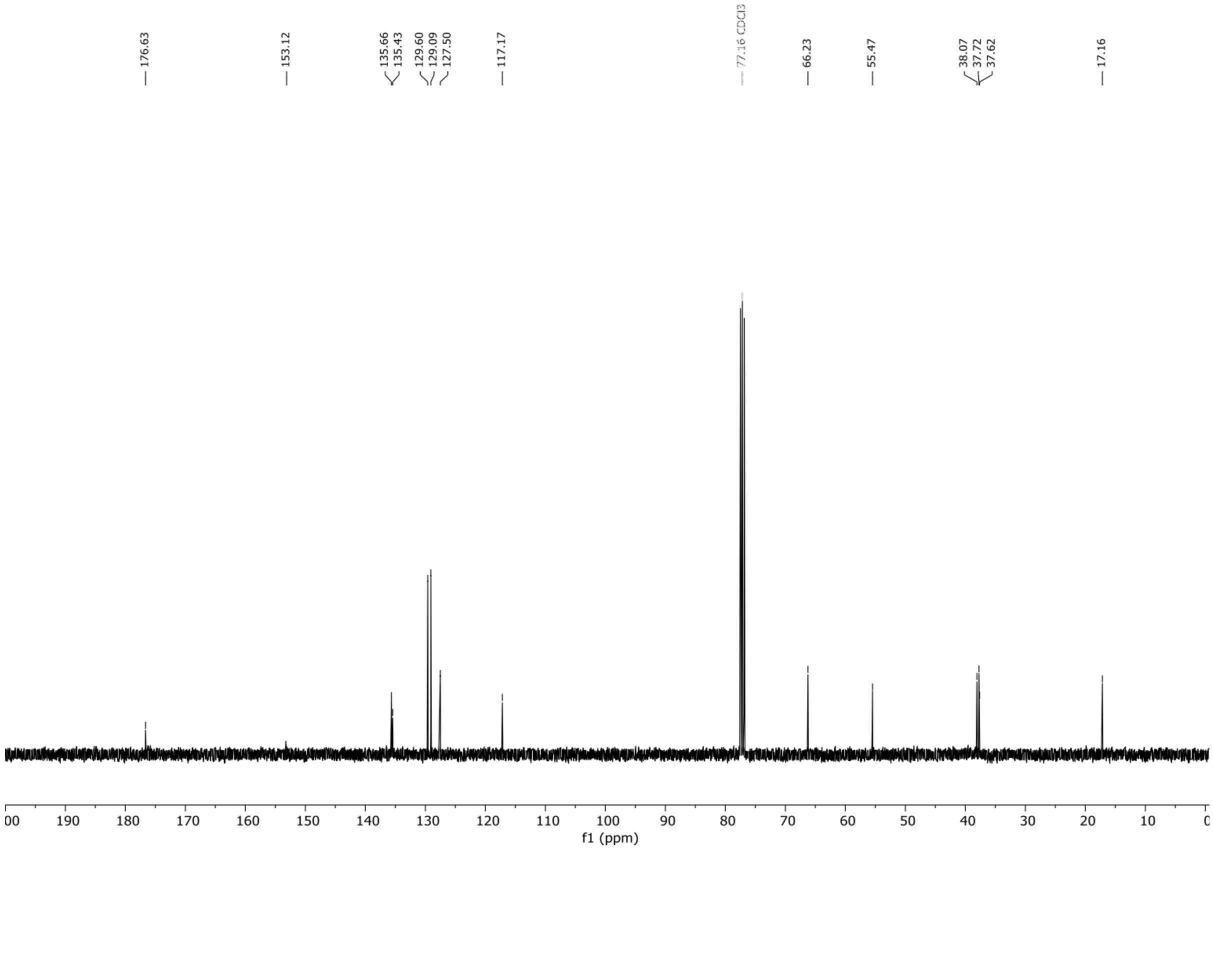

Figure S24. ^{1}H (top) and ^{13}C (bottom) NMR spectra of **3**

12929 NK01-22-F3.10.fid

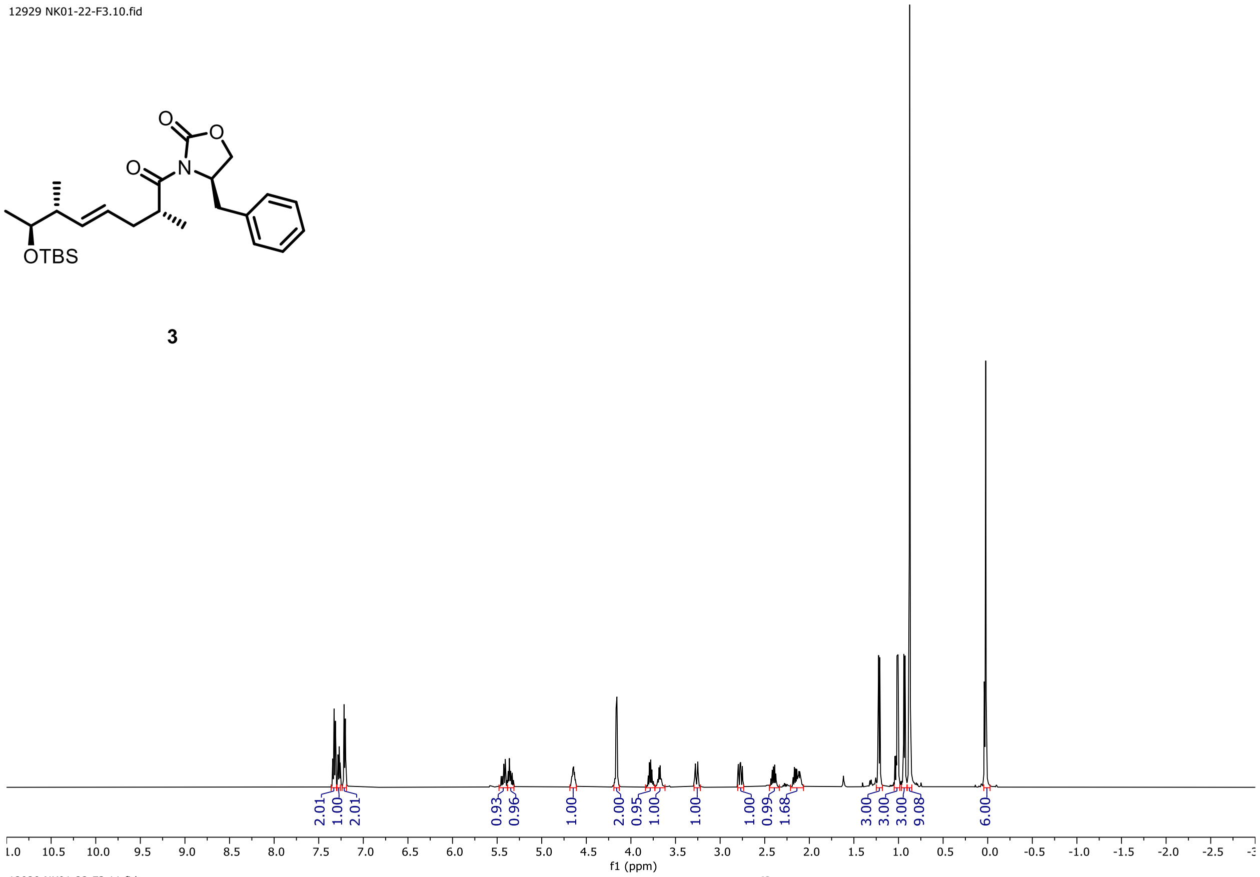

12929 NK01-22-F3.11.fid

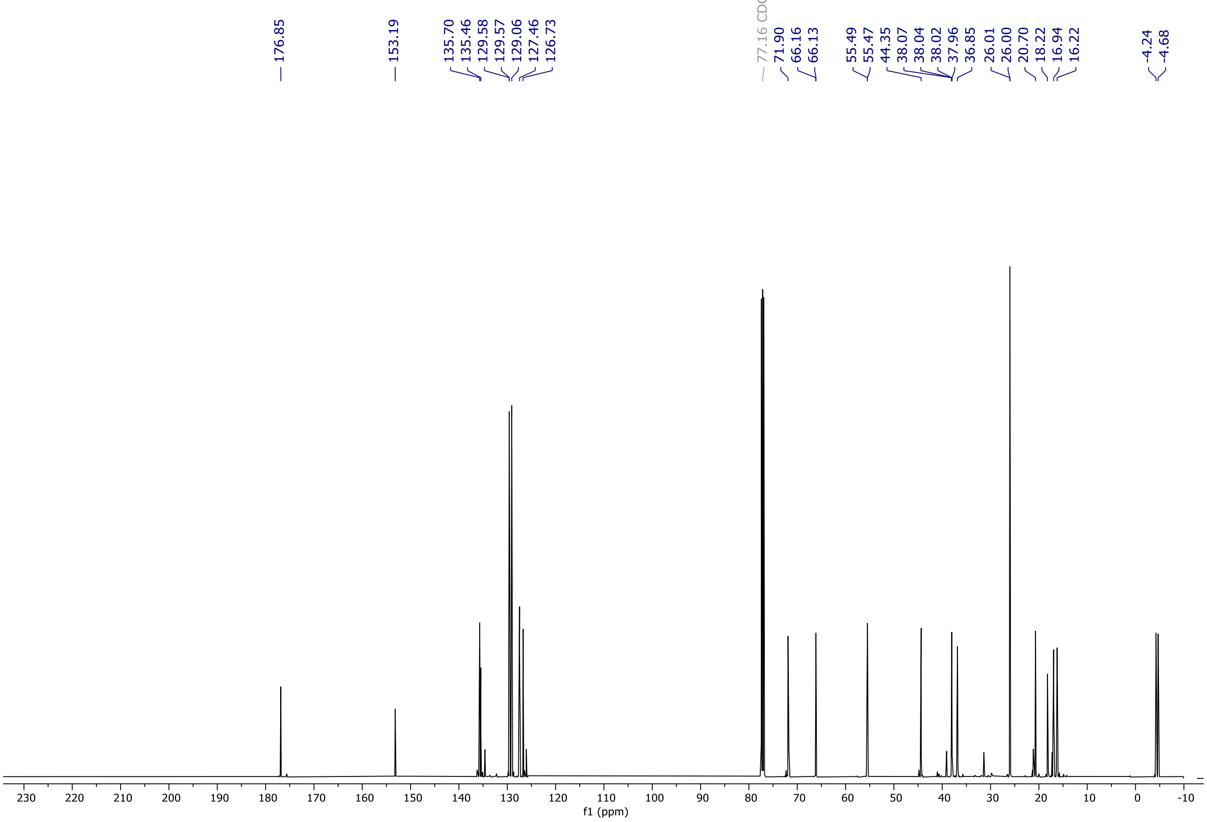

Figure S25. ^{1}H (top) and ^{13}C (bottom) NMR spectra of **S9**

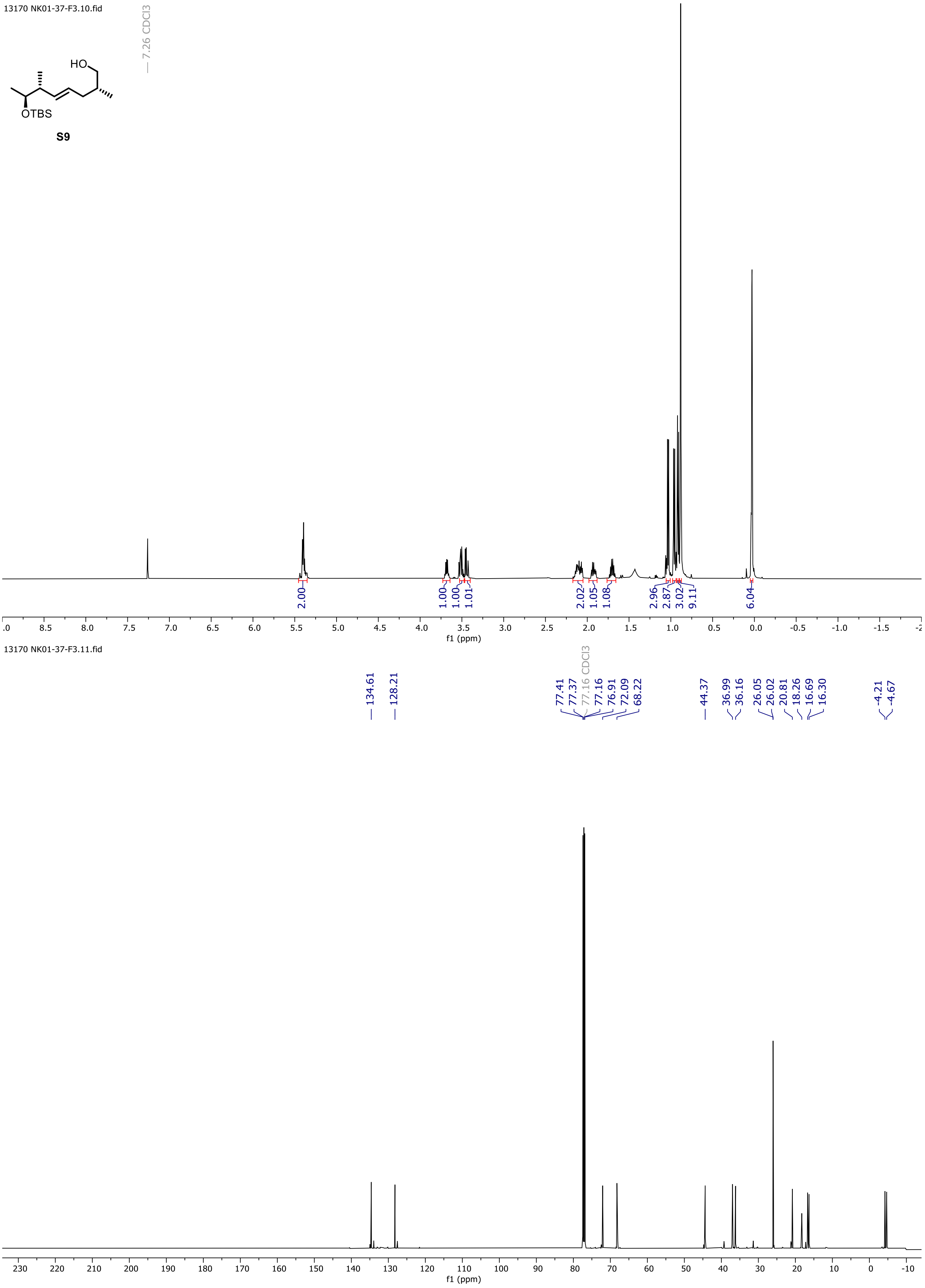

Figure S26. ^{1}H (top) and ^{13}C (bottom) NMR spectra of **4**

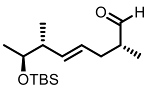

4

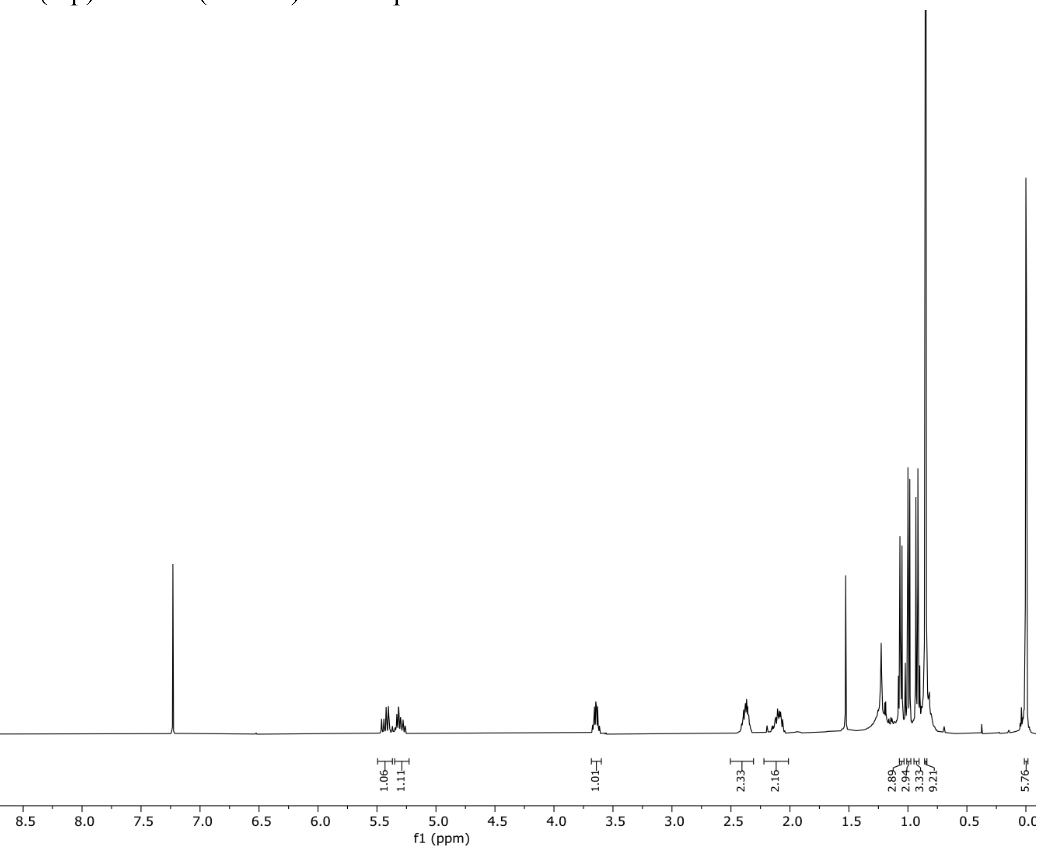

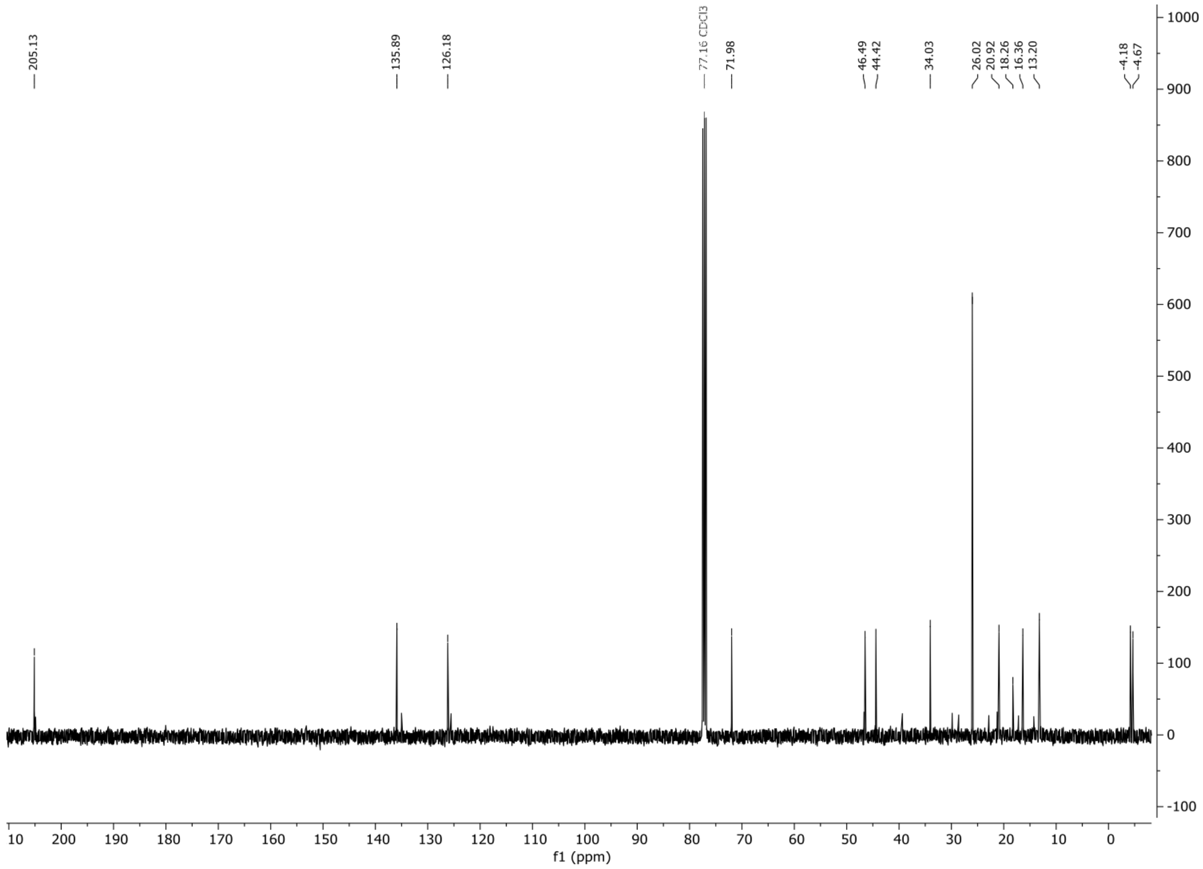
205.13
135.89
126.18
77.16 CDCl3
71.98
46.49
44.42
34.03
26.02
20.92
18.26
16.36
13.20
-4.18
-4.67
f1 (ppm)

Figure S27. ^{1}H (top) and ^{13}C (bottom) NMR spectra of **6**

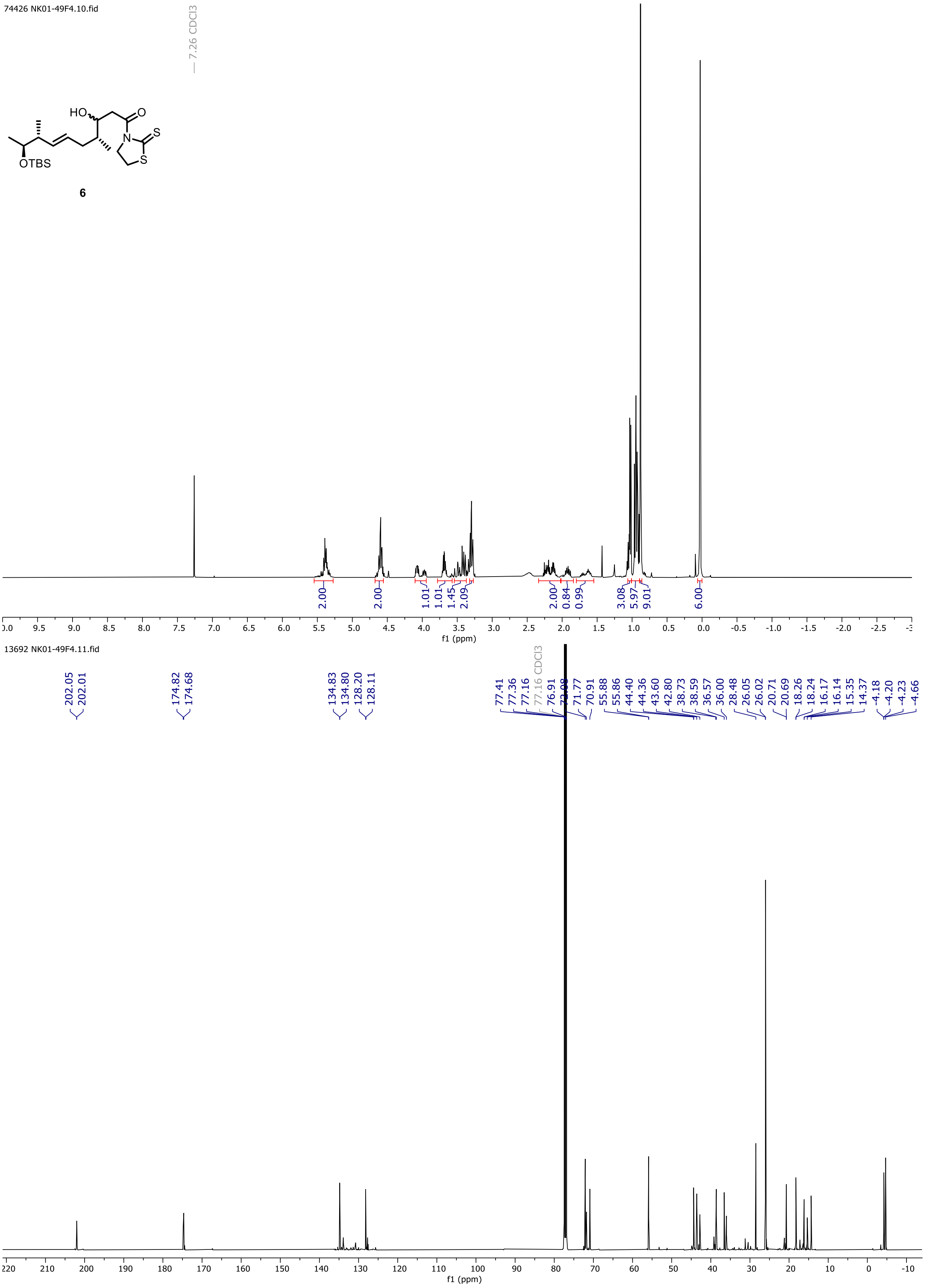

Figure S28. ^{1}H (top) and ^{13}C (bottom) NMR spectra of **S22**

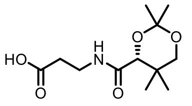

S22

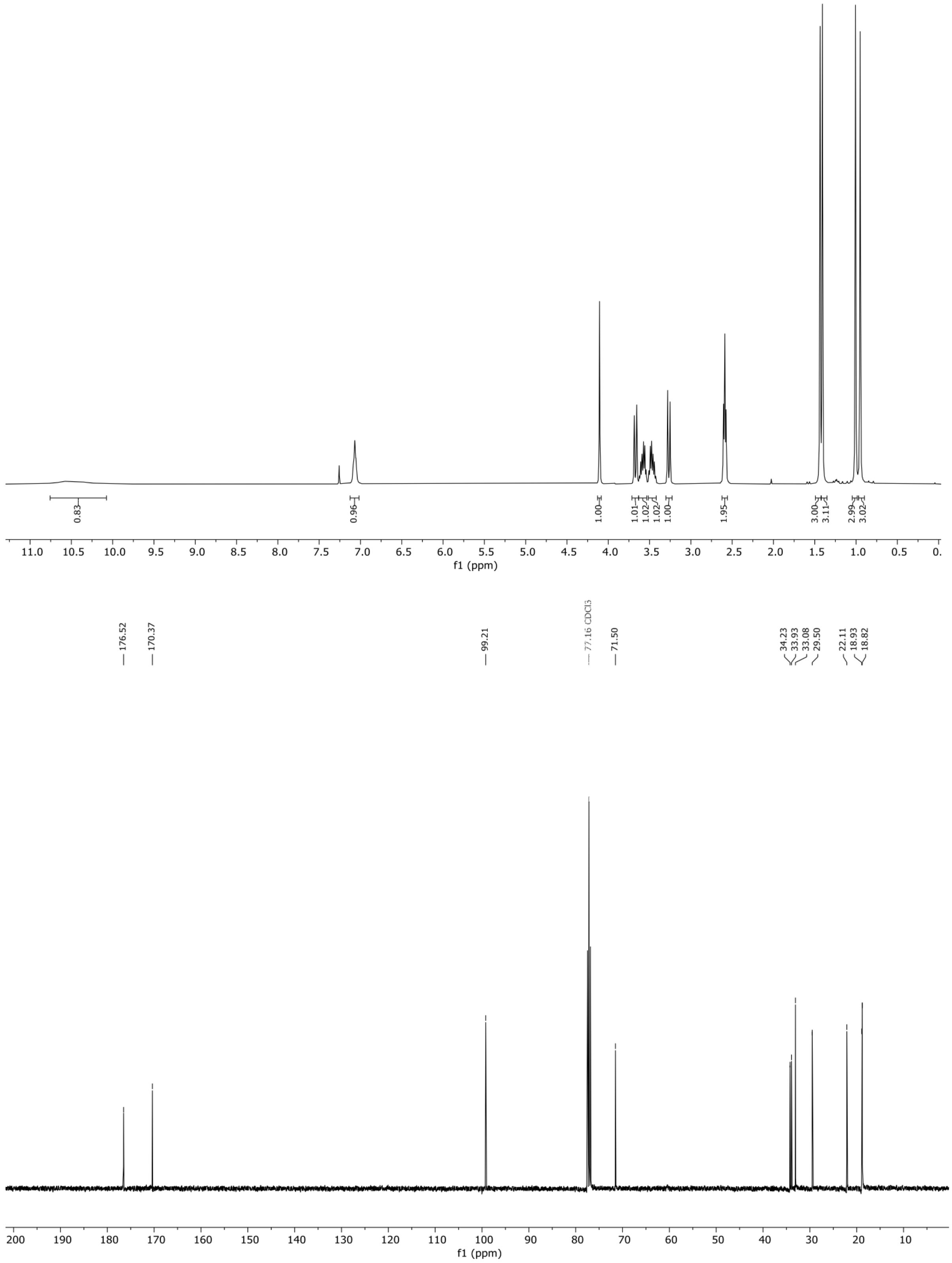

Figure S29. ^{1}H (top) and ^{13}C (bottom) NMR spectra of **7**

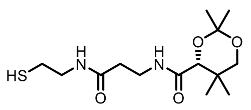

7

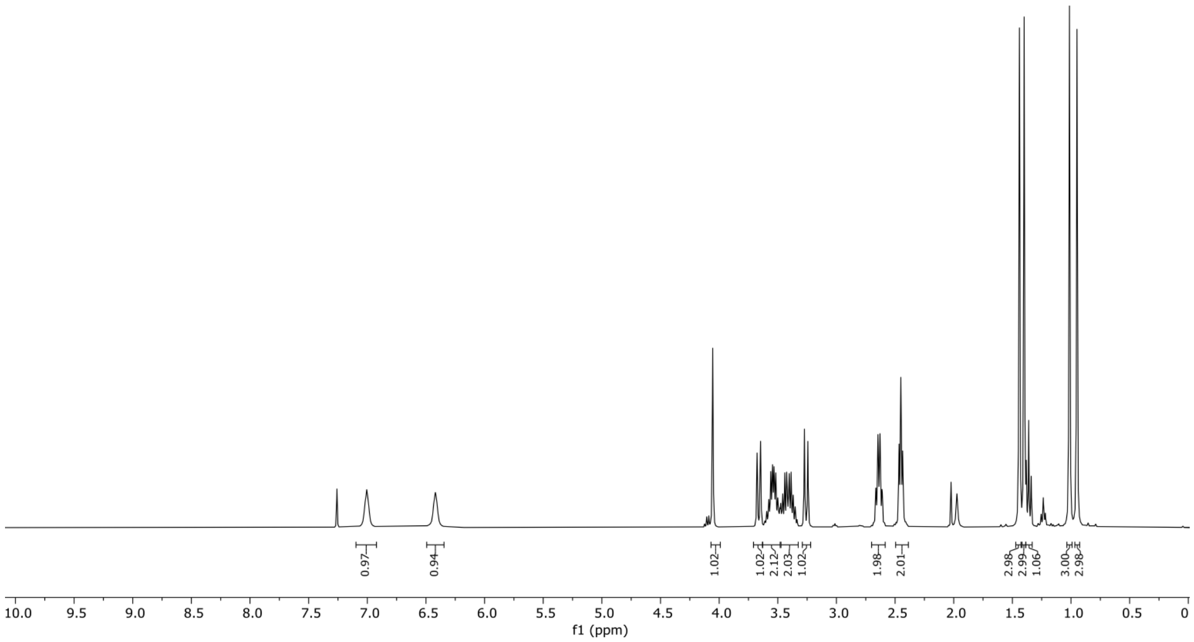

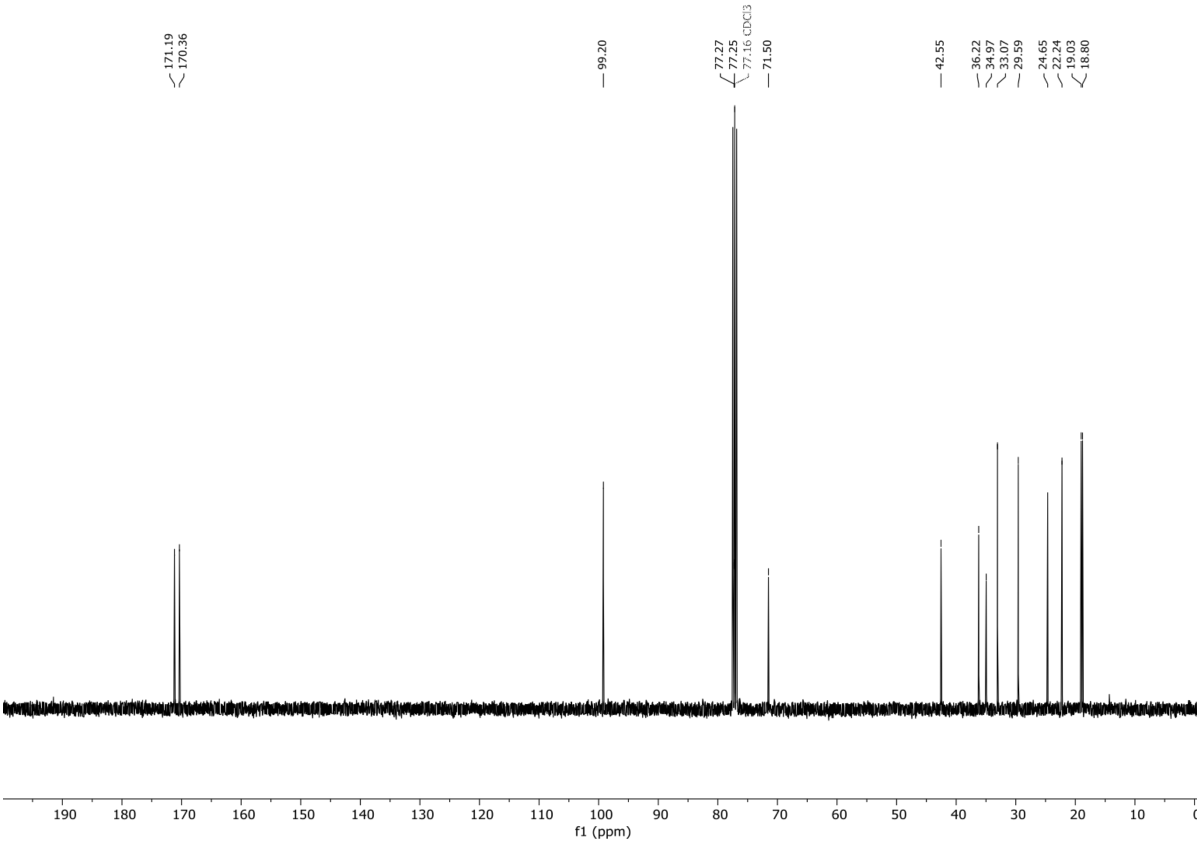

Figure S30. ^{1}H (top) and ^{13}C (bottom) NMR spectra of **8**

73382 NK01-50-F3.10.fid

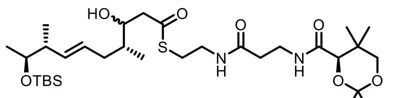

8

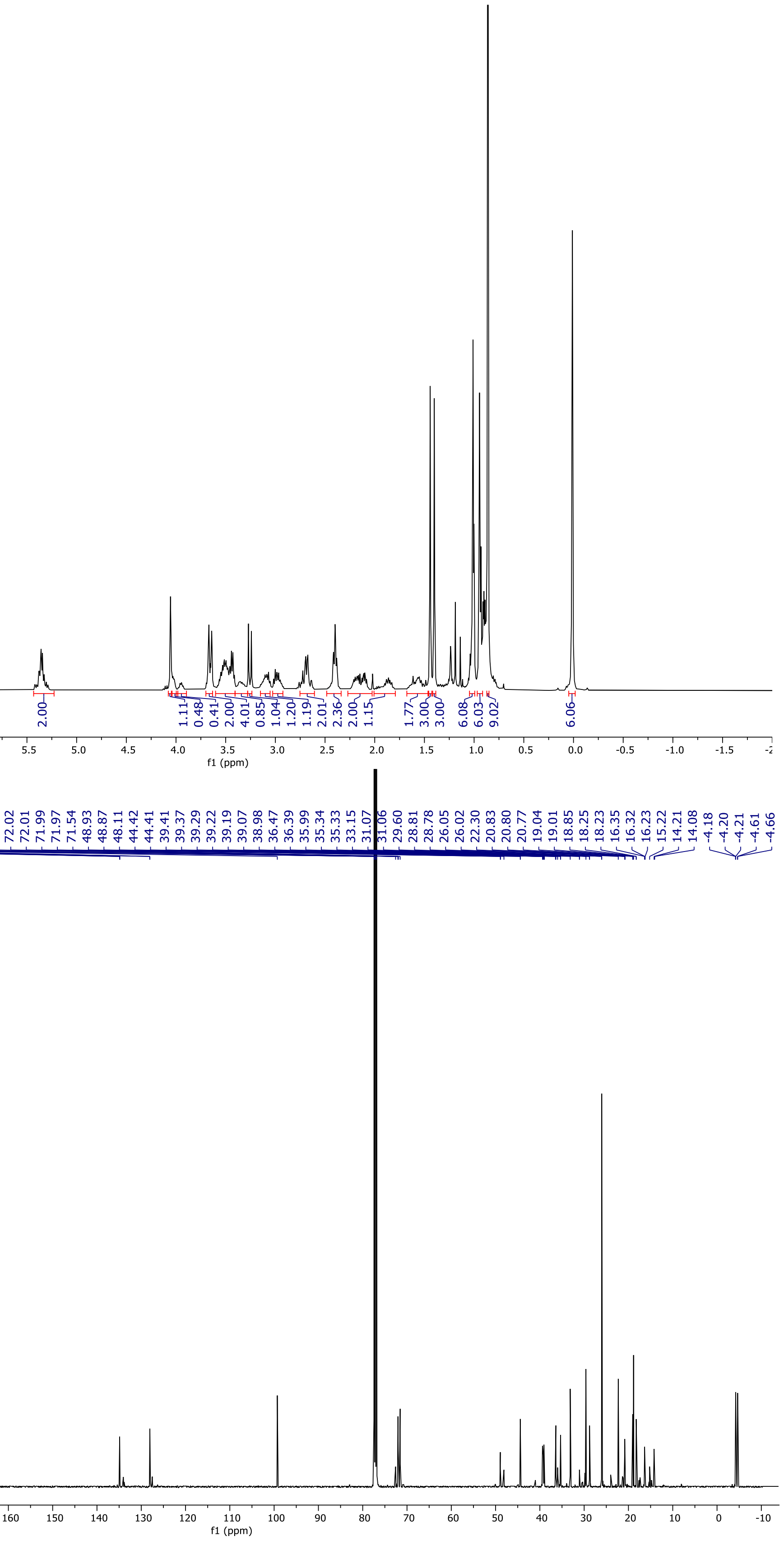

Figure S31. ^{1}H (top) and ^{13}C (bottom) NMR spectra of **S10**

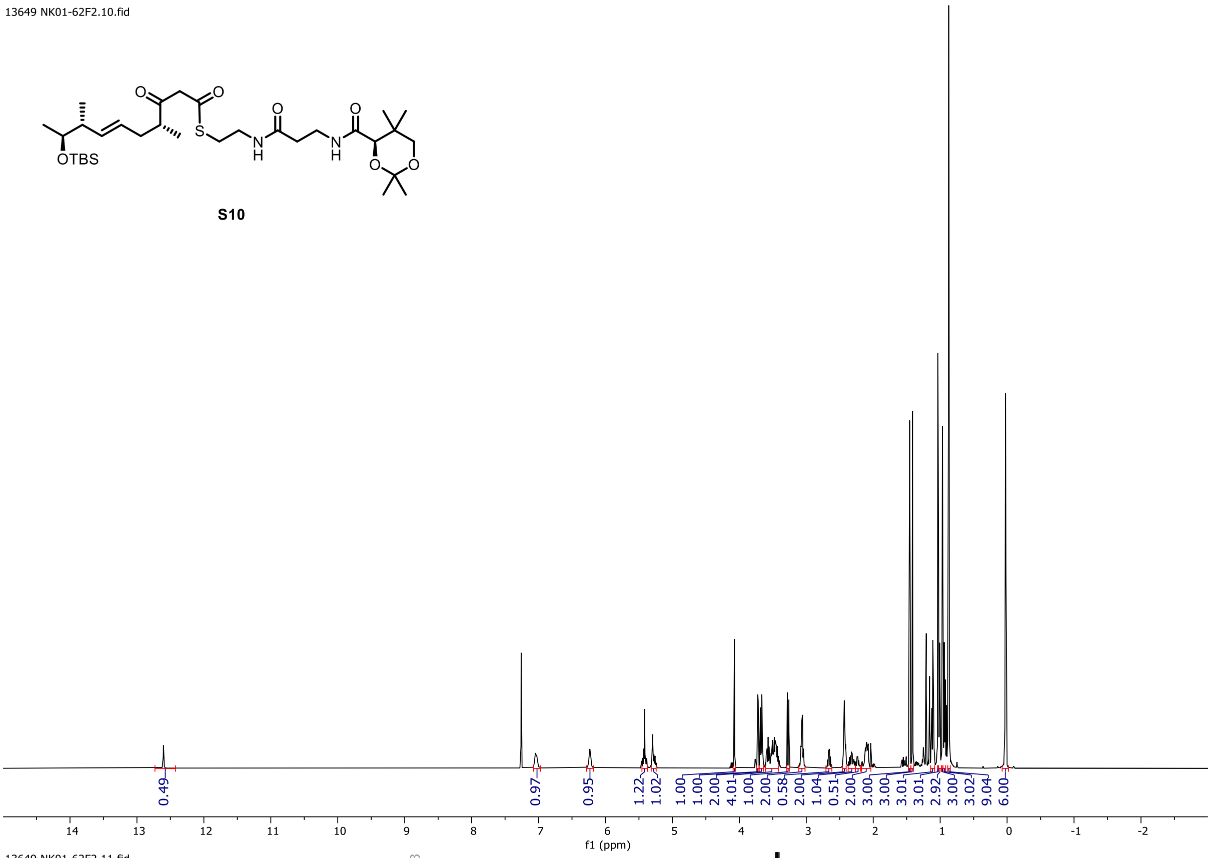

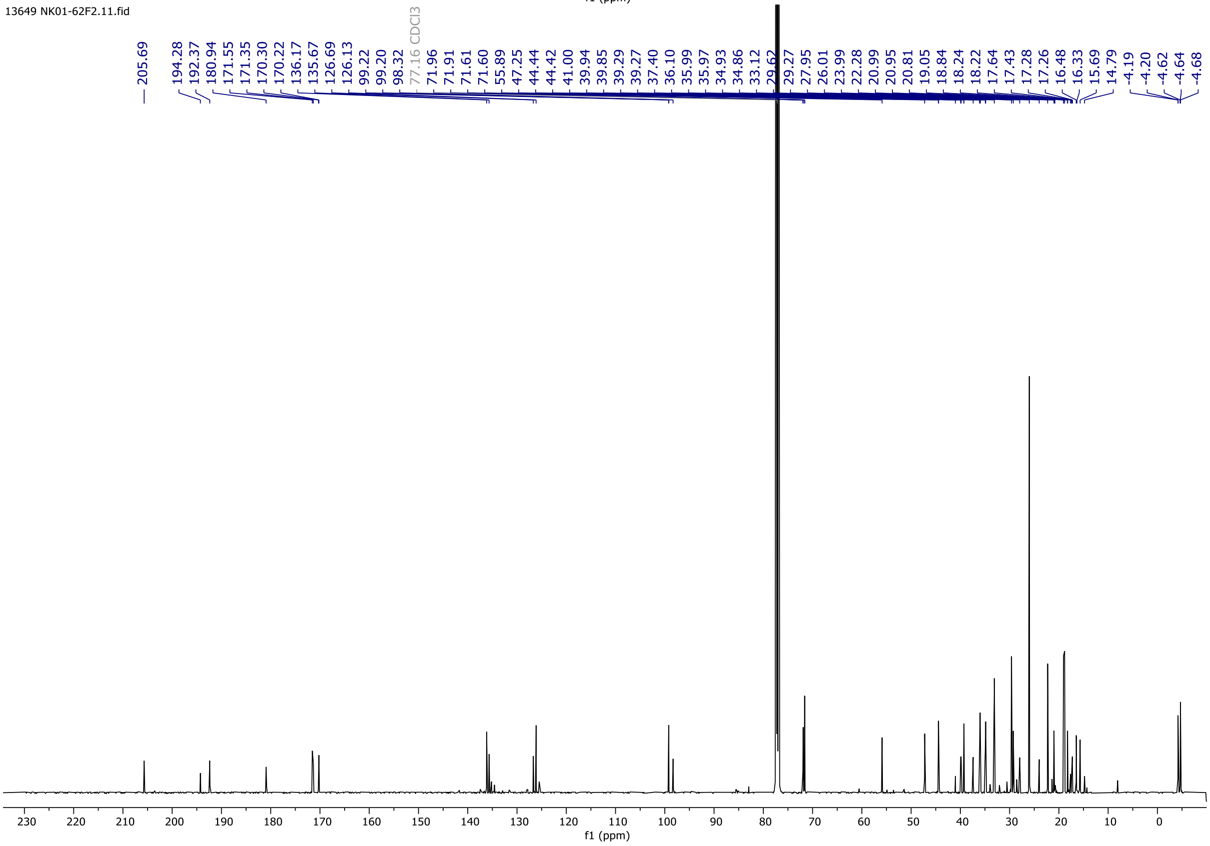

Figure S32. ^{1}H (top) and ^{13}C (bottom) NMR spectra of **9**

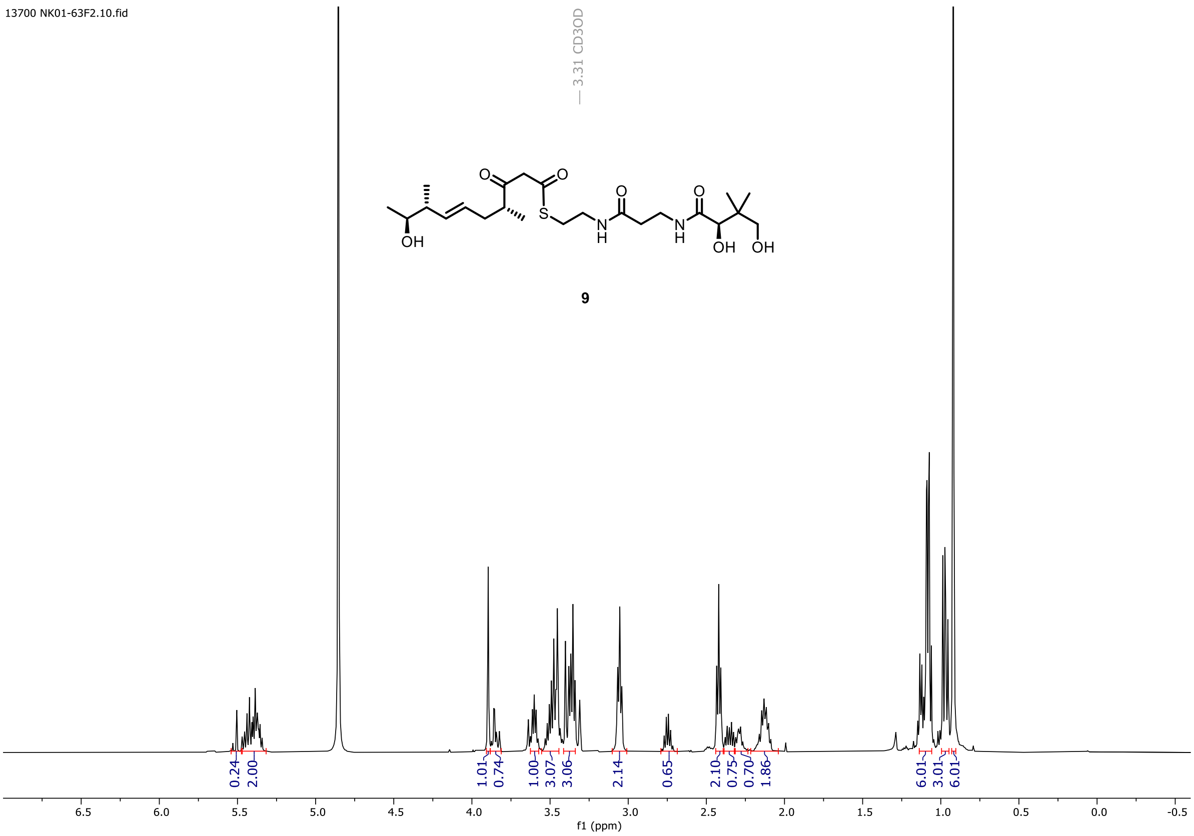
13700 NK01-63F2.10.fid
3.31 CD3OD
9
0.24
2.00
1.01
0.74
1.00
3.07
3.06
2.14
0.65
2.10
0.75
0.70
1.86
6.01
3.01
6.01
f1 (ppm)

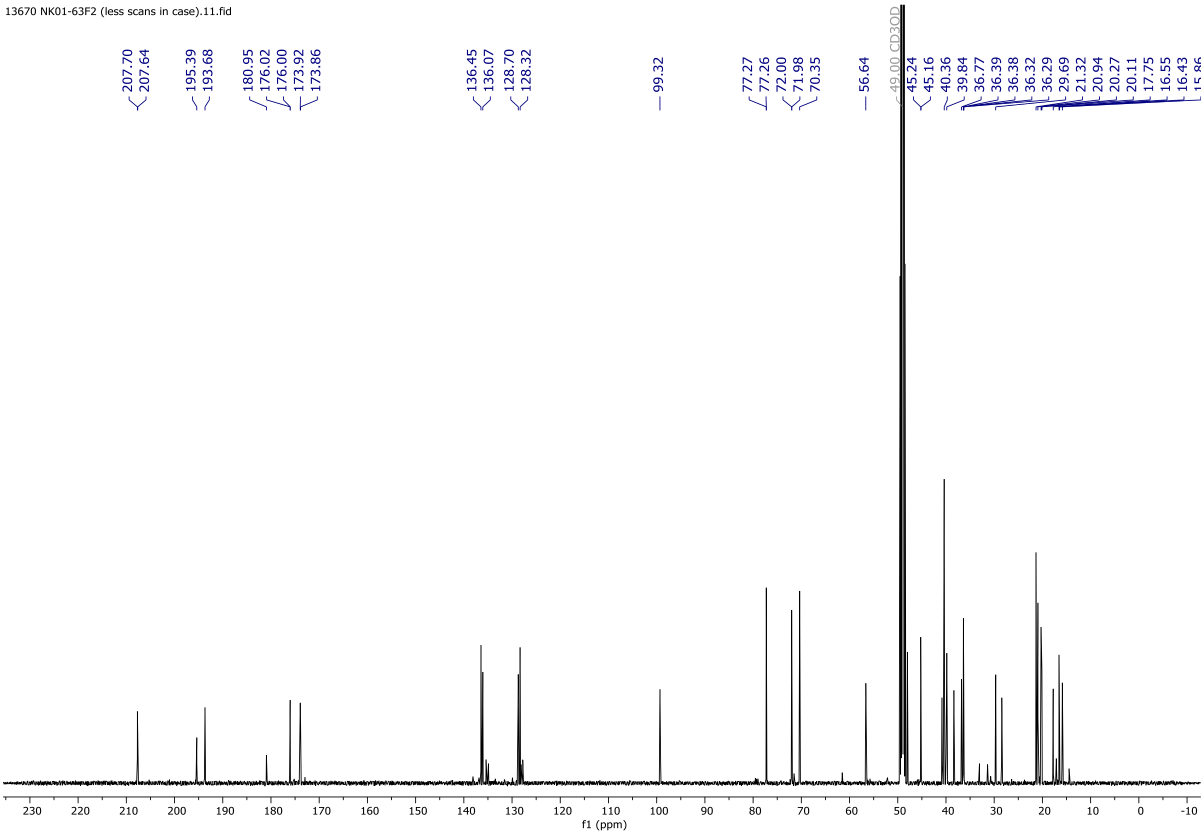
13670 NK01-63F2 (less scans in case).11.fid
207.70
207.64
195.39
193.68
180.95
176.02
176.00
173.92
173.86
136.45
136.07
128.70
128.32
99.32
77.27
77.26
72.00
71.98
70.35
56.64
49.00 CD3OD
45.24
45.16
40.36
39.84
36.77
36.39
36.38
36.32
36.29
29.69
21.32
20.94
20.27
20.11
17.75
16.55
16.43
f1 (ppm)

Figure S33. ^{1}H (top) and ^{13}C (bottom) NMR spectra of (2-^{13}C)**9**

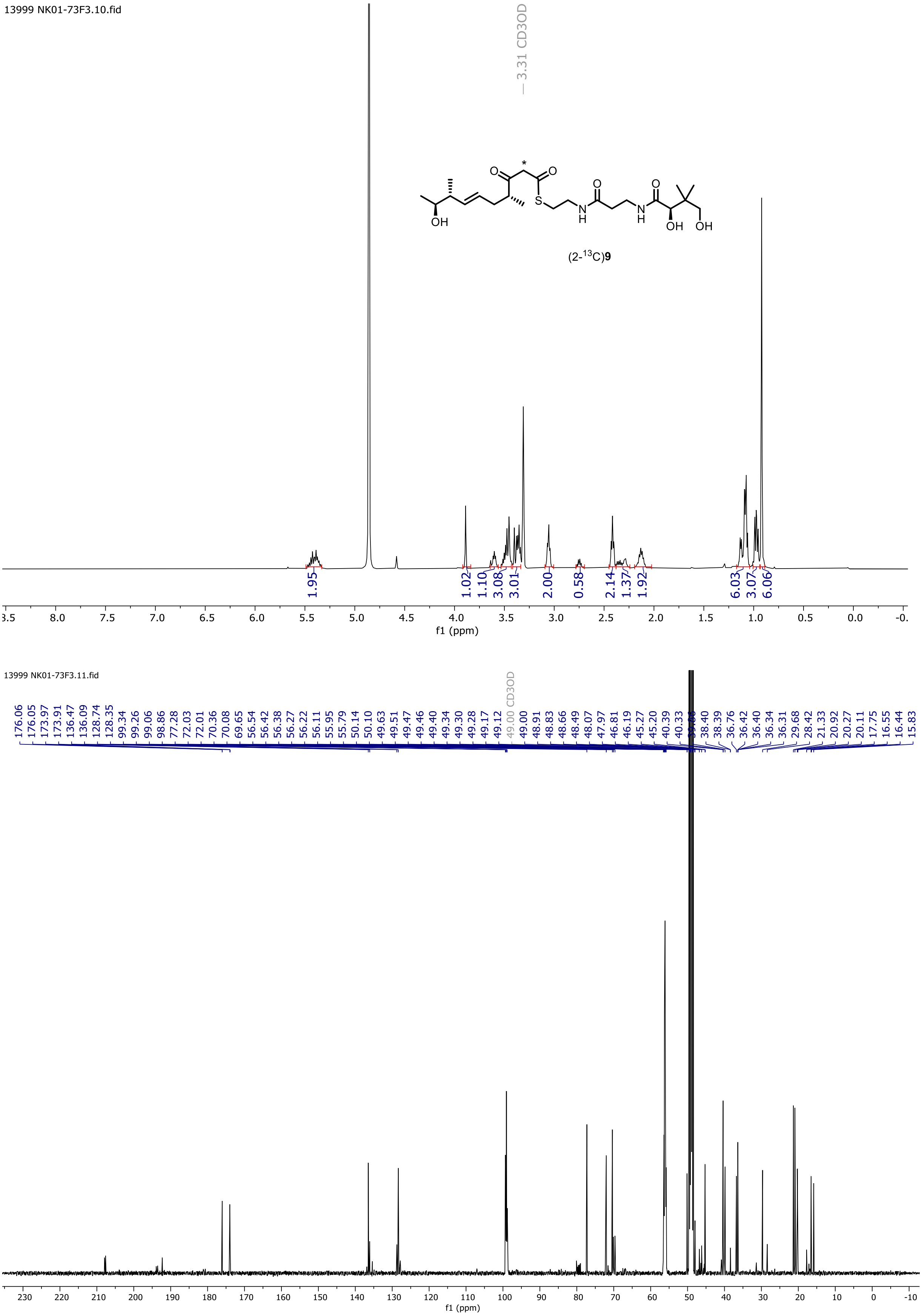

Figure S34. ^{1}H (top) and ^{13}C (bottom) NMR spectra of **S12**

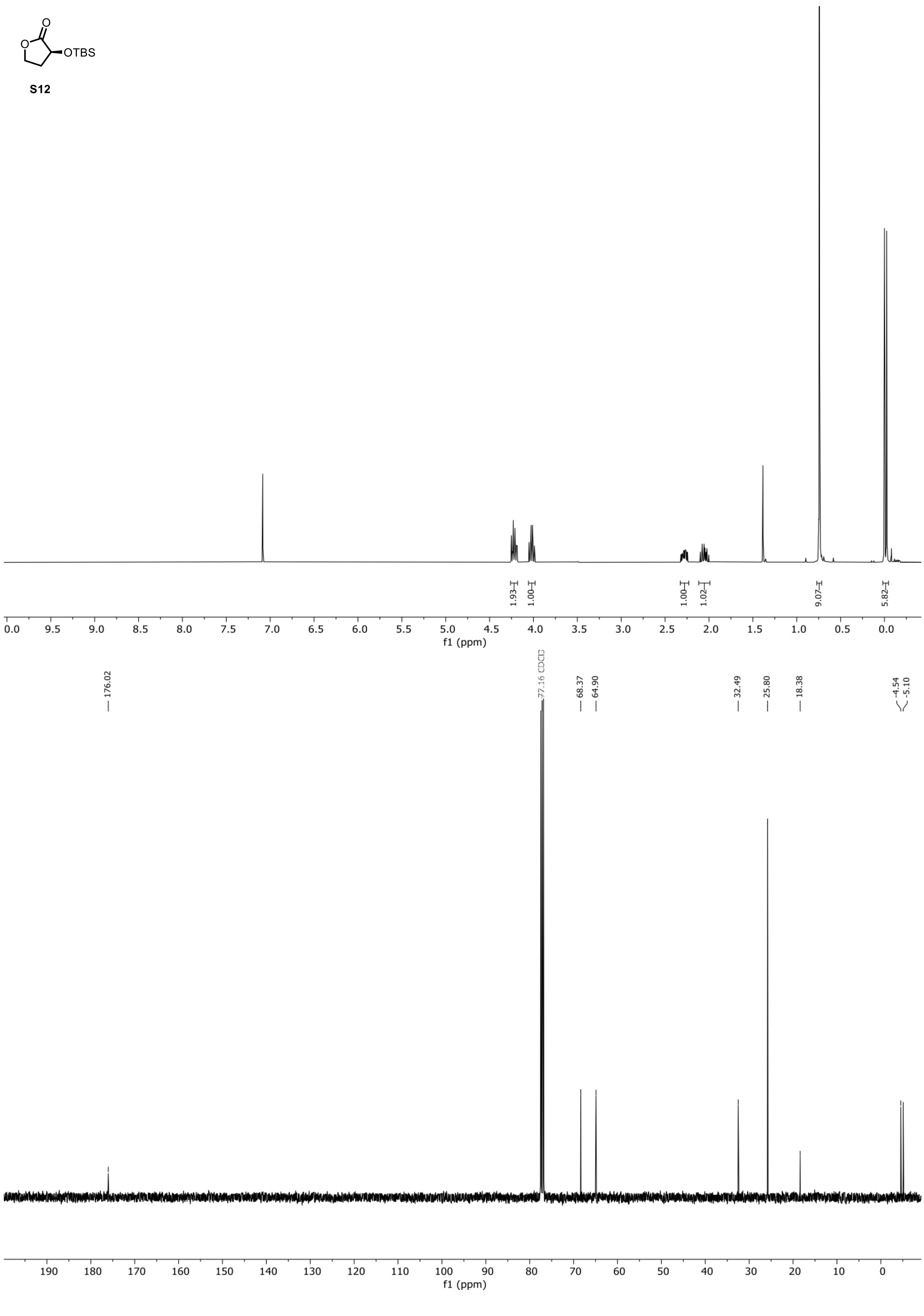

Figure S35. ^{1}H (top) and ^{13}C (bottom) NMR spectra of **S13**

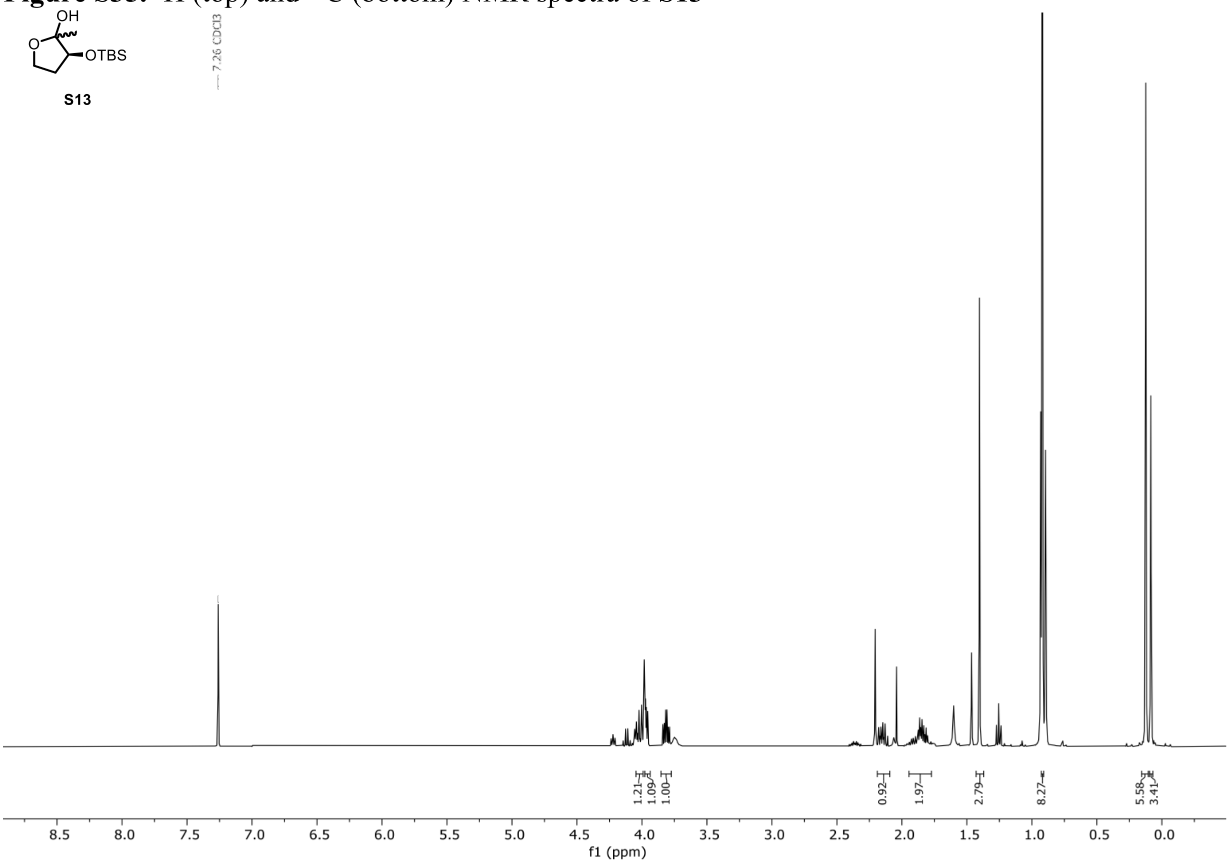

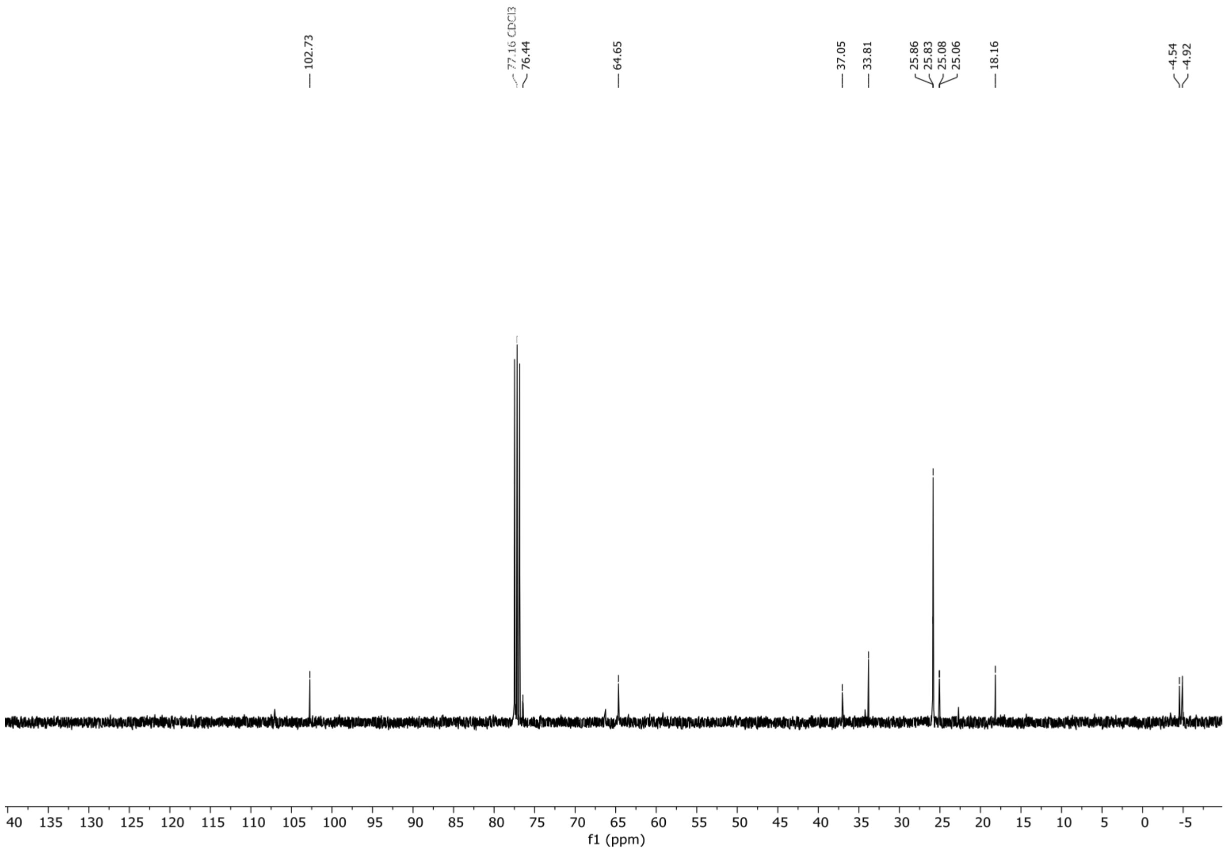

Figure S36. ^{1}H (top) and ^{13}C (bottom) NMR spectra of **S14**

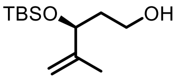

S14

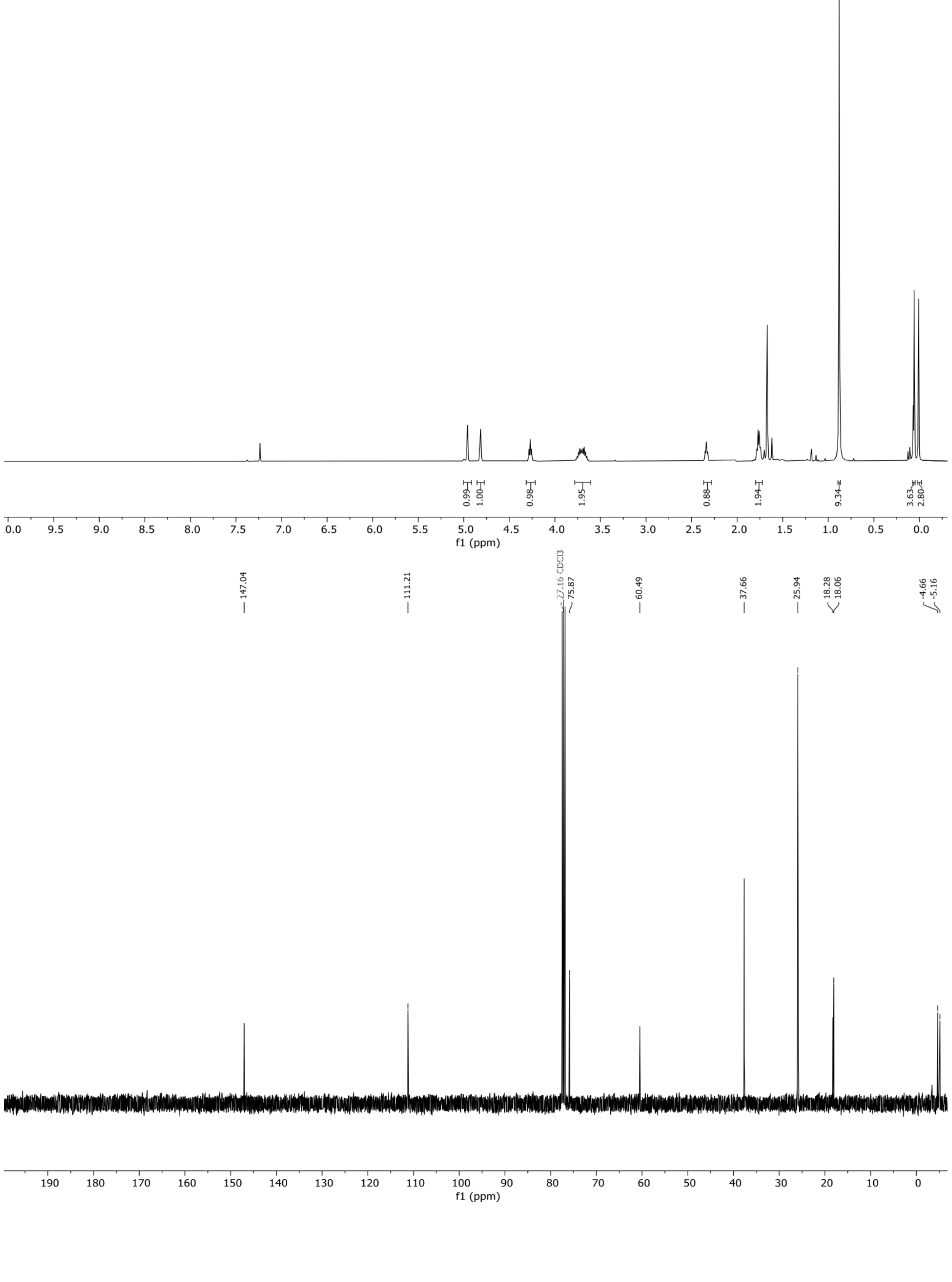

Figure S37. ^{1}H (top) and ^{13}C (bottom) NMR spectra of **S6**

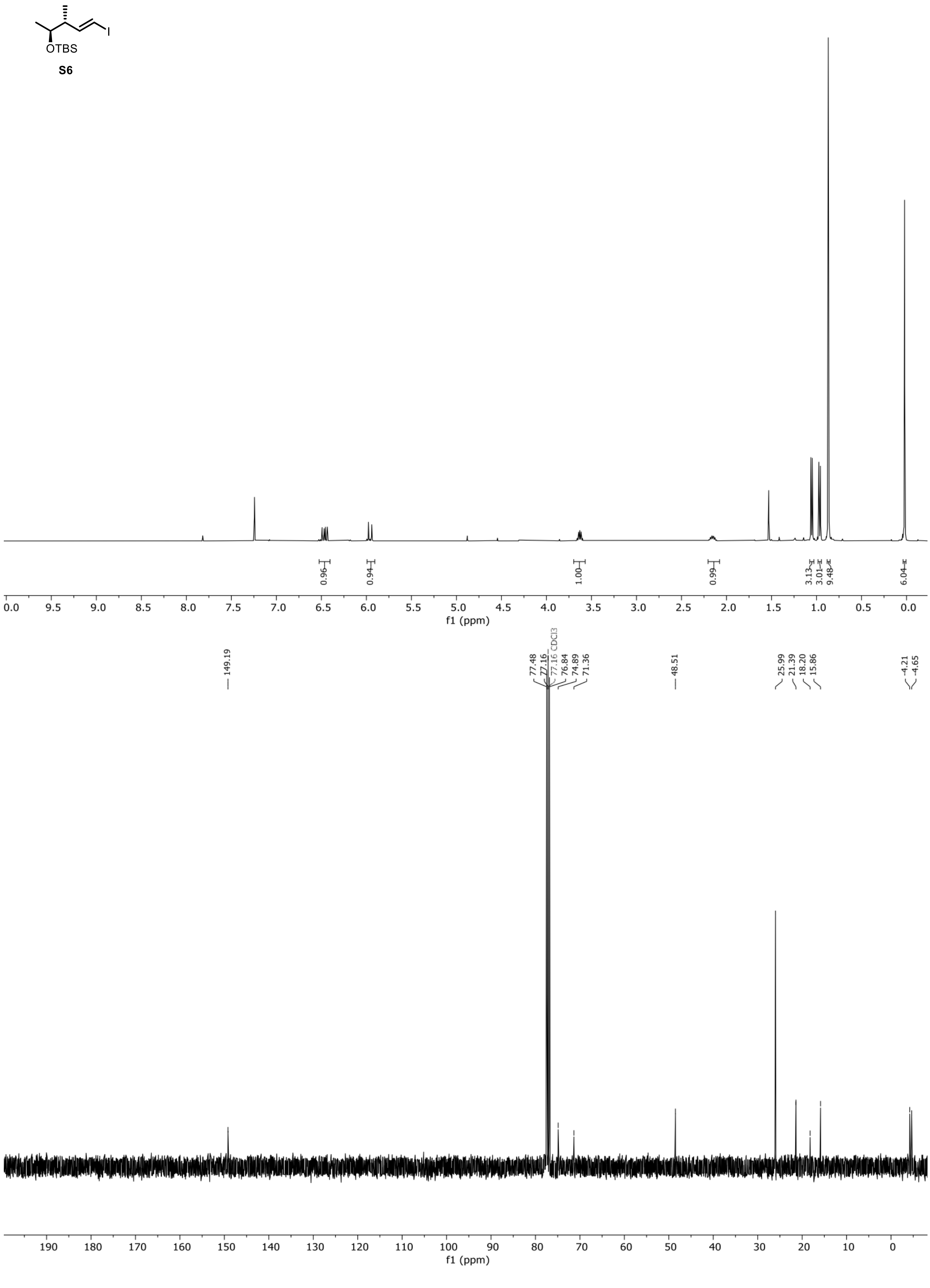

Figure S38. ^{1}H (top) and ^{13}C (bottom) NMR spectra of **S15**

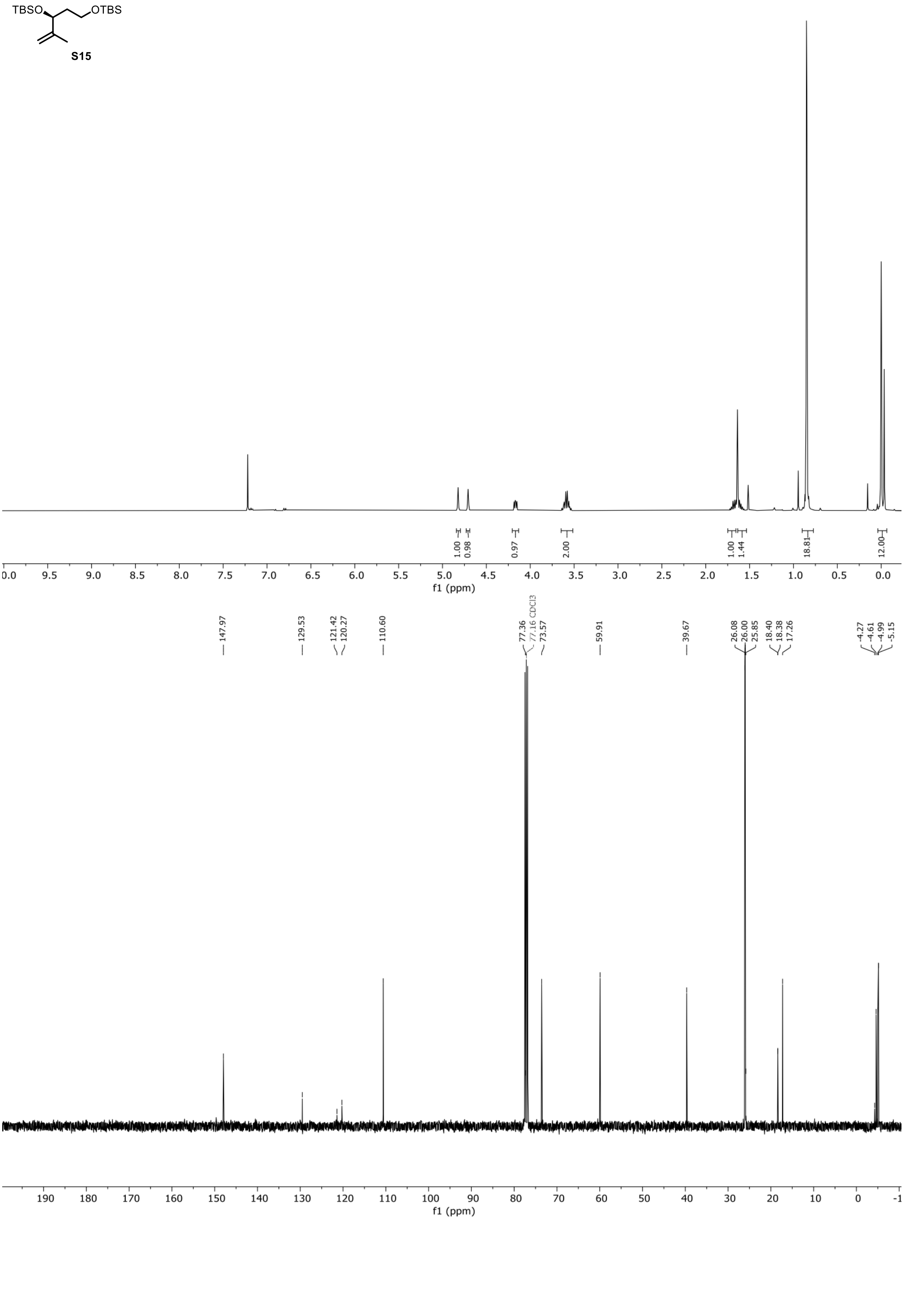

Figure S39. ^{1}H (top) and ^{13}C (bottom) NMR spectra of **S16**

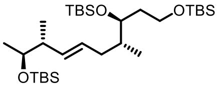

S16

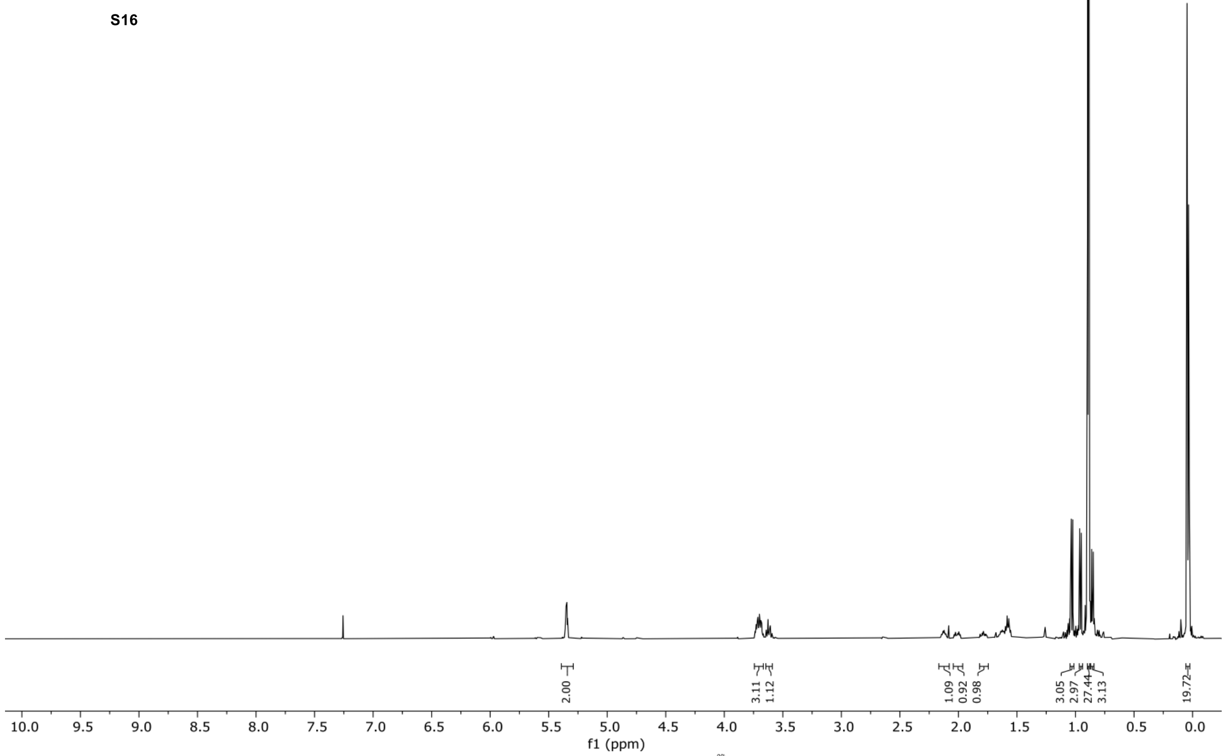

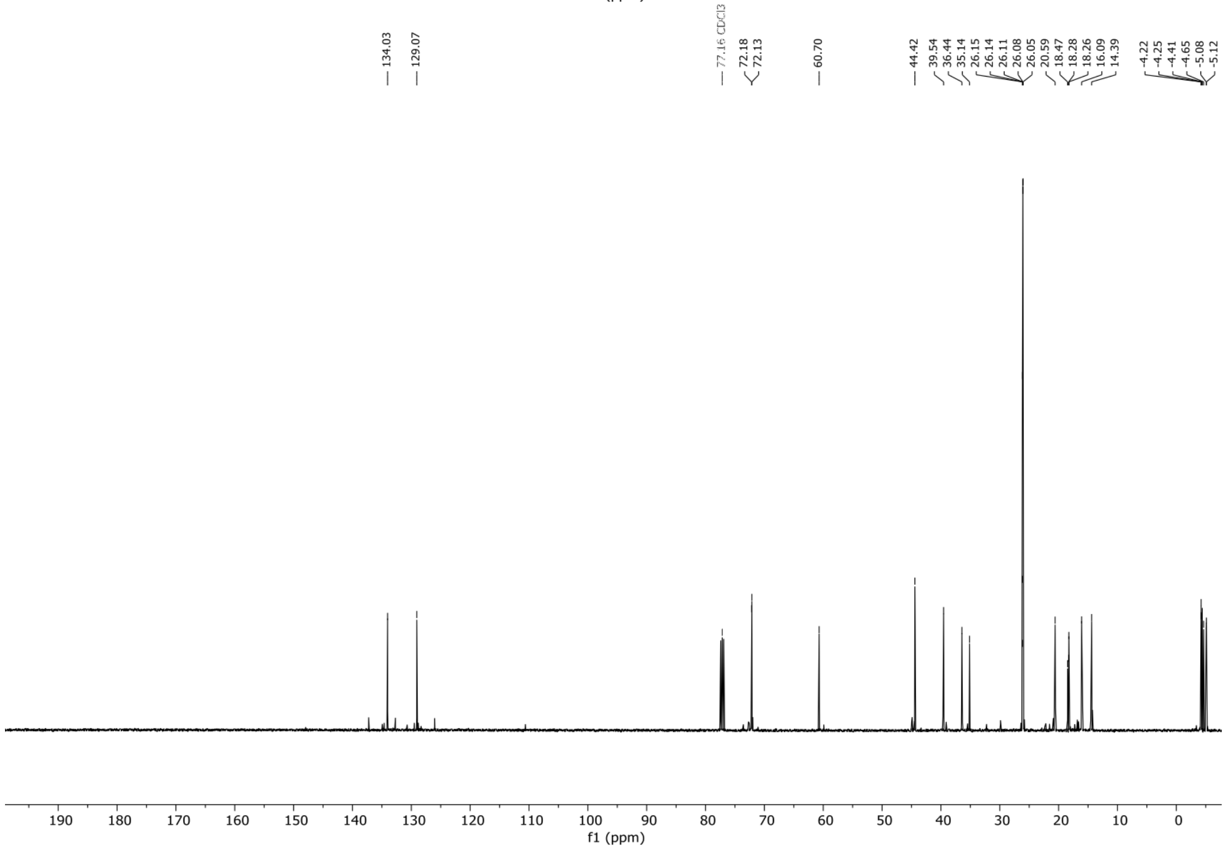

Figure S40. ^{1}H (top) and ^{13}C (bottom) NMR spectra of **S17**

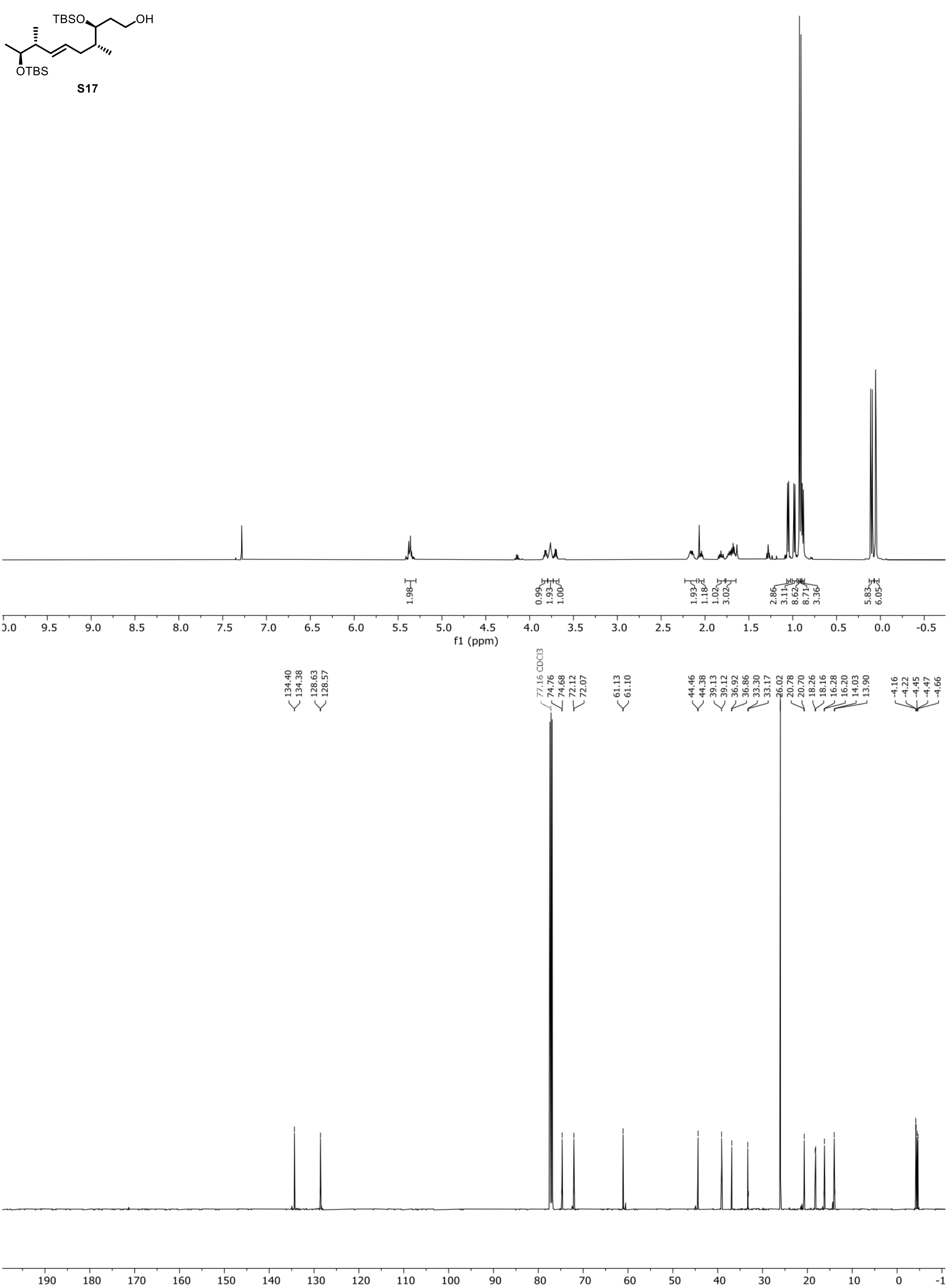

Figure S41. ^{1}H (top) and ^{13}C (bottom) NMR spectra of **S18**

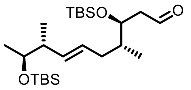

S18

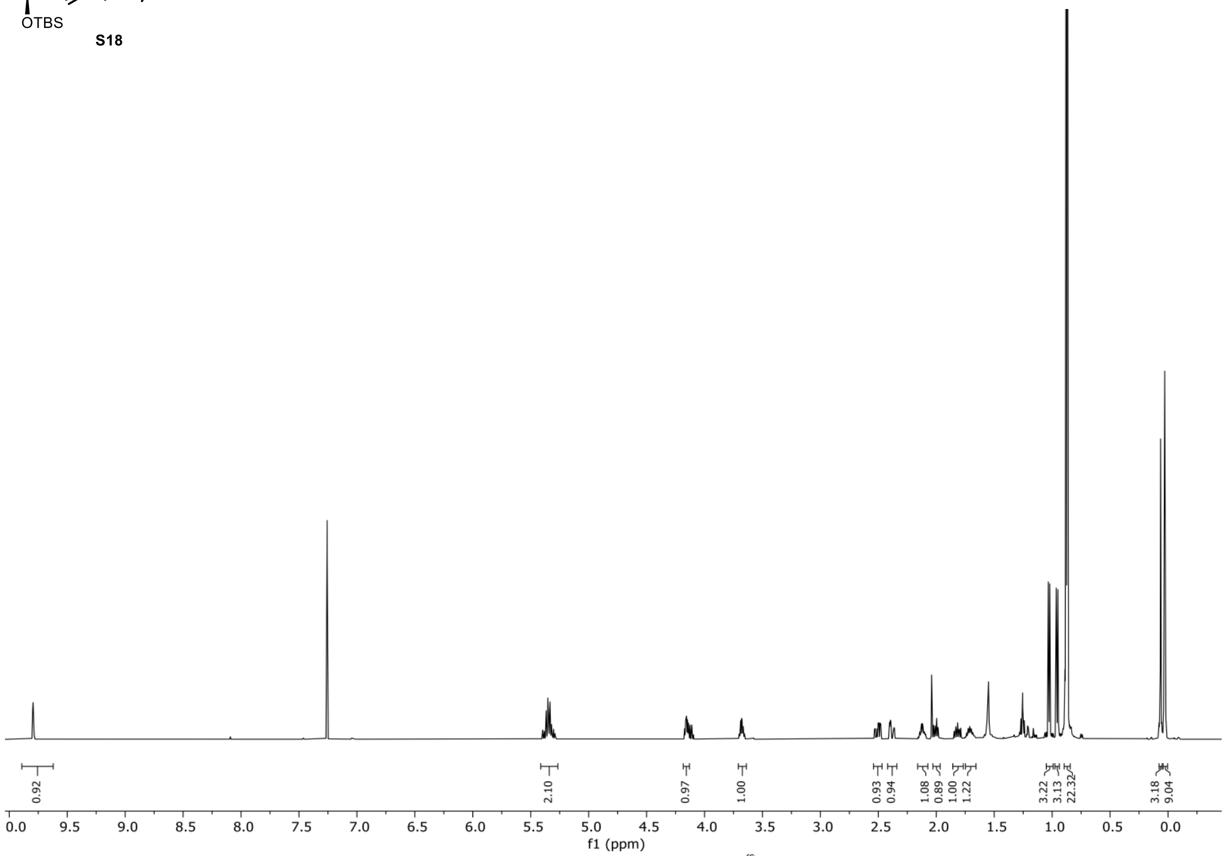

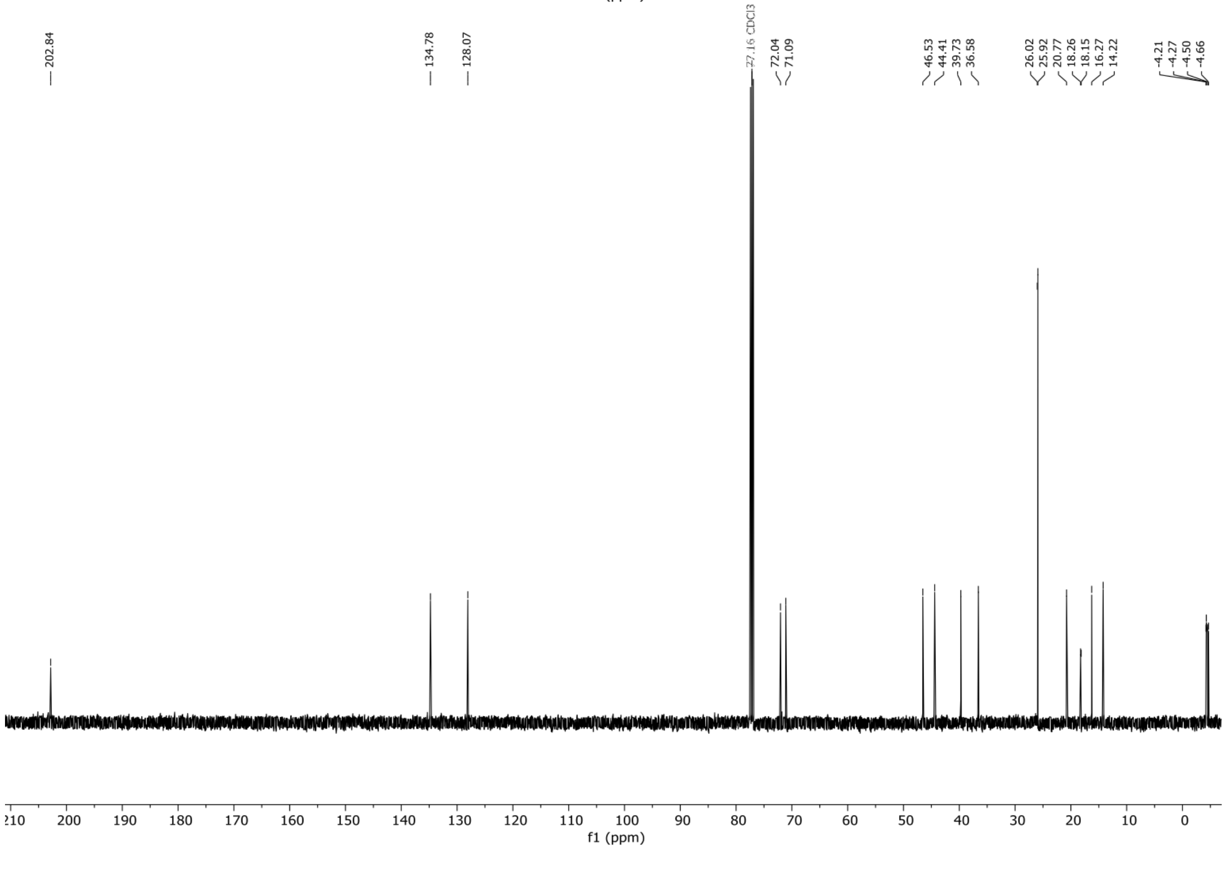

Figure S42. ^{1}H (top) and ^{13}C (bottom) NMR spectra of **S19**

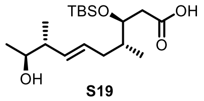

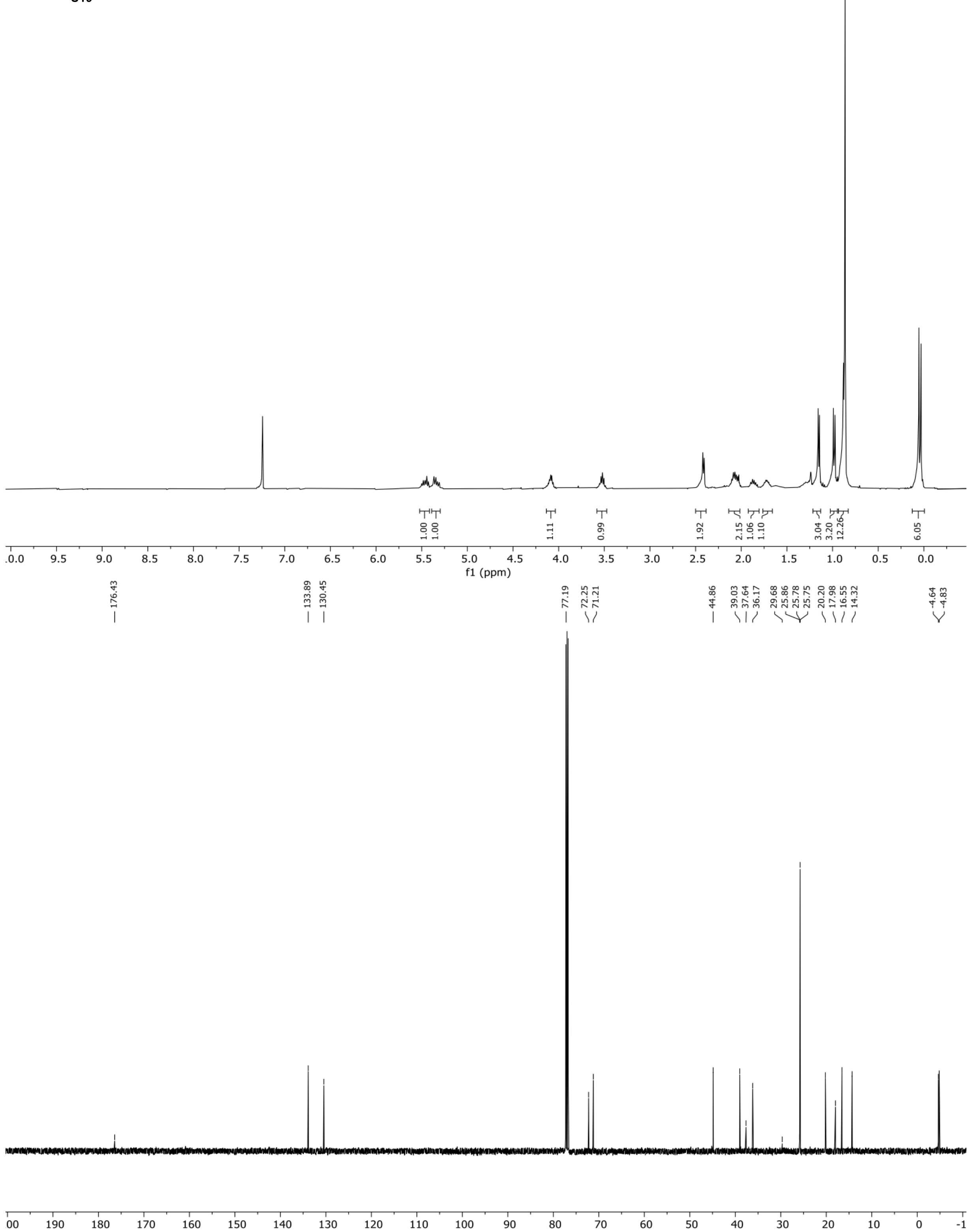

Figure S43. ^{1}H (top) and ^{13}C (bottom) NMR spectra of **12**

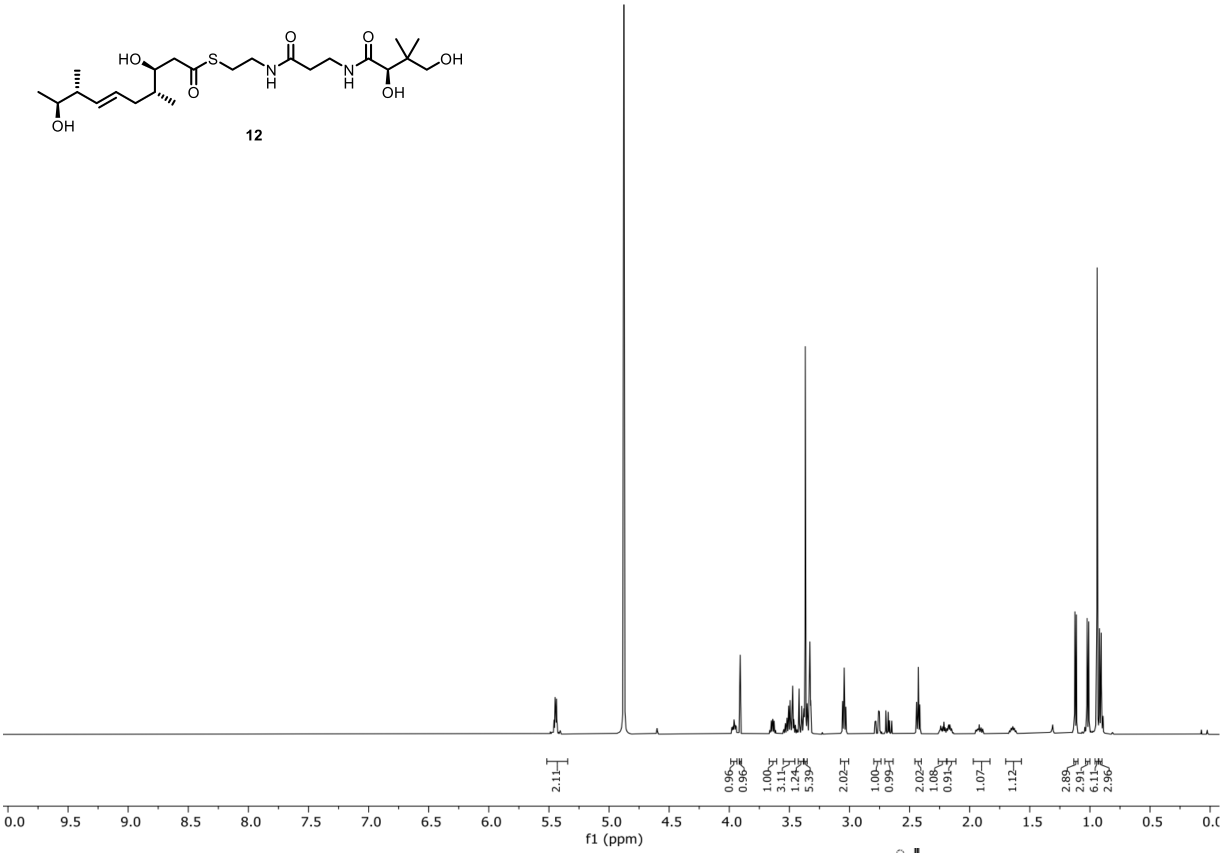

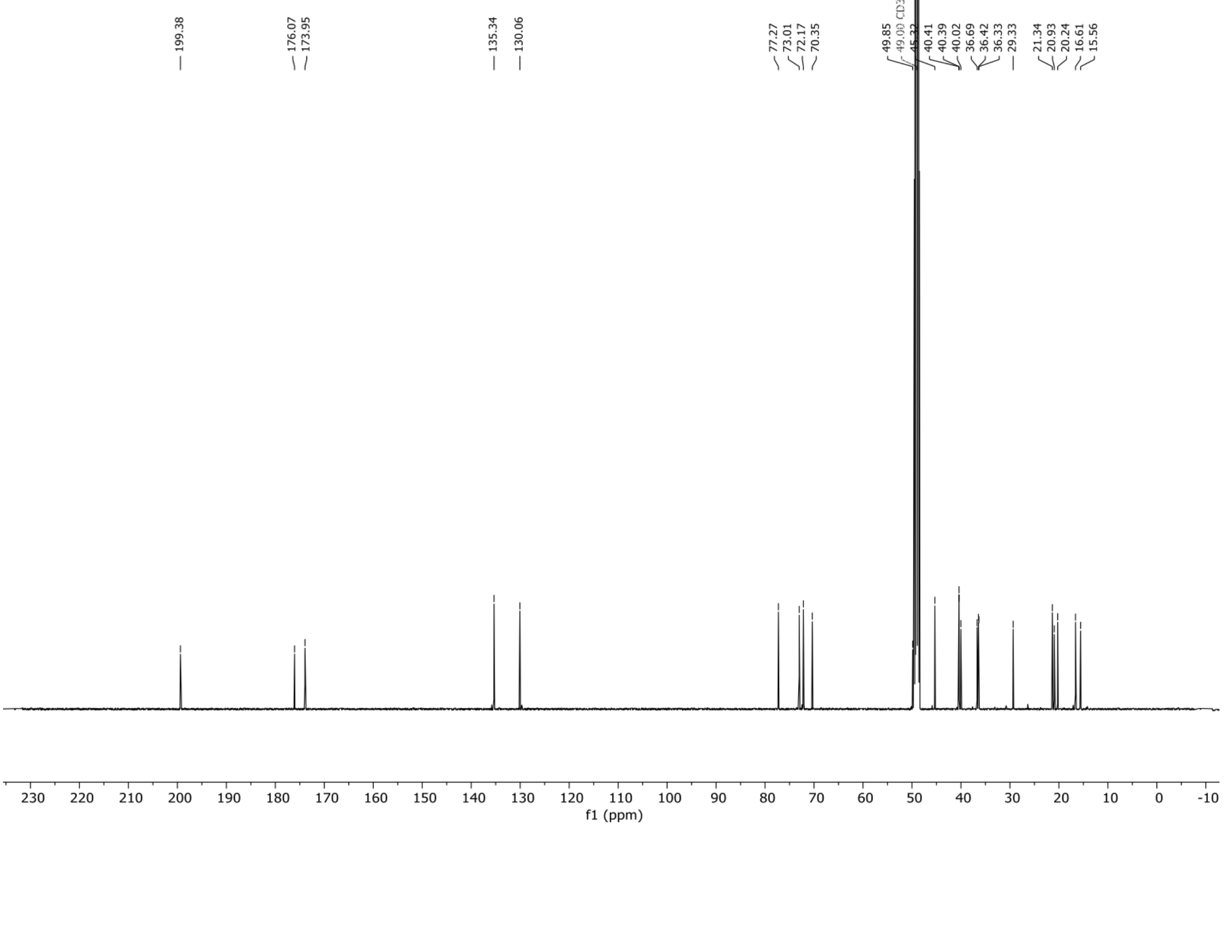

Figure S44. ^{1}H (first), ^{13}C (second), ^{1}H-^{1}H COSY (third), HSQC (fourth) and HMBC (fifth) NMR spectra of Mupirocin A1

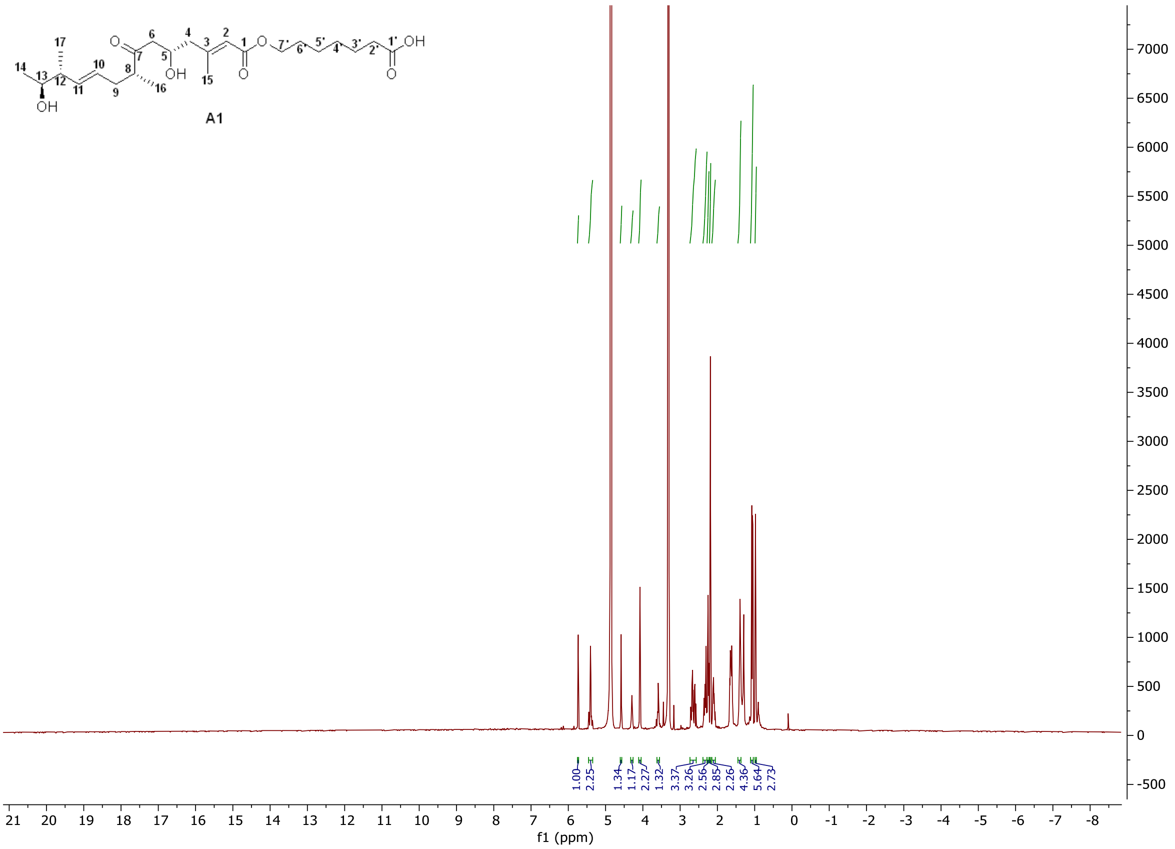

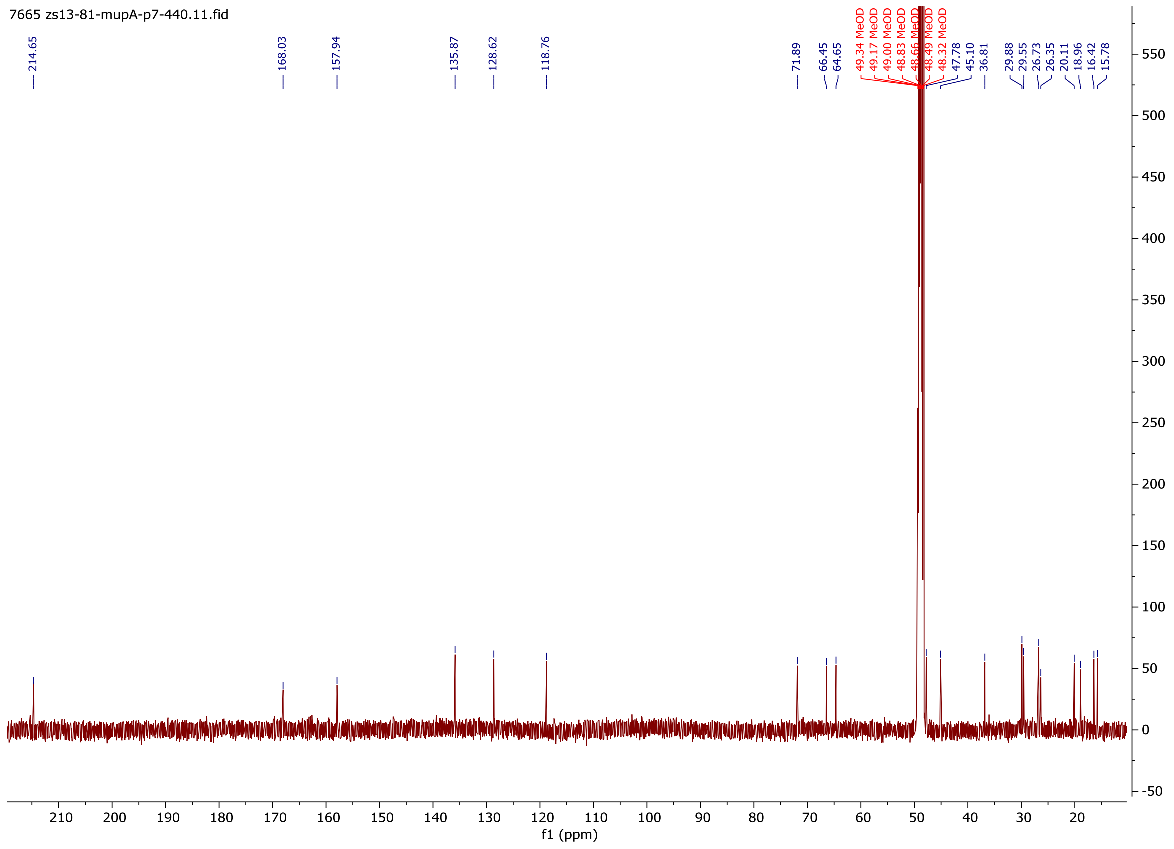
7665 zs13-81-mupA-p7-440.11.fid
214.65
168.03
157.94
135.87
128.62
118.76
71.89
66.45
64.65
49.34 MeOD
49.17 MeOD
49.00 MeOD
48.83 MeOD
48.66 MeOD
48.49 MeOD
48.32 MeOD
47.78
45.10
36.81
29.88
29.55
26.73
26.35
20.11
18.96
16.42
15.78
f1 (ppm)

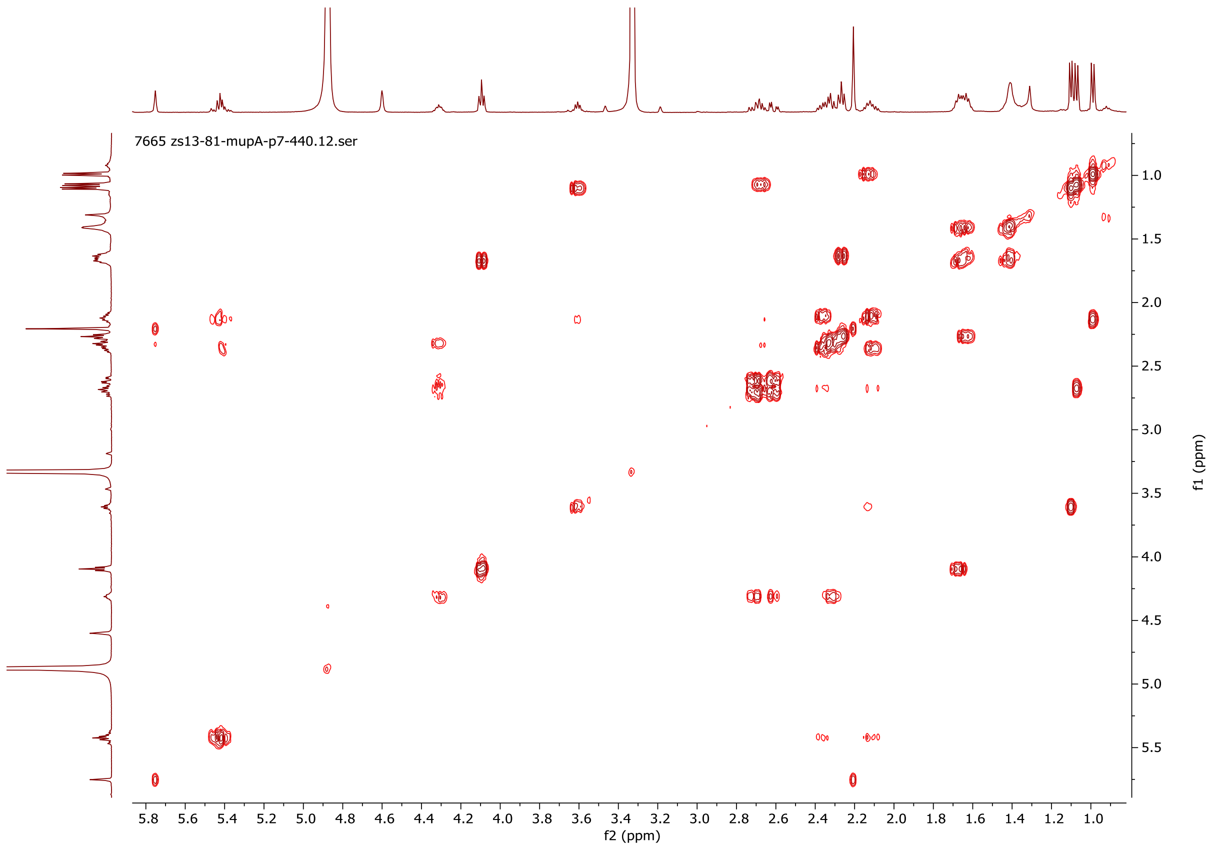
7665 zs13-81-mupA-p7-440.12.ser
f2 (ppm)
f1 (ppm)

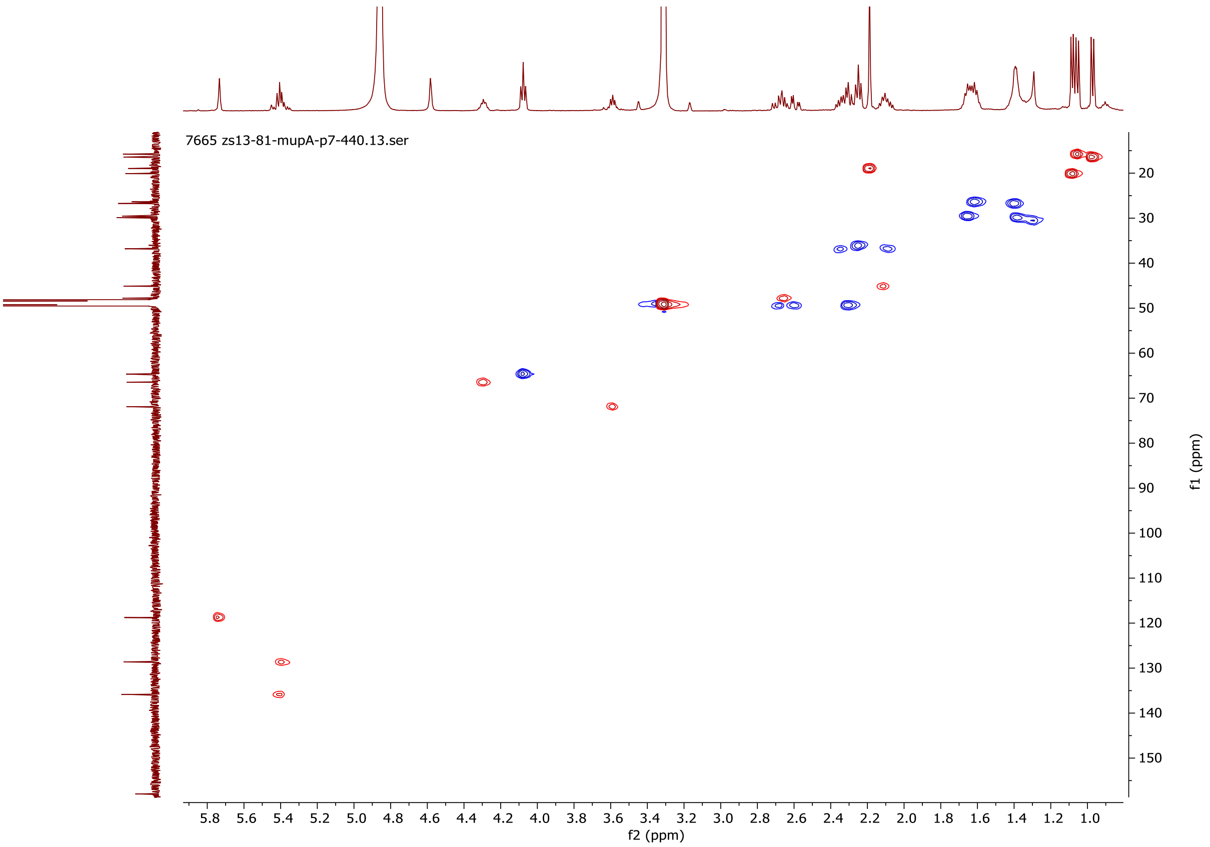
7665 zs13-81-mupA-p7-440.13.ser
f2 (ppm)
f1 (ppm)

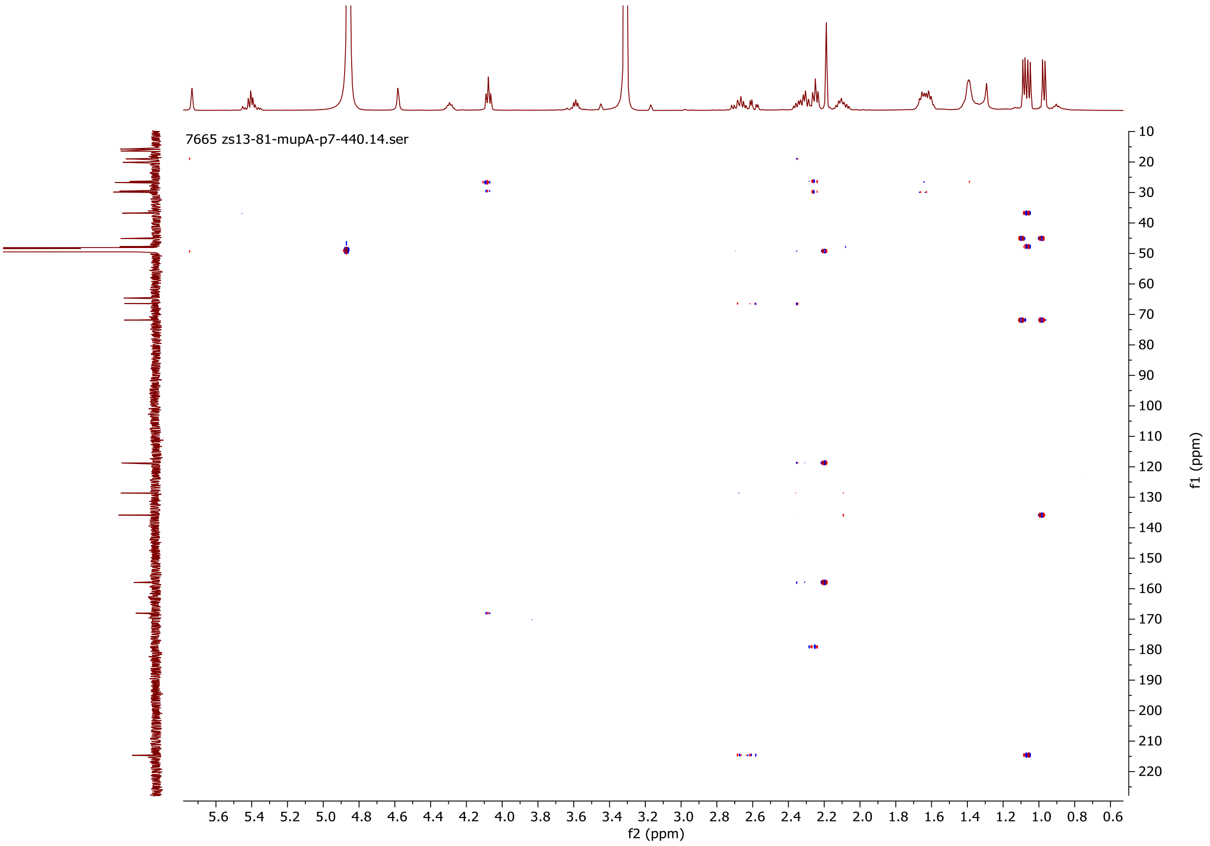
7665 zs13-81-mupA-p7-440.14.ser
f2 (ppm)
f1 (ppm)

Figure S45. ^{1}H (first), ^{1}H-^{1}H COSY (second) NMR spectra of Mupirocin A2

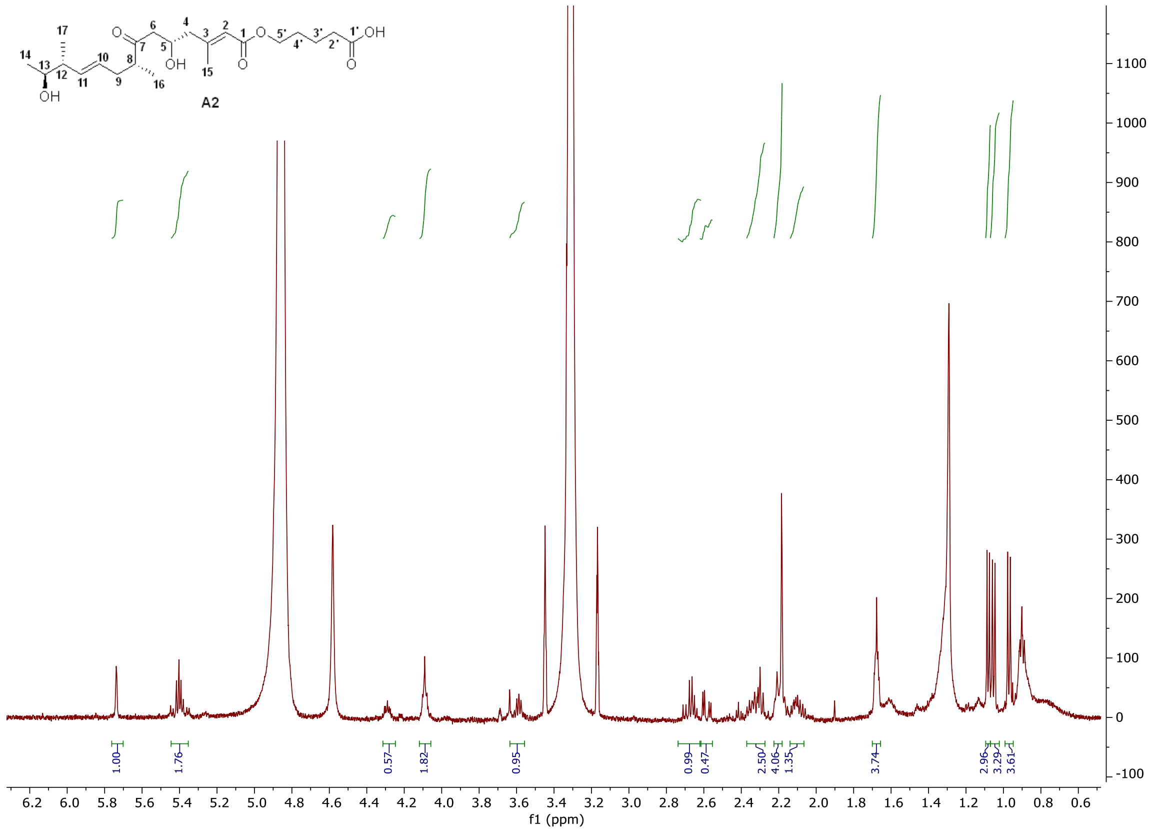

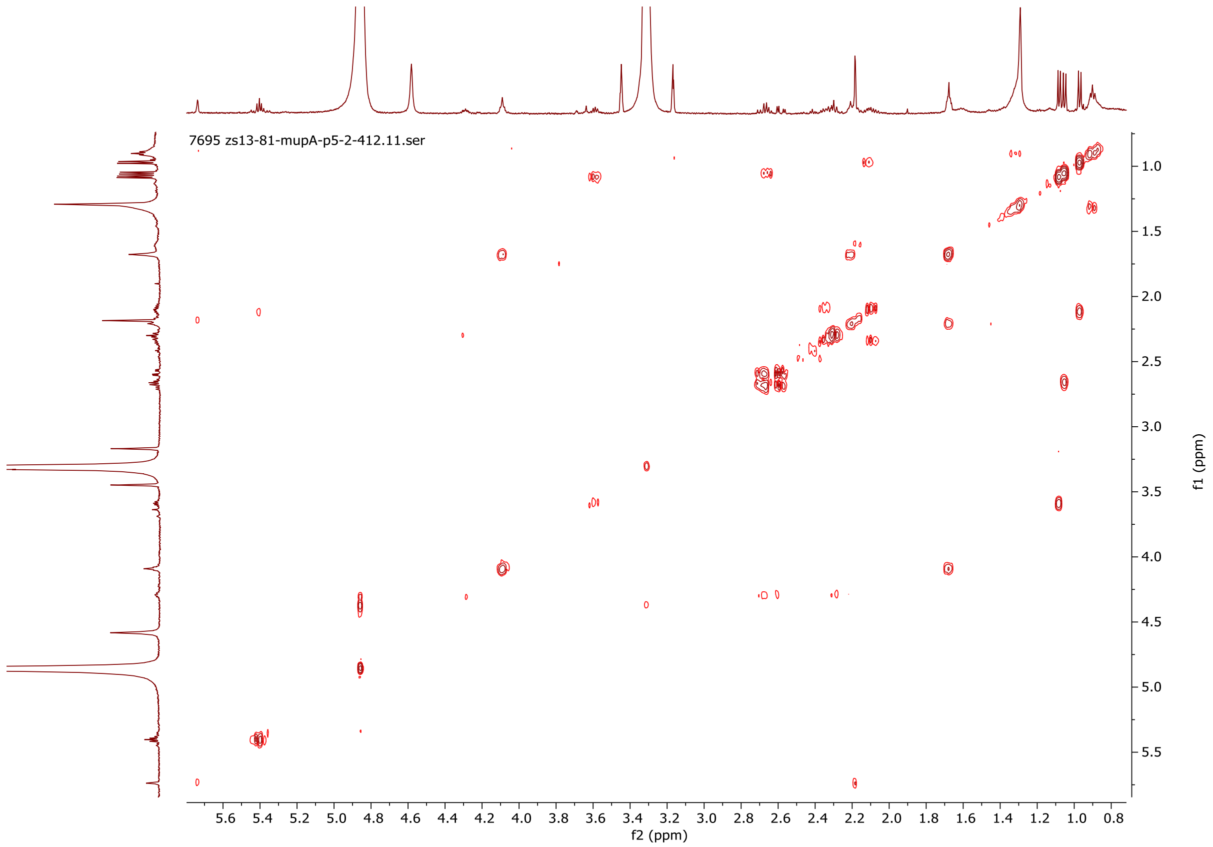

Figure S46. ^{1}H (first), ^{1}H-^{1}H COSY (second), HSQC (third) and HMBC (fourth) NMR spectra of Mupirocin A3

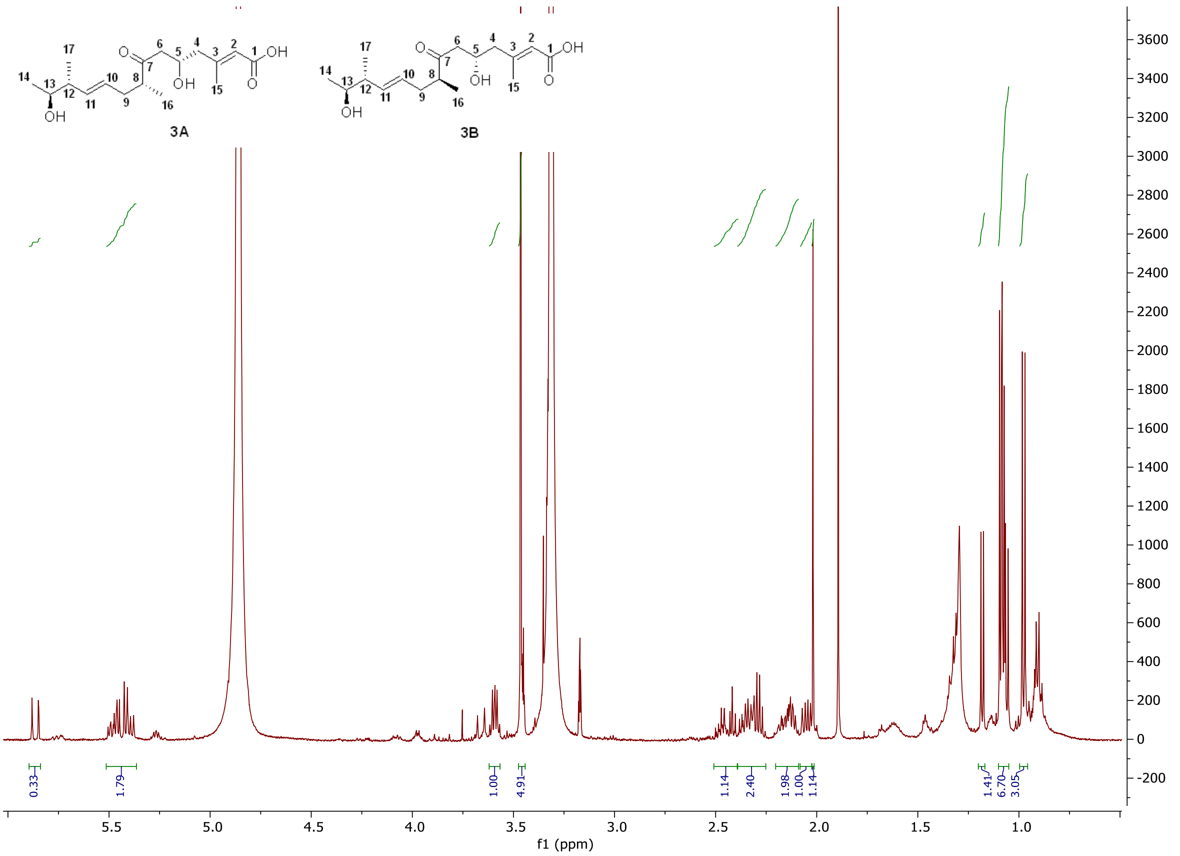

3A
3B
f1 (ppm)

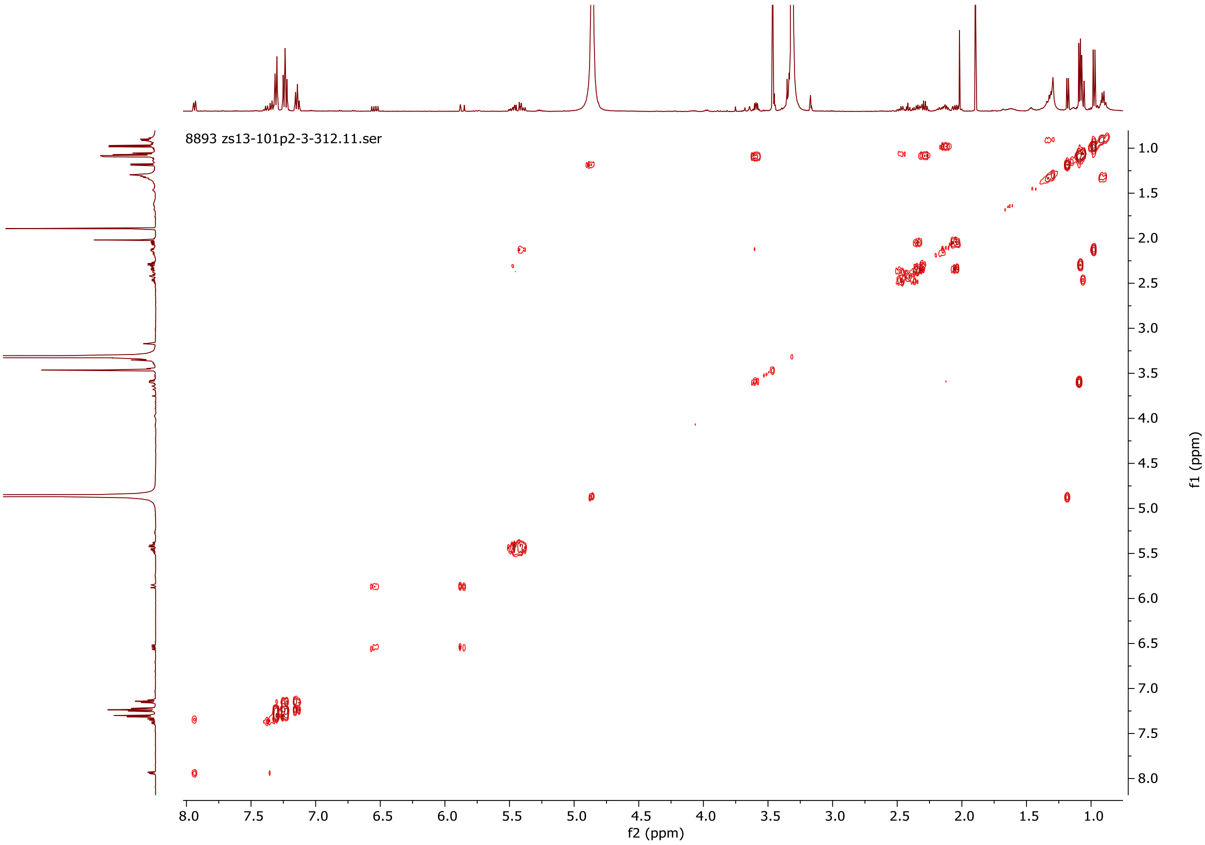

8893 zs13-101p2-3-312.11.ser
f2 (ppm)
f1 (ppm)

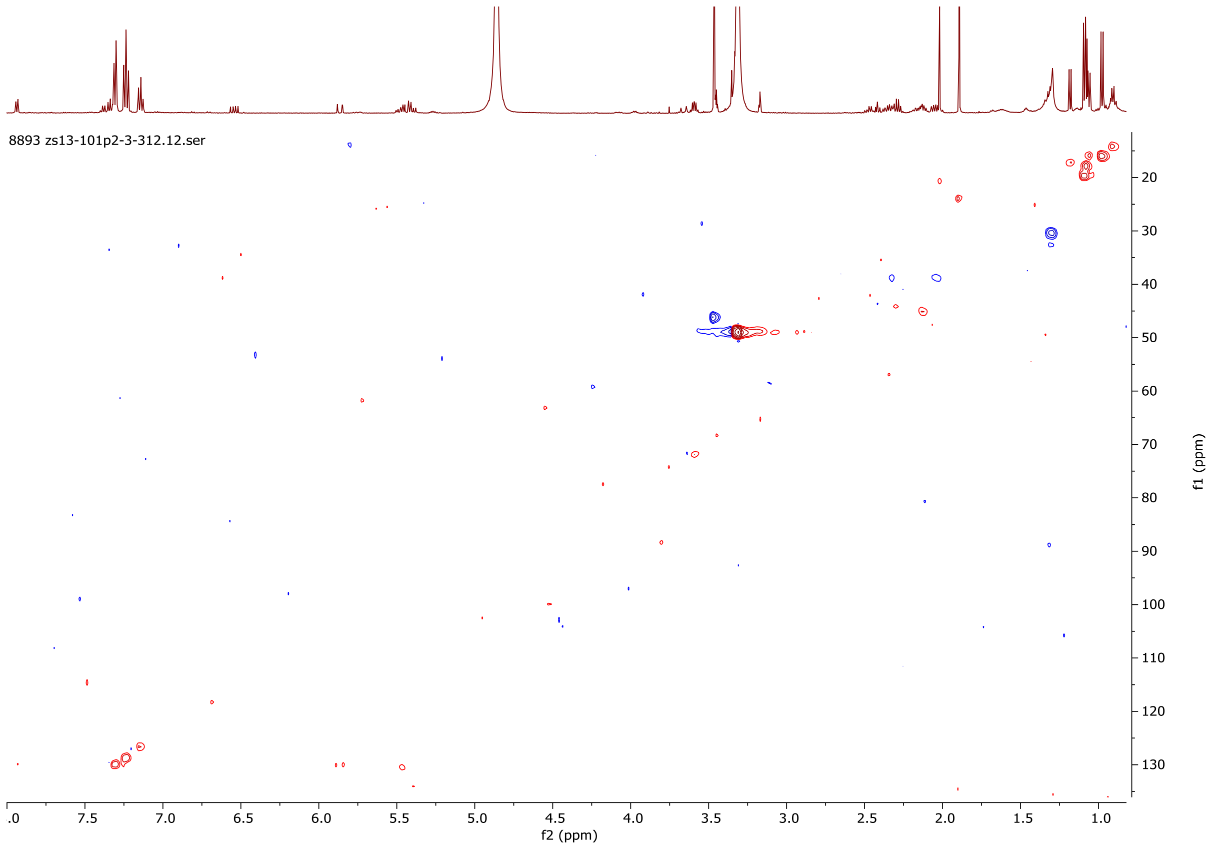
8893 zs13-101p2-3-312.12.ser
f2 (ppm)
f1 (ppm)

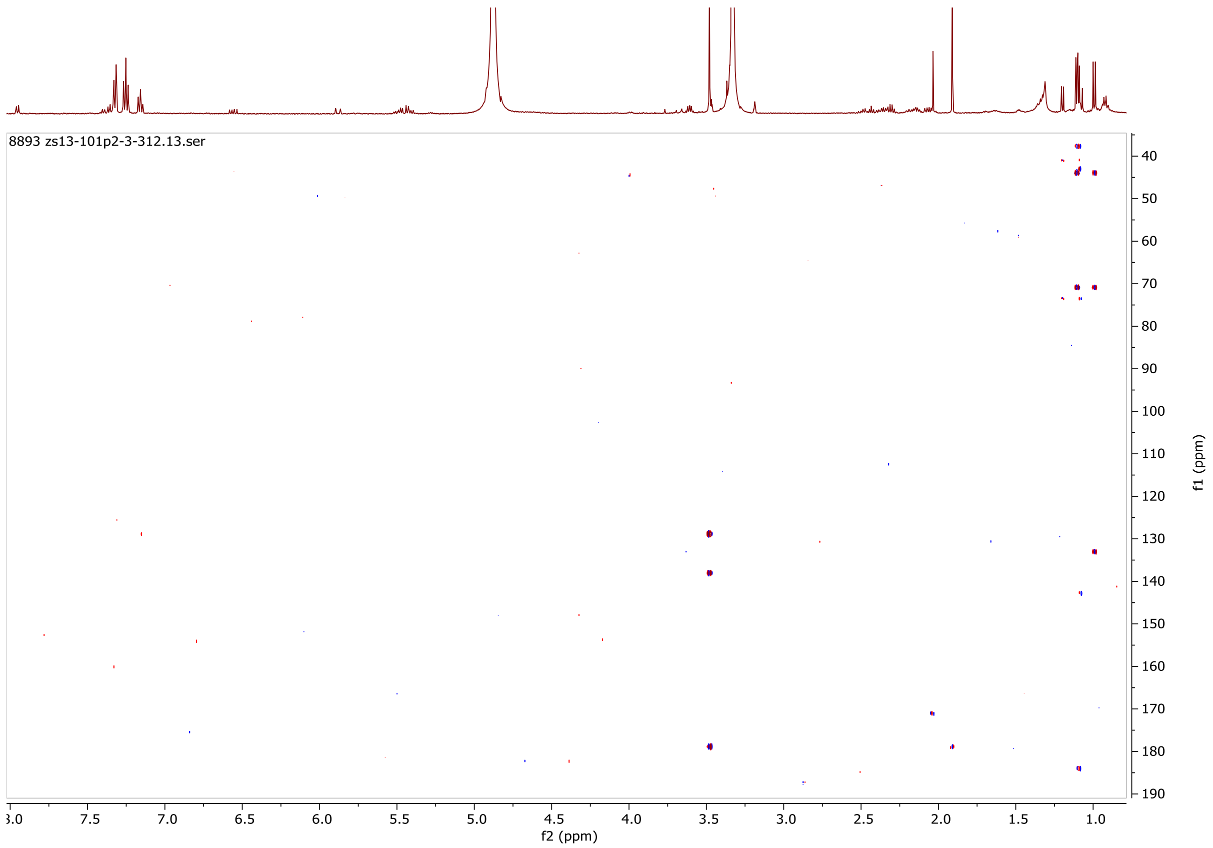
8893 zs13-101p2-3-312.13.ser
f2 (ppm)
f1 (ppm)

Figure S47. ^{1}H (first), ^{1}H-^{1}H COSY (second), HSQC (third) and HMBC (fourth) NMR spectra of Mupirocin A4

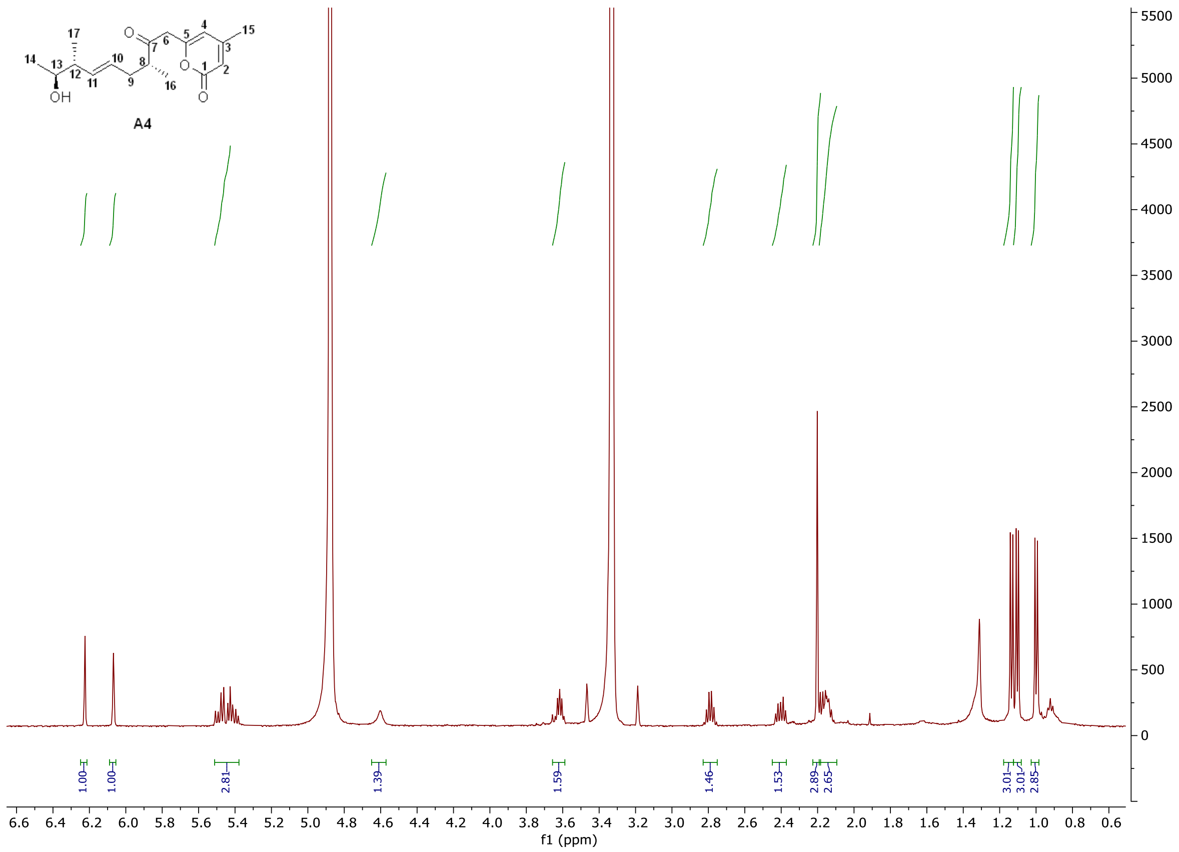

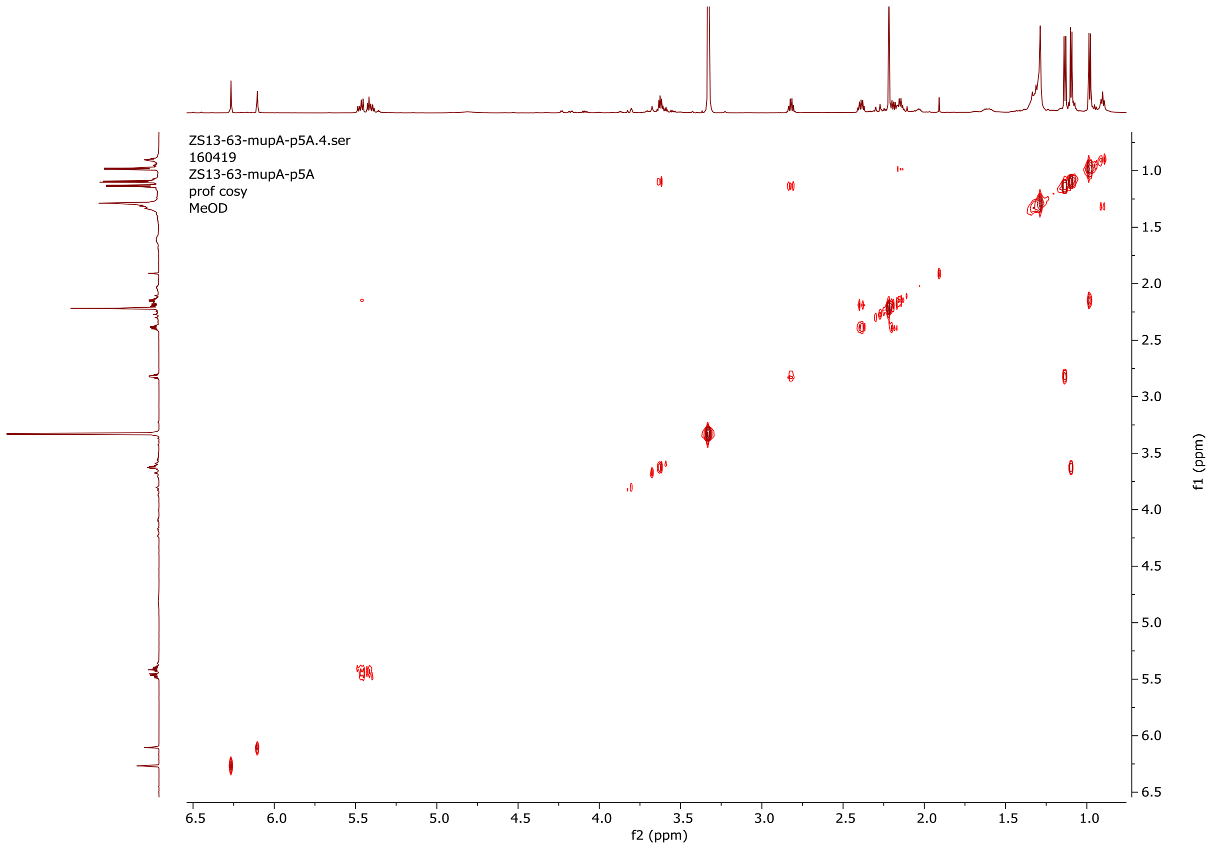
ZS13-63-mupA-p5A.4.ser
160419
ZS13-63-mupA-p5A
prof cosy
MeOD
f2 (ppm)
f1 (ppm)

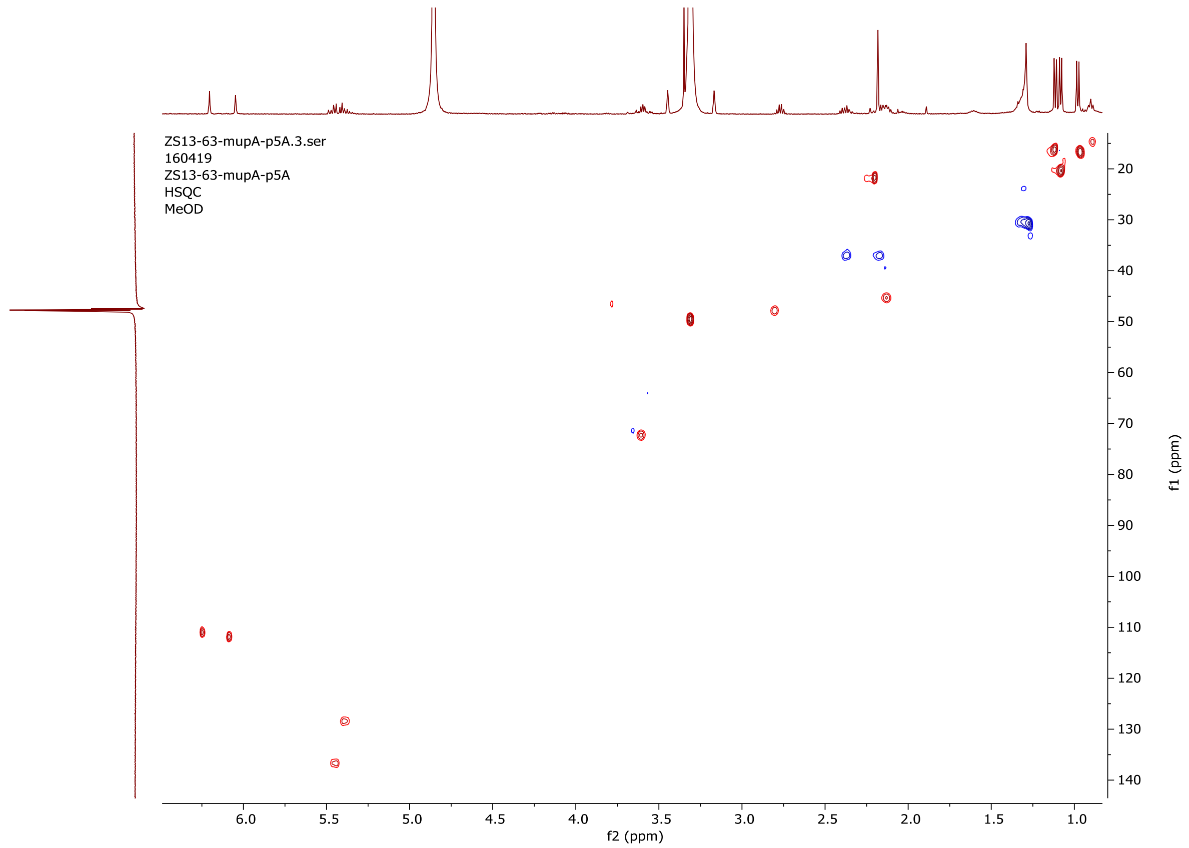
ZS13-63-mupA-p5A.3.ser
160419
ZS13-63-mupA-p5A
HSQC
MeOD
f2 (ppm)
f1 (ppm)

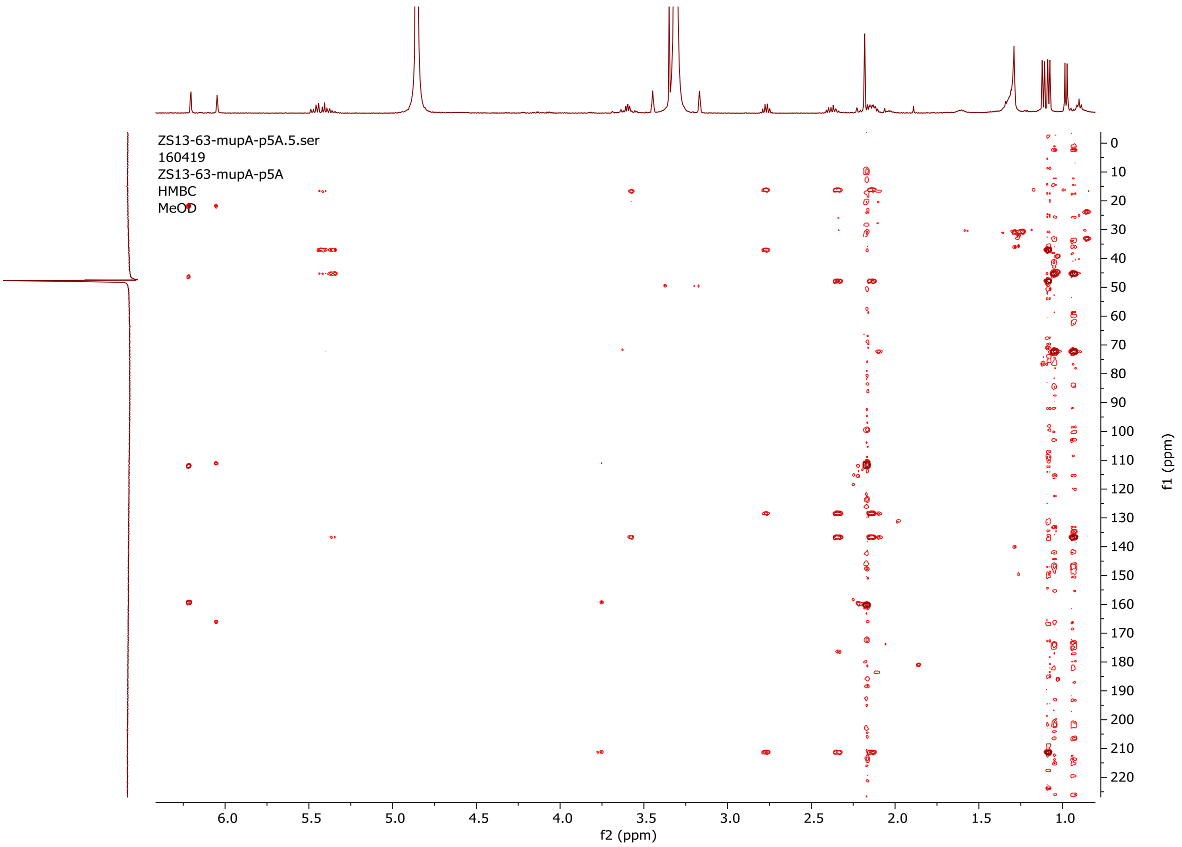
ZS13-63-mupA-p5A.5.ser
160419
ZS13-63-mupA-p5A
HMBC
MeOD
f2 (ppm)
f1 (ppm)

Figure S48. ^{1}H (first), ^{13}C (second) ^{1}H-^{1}H COSY (third), HSQC (fourth) and HMBC (fifth) NMR spectra of Mupirocin A5

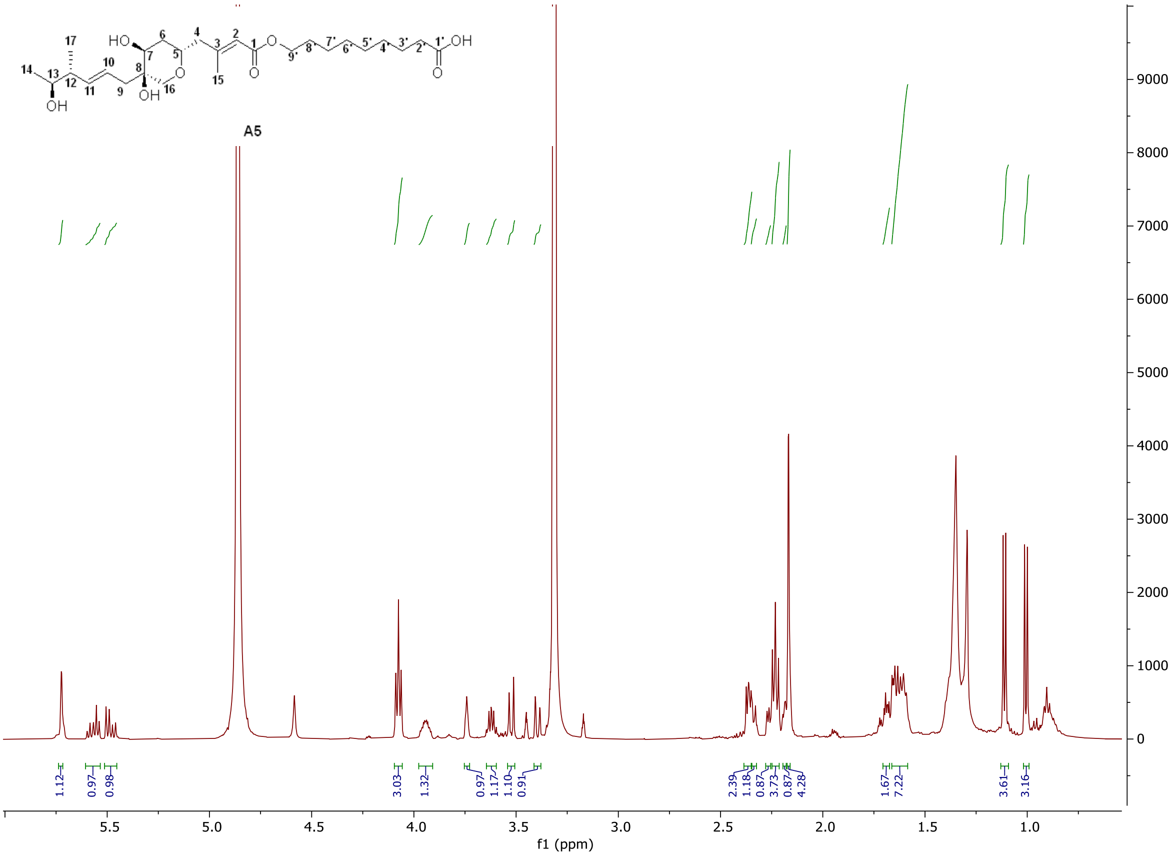

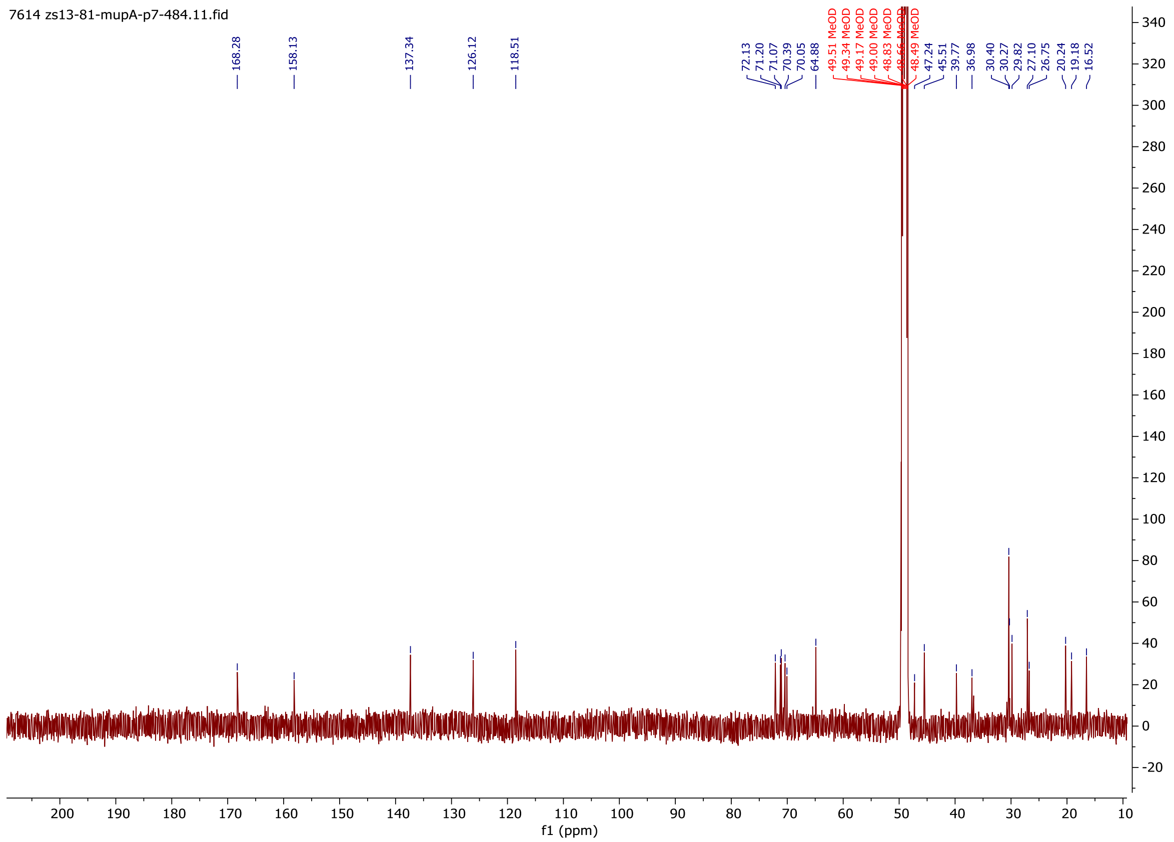
7614 zs13-81-mupA-p7-484.11.fid
168.28
158.13
137.34
126.12
118.51
72.13
71.20
71.07
70.39
70.05
64.88
49.51 MeOD
49.34 MeOD
49.17 MeOD
49.00 MeOD
48.83 MeOD
48.66 MeOD
48.49 MeOD
47.24
45.51
39.77
36.98
30.40
30.27
29.82
27.10
26.75
20.24
19.18
16.52
f1 (ppm)

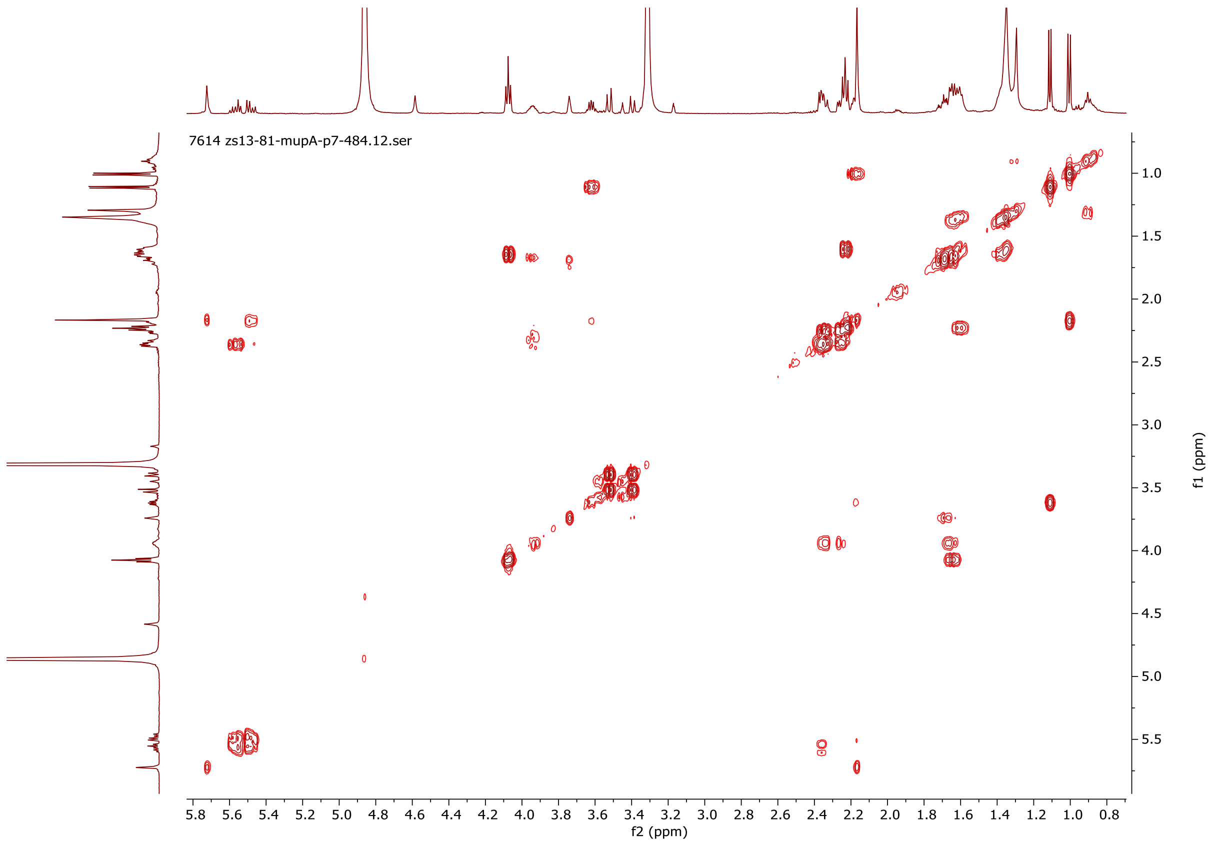
7614 zs13-81-mupA-p7-484.12.ser
f2 (ppm)
f1 (ppm)

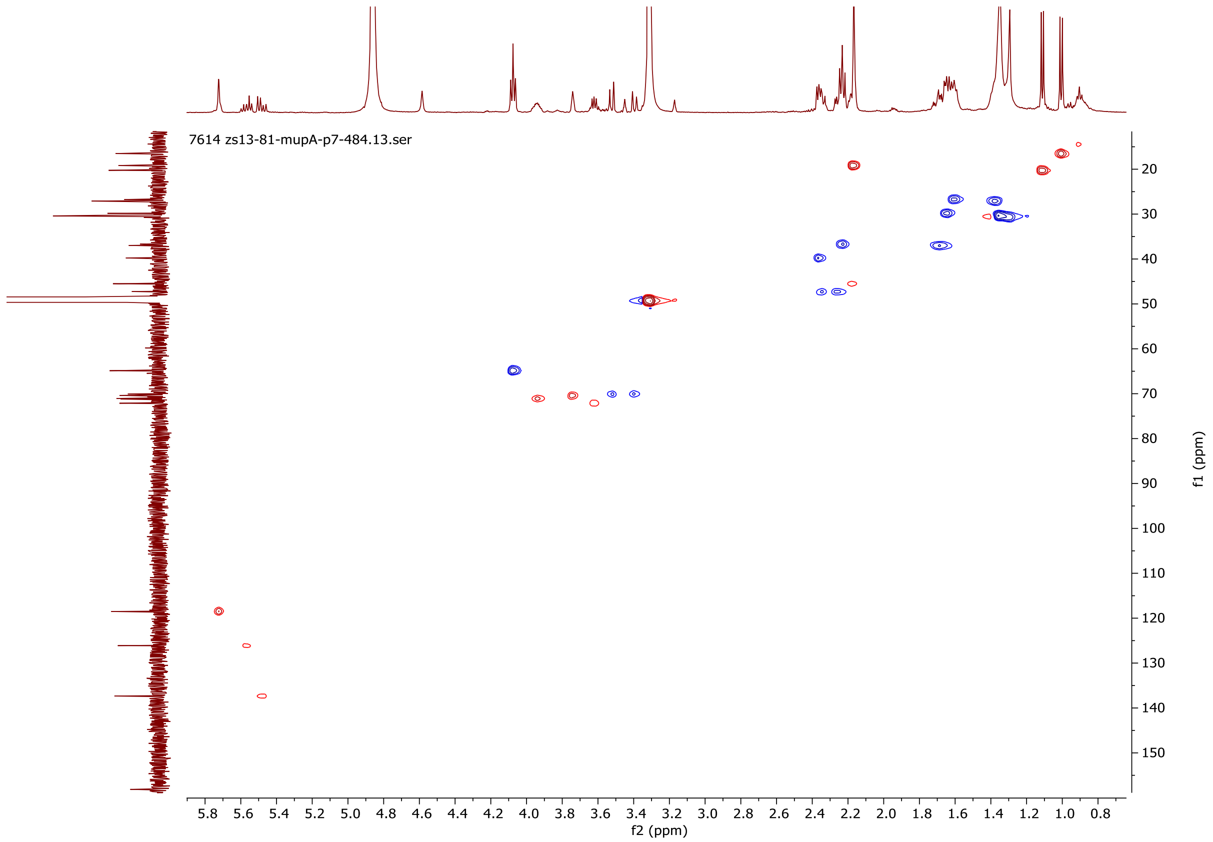
7614 zs13-81-mupA-p7-484.13.ser
f2 (ppm)
f1 (ppm)

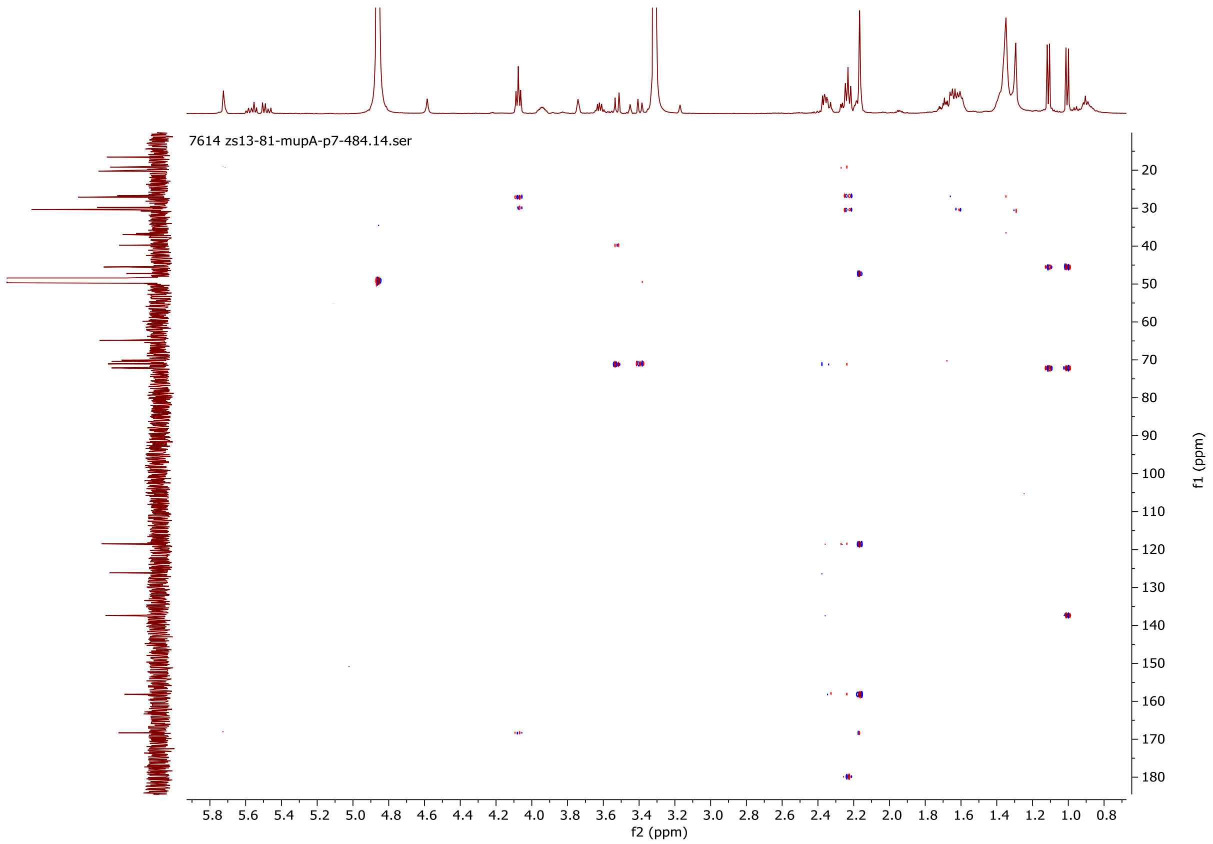
7614 zs13-81-mupA-p7-484.14.ser
f2 (ppm)
f1 (ppm)

Figure S49. ^{1}H (first), ^{13}C (second) ^{1}H-^{1}H COSY (third), HSQC (fourth) and HMBC (fifth) NMR spectra of Mupirocin A6

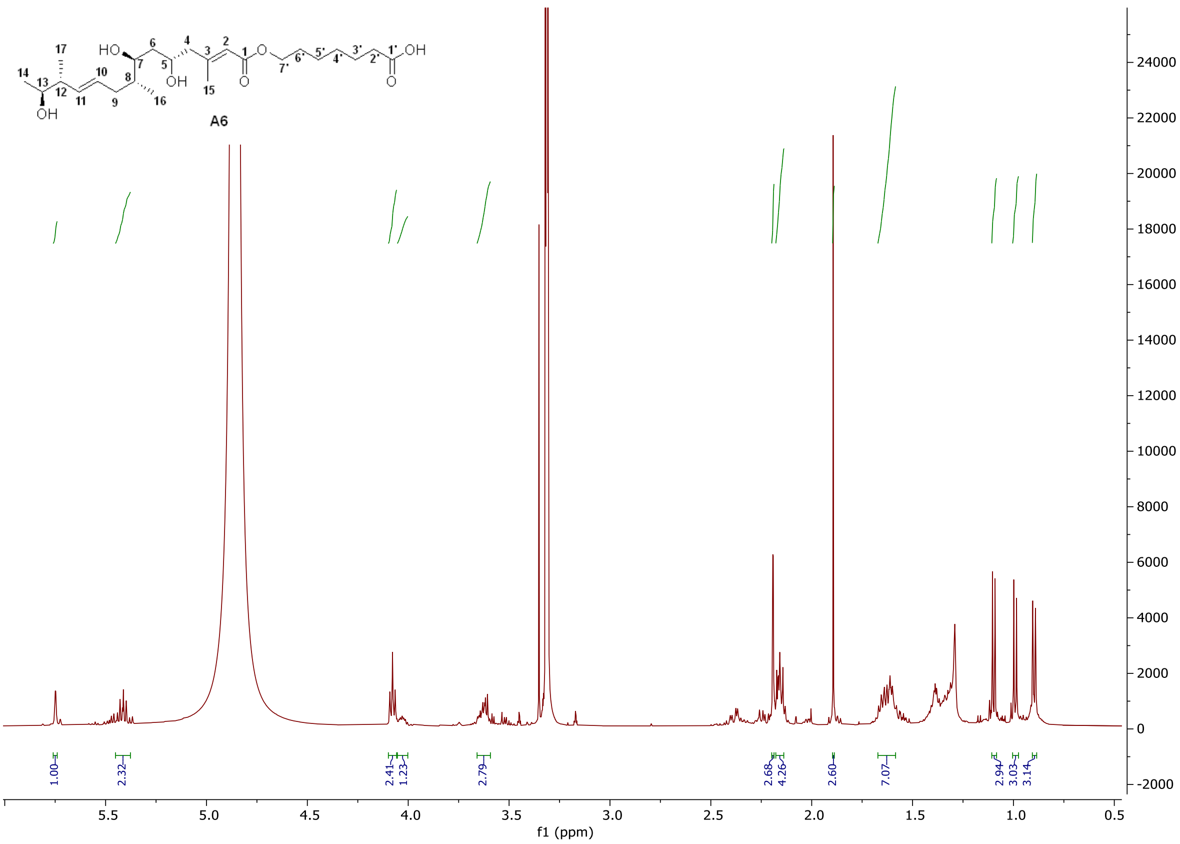

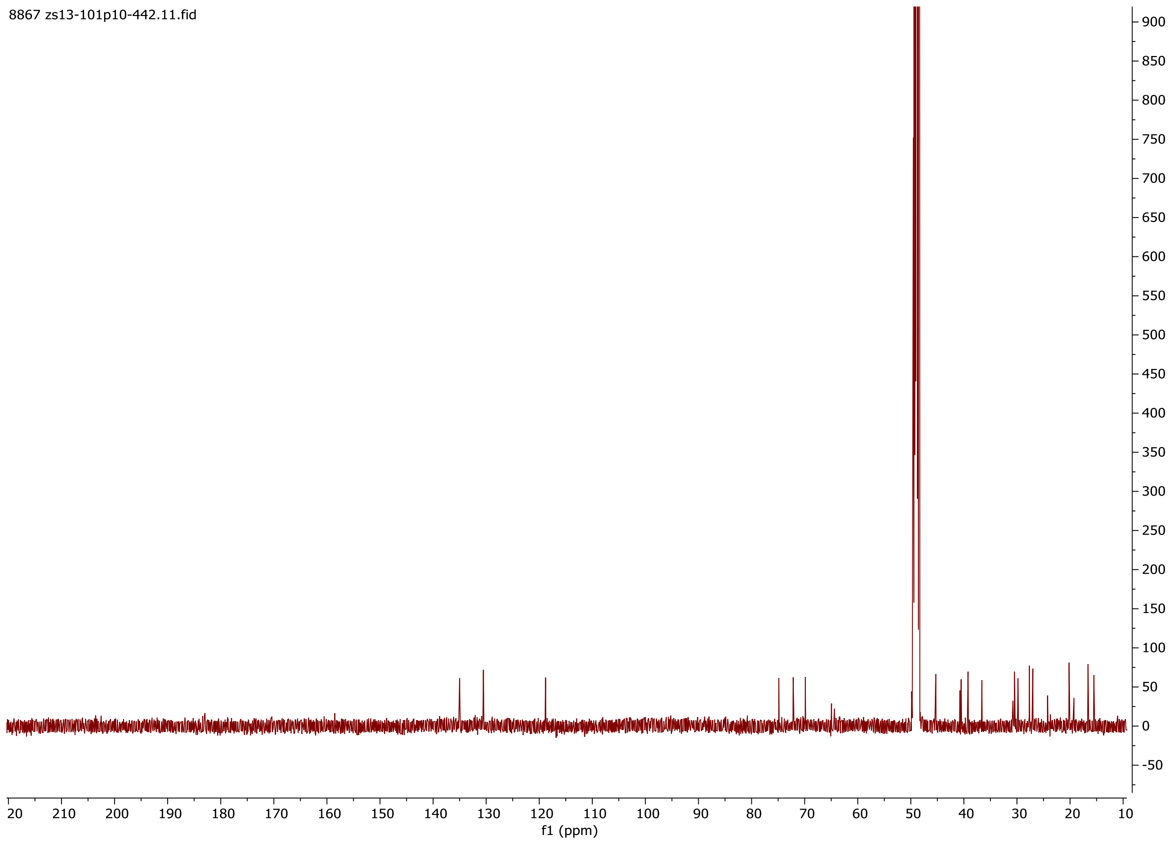
8867 zs13-101p10-442.11.fid
f1 (ppm)

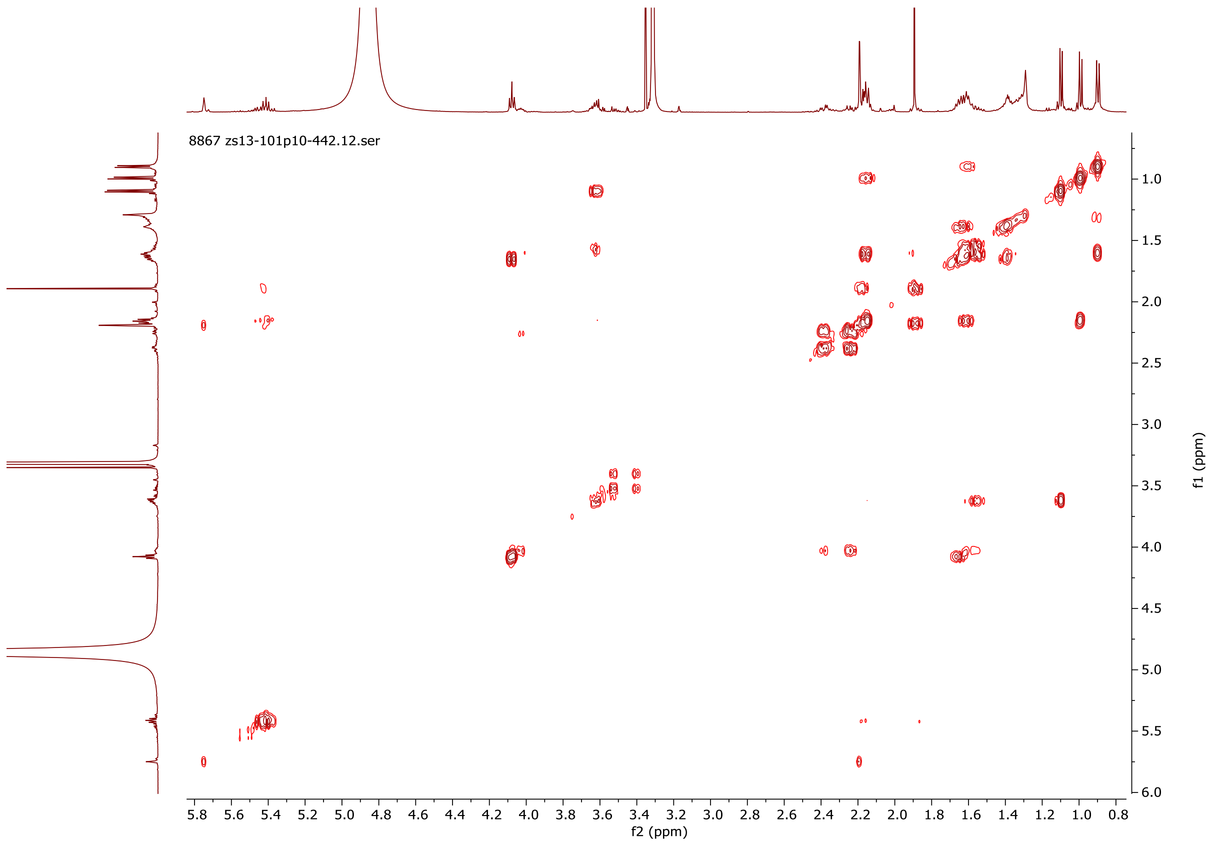
8867 zs13-101p10-442.12.ser
f2 (ppm)
f1 (ppm)

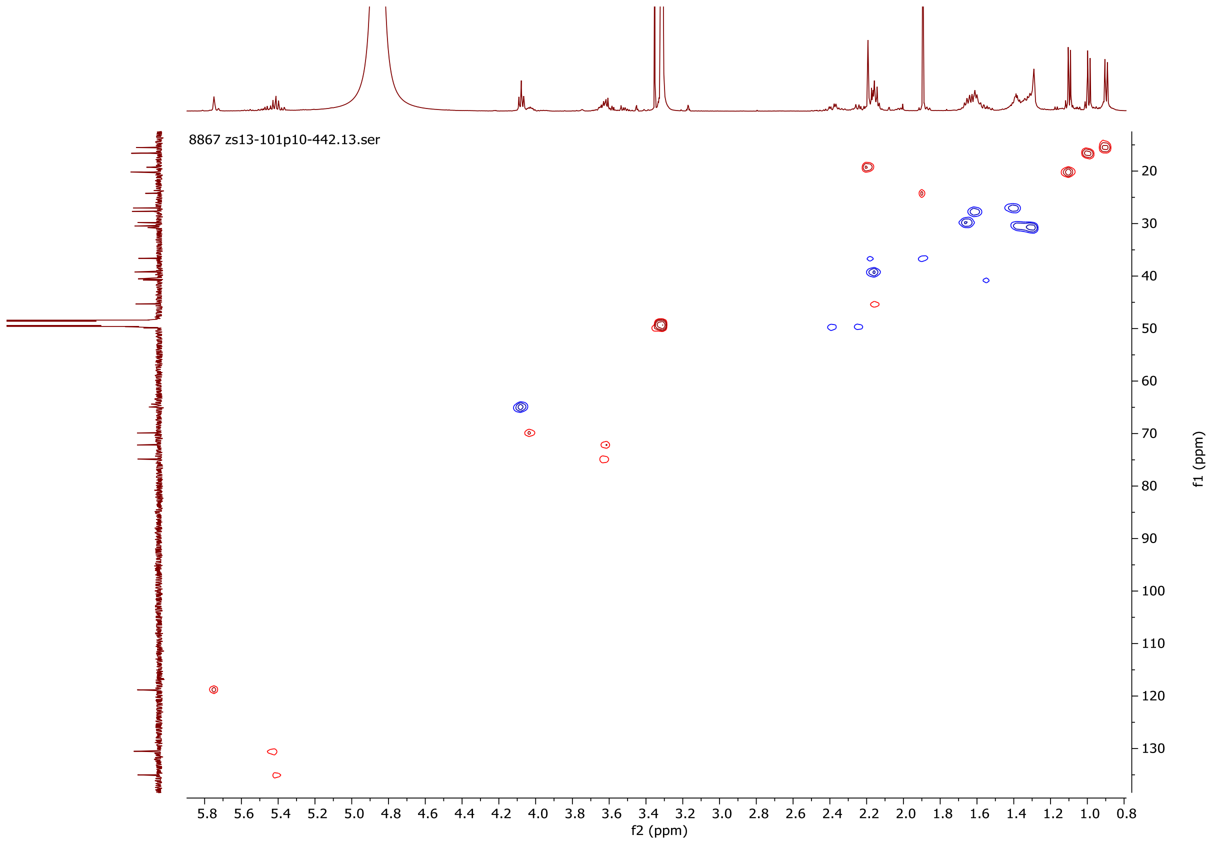
8867 zs13-101p10-442.13.ser
f2 (ppm)
f1 (ppm)

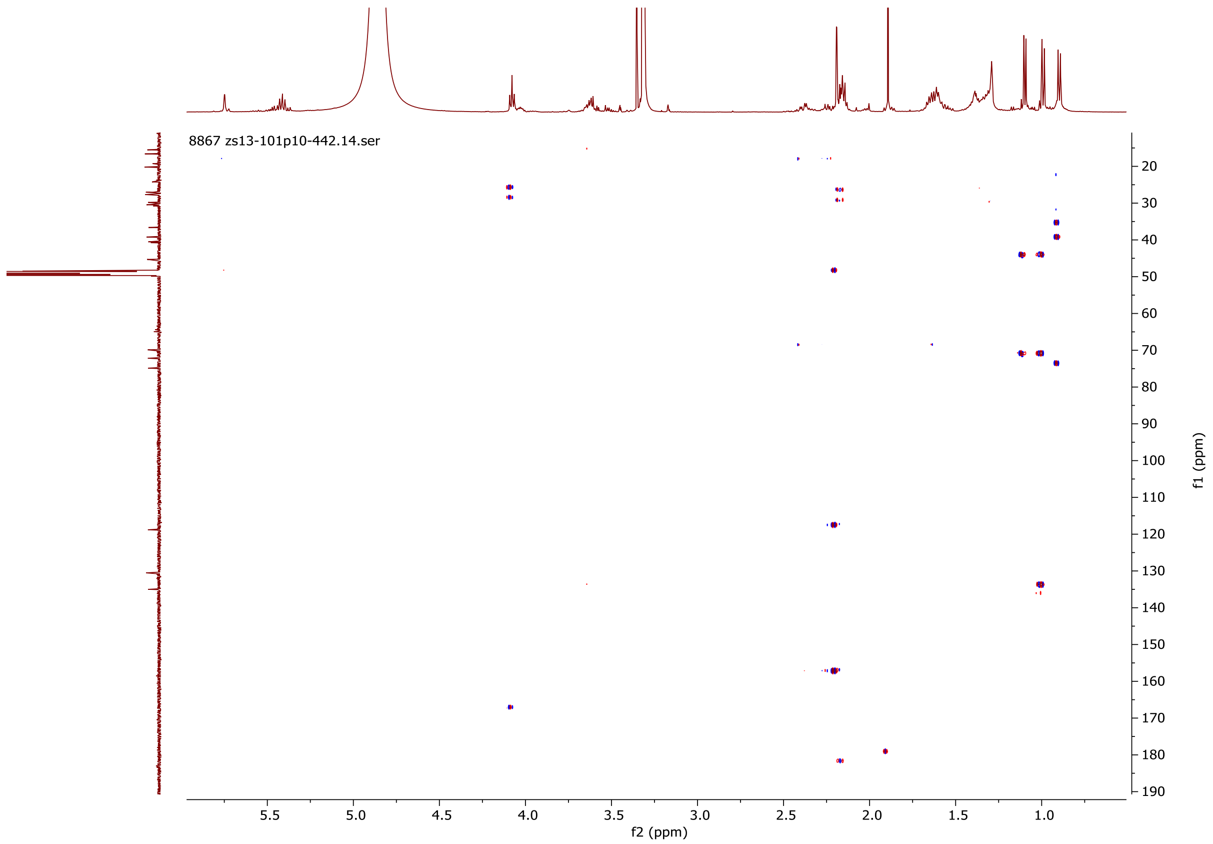
8867 zs13-101p10-442.14.ser
f2 (ppm)
f1 (ppm)

Figure S49. ^{1}H (first), ^{1}H-^{1}H COSY (second), HSQC (third-fourth) and HMBC (fifth) NMR spectra of Mupirocin A7

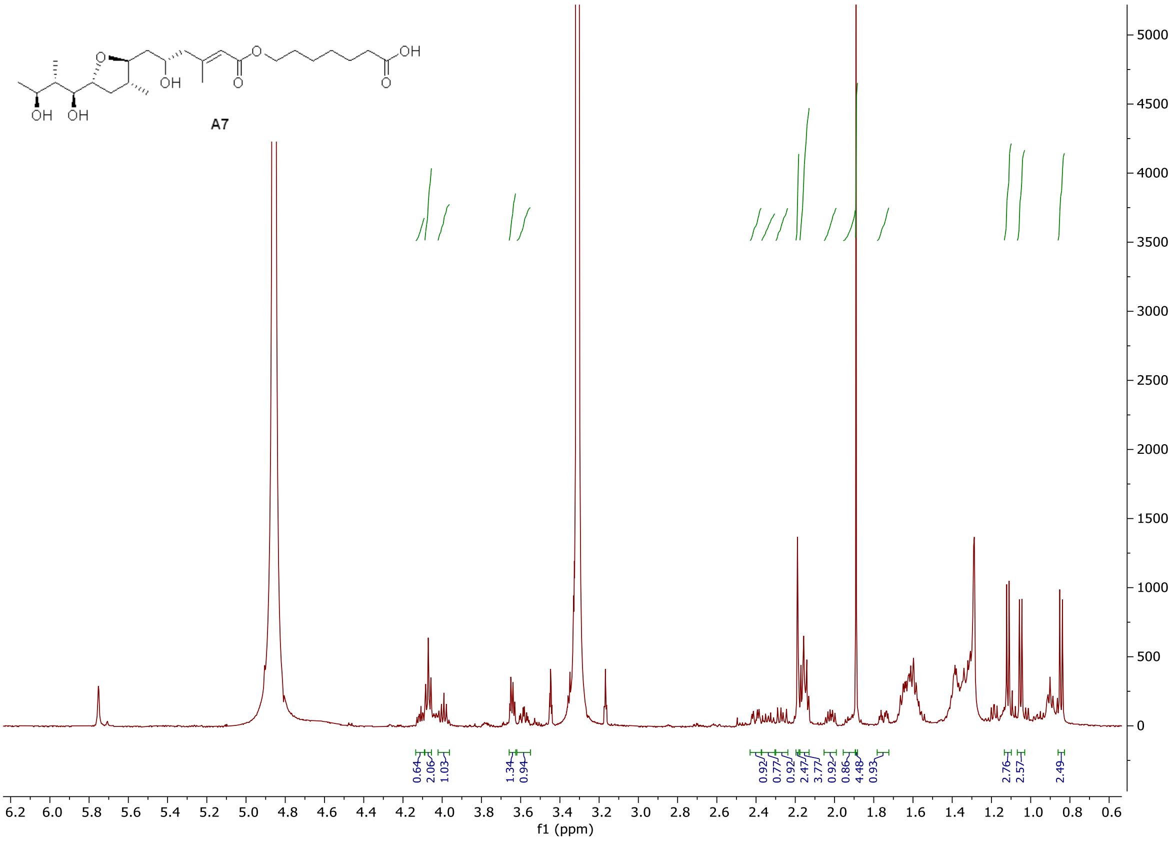

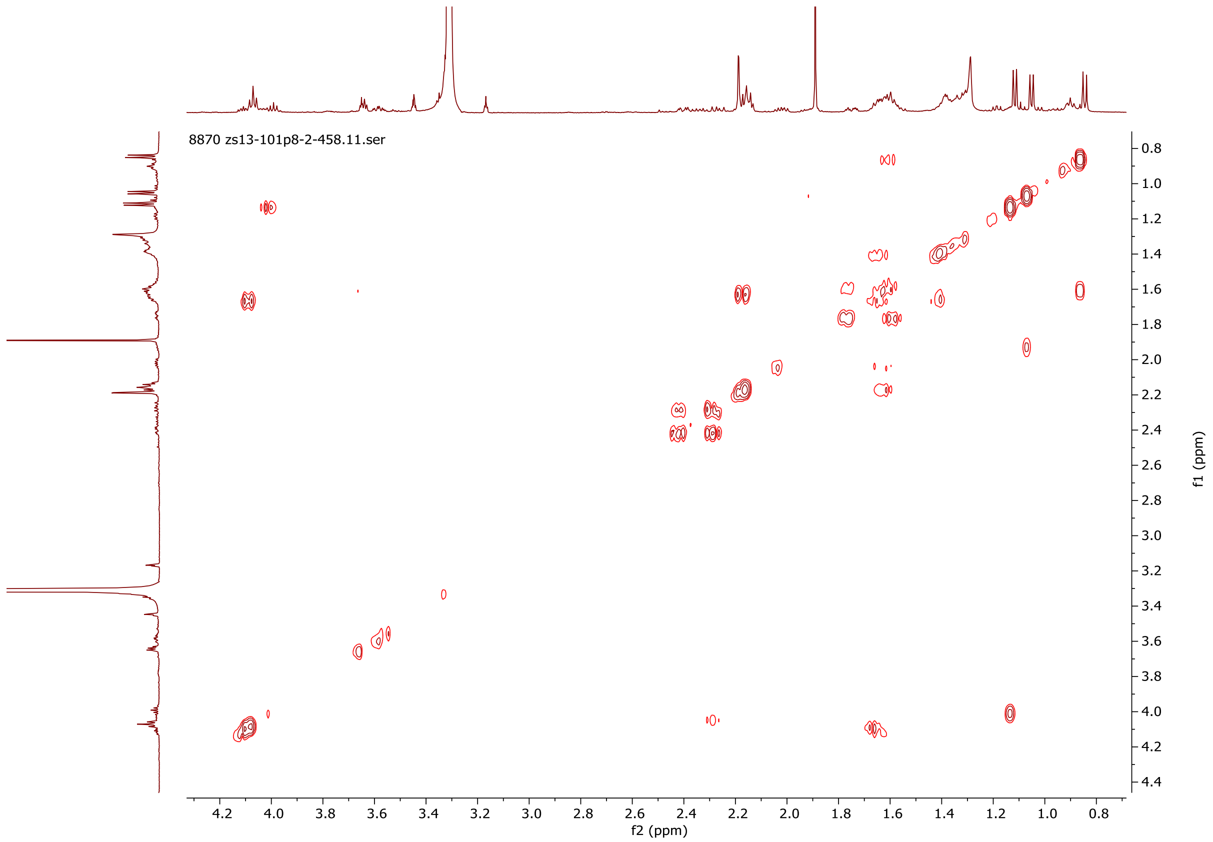
8870 zs13-101p8-2-458.11.ser
f2 (ppm)
f1 (ppm)

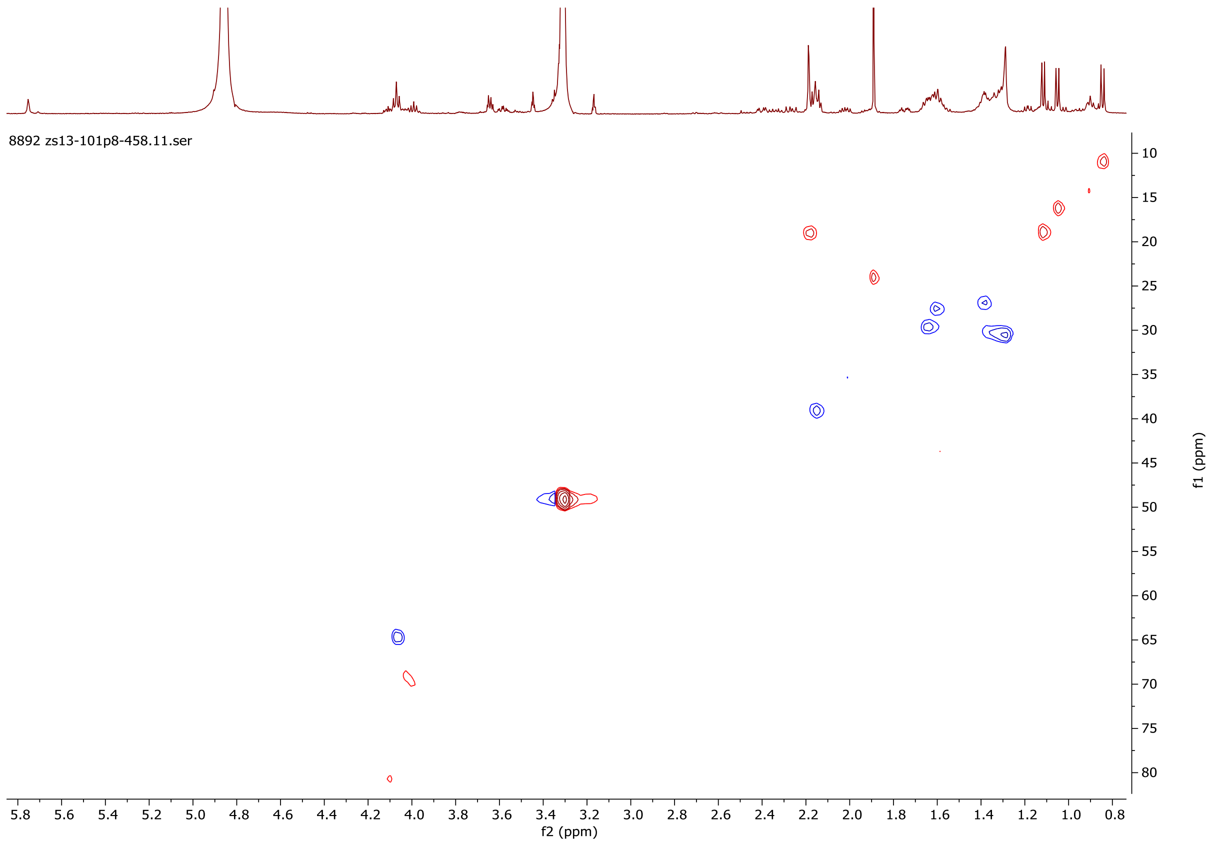
8892 zs13-101p8-458.11.ser
f2 (ppm)
f1 (ppm)

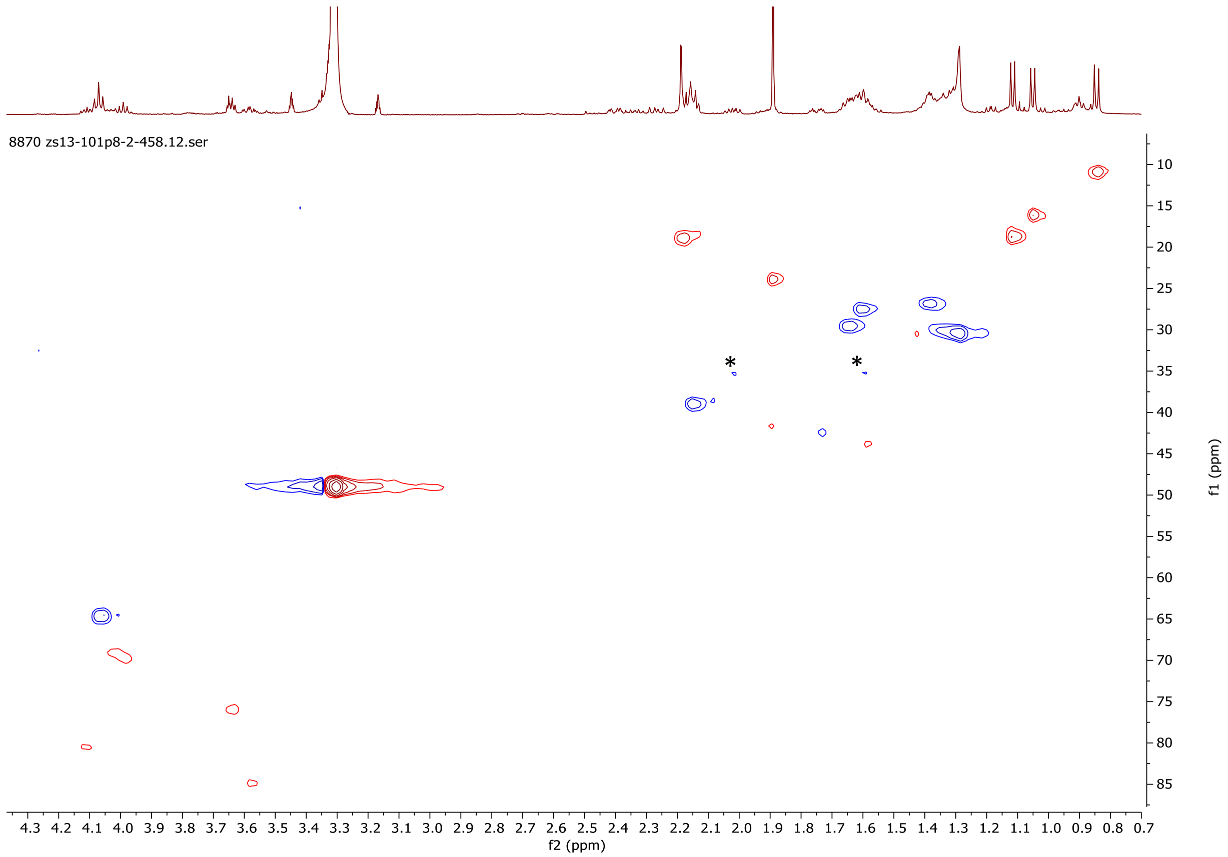

Expansion of HSQC for mupirocin **A7** showing weak correlations for C9 (*).

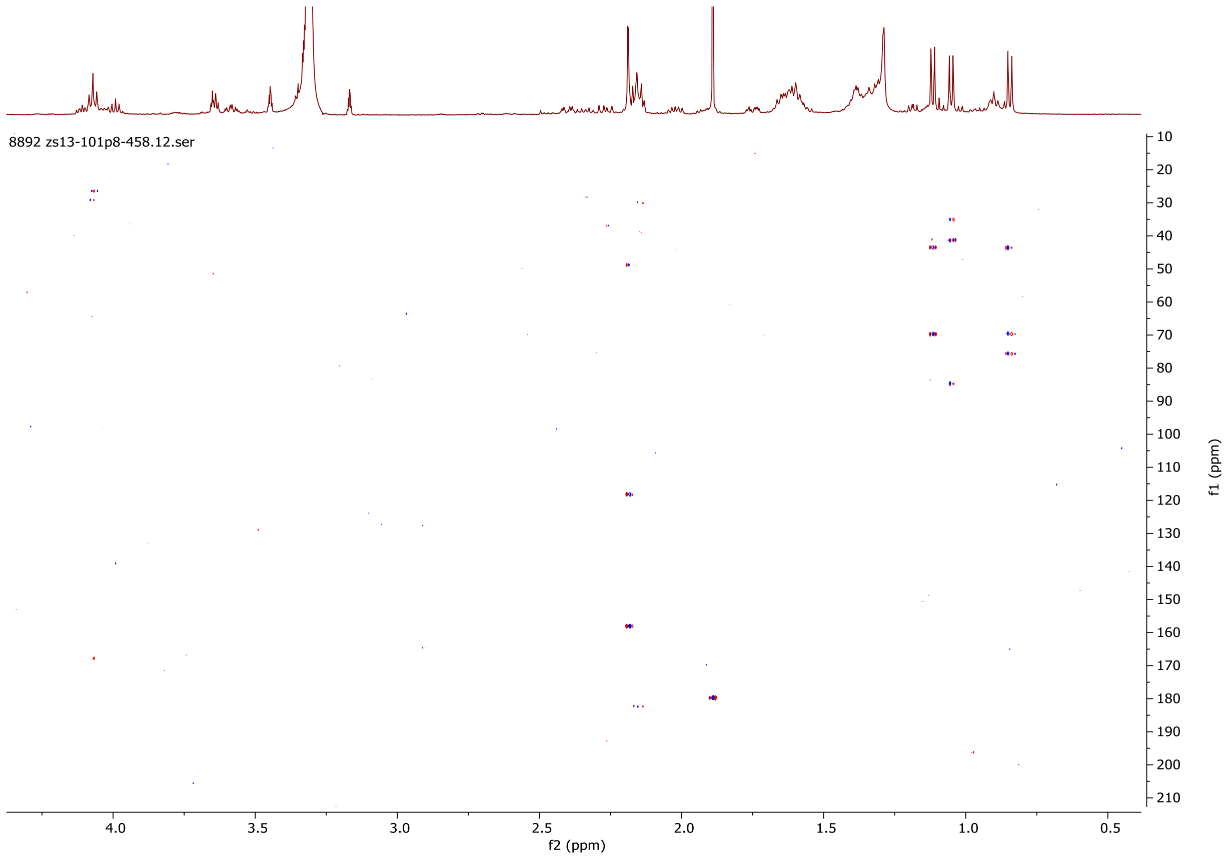
8892 zs13-101p8-458.12.ser
f2 (ppm)
f1 (ppm)
4.0
3.5
3.0
2.5
2.0
1.5
1.0
0.5
10
20
30
40
50
60
70
80
90
100
110
120
130
140
150
160
170
180
190
200
210

List of sequences

MupA

MAHHHHHHSSGLEVLFQGPMSVEQLLGLGVTNAARAPRQPAFSLLFFSDVREDISASEKYEFARSLTLFGD
AQGFEAVYFPERHFHEFGAIYPDSAVMAASLIPQTRHIRFRTAGISLPLHHPARVVESWSMNDVLSGGRVD
LGFGSGWSRPDFLLAPHAYEDRREVMWQRIEQVRRLWAGERLSFPGPGGDPVSVVTFPRPLQTSLNVWILV
AQNTESFIAAGKAGFNVFTMLYGIDLPDLAEKIGLYRQARRDAGFDPASGRVTLMLHTLVGPDSAWVRRAV
EAPFKDYIRSSLVAHMKARATPDGRPLDATEQENVLQYAFERYYETGALFGTVDEVRYRVGQVLETGVDEI
ACLMDFGVDYAVVHQSLPYLEQLVASFQERSAQ

Calc Mw (-M): 43,292.96 Da

E.coli Fre

MHHHHHHGKPIPNPLLGLDSTENLYFQGIDPFTMTTLSCKVTSVEAITDTVYRVRIVPDAAFSFRAGQYLM
VVMDERDKRPFSMASTPDEKGFIELHIGASEINLYAKAVMDRILKDHQIVVDIPHGEAWLRDDEERPMILI
AGGTGFSYARSILLTALARNPNRDITIYWGGREEQHLYDLCELEALSLKHPGLQVVPVVEQPEAGWRGRTG
TVLTAVLQDHGTLAEHDIYIAGRFEMAKIARDLFCSERNAREDRLFGDAFAFI

Calc Mw: 30025.23 Da

MmpA_KS0

MHHHHHHGKPIPNPLLGLDSTENLYFQGIDPFTEPVAIIGLSANVAQSASVRQFWQALDDDRSLIEEIPAT
RFDFTSWYAGSNIEEGKMRTRWGGFIPAIDQFDPVFFGMLPAEARKMDPQQRLLLMSVRQTFEDAGYRHTD
WKGSATGVFIAAERNEYHLNLLQAQIDPGEGLDQAASMLANRVSHFYDLRGPSERIDAMCAGGAVALHHAV
TALRSGQINAAIVGACNLLLRPDVFVTLSQSGQMSPEPTVRSFGAGADGYLRGEGVCSLLLKPLSKAEADG
DHIYGLIRNTAVNYNGGDAASIAAPSVSAHSSLVQDCYRRAGIDPRHVSYIEAQGMGNPVADIAEWDALNH
GLLALGREQGVQLQEGQCAISTLKPMSGHMHAASAIGALFKIIRSLQTEKIHKILDFEQPNLHLHTAGQPC
RLATHTVDWPRQATPRLAGLHSYGAGGNNAHILVEEYVHQAPGRVVPSQAPLLFPLSAPTPALLALLAAQM
HQALIDEPTLCLESVSDTLKFGREKFAAAVVIVAMERPGLLDVLAQLHTGDLTGAVVFAKDVAAPTDAALD
PWAELWLAGERPMAKATRAPRVPLPTTPFDTQSFW

Calc Mw = 65261.2 Da

ACP_D4.6His

MHHHHHHGKPIPNPLLGLDSTENLYFQGIDPFTPSAPRPATVGAGALLEQVREVIERVLVVDEPDLDTAFS
RYGMDSVGAMQVSSALSRALGWLVEPRWLVQHATIRALAEFLQSRNEAATQ

Calc Mw = 13457.23 Da

ACP_D4 (His$_6$ cleaved)

GIDPFTPSAPRPATVGAGALLEQVREVIERVLVVDEPDLDTAFSRYGMDSVGAMQVSSALSRALGWLVEPR
WLVQHATIRALAEFLQSRNEAATQ

Calc Mw = 10304.69 Da

ACP_A1.6His

MHHHHHHGKPIPNPLLGLDSTENLYFQGIDPFTRAWSPDAAEQAVREALAQALEQPAASLDLDAQFSELGF
DSMMVRQLCRHMRDQDIVVEPAVLFEHATPARLVAWLACAPAQ

Calc Mw = 12715.40 Da

ACP_A1 (His$_6$ cleaved)

GIDPFTRAWSPDAAEQAVREALAQALEQPAASLDLDAQFSELGFDSMMVRQLCRHMRDQDIVVEPAVLFEH
ATPARLVAWLACAPAQ

Calc Mw = 9562.85 Da

References

[1] M. J. Burk, J. E. Feaster, R. L. Harlow, *Tetrahedron: Asymmetry* **1991**, *2*, 569-592.
[2] M. Jacolot, M. Jean, N. Levoin, P. van de Weghe, *Org. Lett.* **2012**, *14*, 58-61.
[3] C. Stanley, H. Soo, B. Robert, *Angew. Chem. Int. Ed.* **2015**, *54*, 211-214.
[4] H. J. Bestmann, B. Liepold, A. Kress, A. Hofmann, *Chem. Eur. J.* **1999**, *5*, 2984-2989.
[5] M. Grote, S. Kushnir, N. Pryk, D. Möller, J. Erver, A. Ismail-Ali, F. Schulz, *Org. Biomol. Chem.* **2019**, *17*, 6374-6385.
[6] S. Sengupta, T. Sim, *Eur. J. Org. Chem.* **2014**, *2014*, 5063-5070.
[7] Z. Gao, J. Wang, A. K. Norquay, K. Qiao, Y. Tang, J. C. Vederas, *J. Am. Chem. Soc.* **2013**, *135*, 1735-1738.
[8] J. Kerhervé, C. Botuha, J. Dubois, *Org. Biomol. Chem.* **2009**, *7*, 2214-2222.
[9] A. L. Sewell, M. V. J. Villa, M. Matheson, W. G. Whittingham, R. Marquez, *Org. Lett.* **2011**, *13*, 800-803.
[10] P. D. Walker, C. Williams, A. N. M. Weir, L. Wang, J. Crosby, P. R. Race, T. J. Simpson, C. L. Willis, M. P. Crump, *Angew. Chem. Int. Ed.* **2019**, *58*, 12446-12450.
[11] L. M. Stunkard, A. D. Dixon, T. J. Huth, J. R. Lohman, *J. Am. Chem. Soc.* **2019**, *141*, 5121-5124.
[12] aA. S. Haines, X. Dong, Z. Song, R. Farmer, C. Williams, J. Hothersall, E. Płoskoń, P. Wattana-Amorn, E. R. Stephens, E. Yamada, R. Gurney, Y. Takebayashi, J. Masschelein, R. J. Cox, R. Lavigne, C. L. Willis, T. J. Simpson, J. Crosby, P. J. Winn, C. M. Thomas, M. P. Crump, *Nat. Chem. Biol.* **2013**, *9*, 685-692; bV. Agarwal, S. Diethelm, L. Ray, N. Garg, T. Awakawa, P. C. Dorrestein, B. S. Moore, *Org. Lett.* **2015**, *17*, 4452-4455.
[13] S. Tayyab, S. Qamar, M. Islam, *Biochem. Educ.* **1991**, *19*, 149-152.
[14] P. D. Walker, M. T. Rowe, A. J. Winter, A. N. M. Weir, N. Akter, L. Wang, P. R. Race, C. Williams, Z. Song, T. J. Simpson, C. L. Willis, M. P. Crump, *ACS Chem. Biol.* **2020**, *15*, 494-503.
[15] M. J. L. J. Fürst, A. Gran-Scheuch, F. S. Aalbers, M. W. Fraaije, *ACS Catal.* **2019**, *9*, 11207-11241.
[16] J. Jumper, R. Evans, A. Pritzel, T. Green, M. Figurnov, O. Ronneberger, K. Tunyasuvunakool, R. Bates, A. Žídek, A. Potapenko, A. Bridgland, C. Meyer, S. A. A. Kohl, A. J. Ballard, A. Cowie, B. Romera-Paredes, S. Nikolov, R. Jain, J. Adler, T. Back, S. Petersen, D. Reiman, E. Clancy, M. Zielinski, M. Steinegger, M. Pacholska, T. Berghammer, S. Bodenstein, D. Silver, O. Vinyals, A. W. Senior, K. Kavukcuoglu, P. Kohli, D. Hassabis, *Nature* **2021**, *596*, 583-589.
[17] C. Notredame, D. G. Higgins, J. Heringa, *J. Mol. Biol.* **2000**, *302*, 205-217.
[18] X. Robert, P. Gouet, *Nucleic Acids Res.* **2014**, *42*, W320-W324.
[19] J. Mareš, J. Hájek, P. Urajová, J. Kopecký, P. Hrouzek, *PLoS One* **2014**, *9*, e111904.
[20] R. A. Meoded, R. Ueoka, E. J. N. Helfrich, K. Jensen, N. Magnus, B. Piechulla, J. Piel, *Angew. Chem. Int. Ed.* **2018**, *57*, 11644-11648.
[21] S. F. Altschul, W. Gish, W. Miller, E. W. Myers, D. J. Lipman, *J. Mol. Biol.* **1990**, *215*, 403-410.
[22] L. J. McGuffin, K. Bryson, D. T. Jones, *Bioinformatics* **2000**, *16*, 404-405.
[23] B. Mészáros, G. Erdős, Z. Dosztányi, *Nucleic Acids Res.* **2018**, *46*, W329-W337.
